# Supplementary material for: RhoGDI phosphorylation by PKC promotes its interaction with death receptor p75NTR to gate axon growth and neuron survival
Source: EMBO Rep. 2024 Jan 22;25(3):30. doi: 10.1038/s44319-024-00064-2 (PMC10933337; doi:10.1038/s44319-024-00064-2)
Supplement: Supplementary file 1 — Table EV1 [file 44319_2024_64_MOESM1_ESM.pdf]

Table S1. NMR and refinement statistics for p75^{NTR} JXT:RhoGDI NTD^{S34D}

**Parameters**	
**NMR distance & dihedral constraints**	
Distance constraints	
Total NOE	1477
Intra-residue	722
Inter-residue	
Sequential (|i-j| = 1)	498
Medium-range (|i-j| ≤ 4)	188
Long-range (|i-j ≥ 5)	12
Intermolecular NOE	54
Total dihedral angle restraints[a]	121
**Structure Statistics**	
Violations (mean and s.d.)	
Distance constraints (Å)	0.36±0.05
Dihedral angle constraints (º)	2.88±0.76
Max. dihedral angle violation (º)	4.53
Max. distance constraint violation (Å)	0.44
Ramachandran Plot[b]	
Most favoured regions	59.7%
Additional allowed regions	33.8%
Generously allowed regions	6.5%
Disallowed regions	0.0%
Average RMSD (Å)[c]	
Backbone atoms	0.84±0.17
Heavy atoms	1.33±0.19

[a]Dihedral angle constraints were generated by TALOS based on Cα and Cβ chemical shifts.

[b]The selected residues are 8-17,32-57 of RhoGDI NTD^{S34D} and 291-322 of p75^{NTR} JXT in the non-disordered region.

[c]Average r.m.s. deviation (RMSD) to the mean structure was calculated among 10 refined structures.
