# Supplementary material for: RhoGDI phosphorylation by PKC promotes its interaction with death receptor p75NTR to gate axon growth and neuron survival
Source: EMBO Rep. 2024 Jan 22;25(3):30. doi: 10.1038/s44319-024-00064-2 (PMC10933337; doi:10.1038/s44319-024-00064-2)
Supplement: Supplementary file 2 — Table EV2 [file 44319_2024_64_MOESM2_ESM.pdf]

Table EV2. Summary of plasmids used in the study

Name	backbone	Reporter/tag	Description
pCDNA5-p75^{NTR}	pCDNA5-FRT-TO	None	Mammalian overexpression vector for mouse p75^{NTR}.
p75^{NTR} V246N	pCDNA5-FRT-TO	None	Mammalian overexpression vector for γ-secretase uncleavable mutant variant of mouse p75^{NTR} with V246N mutation to prevent γ-secretase mediated cleavage.
p75^{NTR} JXT	pCDNA5-FRT-TO<br>pCMV3-N-Flag	N-HA<br>C-GST	Mammalian overexpression vector for expressing juxtamembrane region of mouse p75^{NTR}.
pCMV-RhoGDI	pCMV3-N-Flag	N-Flag	Mammalian expression vector with CMV promoter for mouse RhoGDI.
RhoGDI-59	pCMV3-N-Flag	N-Flag<br>C-GST	Mammalian overexpression vector for N-flag and C-GST tagged first 59 residues of mouse RhoGDI.
RhoGDI-Δ59	pCMV3-N-Flag	N-Flag	Mammalian overexpression vector for N-flag tagged mouse RhoGDI lacking the N-terminus 59 residues.
pHAGE-IRES-eGFP	pHAGE	eGFP	Empty lentiviral vector containing enhanced (CMV enhancer) human synapsin I promoter for neuronal expression.
pHAGE-p75^{NTR}	pHAGE	eGFP	Neuronal overexpression vector for EGFP fusion mouse p75^{NTR}
pHAGE-p75^{NTR}K303A	pHAGE	eGFP	Neuronal overexpression vector for a mutant variant of mouse p75^{NTR} with K303A mutation in p75^{NTR} juxtamembrane.
pHAGE-RhoGDI	pHAGE	eGFP	Neuronal overexpression vector for EGFP fusion mouse RhoGDI.
pHAGE-RhoGDI S34D	pHAGE	eGFP	Neuronal overexpression vector for a mutant variant of mouse RhoGDI with S34D mutation in RhoGDI.
