# Supplementary material for: RhoGDI phosphorylation by PKC promotes its interaction with death receptor p75NTR to gate axon growth and neuron survival
Source: EMBO Rep. 2024 Jan 22;25(3):30. doi: 10.1038/s44319-024-00064-2 (PMC10933337; doi:10.1038/s44319-024-00064-2)
Supplement: Supplementary file 3 — Table EV3 [file 44319_2024_64_MOESM3_ESM.pdf]

Table EV3. Summary of antibodies used in the study

Antibodies	Catalog No.	Company	Host	Description
$p75^{NTR}$	GT15057	Neuromics	Goat	Western blot
$p75^{NTR}$	ANT-007	Alomone	Rabbit	Immunoprecipitation
$p75^{NTR}$	ANT-011	Alomone	Rabbit	Used in PLA and Co-IP pulldown of $p75^{NTR}$ V246N mutant.
Flag	F3165-1MG	Sigma	Mouse	Western blot
HA	H3663	SIGMA	Mouse	Western blot
RhoGDI-alpha	SC373724	Santa Cruz	Mouse	Western blot
RhoGDI-alpha	2564S	Cell Signaling Technologies	Rabbit	Western blot
RhoA	2117S	CST	Rabbit	Immunoprecipitation and western blot
p-(s)PKC Substrate	2261S	CST	Rabbit	Western blot
Anti- c-Myc	11667149001	Merck (Sigma)	Mouse	Western blot
RIP2(m)	612349	BD	Mouse	Western blot
TRAF6(H274)	SC7221	SC	Rabbit	Western blot
GAPDH	G9545	Sigma	Rabbit	Western blot
Cleaved caspase-3	9661L	CST	Rabbit	Immunofluorescence microscopy
TUJ1(b-Tubulin)		Covance	Mouse	Immunofluorescence microscopy
MAP2	Ab5392	Abcam	Chicken	Immunofluorescence microscopy
p-Ser-96-RhoGDI		Sino biologicals	Rabbit	Western blot
