# Supplementary material for: RhoGDI phosphorylation by PKC promotes its interaction with death receptor p75NTR to gate axon growth and neuron survival
Source: EMBO Rep. 2024 Jan 22;25(3):30. doi: 10.1038/s44319-024-00064-2 (PMC10933337; doi:10.1038/s44319-024-00064-2)
Supplement: Supplementary file 4 — Appendix [file 44319_2024_64_MOESM4_ESM.pdf]

Appendix to Ramanujan et al. "RhoGDI phosphorylation by PKC regulates its interaction with death receptor p75^{NTR} to gate axon growth and neuron survival pathways"

Table of contents:

Appendix Figure S1……………………………………………………page 2

Appendix Figure S2……………………………………………………page 3

Appendix Figure S3……………………………………………………page 4

Appendix Figure S4……………………………………………………page 5

Appendix Figure S5……………………………………………………page 6

Appendix Figure S6……………………………………………………page 7

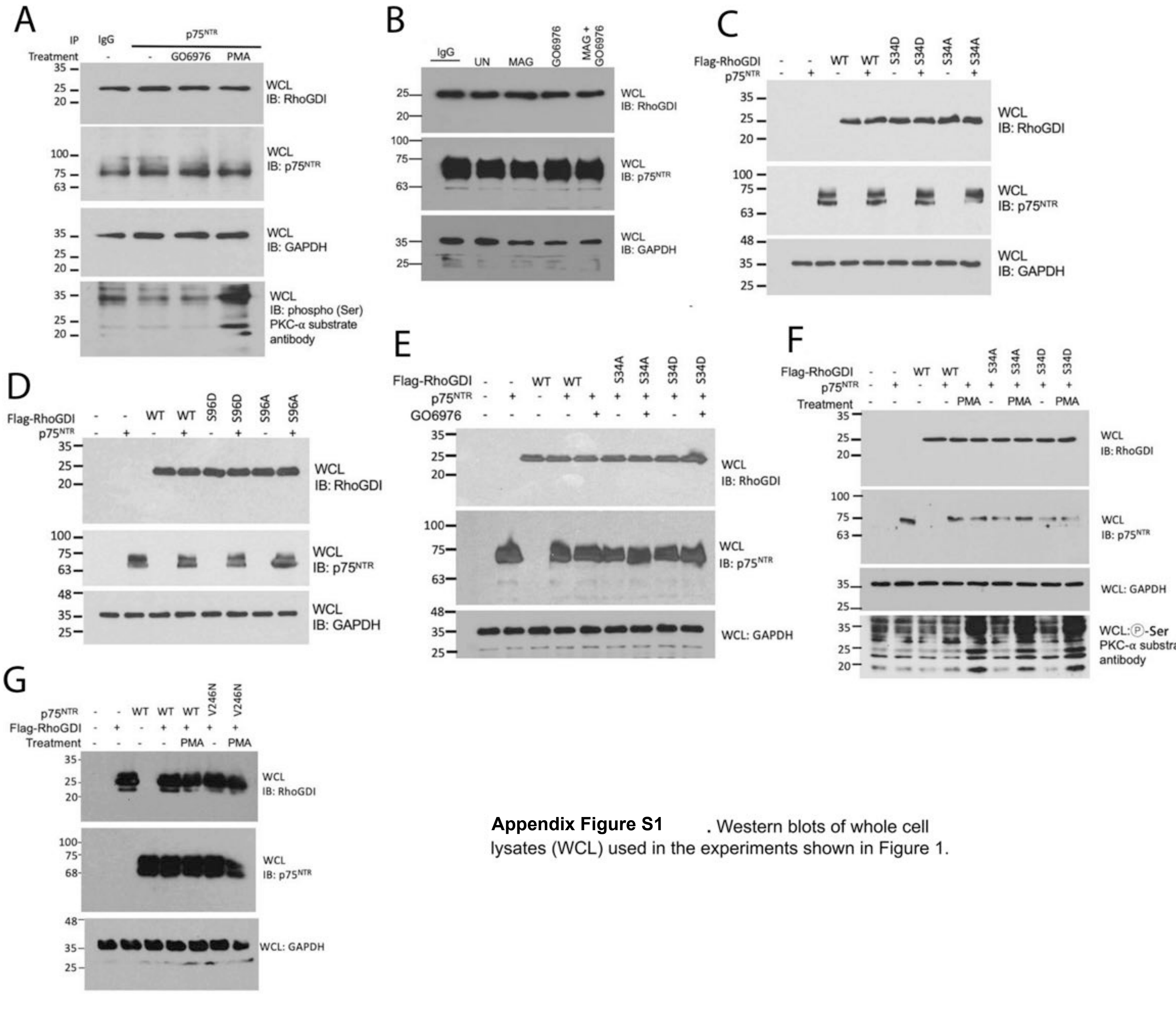

Appendix Figure S1. Western blots of whole cell lysates (WCL) used in the experiments shown in Figure 1.

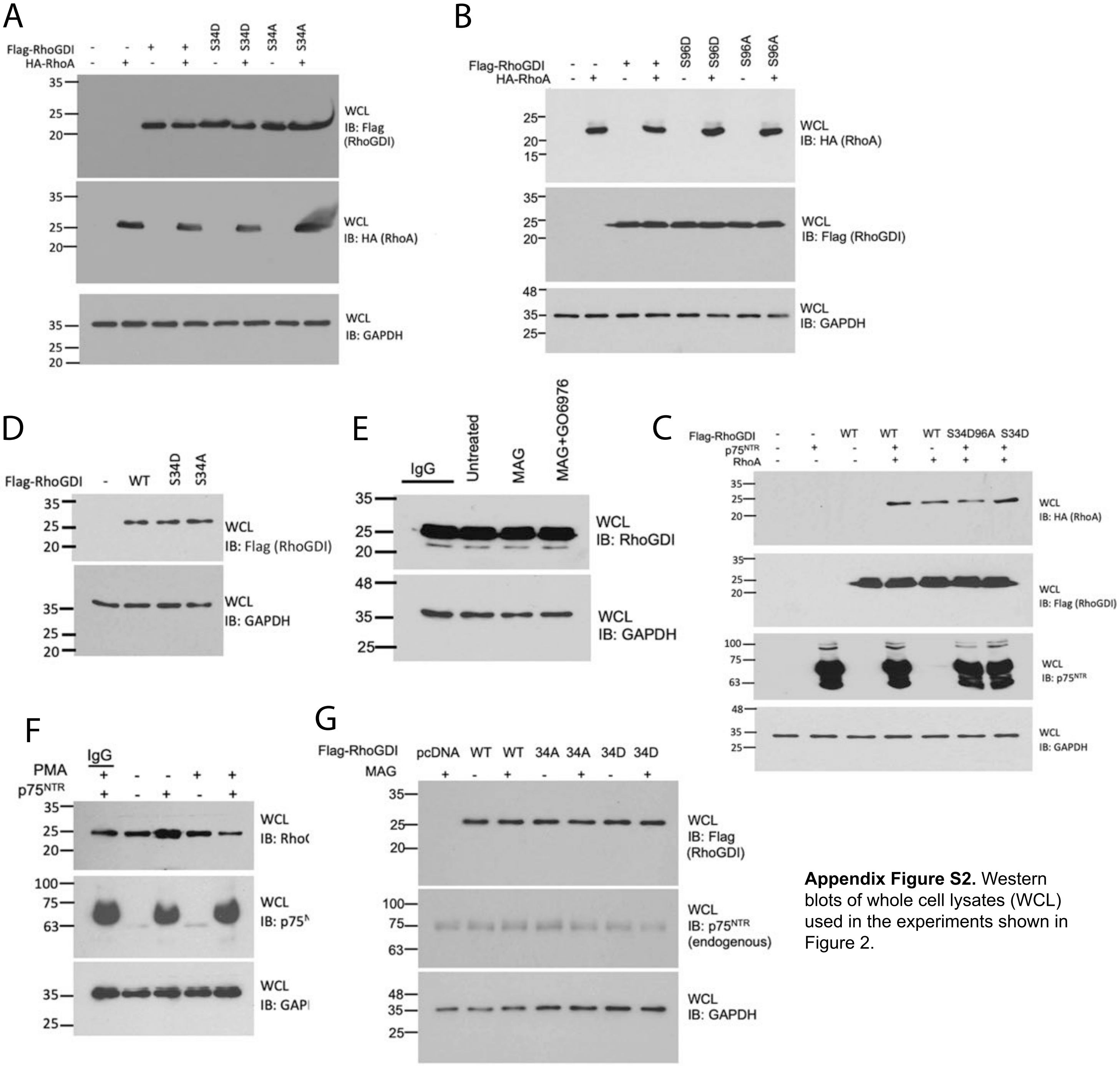

Appendix Figure S2. Western blots of whole cell lysates (WCL) used in the experiments shown in Figure 2.

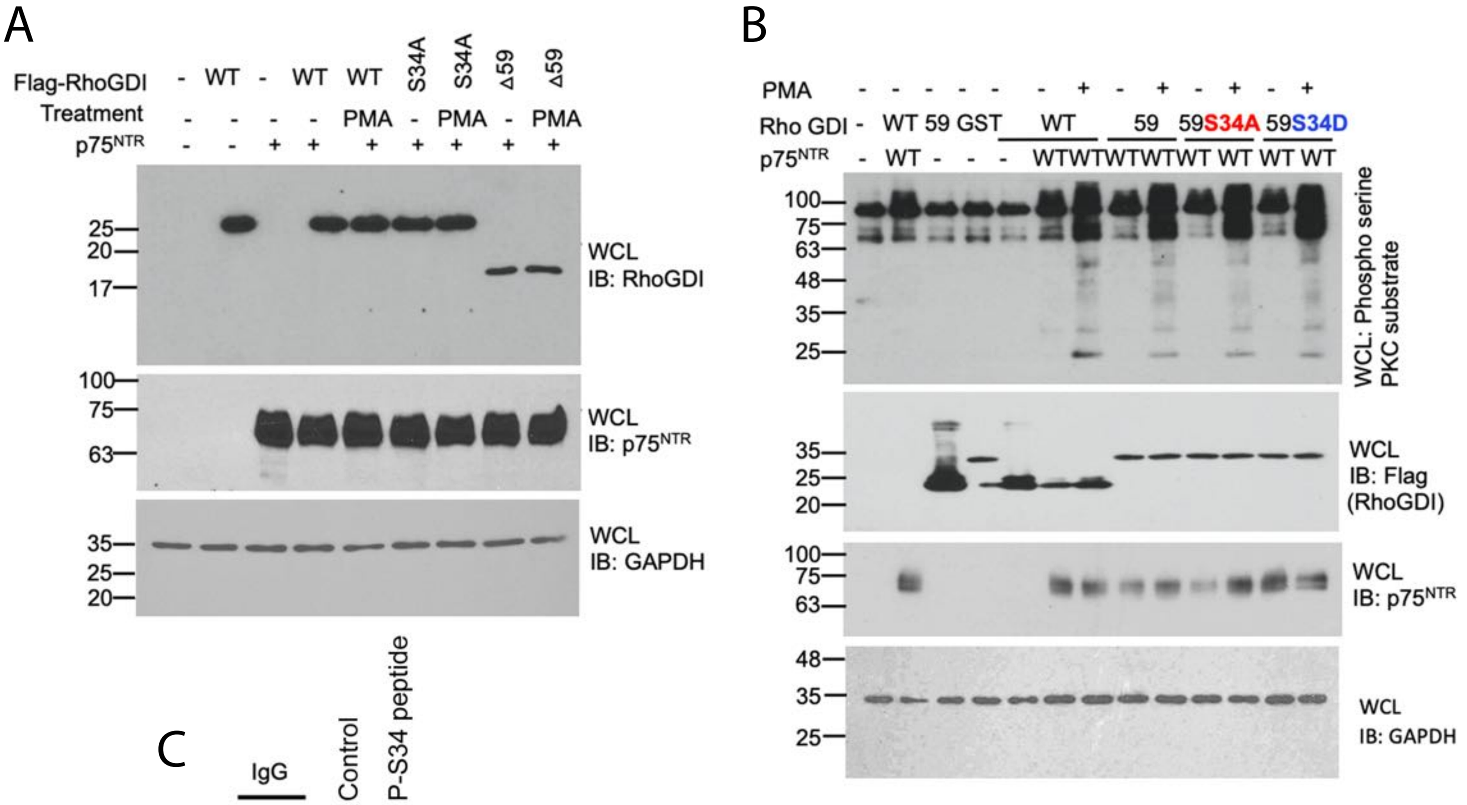

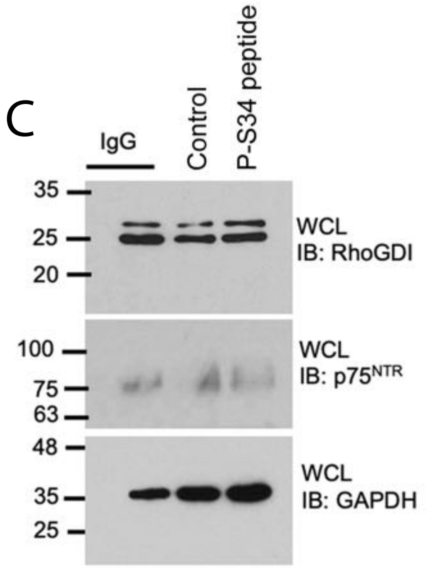

Appendix Figure S3. Western blots of whole cell lysates (WCL) used in the experiments shown in Figure 3.

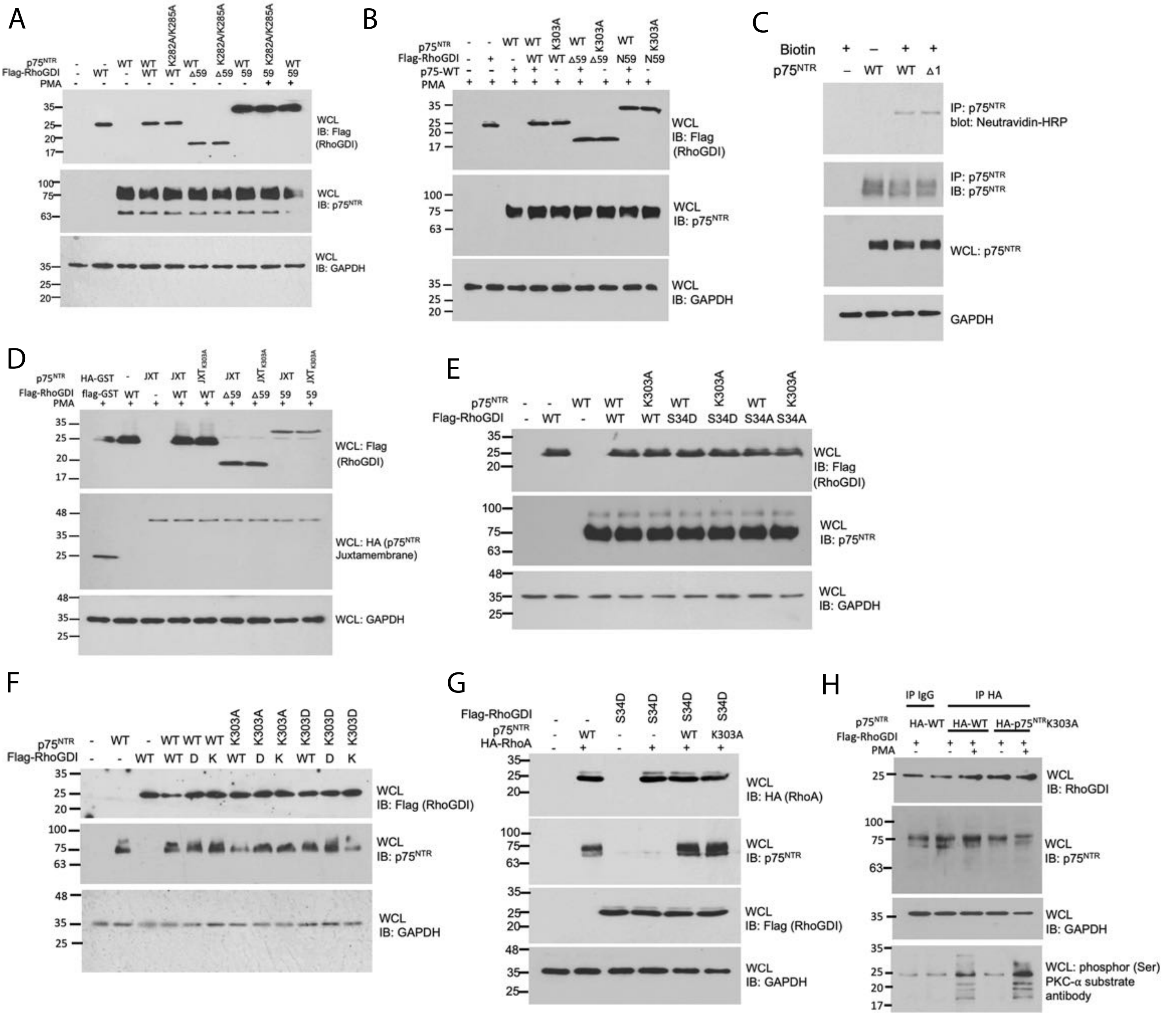

Appendix Figure S4.

(A, B) Western blots of whole cell lysates (WCL) used in the experiments shown in Figure 4.

(C) Cell surface biotinylation of NIH3T3 cells expressing wild type (WT) $p75^{NTR}$ or deletion mutant lacking first 10 residues in JXT (Δ1), including Lys^{303} followed by immunoprecipitation for $p75^{NTR}$. The result shows normal cell surface expression of the mutant.

(D-H) Western blots of whole cell lysates (WCL) used in the experiments shown in Figure 4.

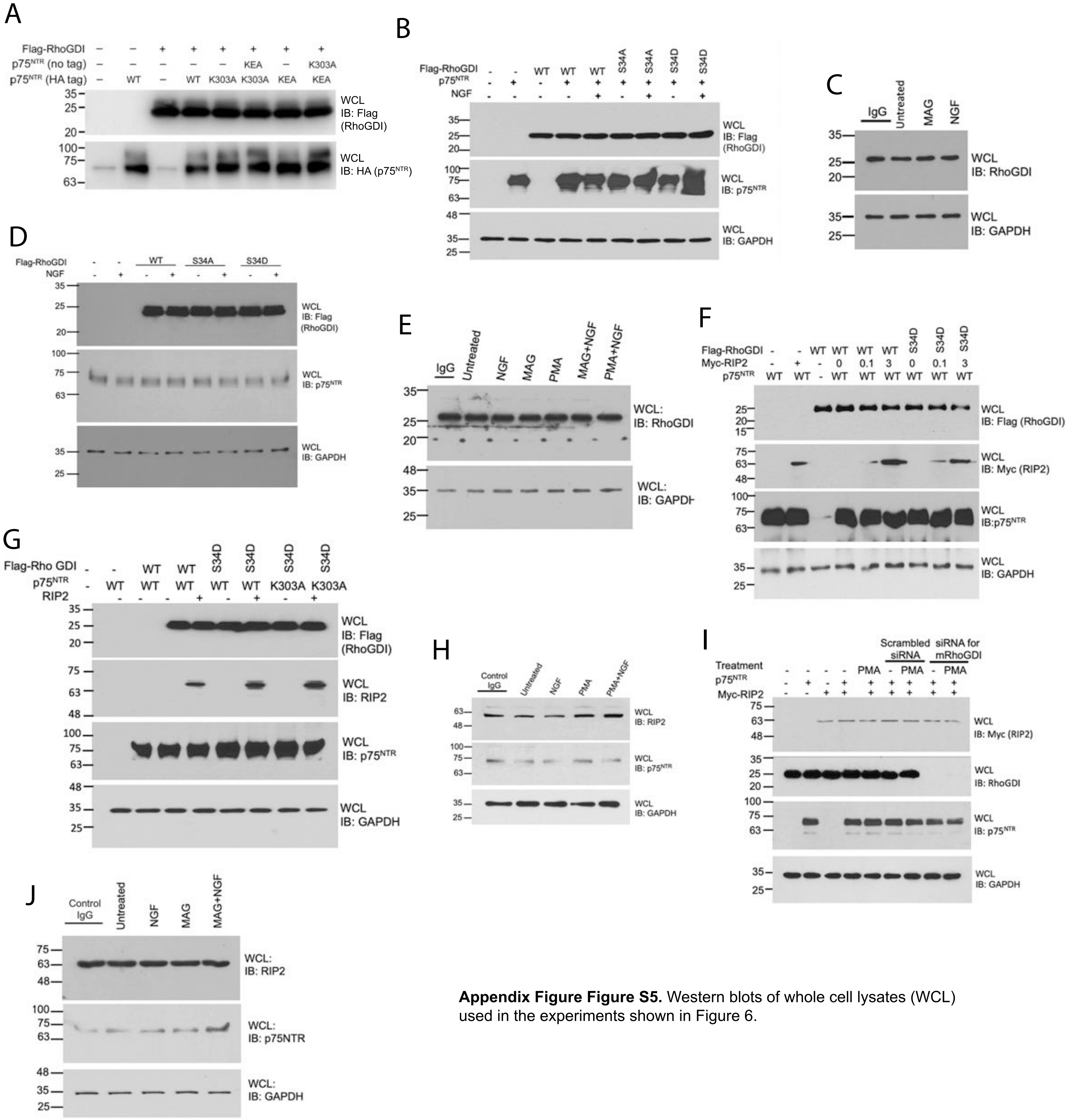

Appendix Figure Figure S5. Western blots of whole cell lysates (WCL) used in the experiments shown in Figure 6.

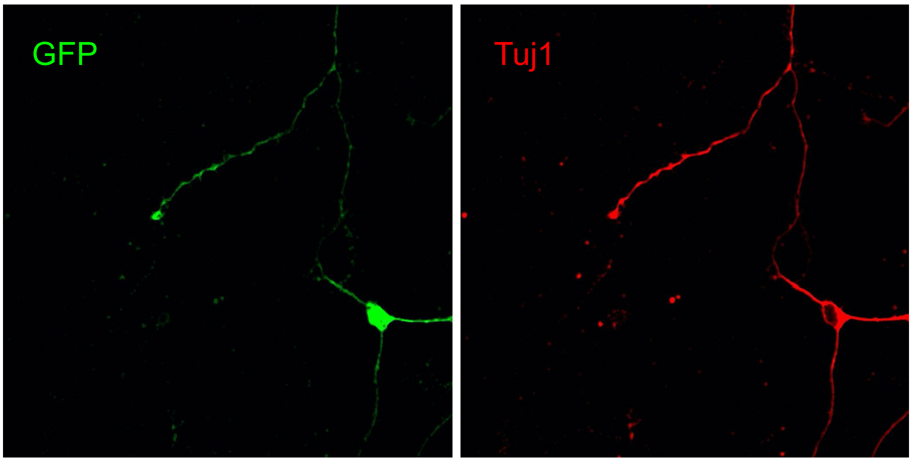

Appendix Figure S6. GFP fluorescence and counterstaining with Tuj1 antibodies of neuron and growth cone from Figure 7B showing axonal integrity.
