# Supplementary figures and images for: RhoGDI phosphorylation by PKC promotes its interaction with death receptor p75NTR to gate axon growth and neuron survival (part 1 of 3)
Source: EMBO Rep. 2024 Jan 22;25(3):30. doi: 10.1038/s44319-024-00064-2 (PMC10933337; doi:10.1038/s44319-024-00064-2)

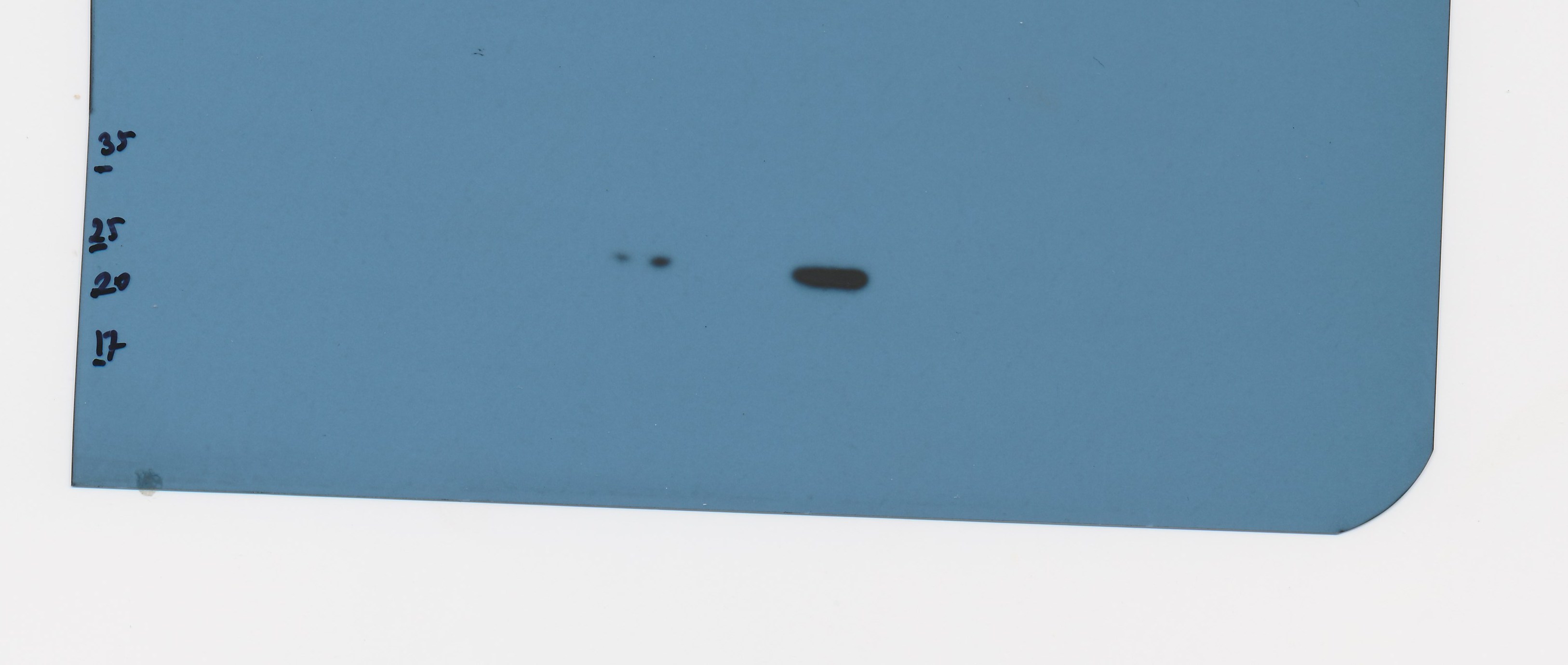

Supplement: Supplementary file 5 — Source Data Fig. 1 [file 44319_2024_64_MOESM5_ESM.zip › 1A/IP p75NTR:IB RhoGDI.jpg]

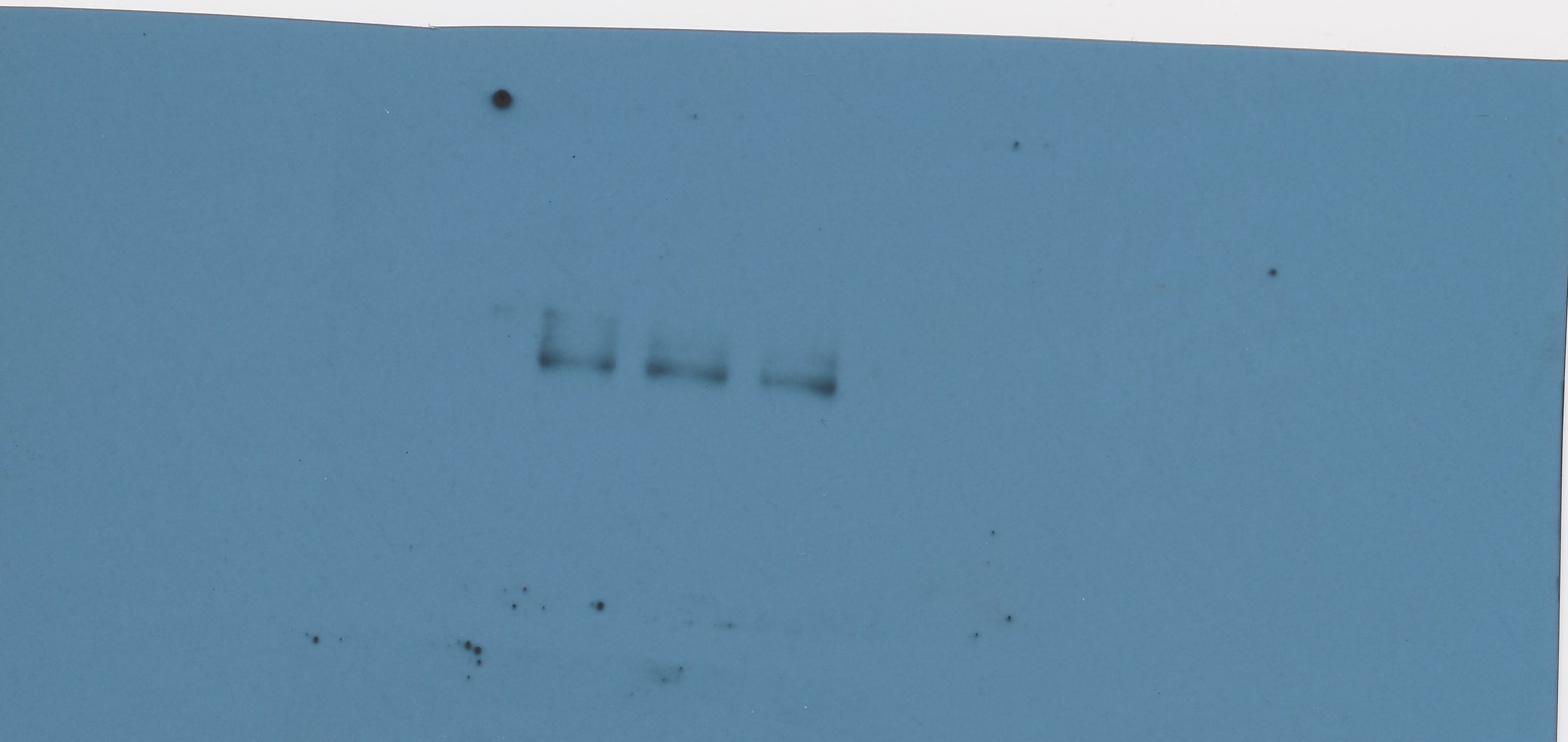

Supplement: Supplementary file 5 — Source Data Fig. 1 [file 44319_2024_64_MOESM5_ESM.zip › 1A/IP p75NTR:IB p75NTR.jpg]

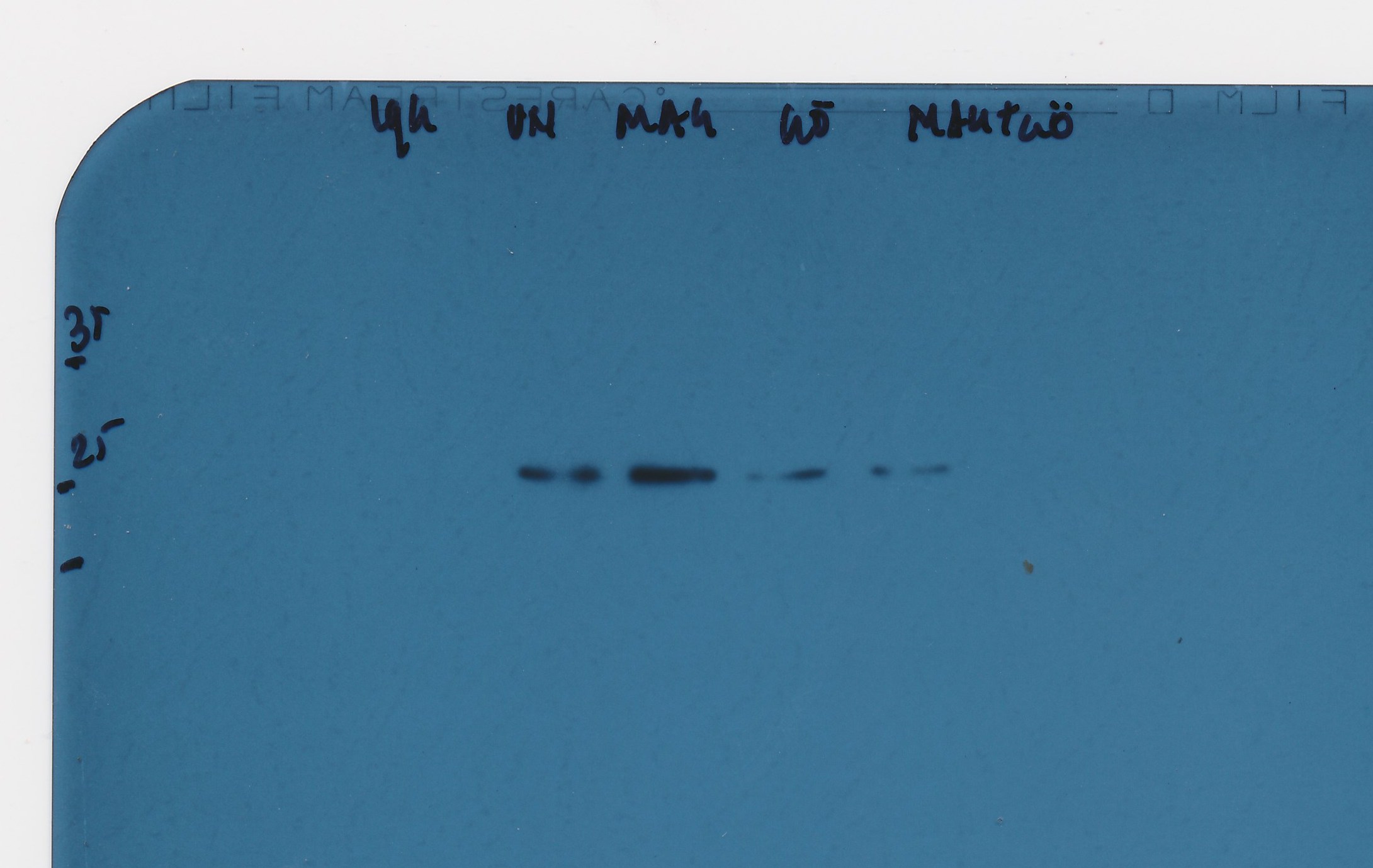

Supplement: Supplementary file 5 — Source Data Fig. 1 [file 44319_2024_64_MOESM5_ESM.zip › 1B/IP P75NTR:IB RhoGDI.jpg]

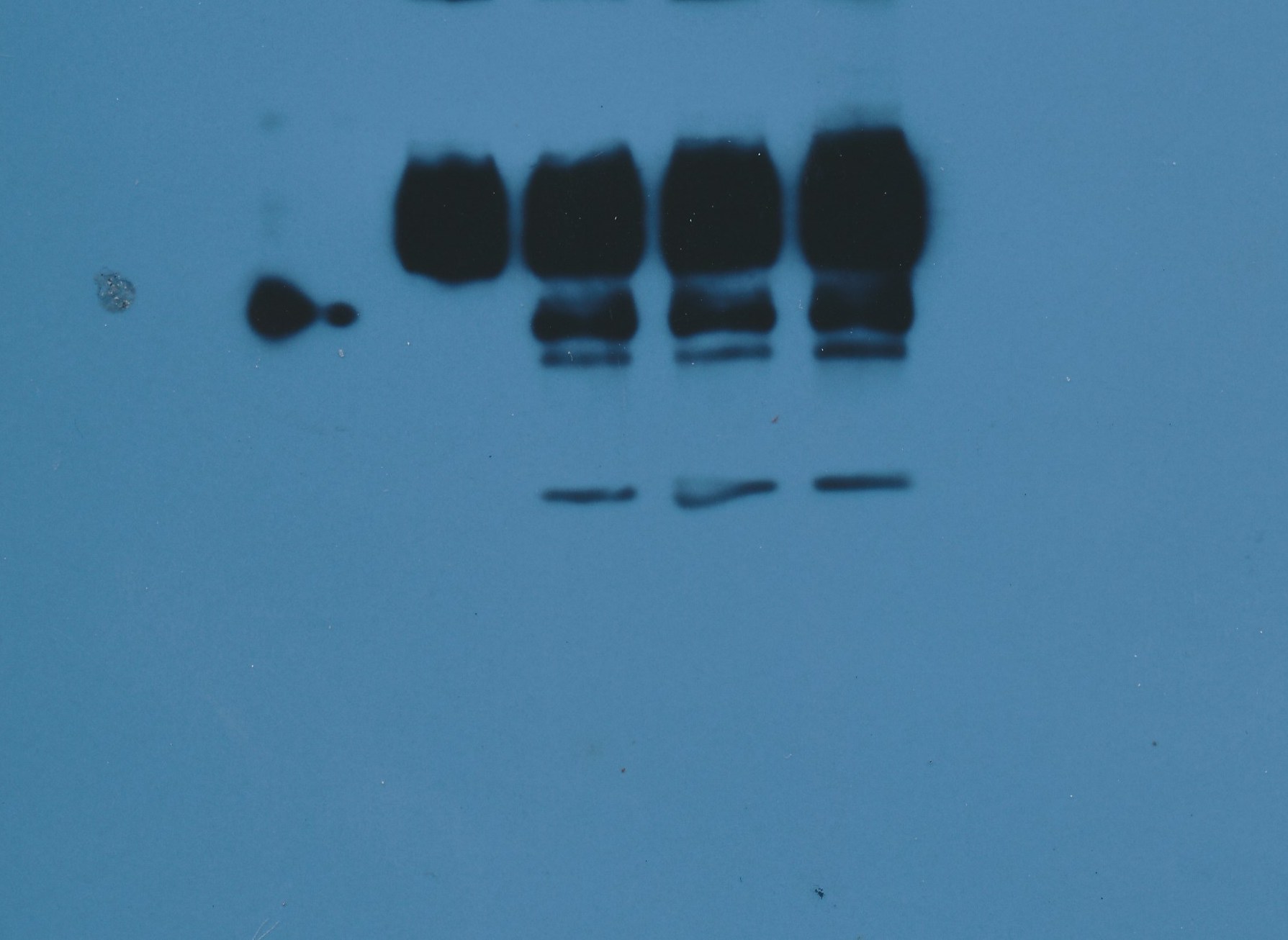

Supplement: Supplementary file 5 — Source Data Fig. 1 [file 44319_2024_64_MOESM5_ESM.zip › 1B/IP P75NTR:IB P75NTR.jpeg]

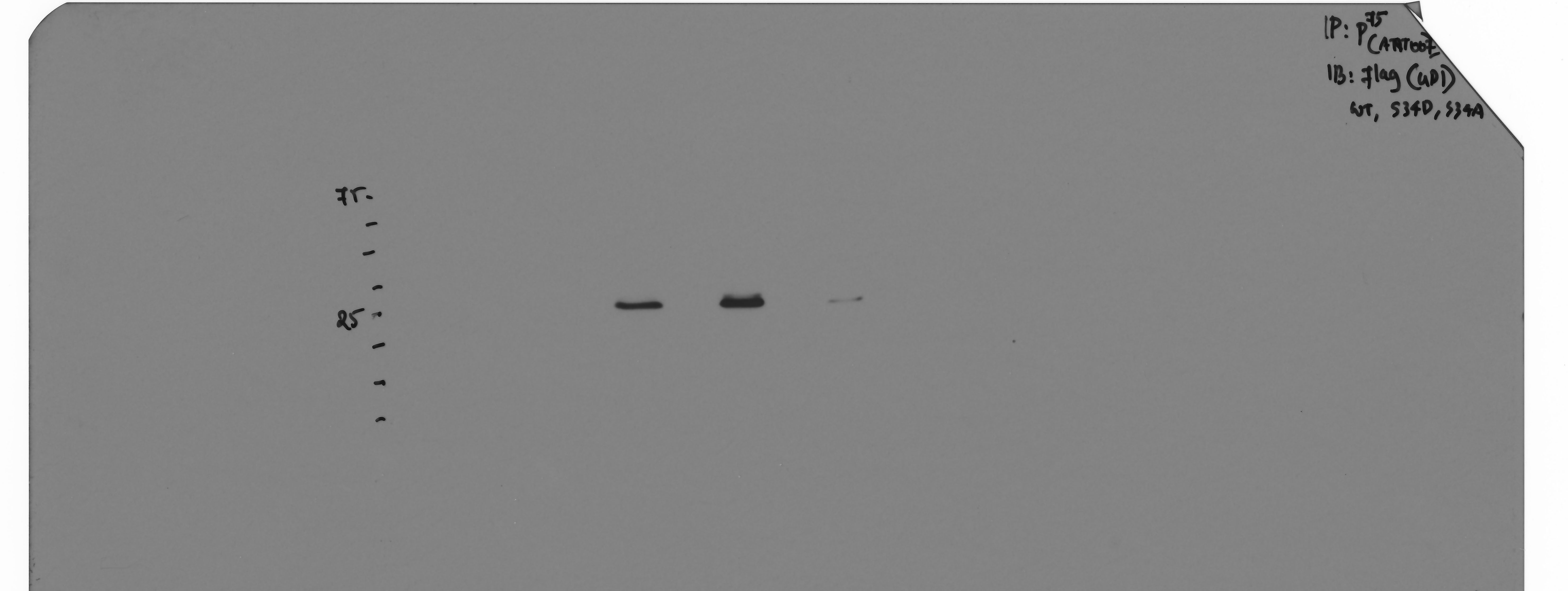

Supplement: Supplementary file 5 — Source Data Fig. 1 [file 44319_2024_64_MOESM5_ESM.zip › 1D/IP p75NTR:IB Flag (RhoGDI).jpg]

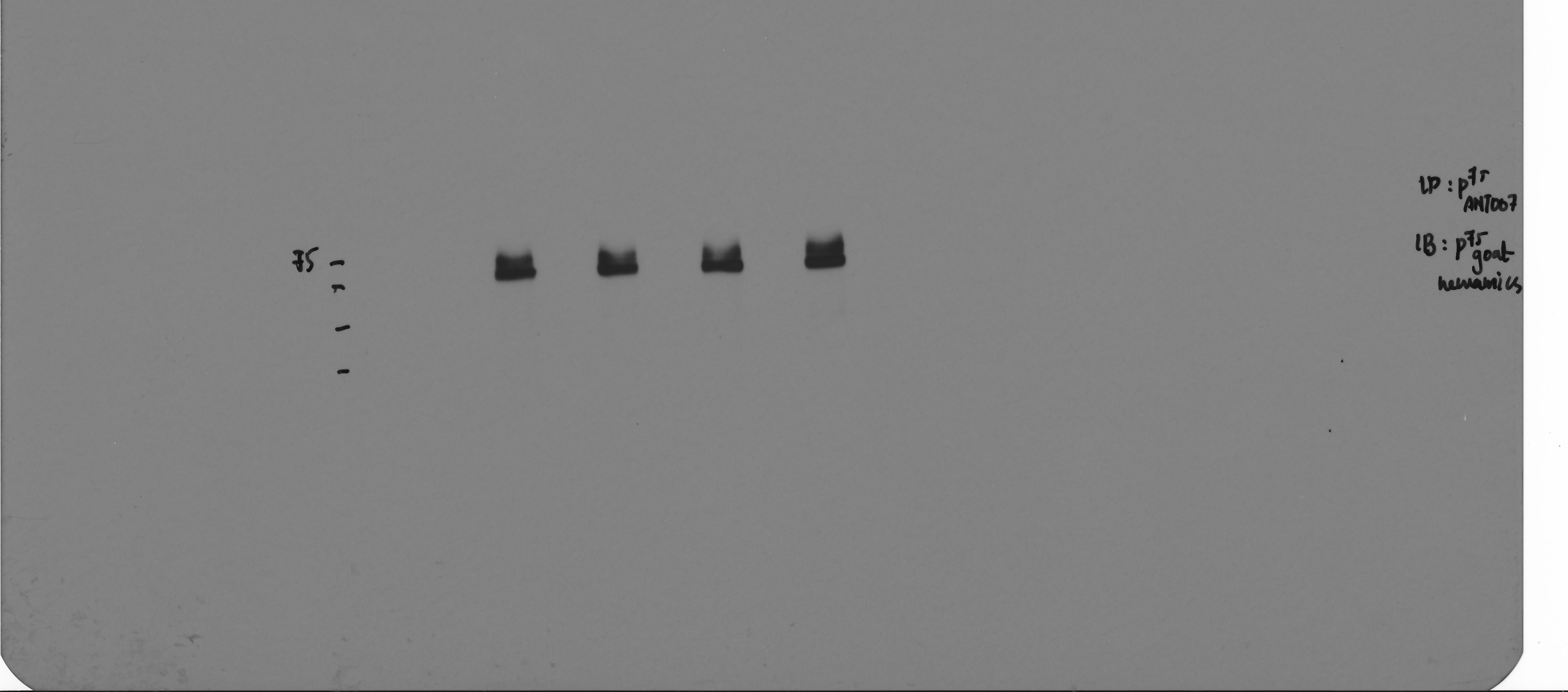

Supplement: Supplementary file 5 — Source Data Fig. 1 [file 44319_2024_64_MOESM5_ESM.zip › 1D/IP p75NTR:IB p75.jpg]

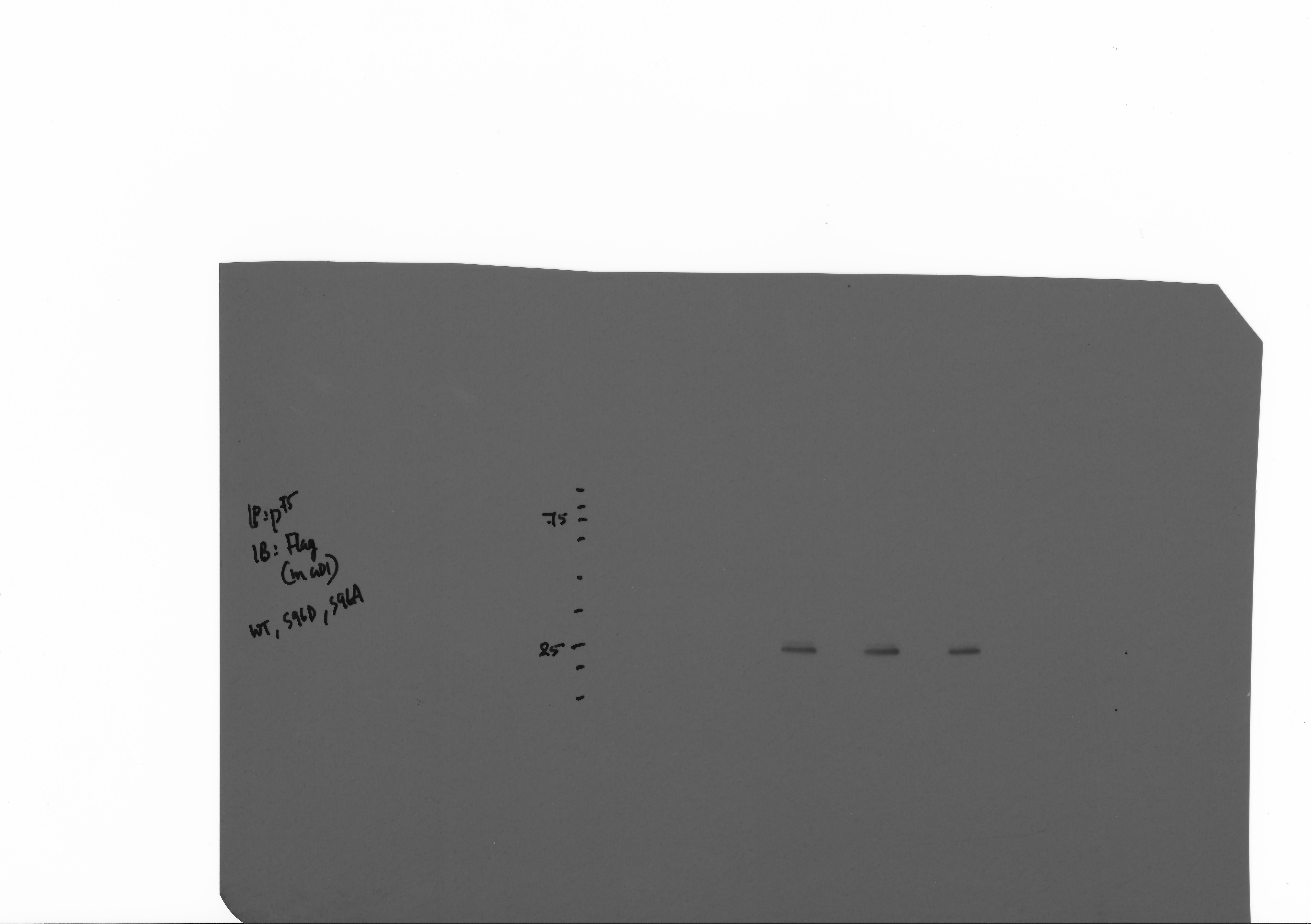

Supplement: Supplementary file 5 — Source Data Fig. 1 [file 44319_2024_64_MOESM5_ESM.zip › 1F/IP p75NTR:IB Flag (RhoGDI).jpeg]

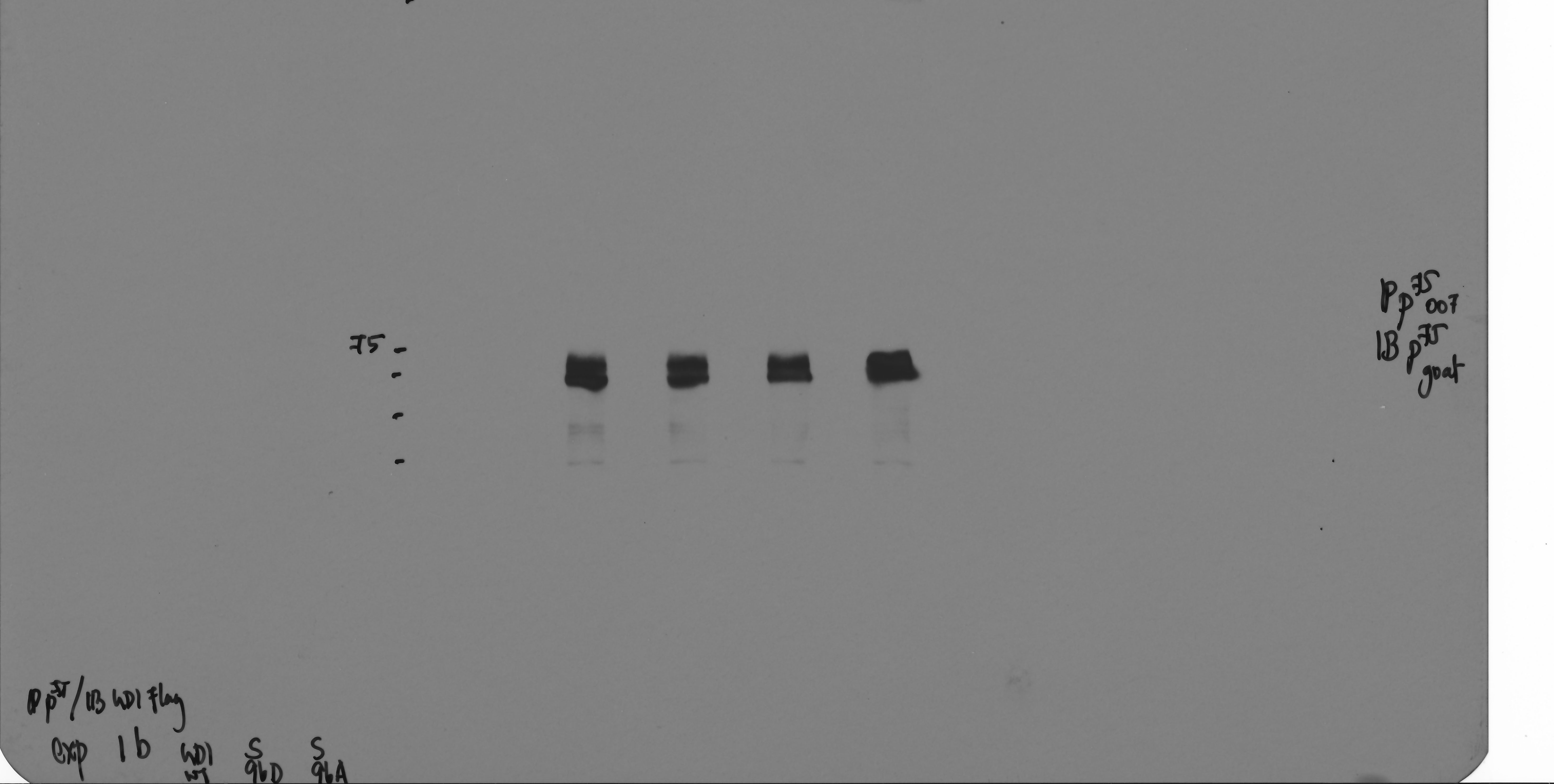

Supplement: Supplementary file 5 — Source Data Fig. 1 [file 44319_2024_64_MOESM5_ESM.zip › 1F/IP p75NTR:IB p75NTR.jpg]

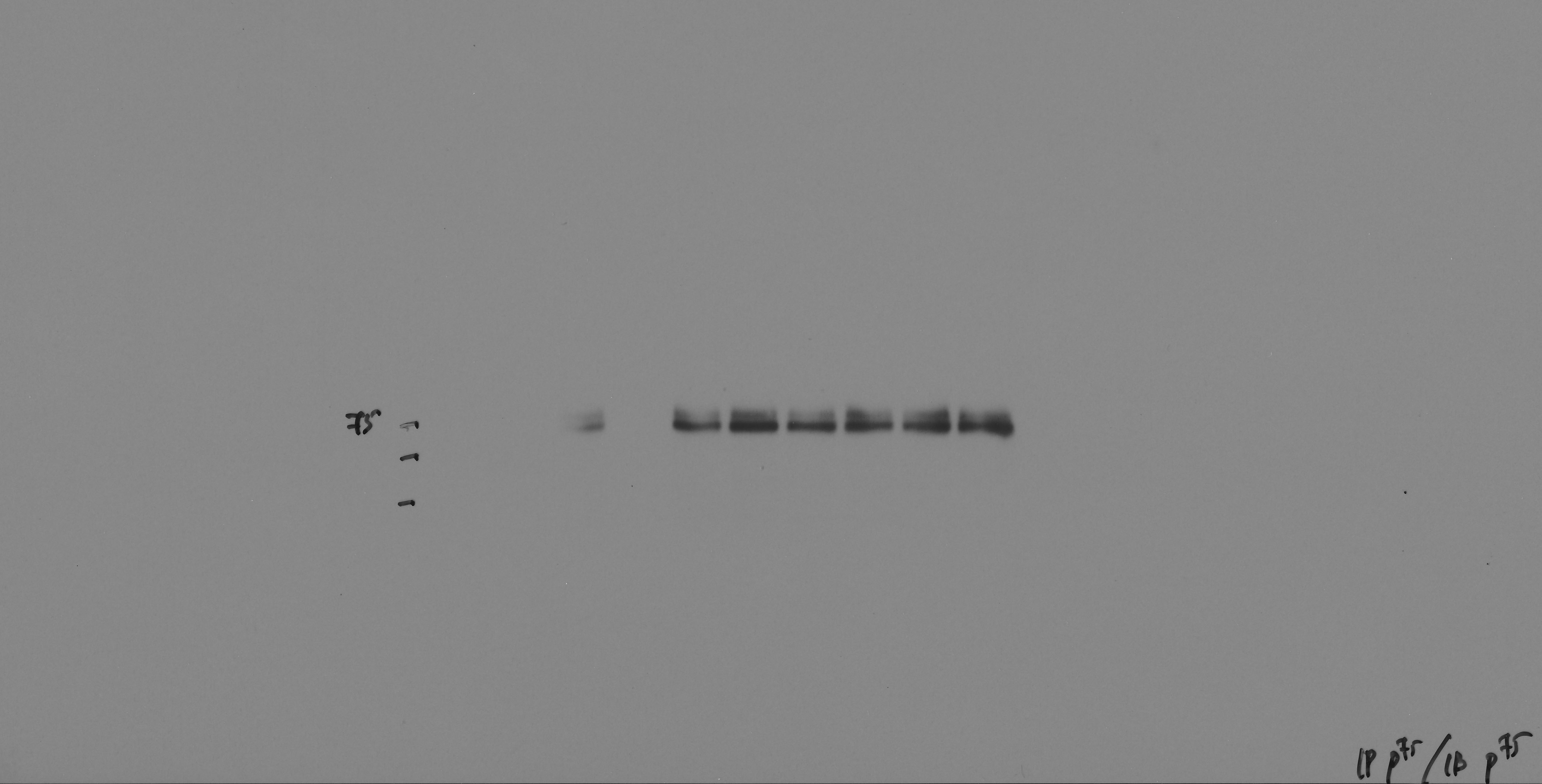

Supplement: Supplementary file 5 — Source Data Fig. 1 [file 44319_2024_64_MOESM5_ESM.zip › 1G/IP p75NTR:IB p75NTR.jpg]

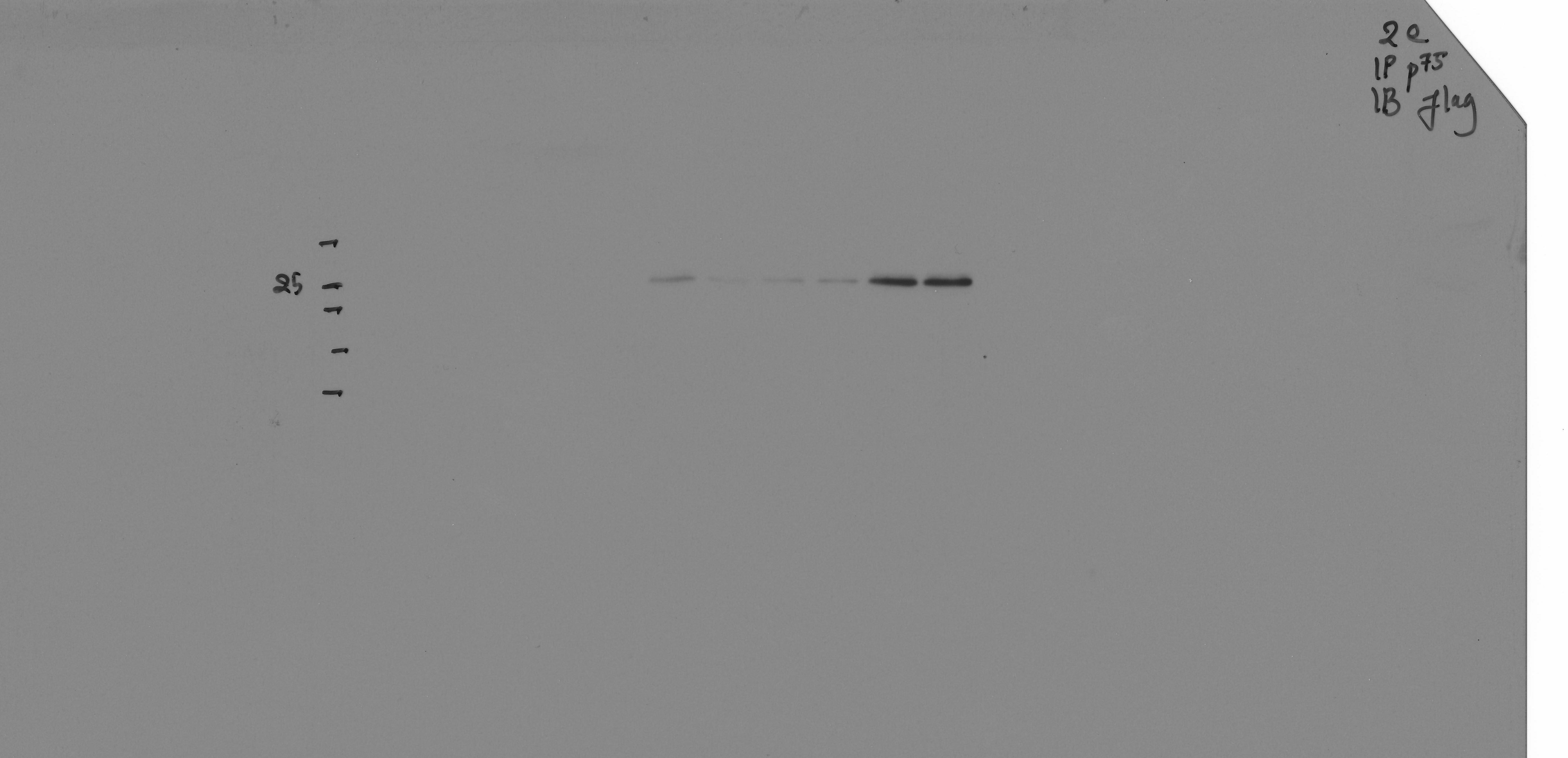

Supplement: Supplementary file 5 — Source Data Fig. 1 [file 44319_2024_64_MOESM5_ESM.zip › 1G/IP p75NTR:IB flag (RhoGDI).jpg]

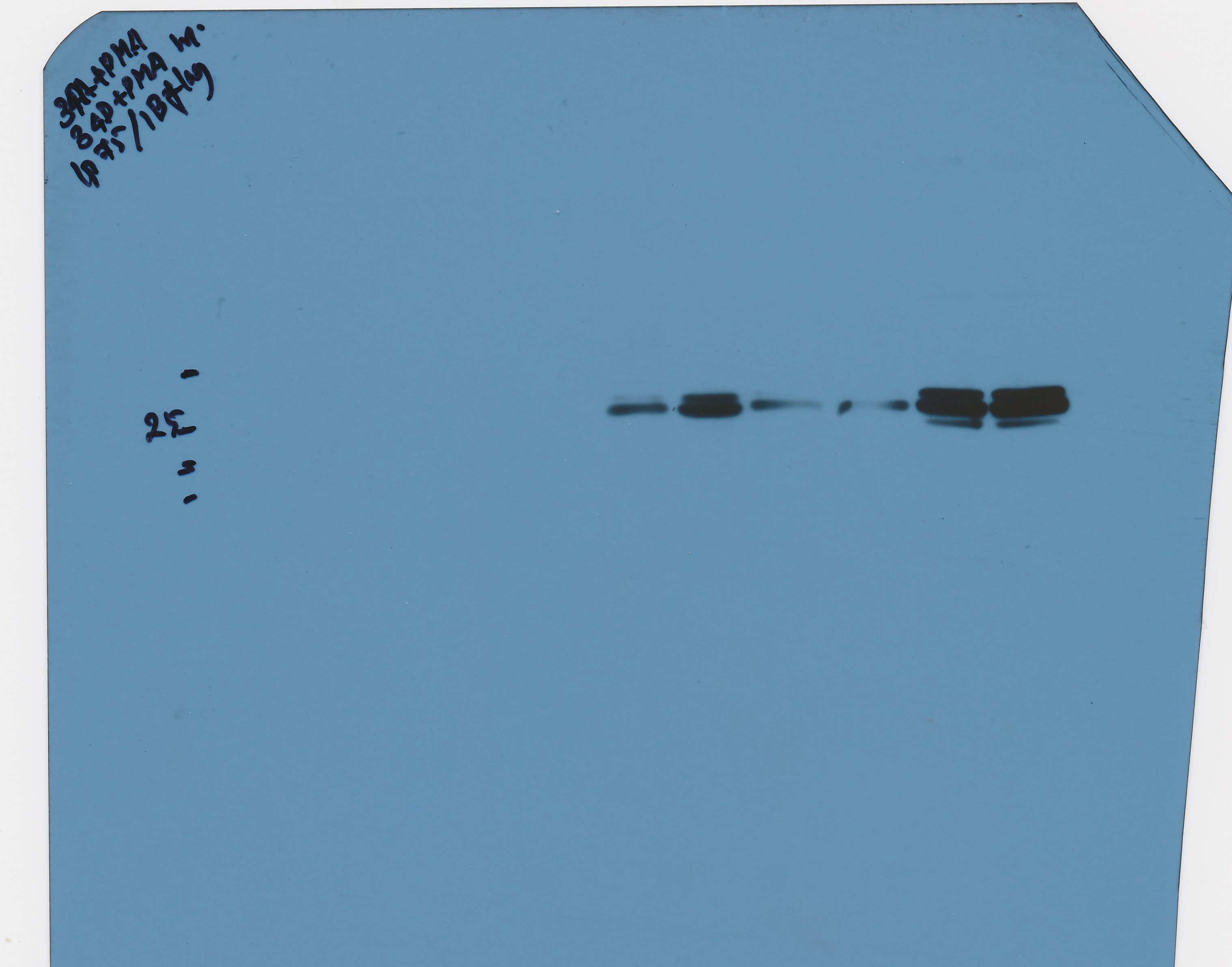

Supplement: Supplementary file 5 — Source Data Fig. 1 [file 44319_2024_64_MOESM5_ESM.zip › 1H/IP p75NTR:IB Flag (RhoGDI).jpg]

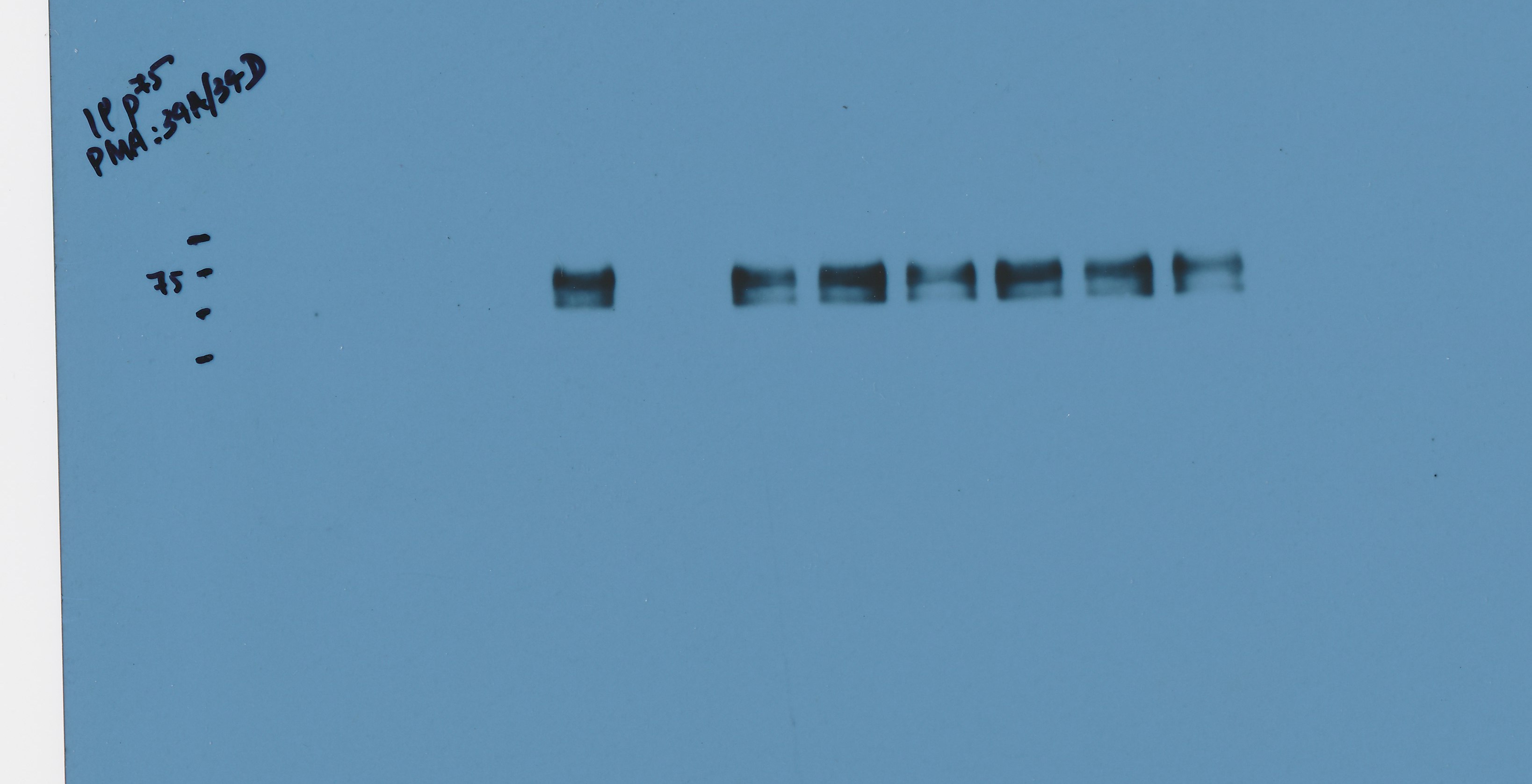

Supplement: Supplementary file 5 — Source Data Fig. 1 [file 44319_2024_64_MOESM5_ESM.zip › 1H/ IP p75NTR:IB p75NTR.jpg]

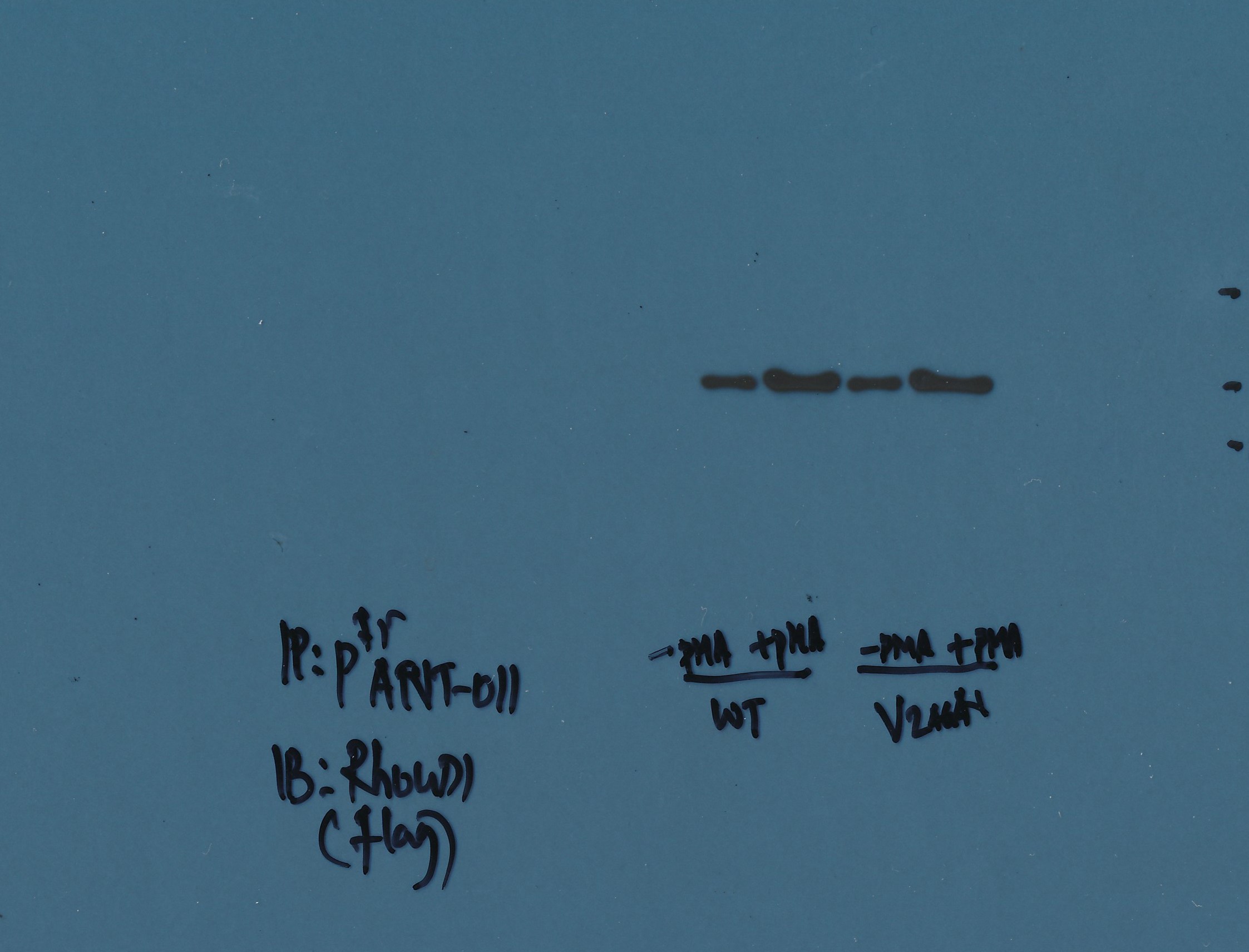

Supplement: Supplementary file 5 — Source Data Fig. 1 [file 44319_2024_64_MOESM5_ESM.zip › 1I/IP p75NTR (ICD):IB Flag (RhoGDI).jpg]

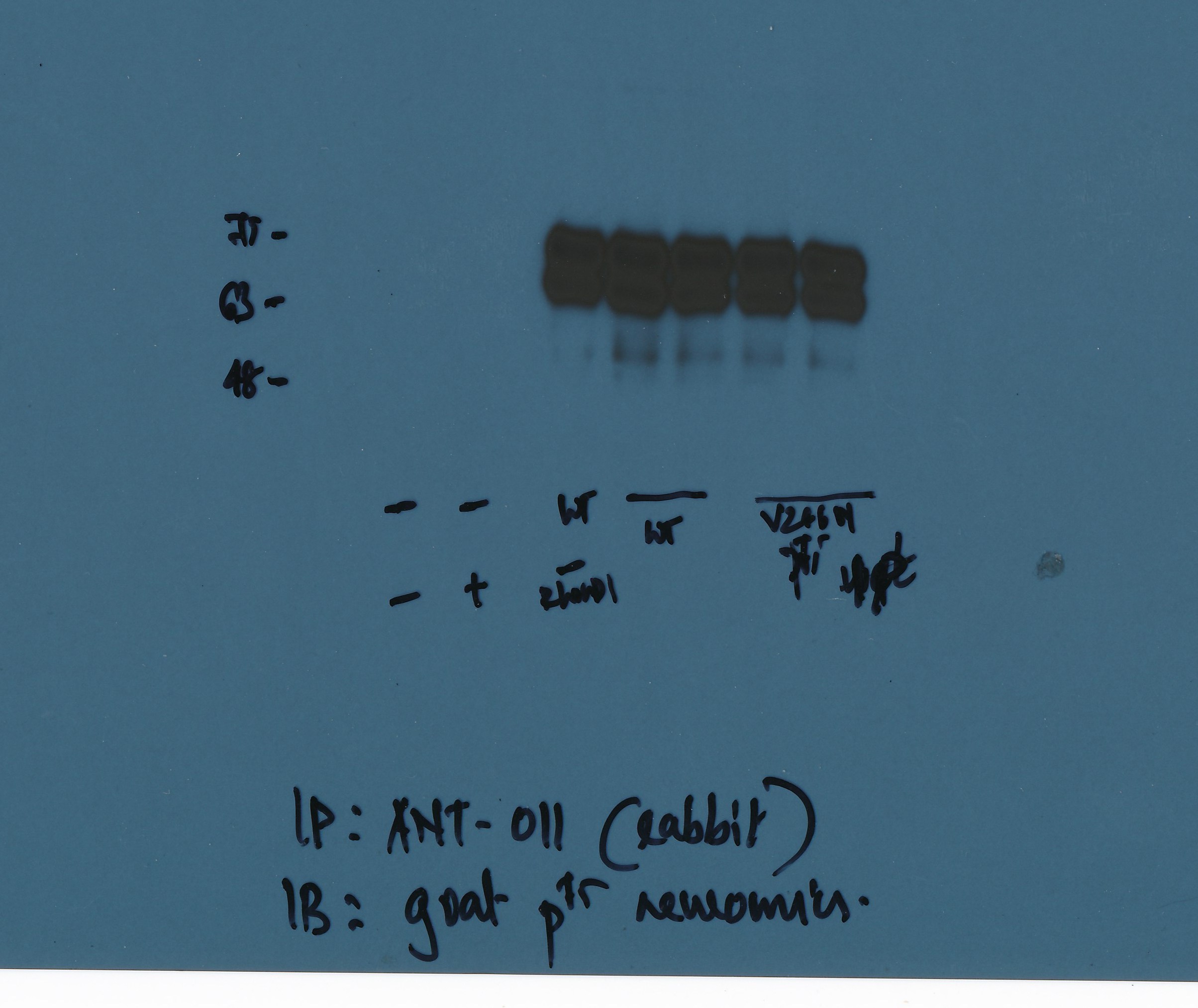

Supplement: Supplementary file 5 — Source Data Fig. 1 [file 44319_2024_64_MOESM5_ESM.zip › 1I/IP p75NTR (ICD):IB p75NTR.jpg]

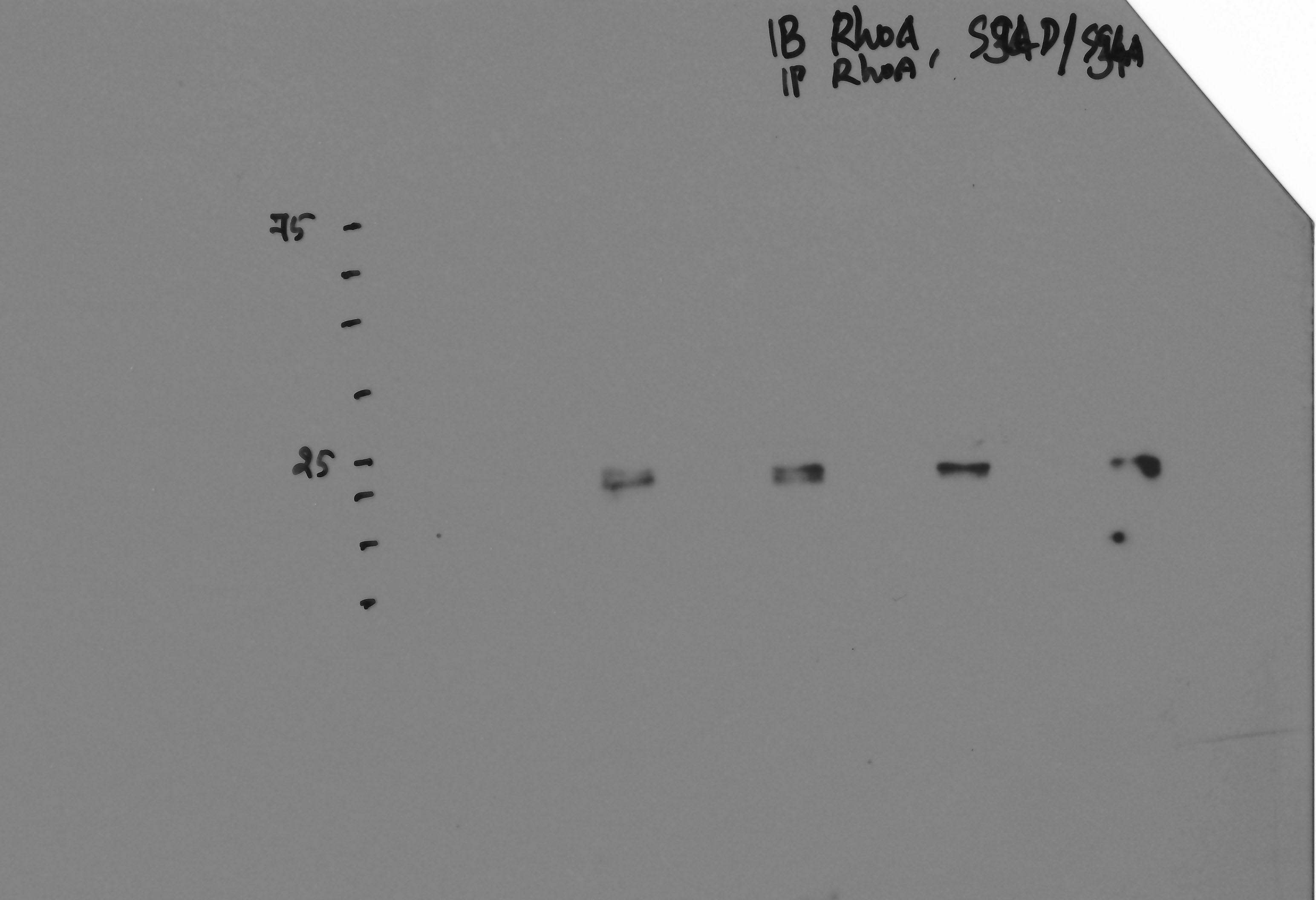

Supplement: Supplementary file 6 — Source Data Fig. 2 [file 44319_2024_64_MOESM6_ESM.zip › 2A/IP RhoA:IB HA (RhoA).jpg]

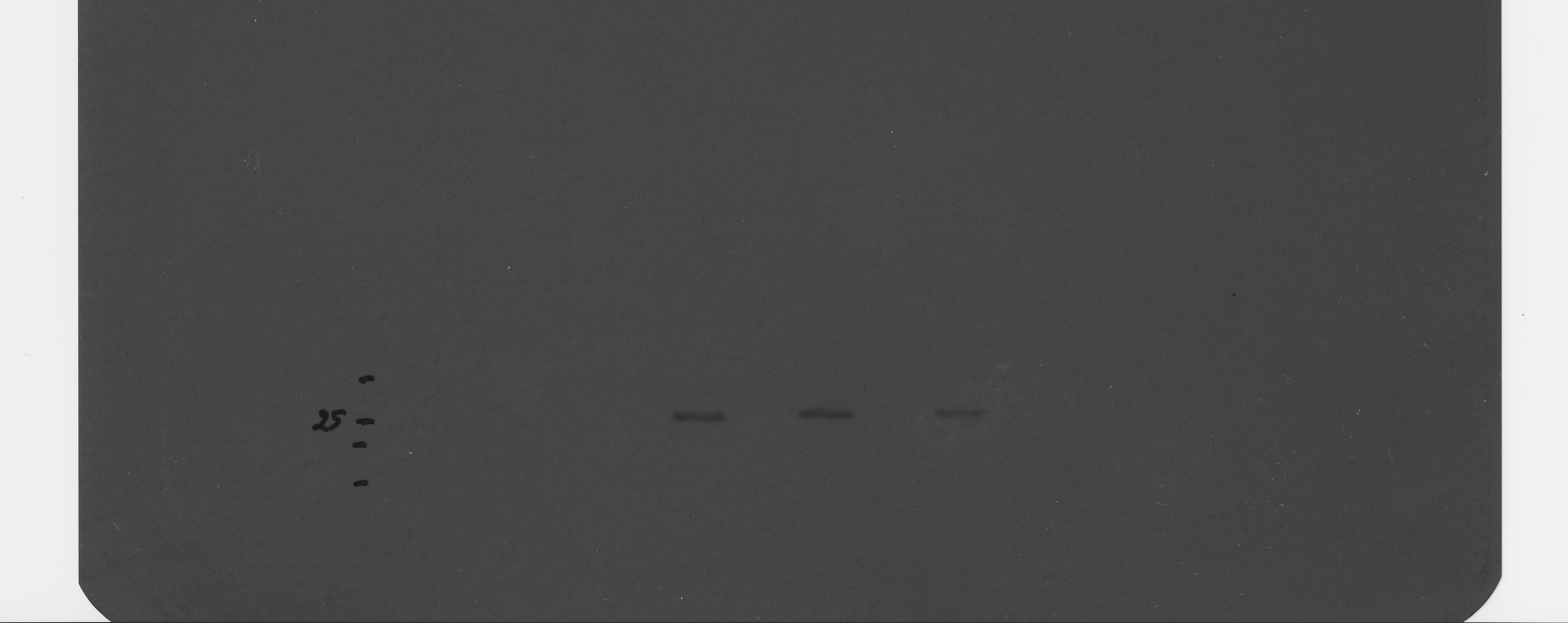

Supplement: Supplementary file 6 — Source Data Fig. 2 [file 44319_2024_64_MOESM6_ESM.zip › 2A/IP RhoA:IB Flag (RhoGDI).jpeg]

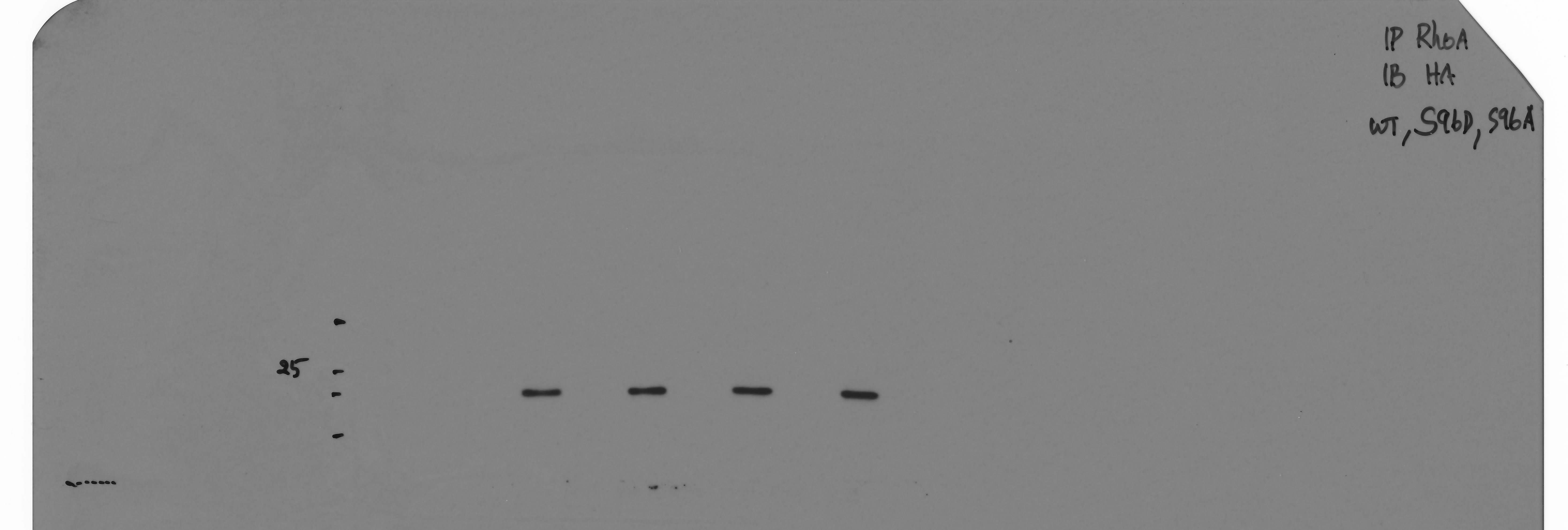

Supplement: Supplementary file 6 — Source Data Fig. 2 [file 44319_2024_64_MOESM6_ESM.zip › 2B/IP RhoA:IB HA (RhoA).jpg]

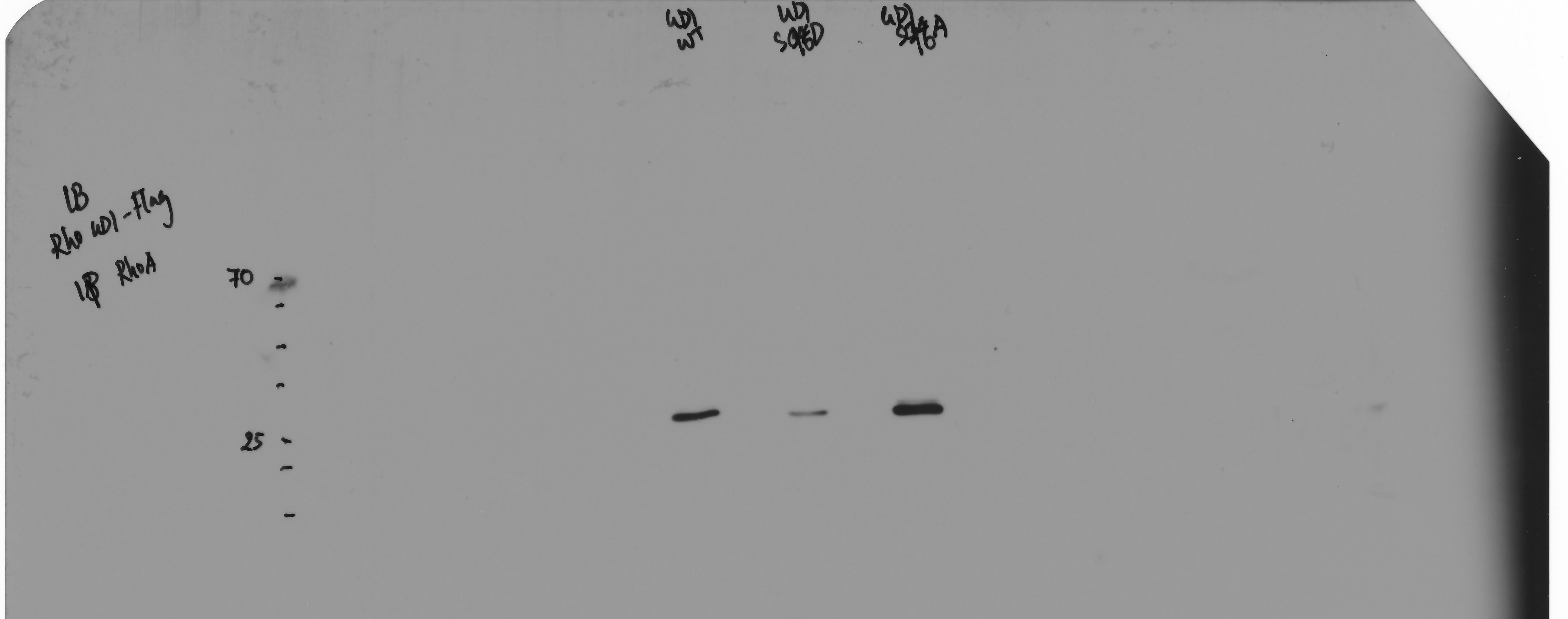

Supplement: Supplementary file 6 — Source Data Fig. 2 [file 44319_2024_64_MOESM6_ESM.zip › 2B/IP RhoA:IB Flag (RhoGDI).jpg]

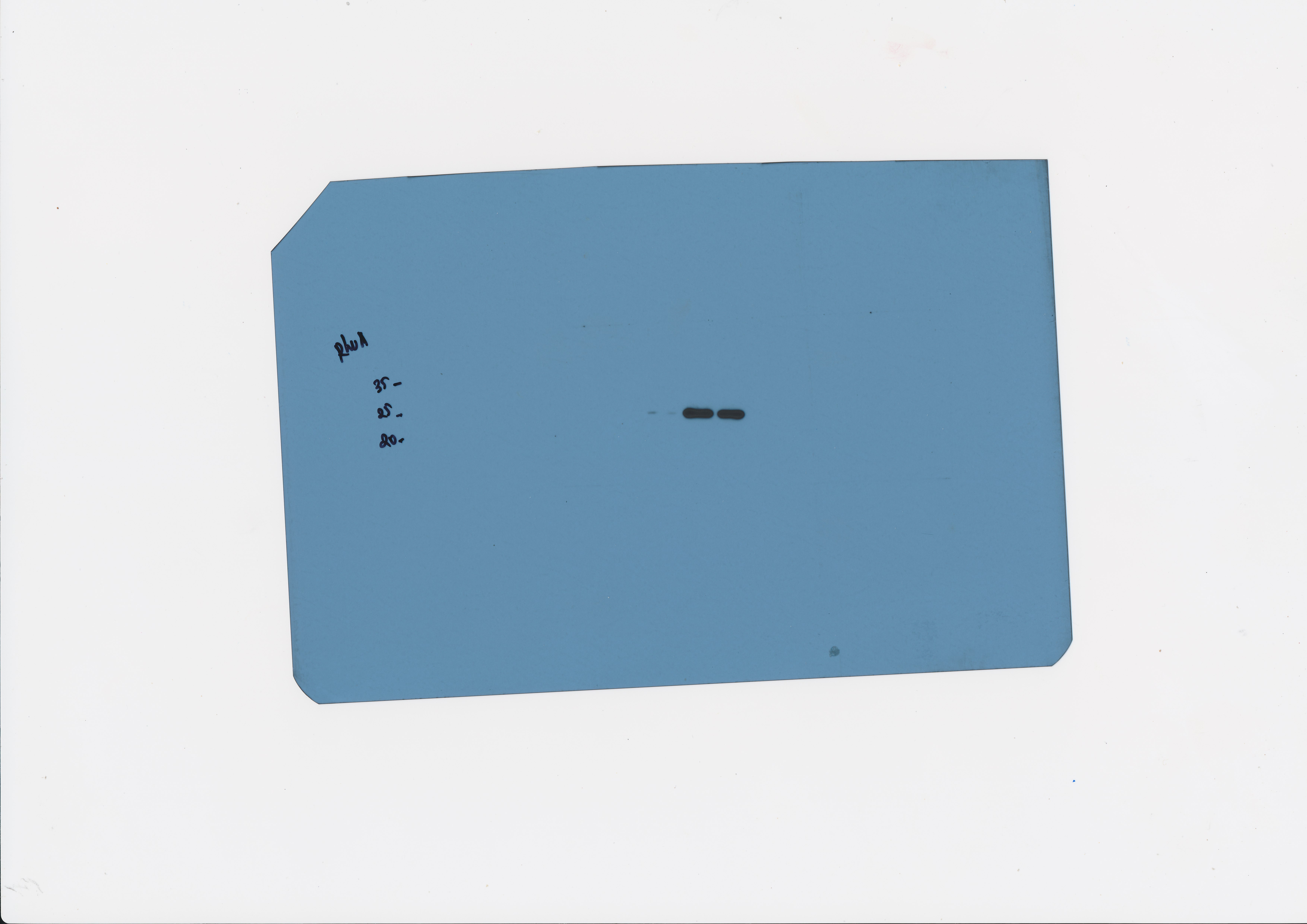

Supplement: Supplementary file 6 — Source Data Fig. 2 [file 44319_2024_64_MOESM6_ESM.zip › 2D/IP Flag:IB HA (RhoA).jpg]

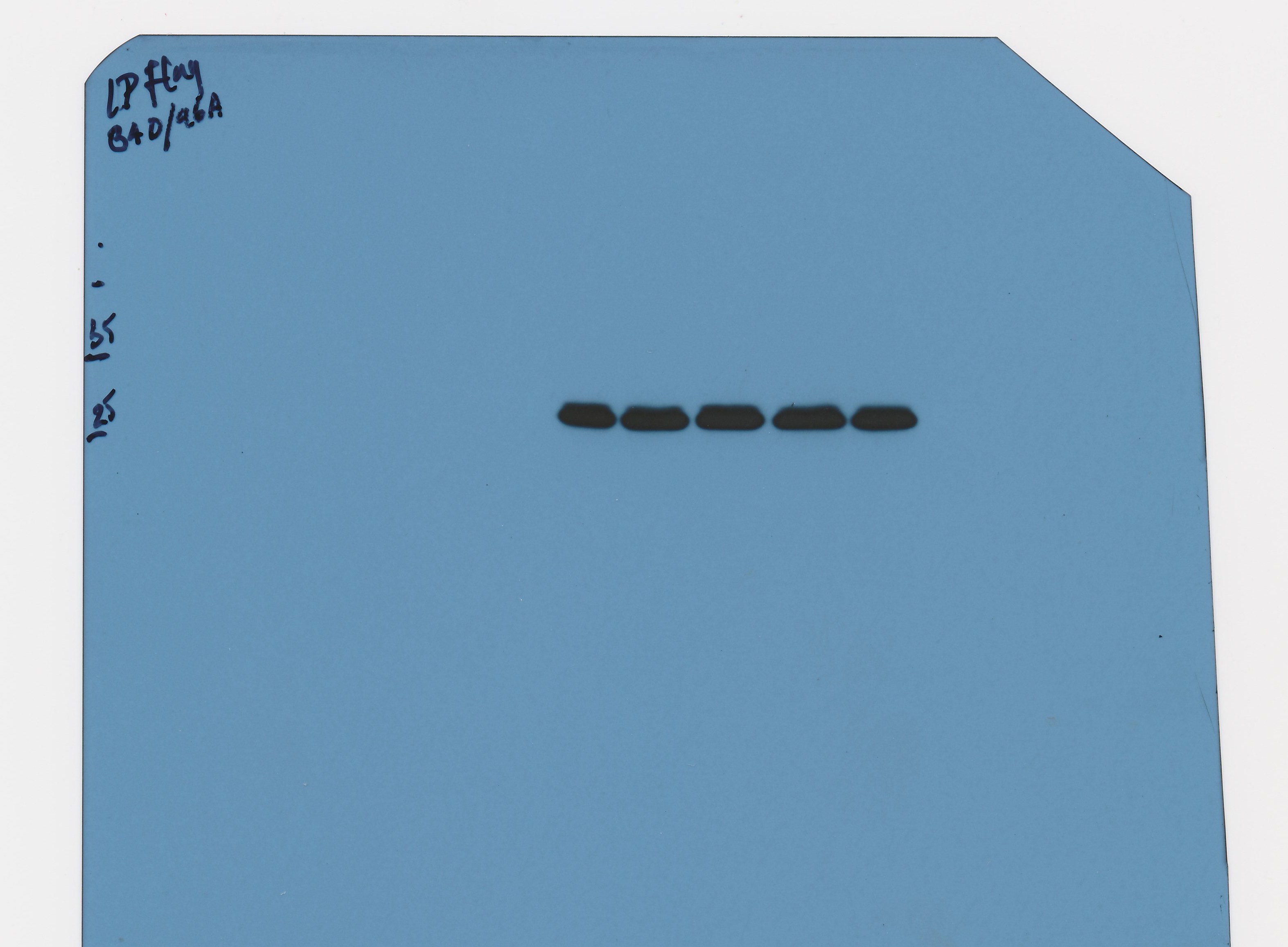

Supplement: Supplementary file 6 — Source Data Fig. 2 [file 44319_2024_64_MOESM6_ESM.zip › 2D/IP Flag:IB RhoGDI.jpg]

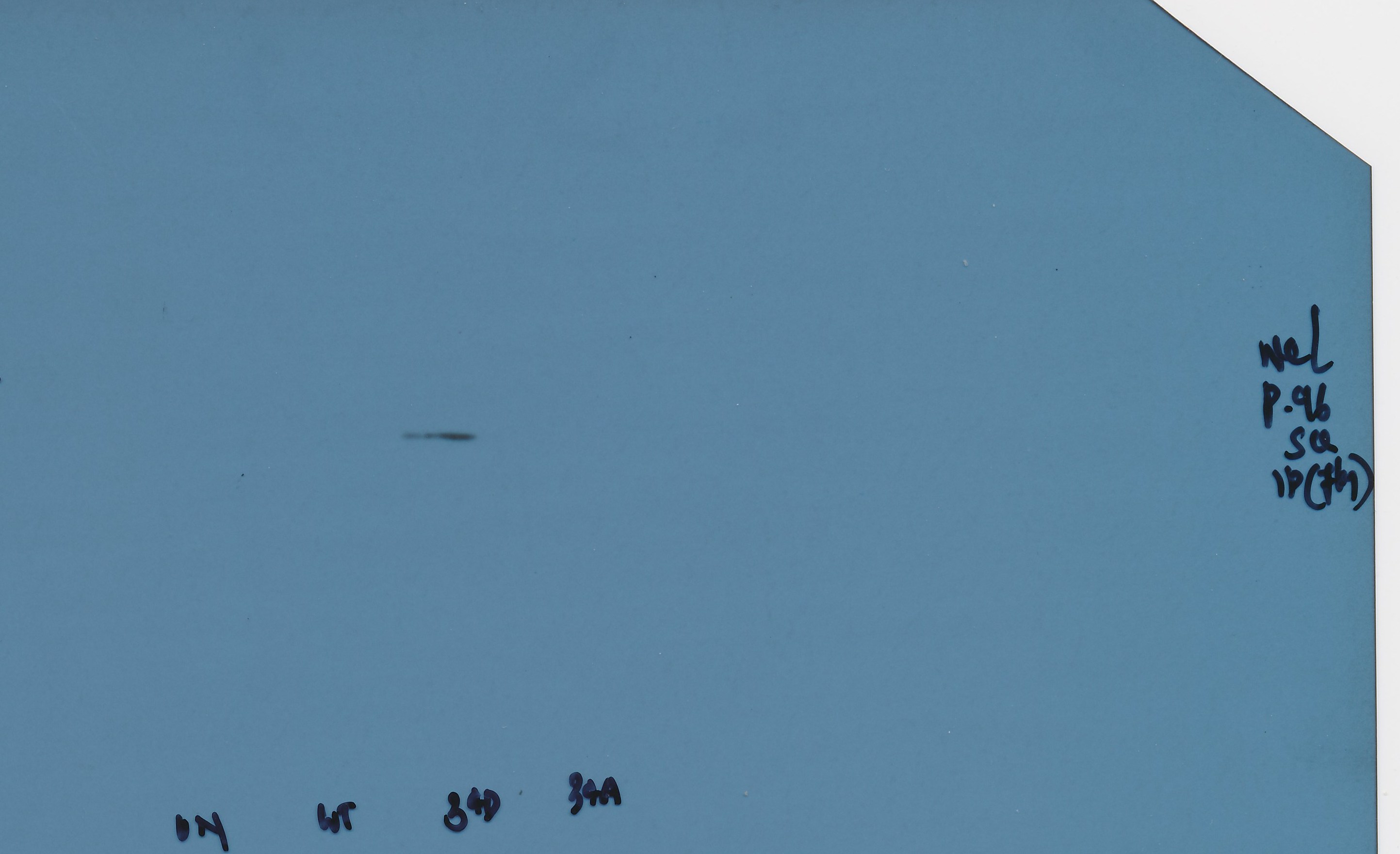

Supplement: Supplementary file 6 — Source Data Fig. 2 [file 44319_2024_64_MOESM6_ESM.zip › 2E/IP Flag (RhoGDI):IB P-Ser96 RhoGDI.jpeg]

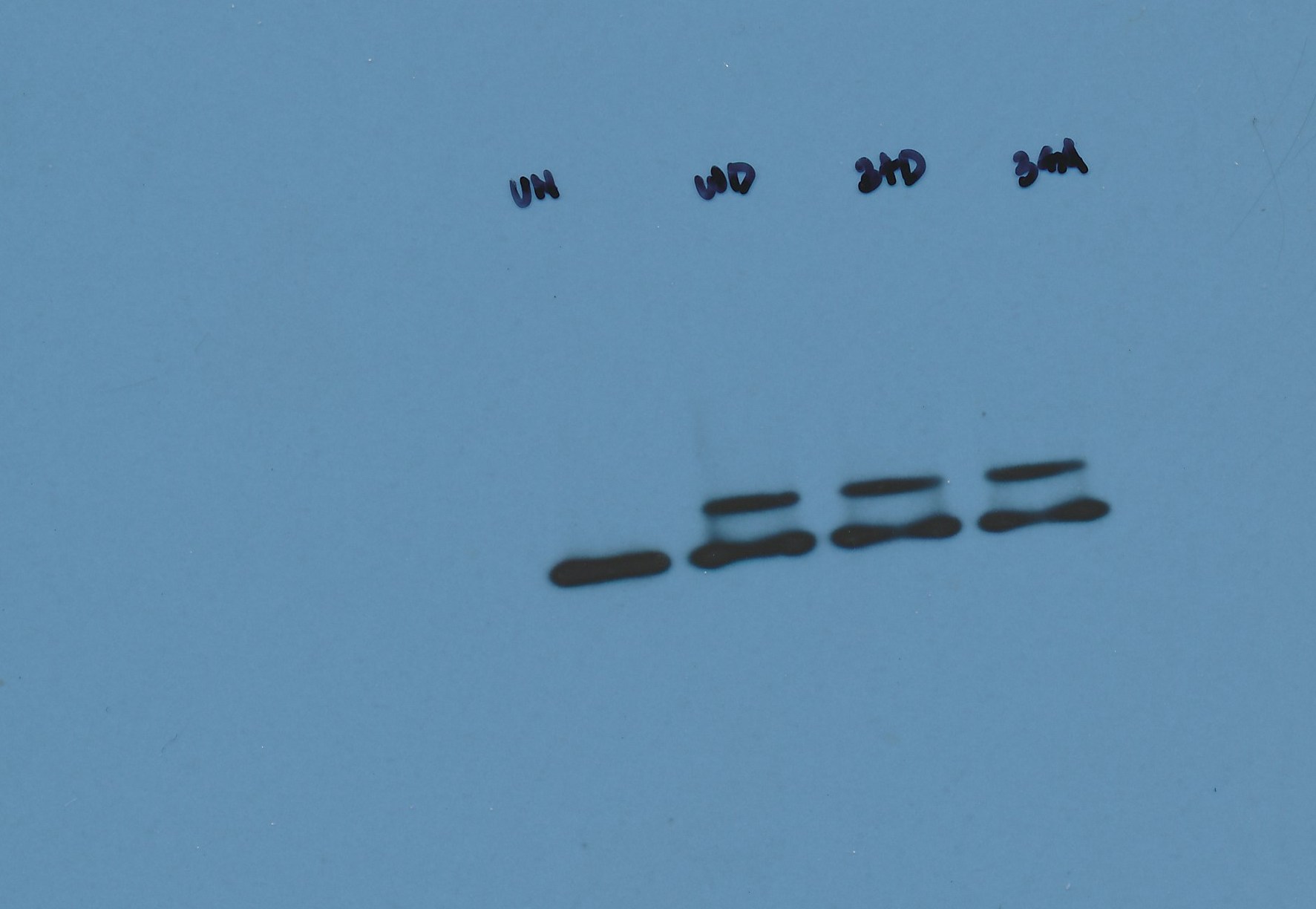

Supplement: Supplementary file 6 — Source Data Fig. 2 [file 44319_2024_64_MOESM6_ESM.zip › 2E/IP Flag:IB RhoGDI.jpeg]

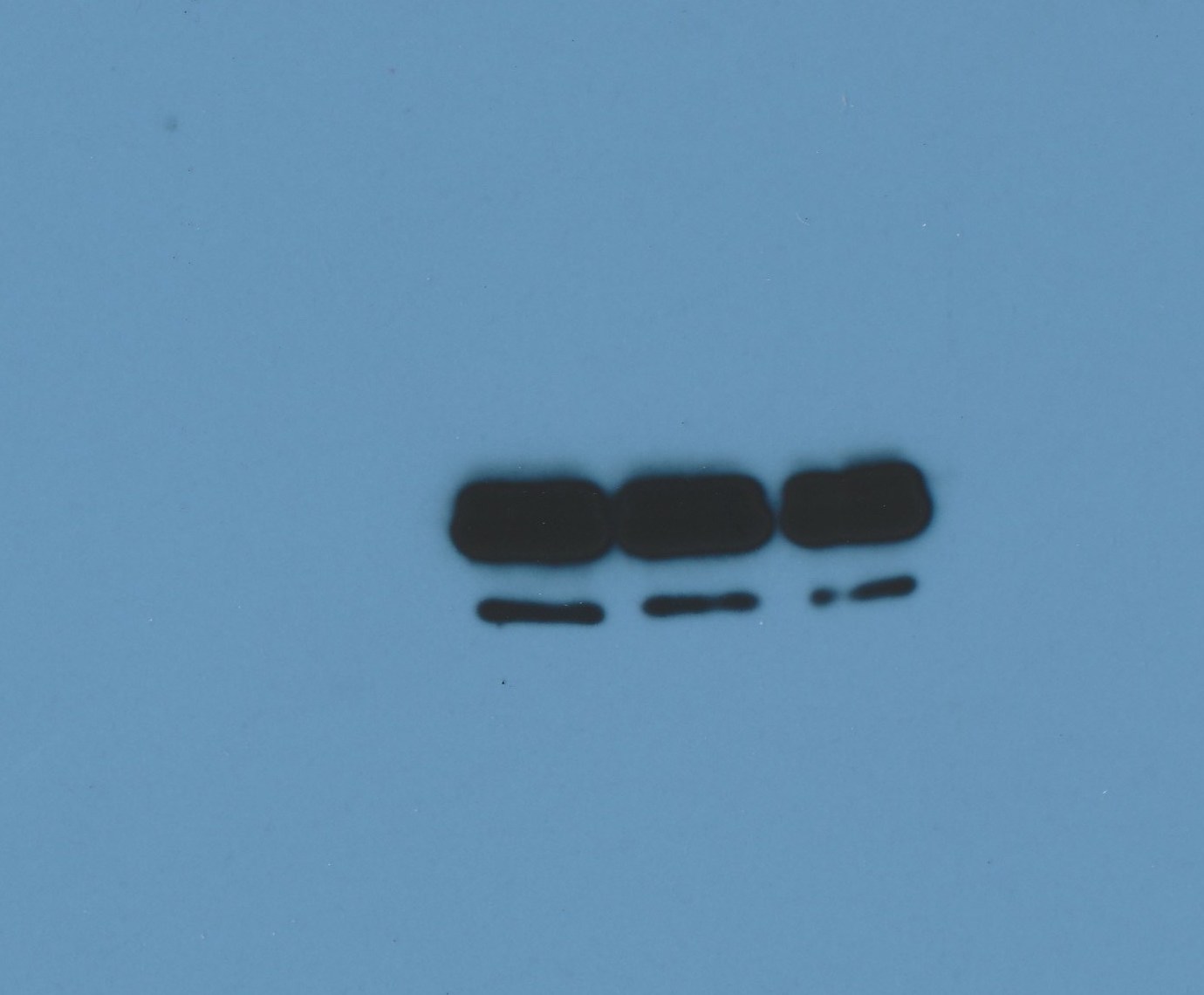

Supplement: Supplementary file 6 — Source Data Fig. 2 [file 44319_2024_64_MOESM6_ESM.zip › 2F/IP RhoGDI:IB RhoGDI.jpeg]

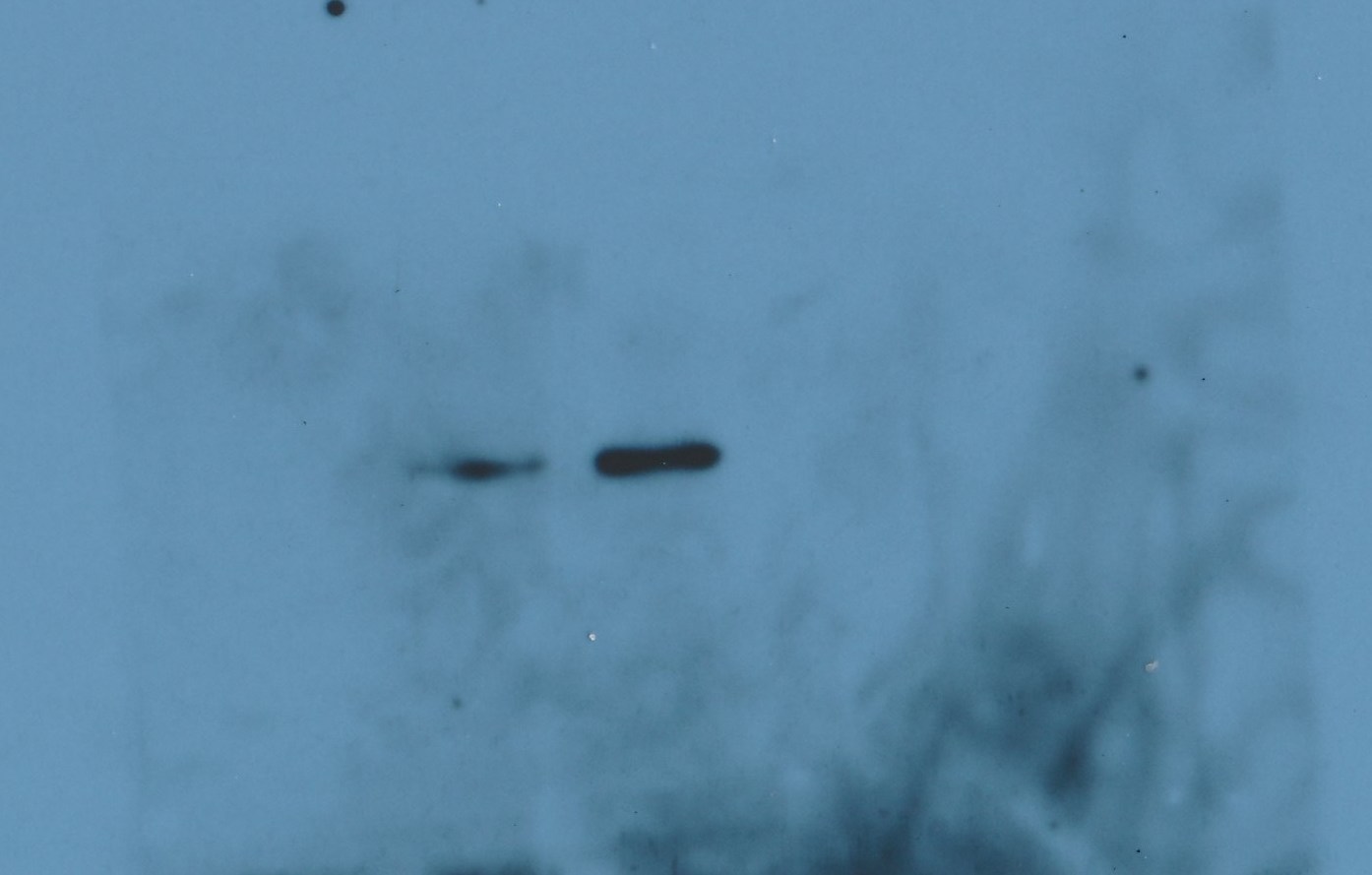

Supplement: Supplementary file 6 — Source Data Fig. 2 [file 44319_2024_64_MOESM6_ESM.zip › 2F/IP RhoGDI:IB P-Ser96 RhoGDI.jpeg]

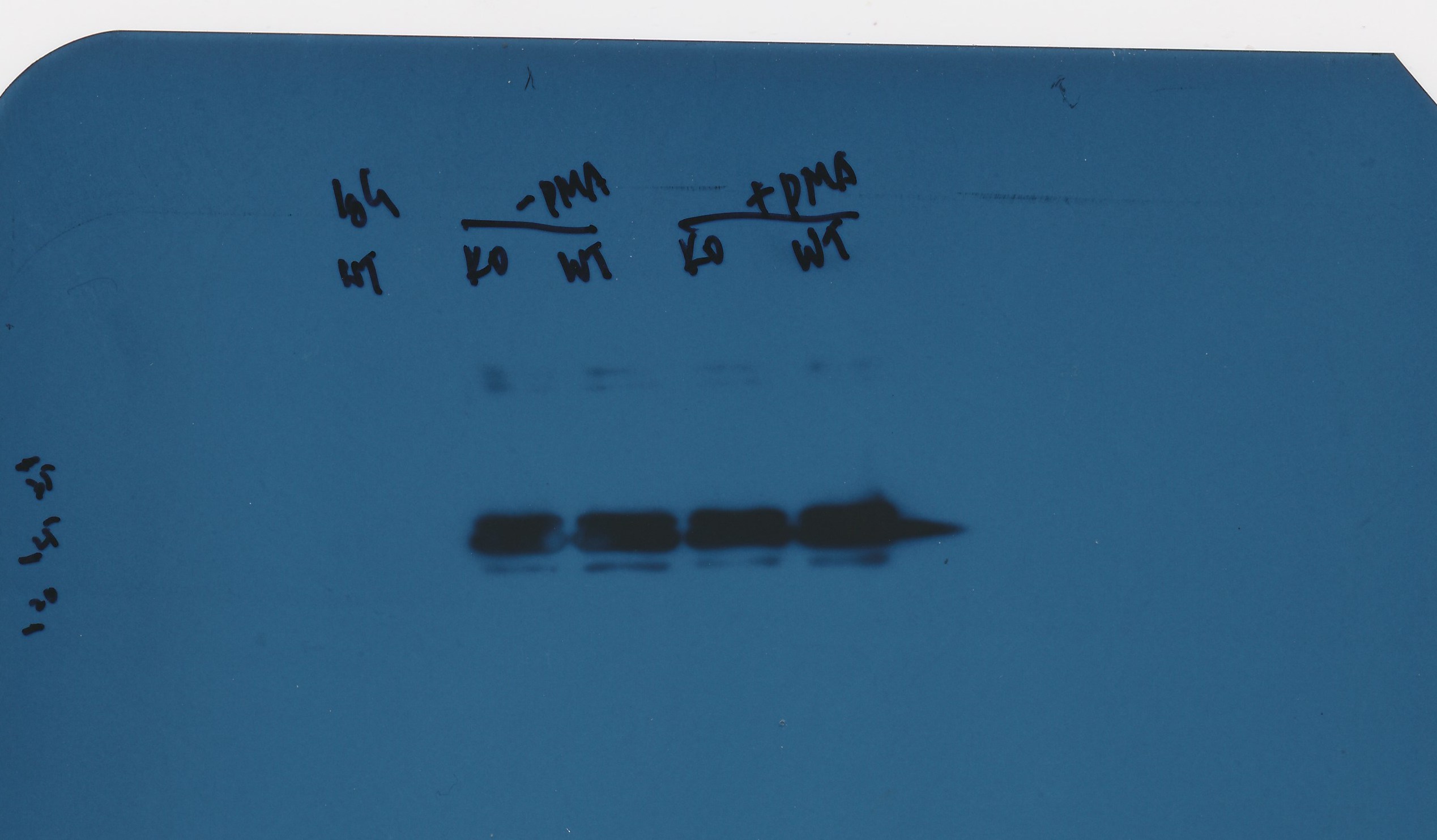

Supplement: Supplementary file 6 — Source Data Fig. 2 [file 44319_2024_64_MOESM6_ESM.zip › 2G/IP RhoGDI:IB RhoGDI.jpeg]

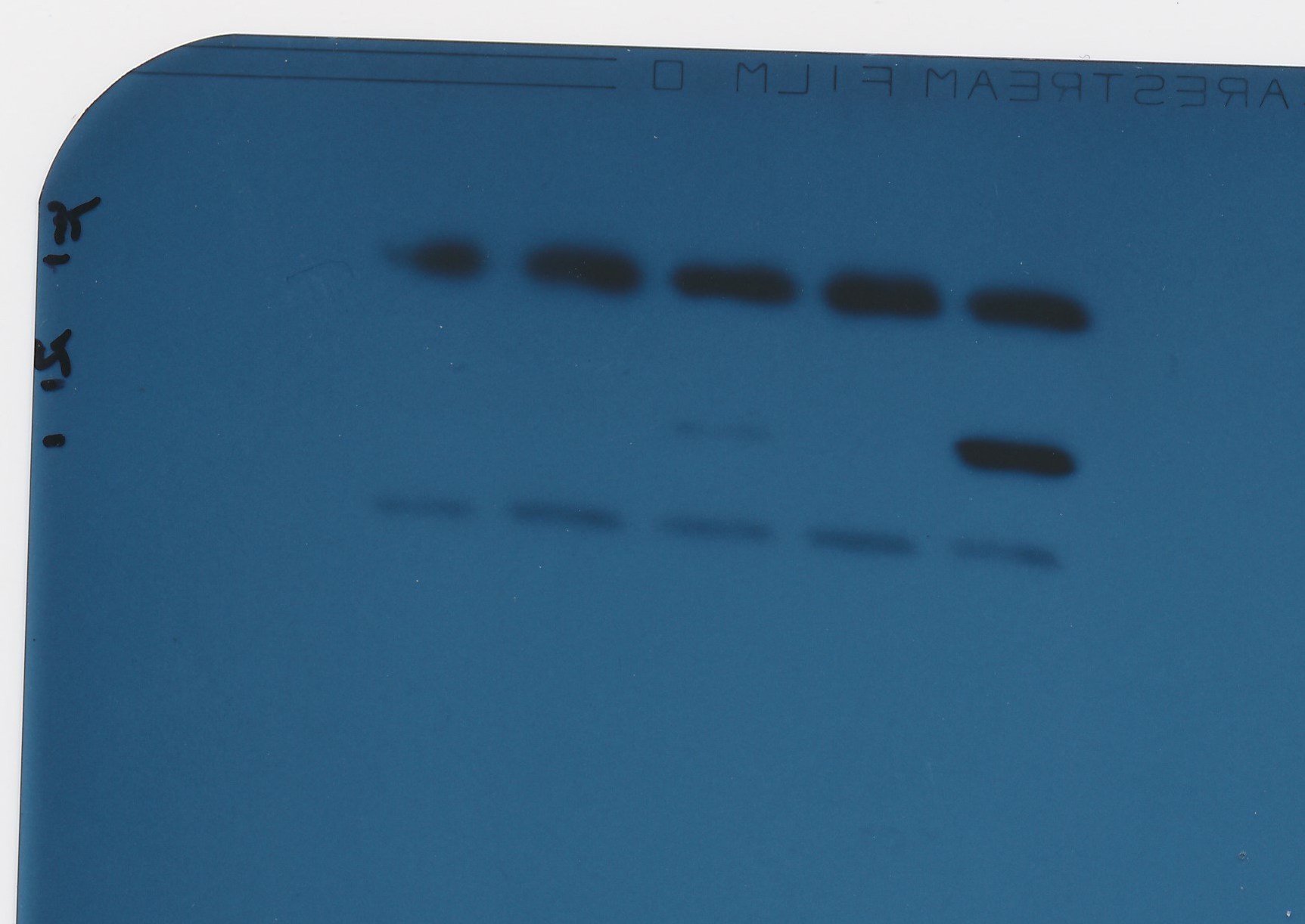

Supplement: Supplementary file 6 — Source Data Fig. 2 [file 44319_2024_64_MOESM6_ESM.zip › 2G/IP RhoGDI:IB P-Ser96 RhoGDI.jpg]

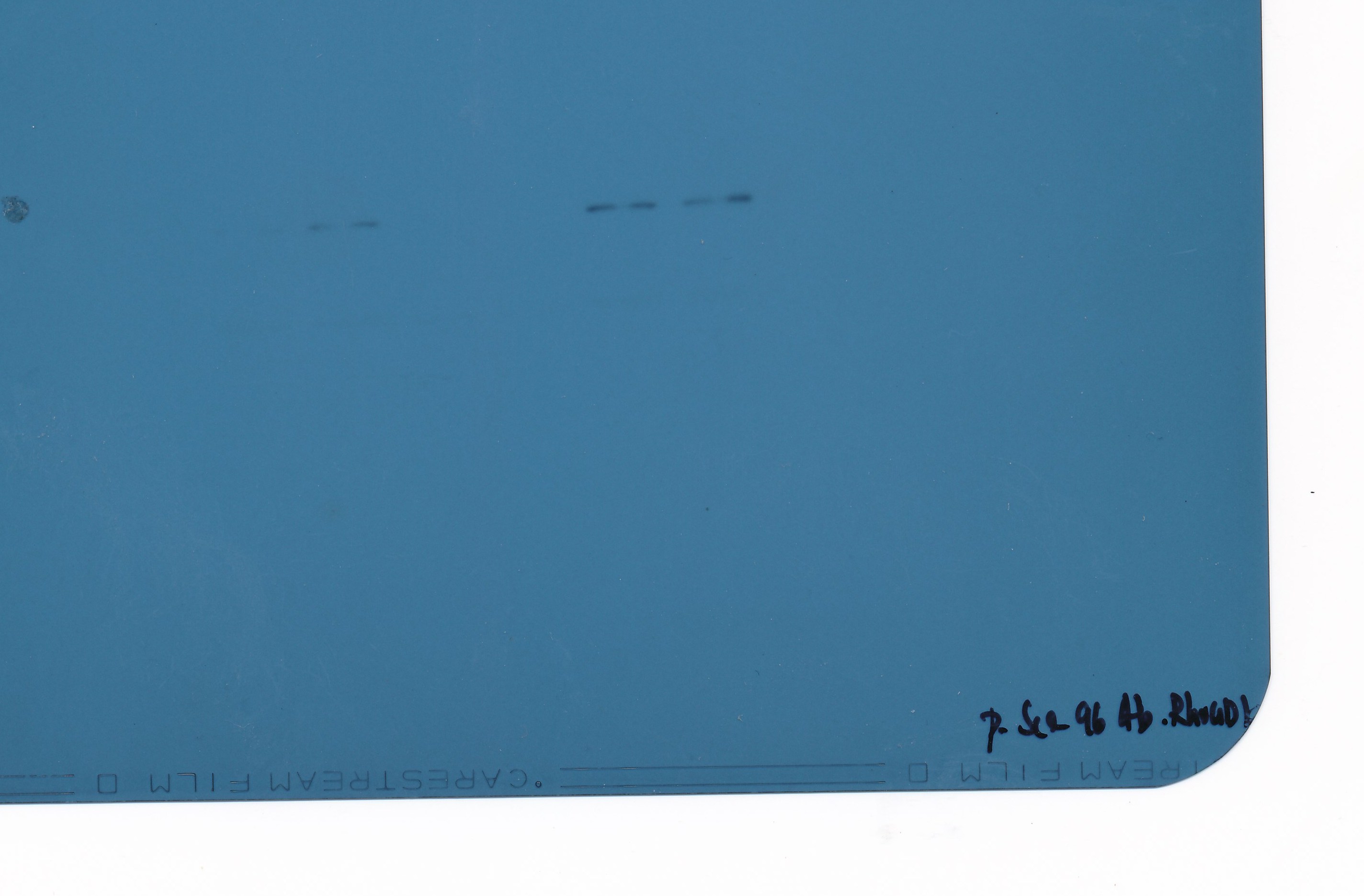

Supplement: Supplementary file 6 — Source Data Fig. 2 [file 44319_2024_64_MOESM6_ESM.zip › 2H/IP Flab:IB P-Ser96 RhoGDI.jpg]

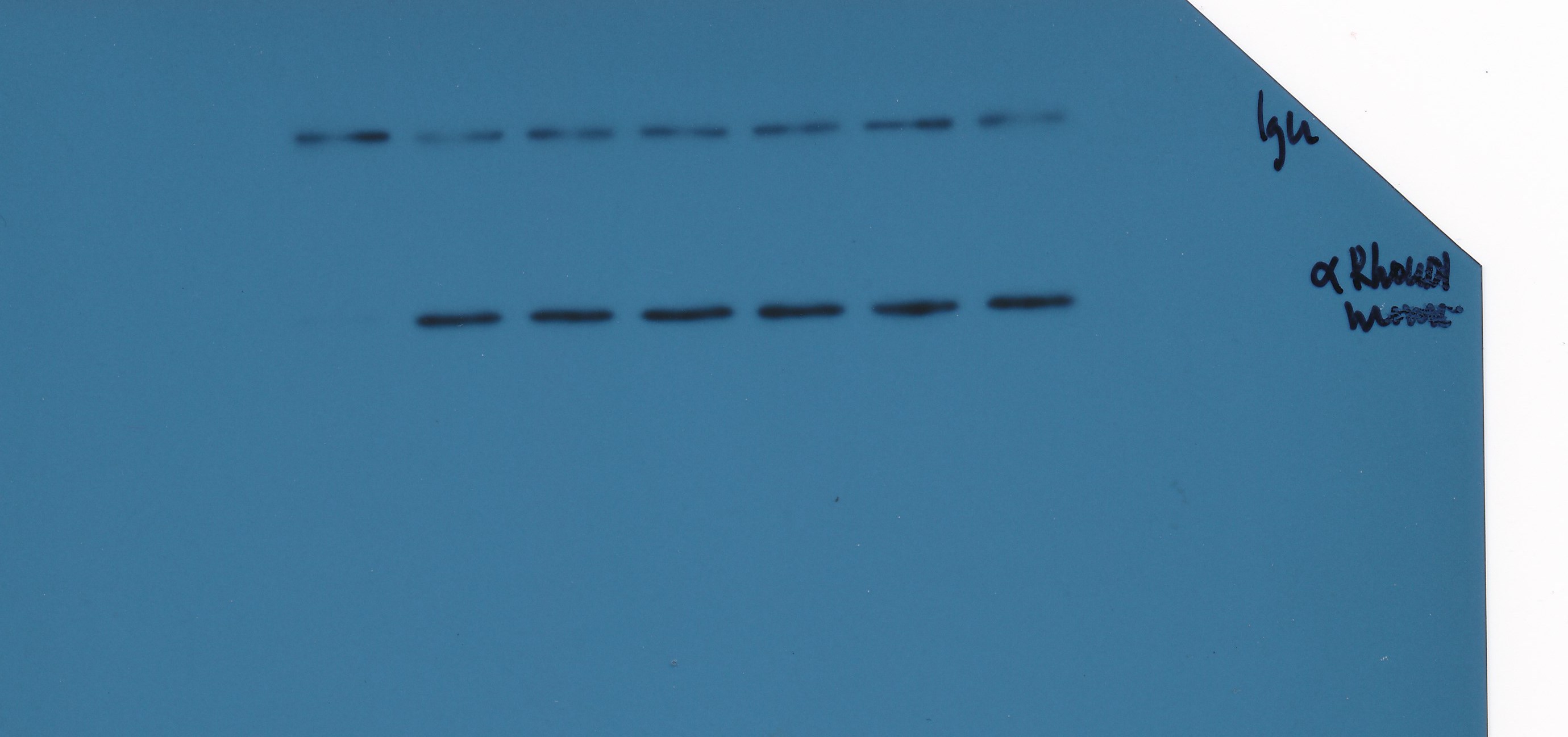

Supplement: Supplementary file 6 — Source Data Fig. 2 [file 44319_2024_64_MOESM6_ESM.zip › 2H/IP Flag:IB RhoGDI.jpg]

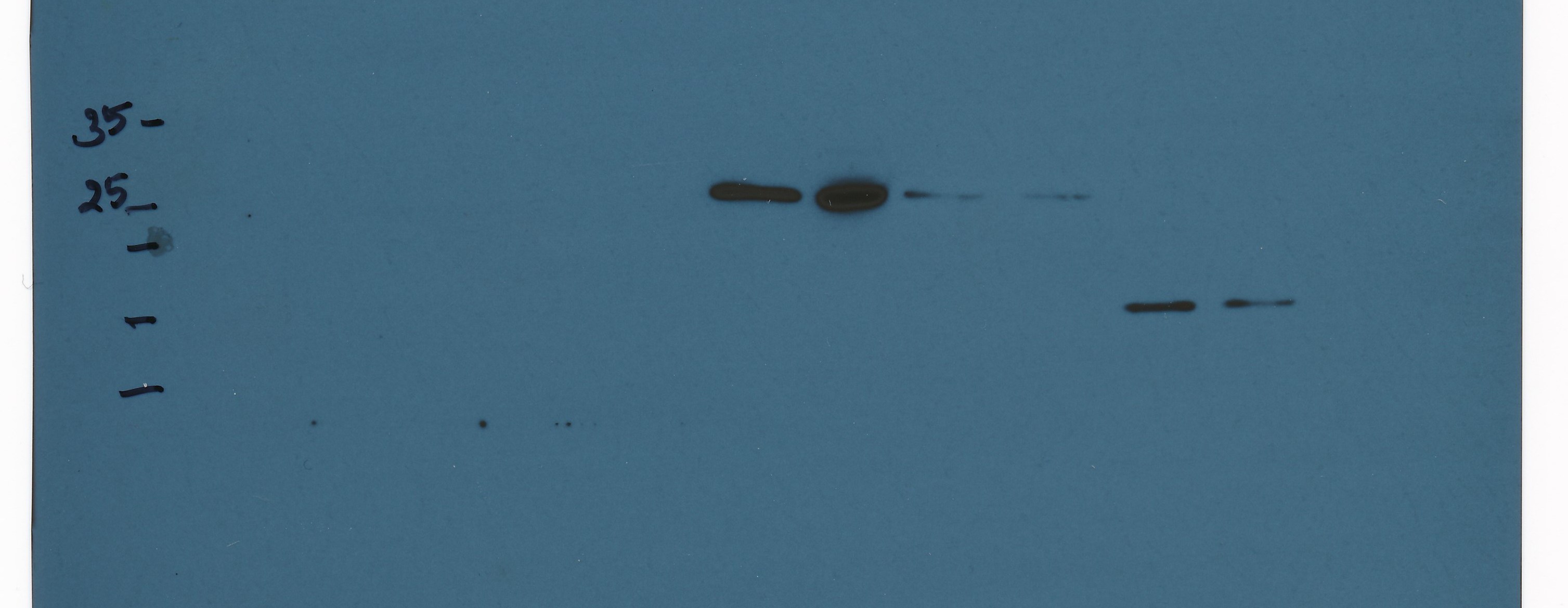

Supplement: Supplementary file 7 — Source Data Fig. 3 [file 44319_2024_64_MOESM7_ESM.zip › 3B/IP p75NTR:IB Flag (RhoGDI).jpeg]

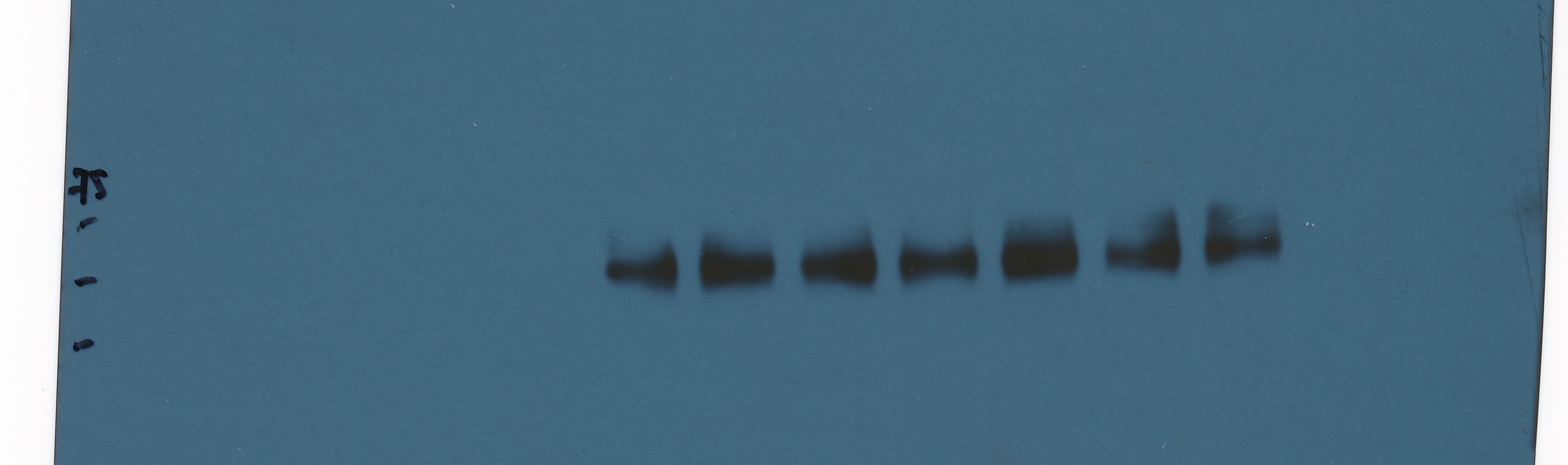

Supplement: Supplementary file 7 — Source Data Fig. 3 [file 44319_2024_64_MOESM7_ESM.zip › 3B/IP p75NTR:IB p75NTR.jpeg]

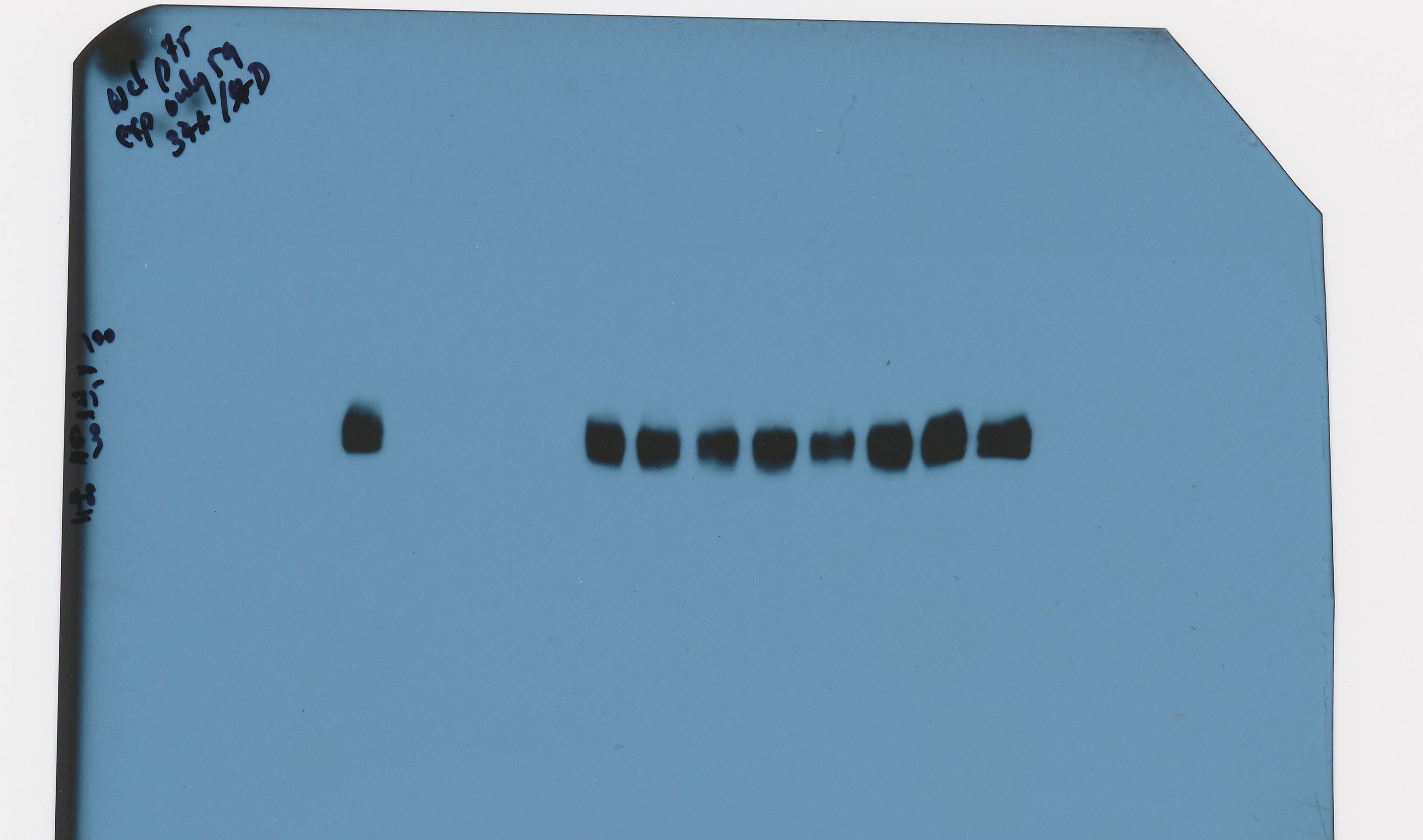

Supplement: Supplementary file 7 — Source Data Fig. 3 [file 44319_2024_64_MOESM7_ESM.zip › 3E/IP p75NTR:IB p75NTR.jpg]

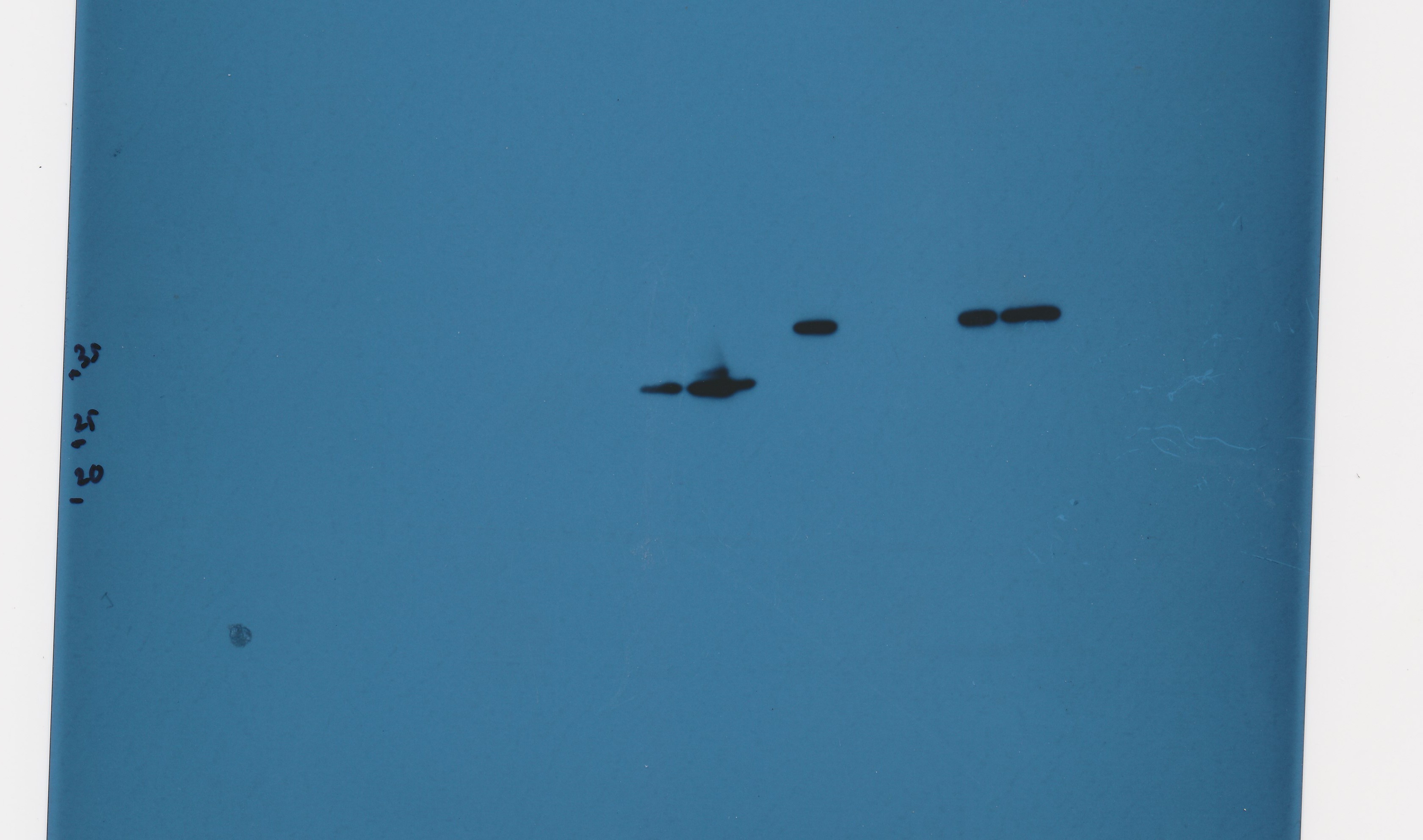

Supplement: Supplementary file 7 — Source Data Fig. 3 [file 44319_2024_64_MOESM7_ESM.zip › 3E/IP p75NTR:IB Flag (RhoGDI).jpg]

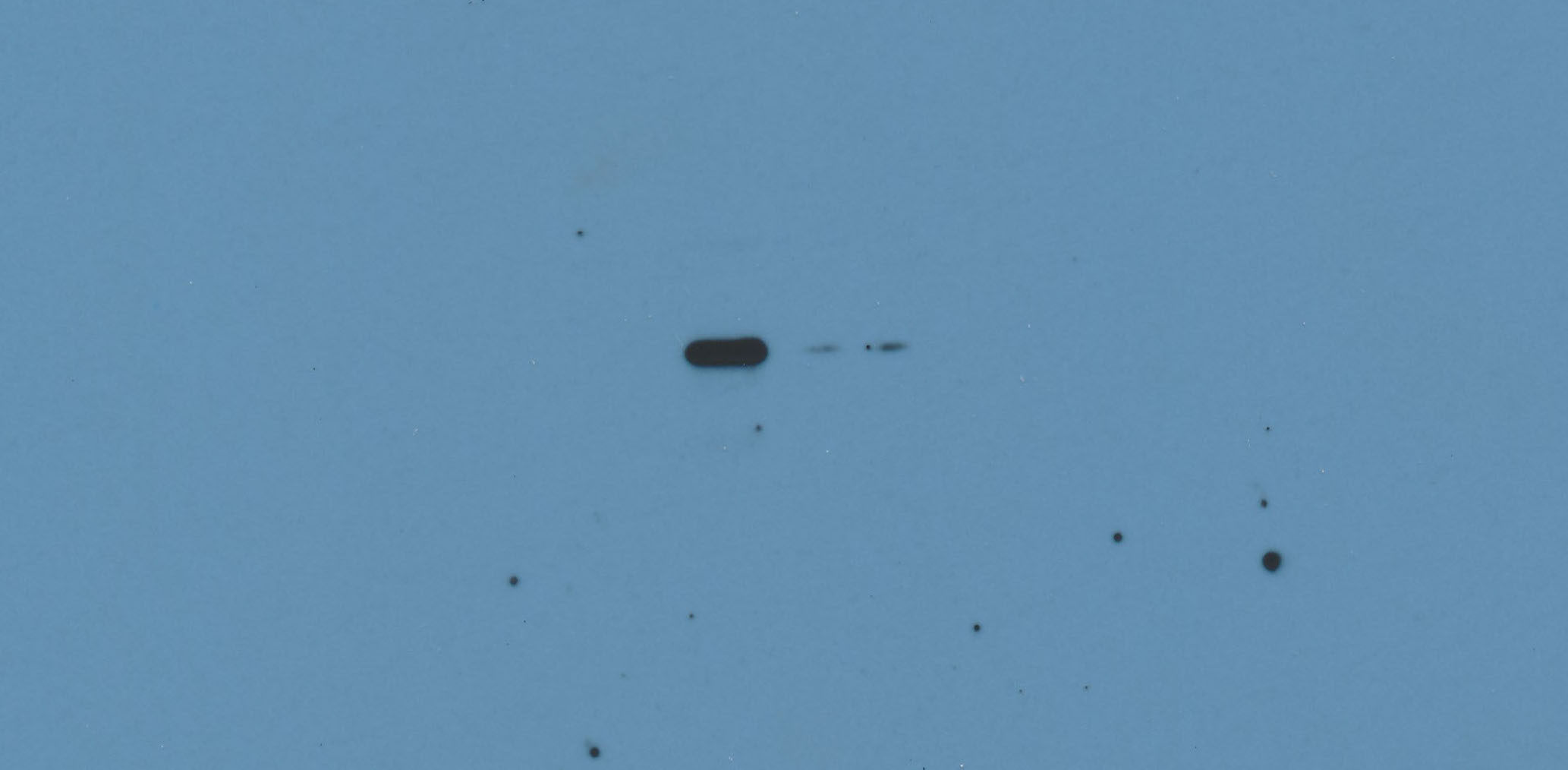

Supplement: Supplementary file 7 — Source Data Fig. 3 [file 44319_2024_64_MOESM7_ESM.zip › 3F/IP p75NTR:IB RhoGDI.jpg]

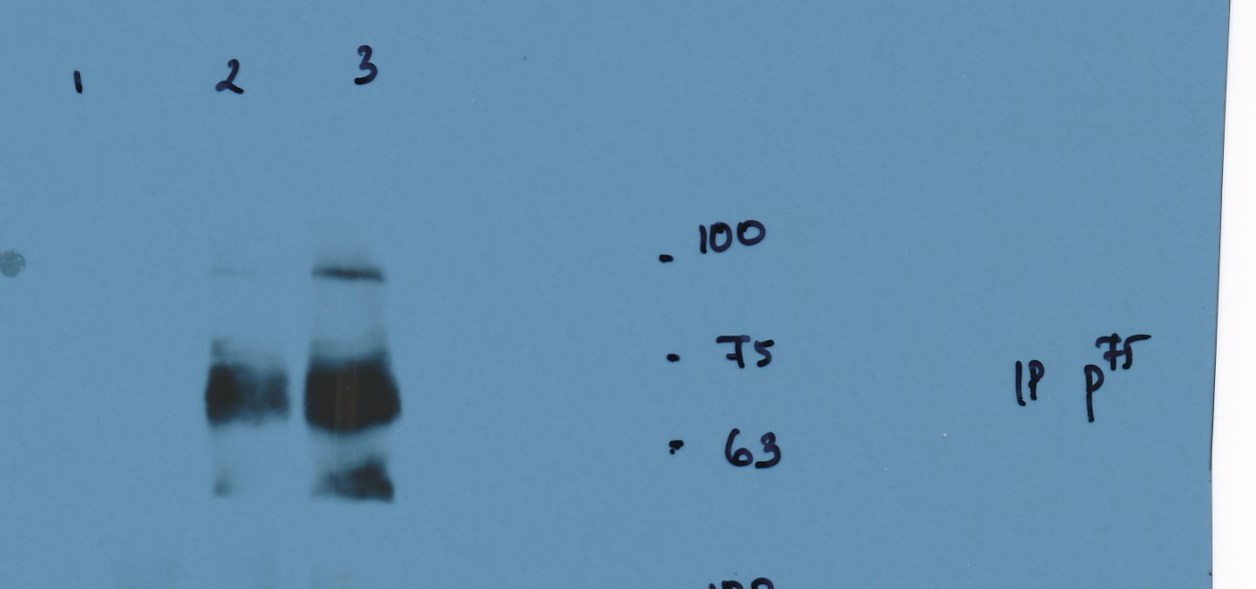

Supplement: Supplementary file 7 — Source Data Fig. 3 [file 44319_2024_64_MOESM7_ESM.zip › 3F/IP p75NTR:IB p75NTR.jpg]

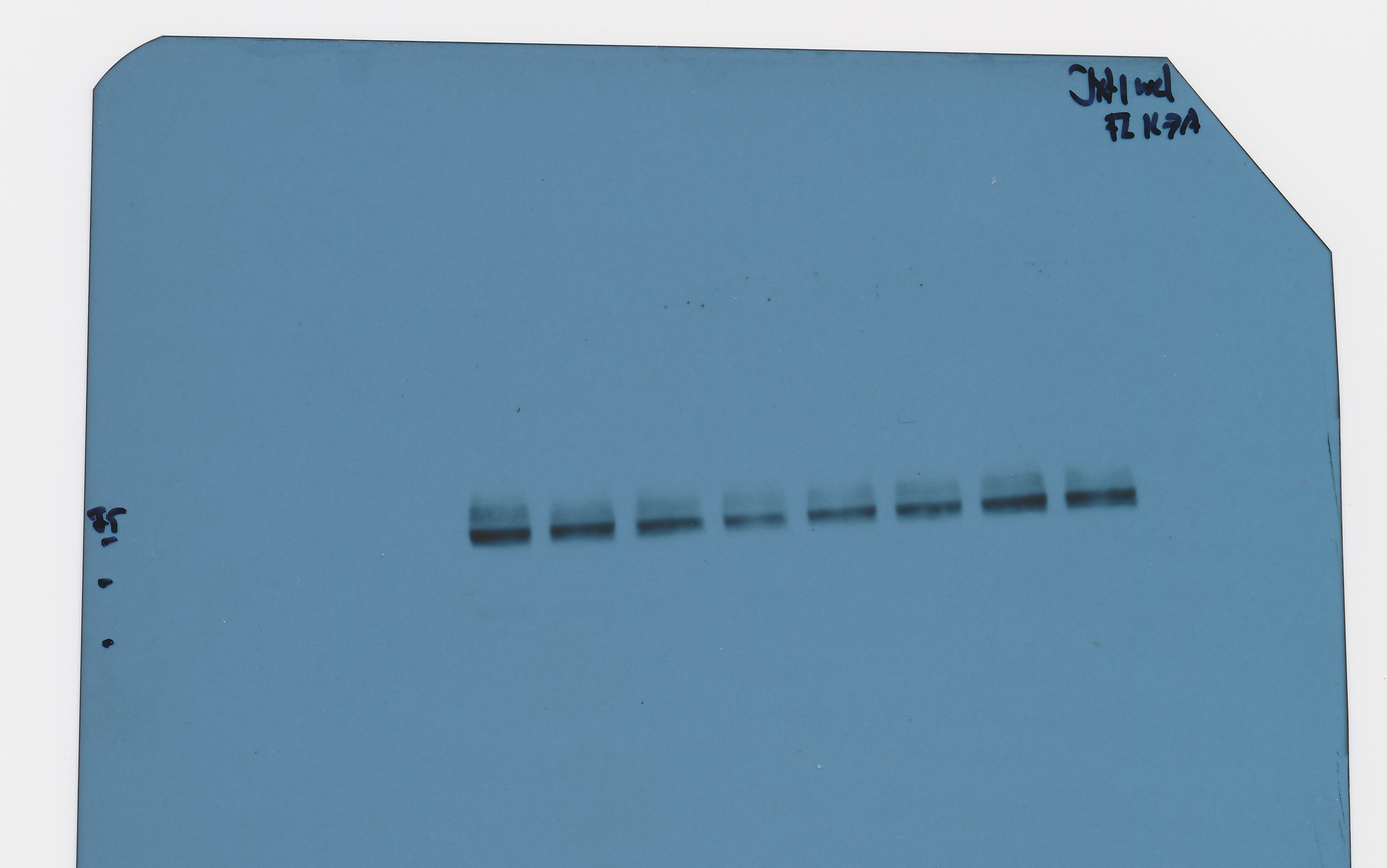

Supplement: Supplementary file 8 — Source Data Fig. 4 [file 44319_2024_64_MOESM8_ESM.zip › 4B/IP p75NTR:IB p75NTR.jpg]

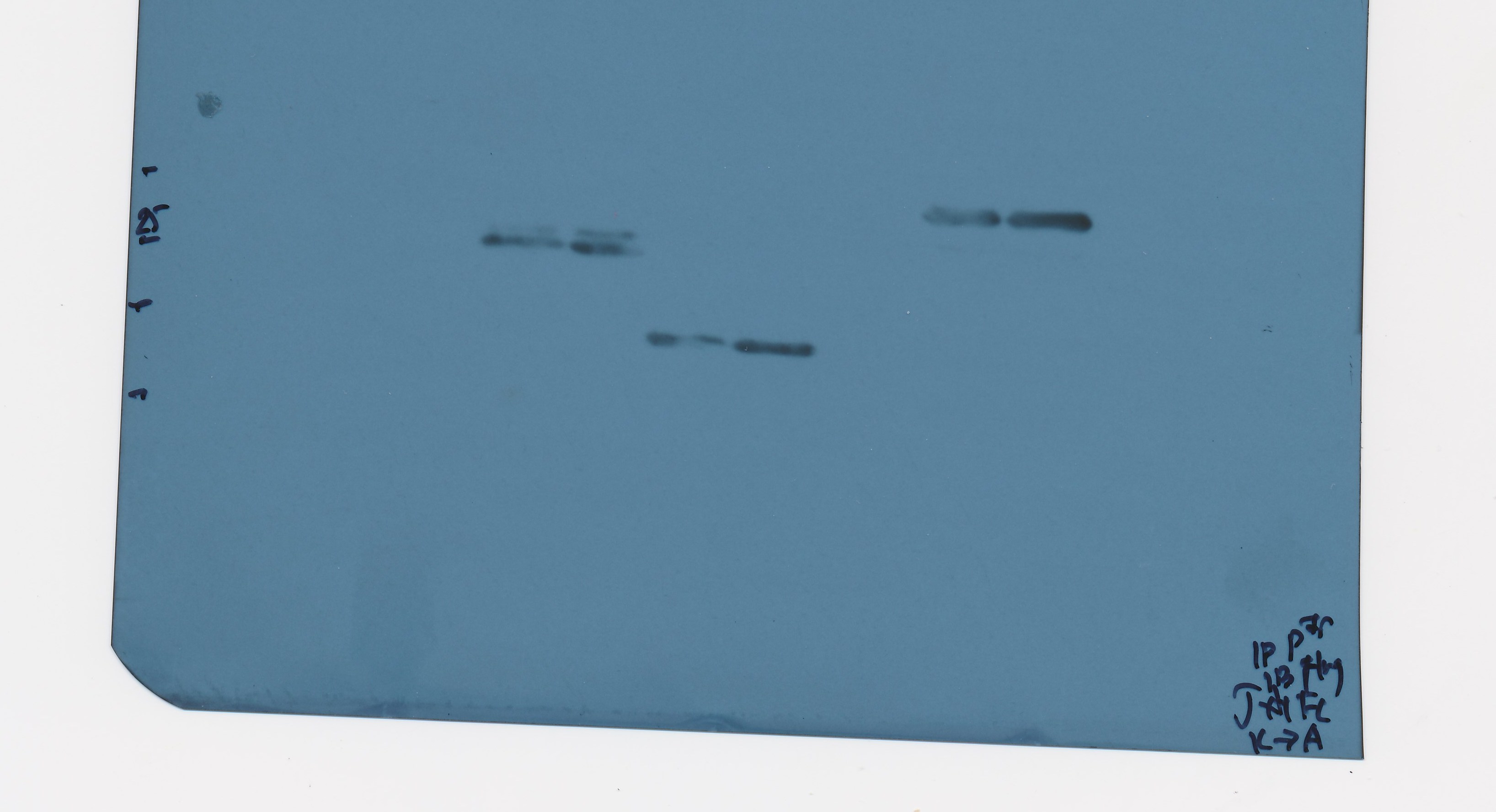

Supplement: Supplementary file 8 — Source Data Fig. 4 [file 44319_2024_64_MOESM8_ESM.zip › 4B/IP p75NTR:IB Flag (RhoGDI).jpg]

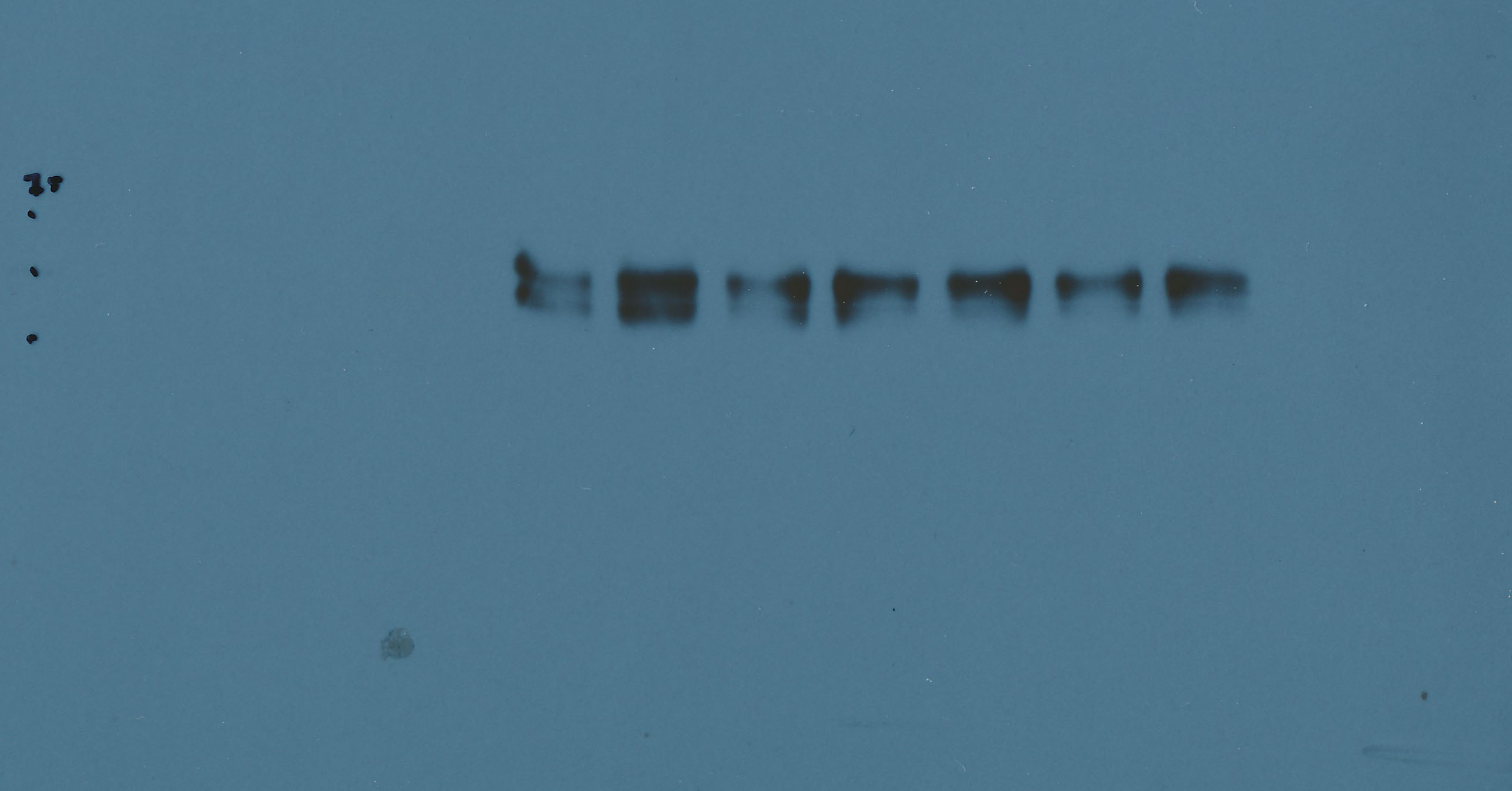

Supplement: Supplementary file 8 — Source Data Fig. 4 [file 44319_2024_64_MOESM8_ESM.zip › 4C/IP p75NTR:IB p75NTR.jpg]

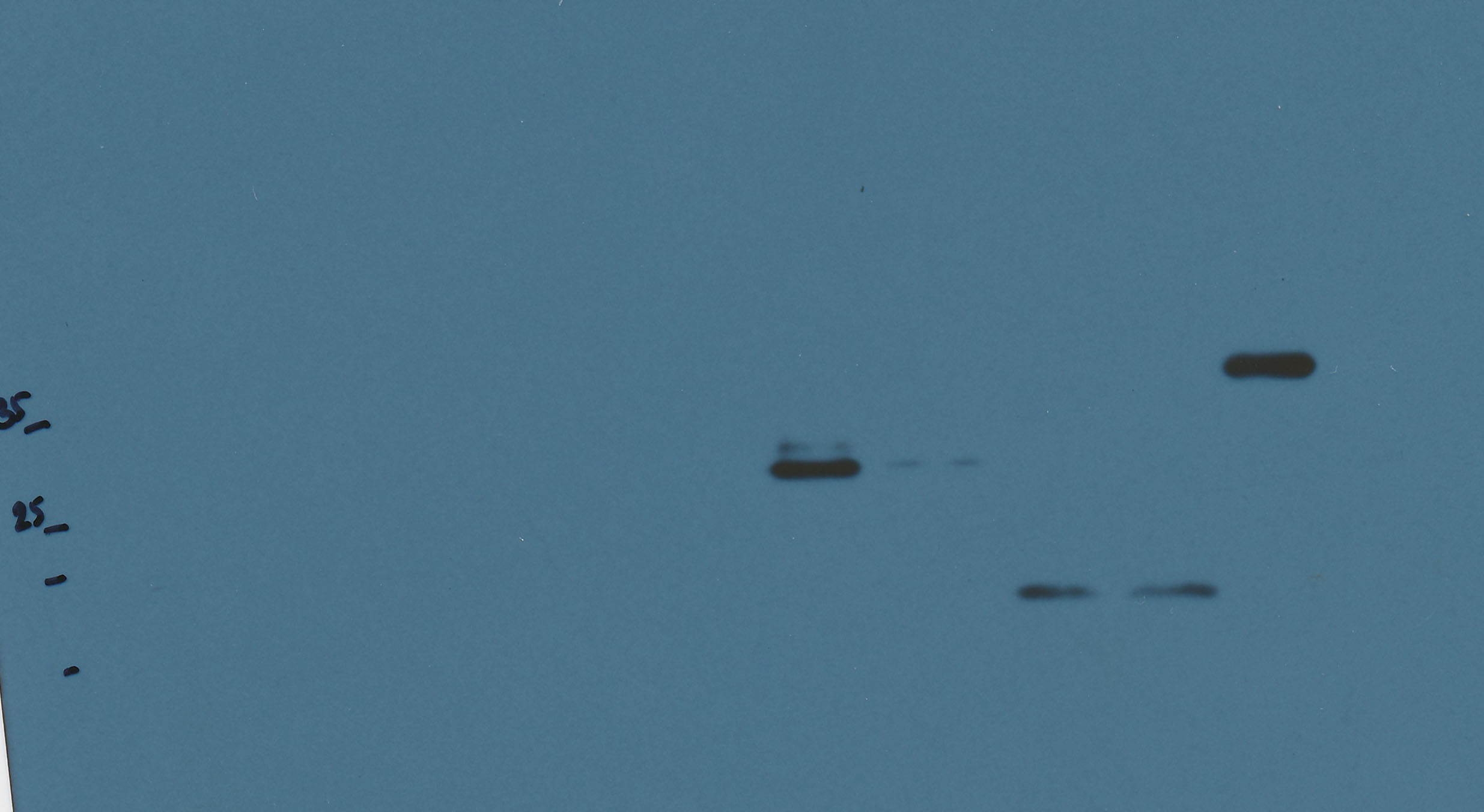

Supplement: Supplementary file 8 — Source Data Fig. 4 [file 44319_2024_64_MOESM8_ESM.zip › 4C/IP p75NTR:IB Flag (RhoGDI).jpg]

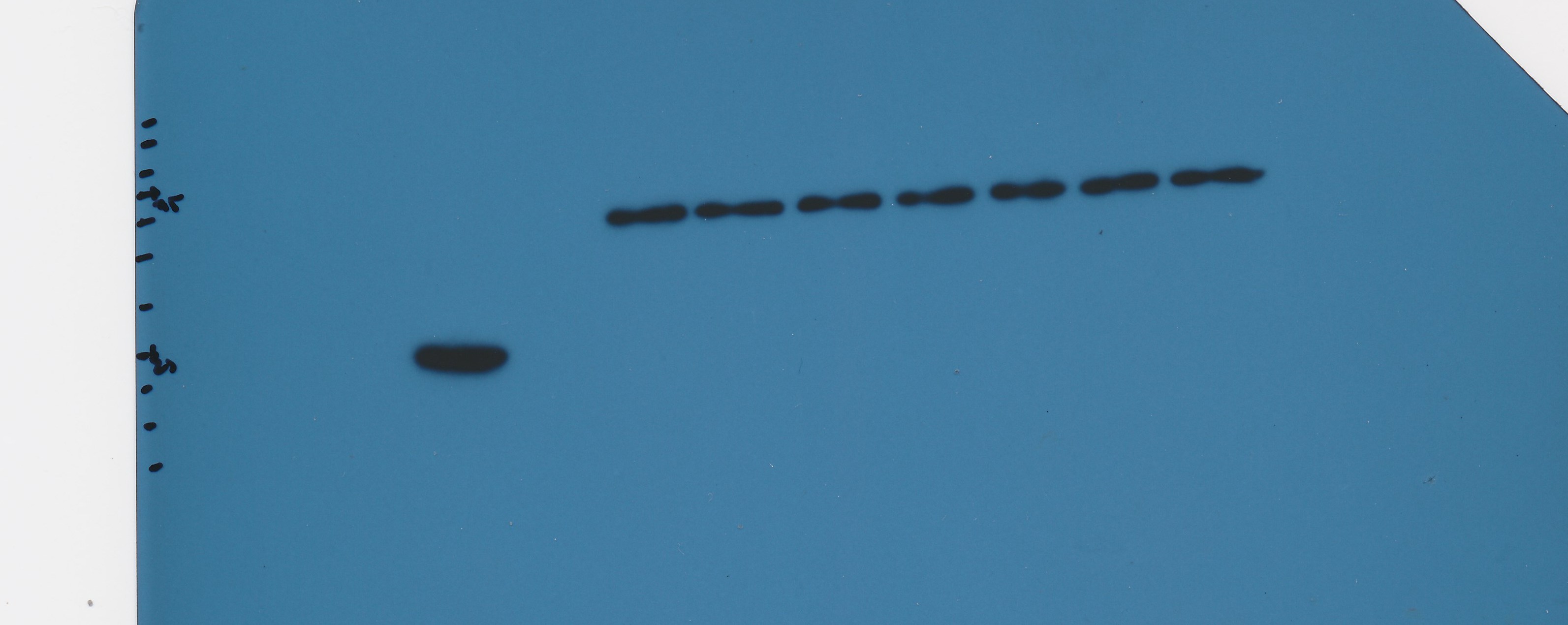

Supplement: Supplementary file 8 — Source Data Fig. 4 [file 44319_2024_64_MOESM8_ESM.zip › 4D/IP HA:IB HA (p75NTR).jpg]

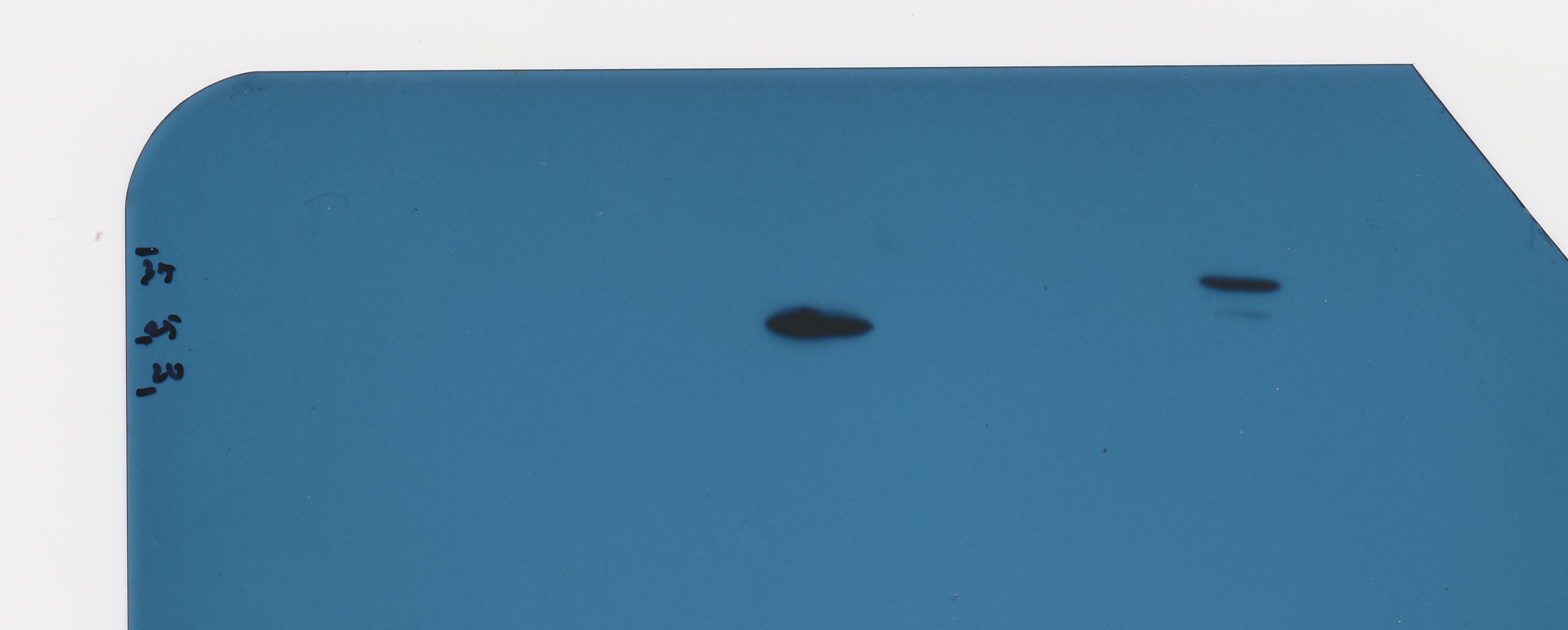

Supplement: Supplementary file 8 — Source Data Fig. 4 [file 44319_2024_64_MOESM8_ESM.zip › 4D/IP HA(p75NTR):IB Flag (RhoGDI).jpg]

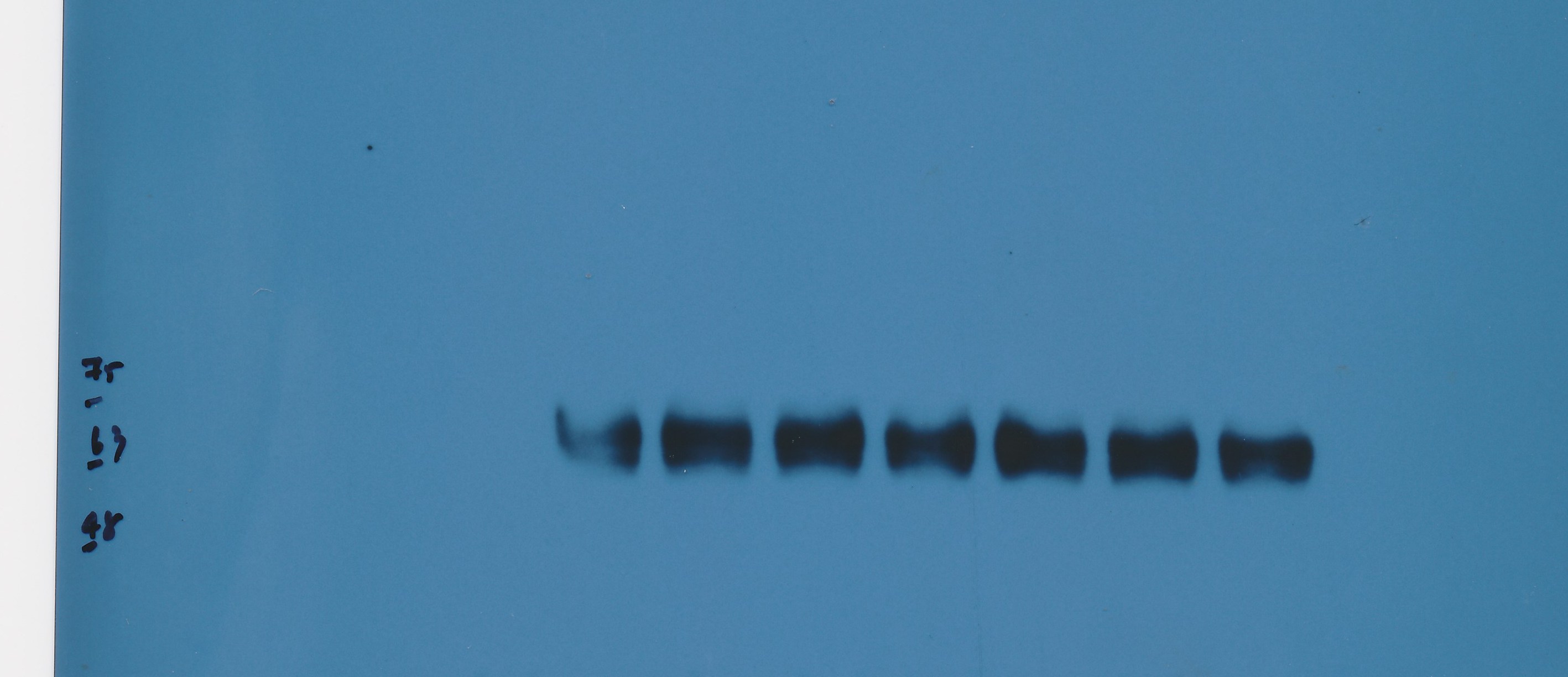

Supplement: Supplementary file 8 — Source Data Fig. 4 [file 44319_2024_64_MOESM8_ESM.zip › 4E/IP p75NTR: IB p75NTR.jpg]

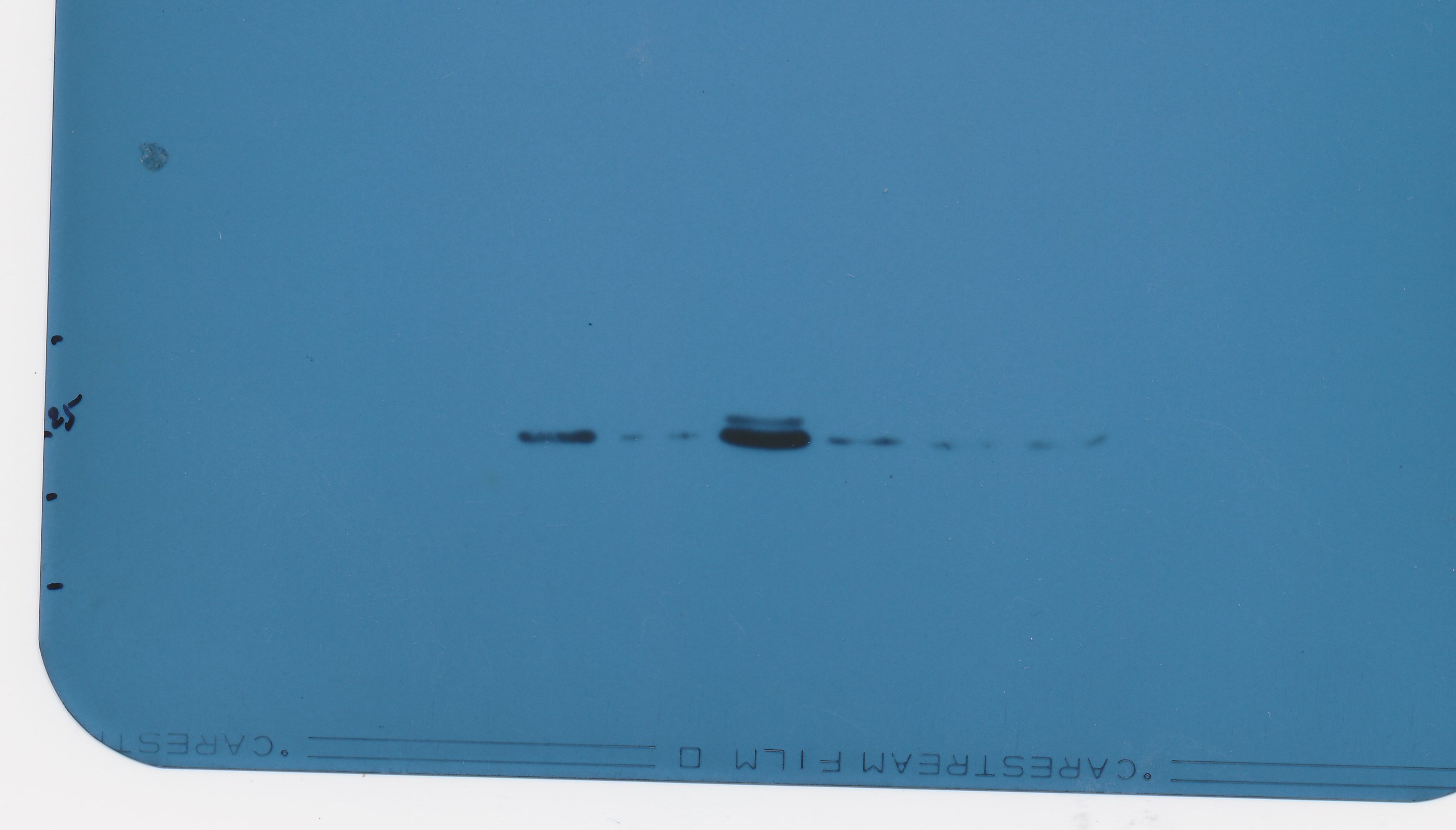

Supplement: Supplementary file 8 — Source Data Fig. 4 [file 44319_2024_64_MOESM8_ESM.zip › 4E/IP p75NTR:IB Flag (RhoGDI).jpg]

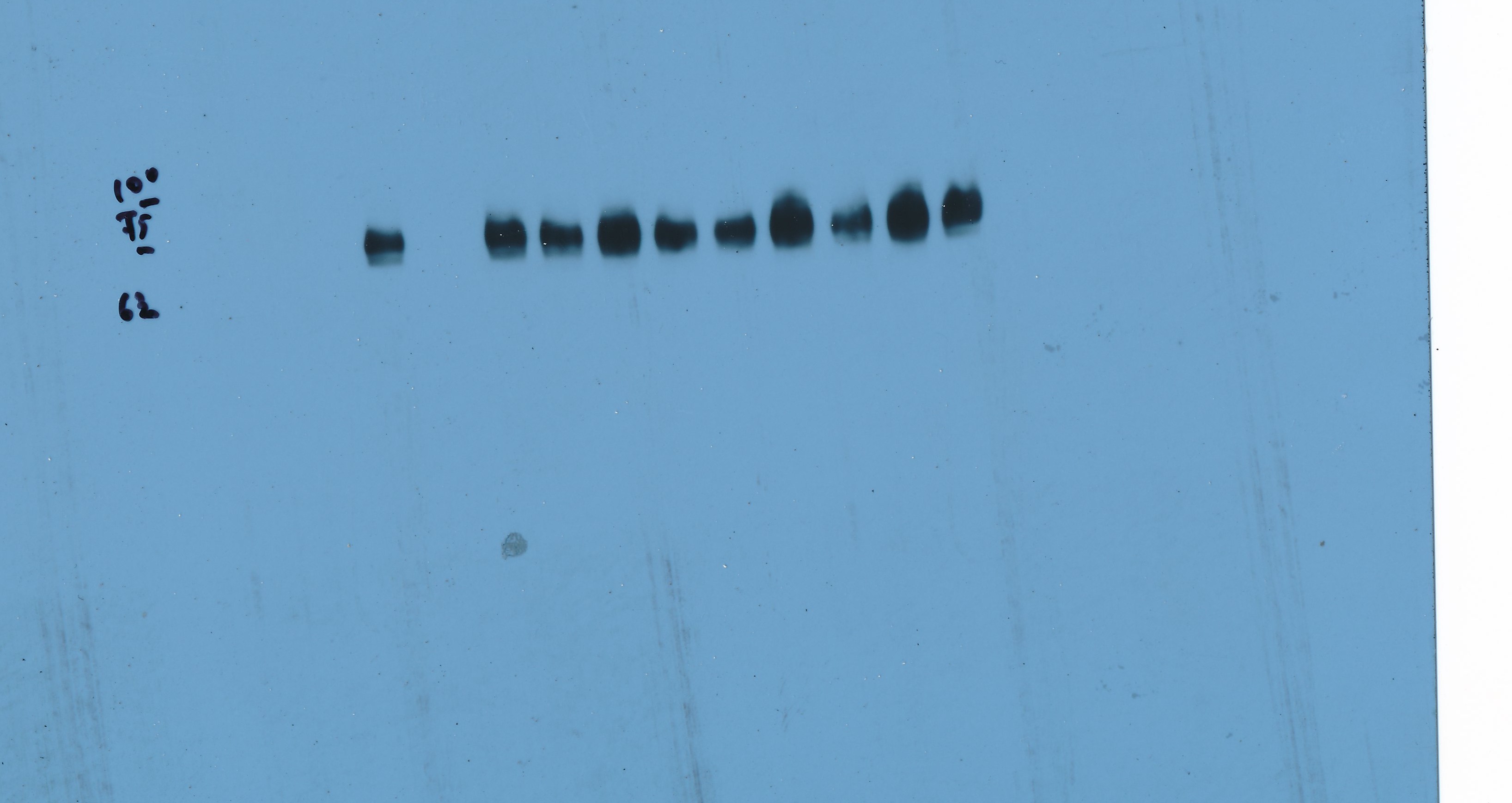

Supplement: Supplementary file 8 — Source Data Fig. 4 [file 44319_2024_64_MOESM8_ESM.zip › 4G/IP p75NTR:IB p75NTR.jpg]

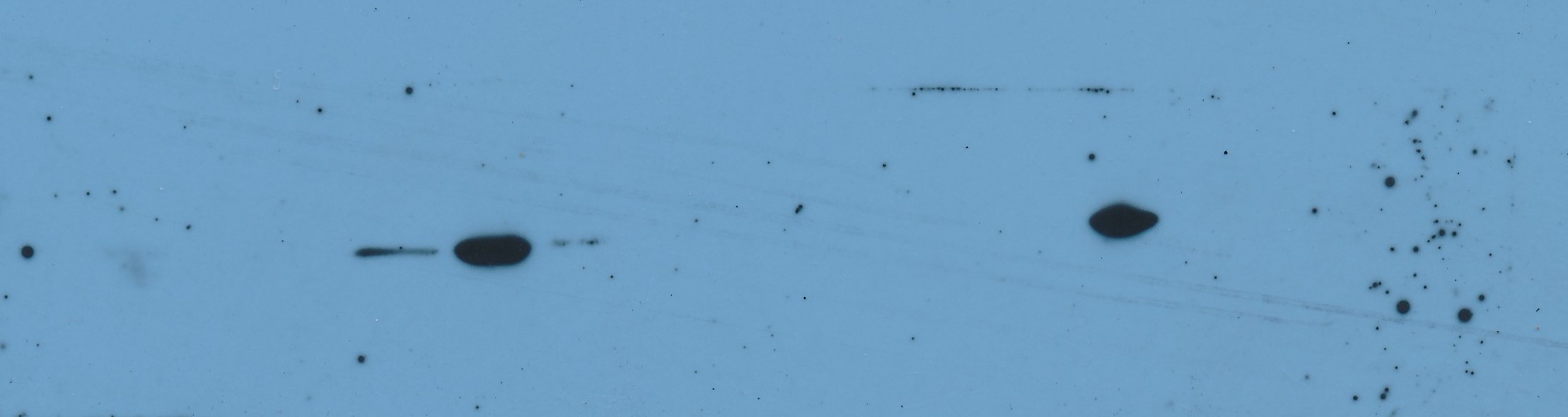

Supplement: Supplementary file 8 — Source Data Fig. 4 [file 44319_2024_64_MOESM8_ESM.zip › 4G/IP p75NTR:IB Flag (RhoGDI).jpg]

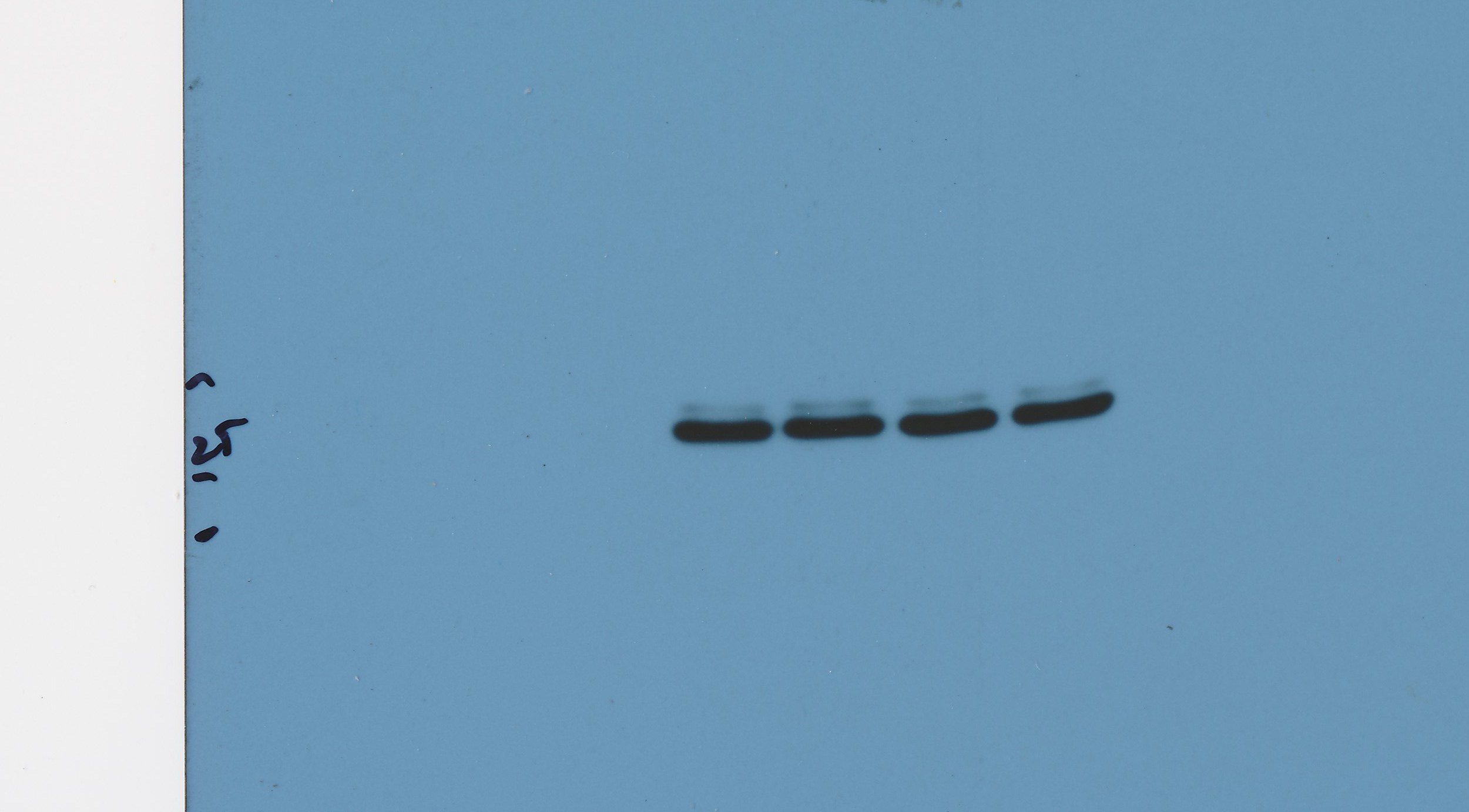

Supplement: Supplementary file 8 — Source Data Fig. 4 [file 44319_2024_64_MOESM8_ESM.zip › 4H/IP Flag (RhoGDI):IB RhoGDI.jpg]

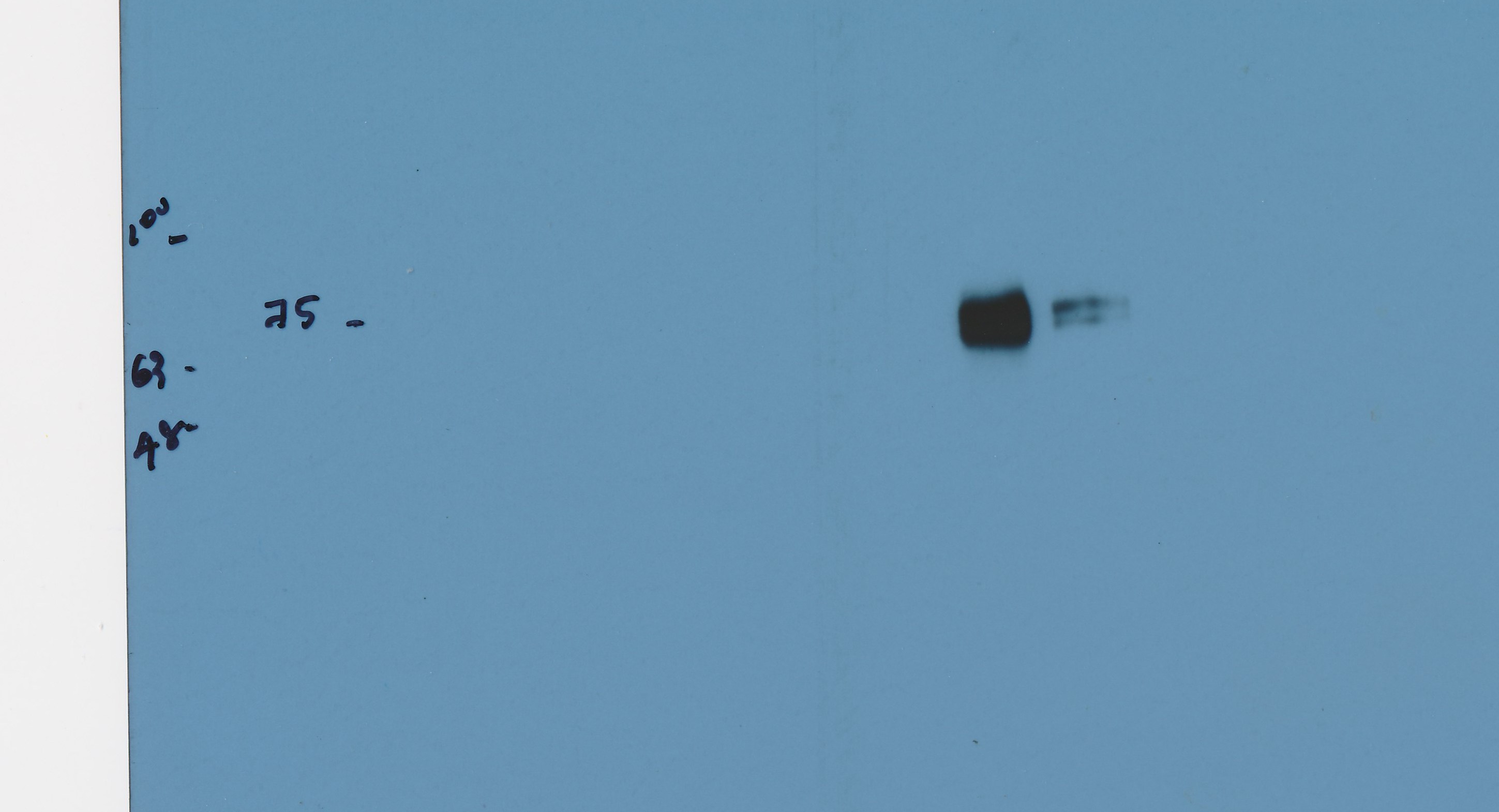

Supplement: Supplementary file 8 — Source Data Fig. 4 [file 44319_2024_64_MOESM8_ESM.zip › 4H/IP Flag (RhoGDI):IB p75NTR.jpg]

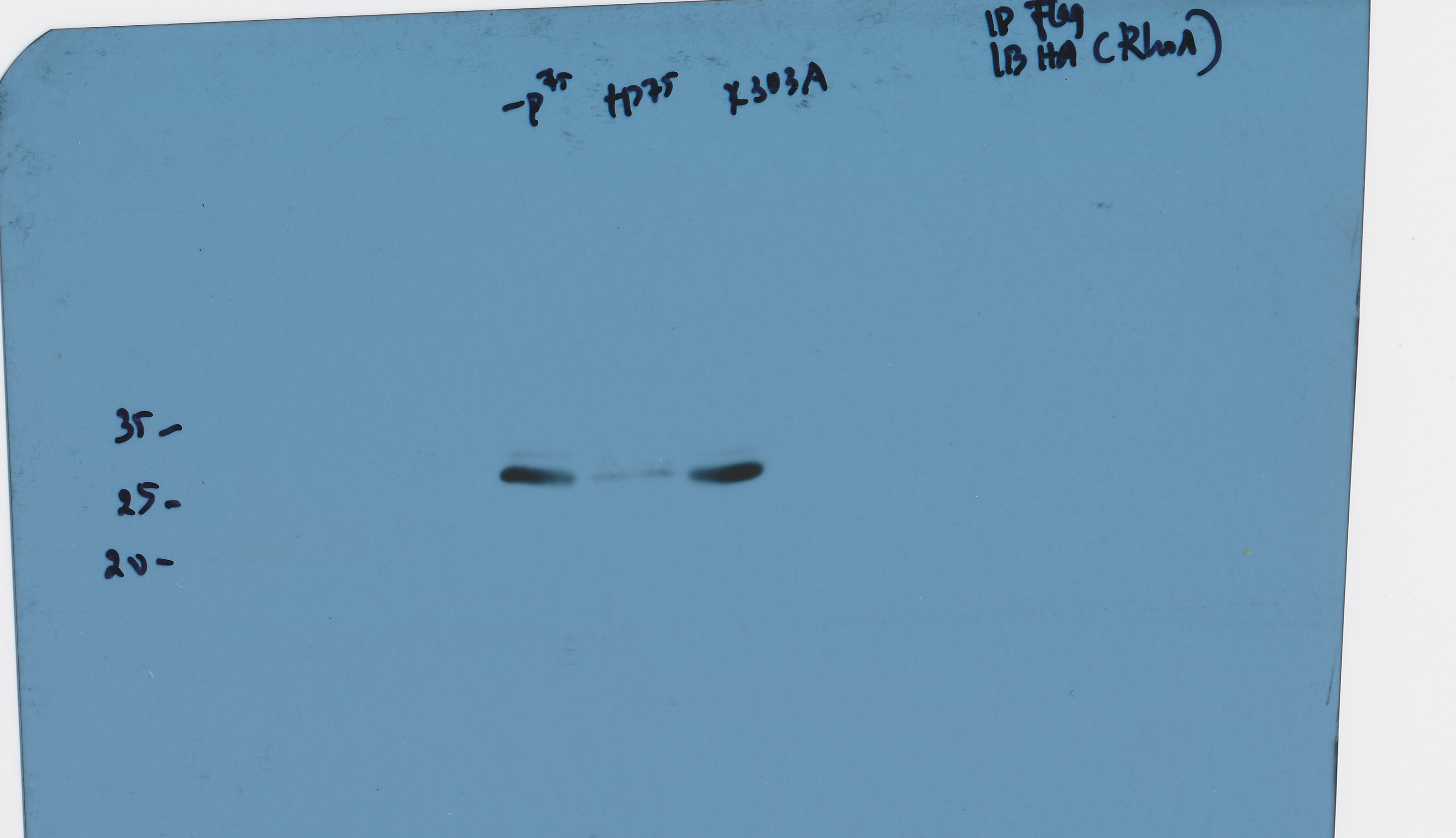

Supplement: Supplementary file 8 — Source Data Fig. 4 [file 44319_2024_64_MOESM8_ESM.zip › 4H/IP Flag (RhoGDI):IB HA (RhoA).jpg]

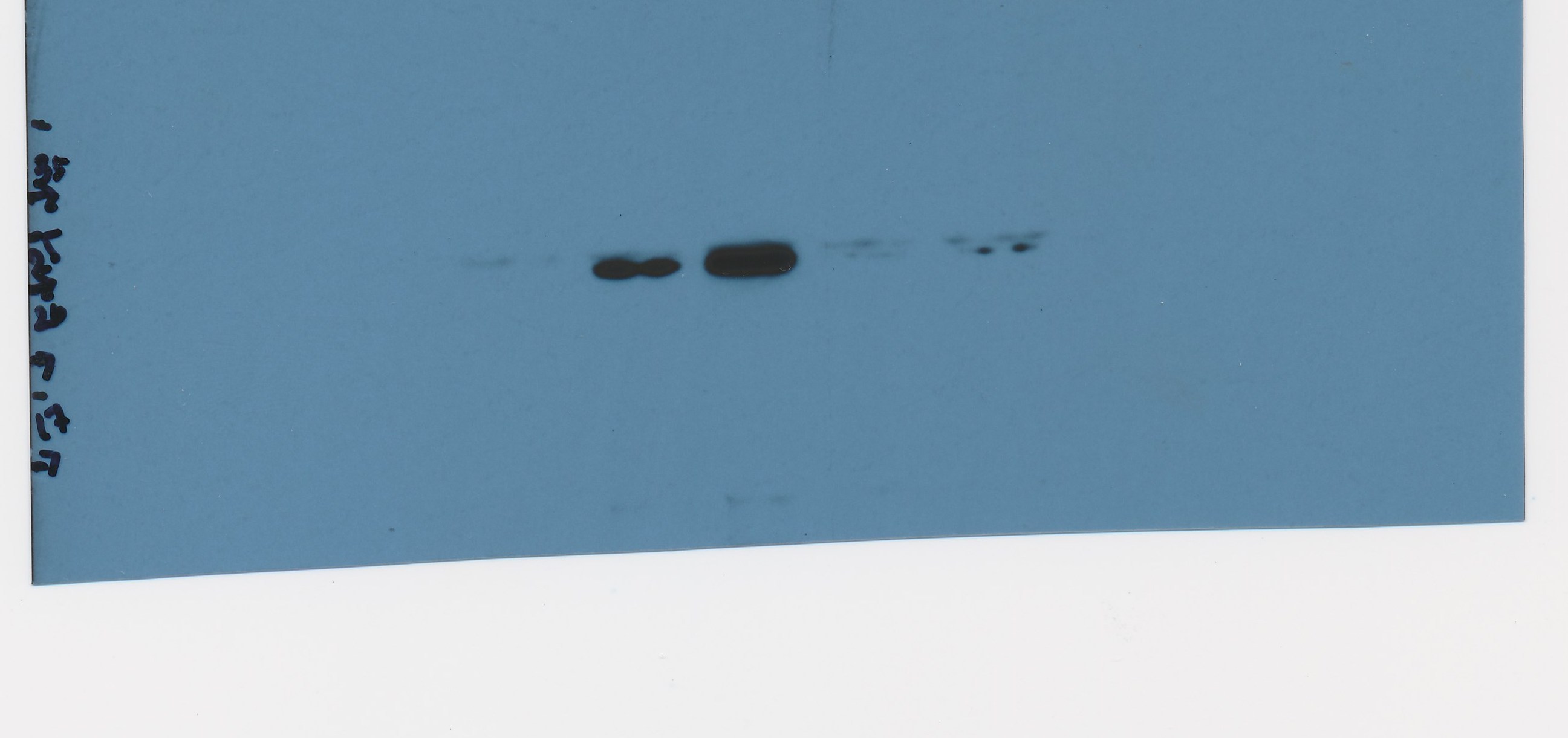

Supplement: Supplementary file 8 — Source Data Fig. 4 [file 44319_2024_64_MOESM8_ESM.zip › 4J/IP HA (p75NTR):IB RhoGDI.jpg]

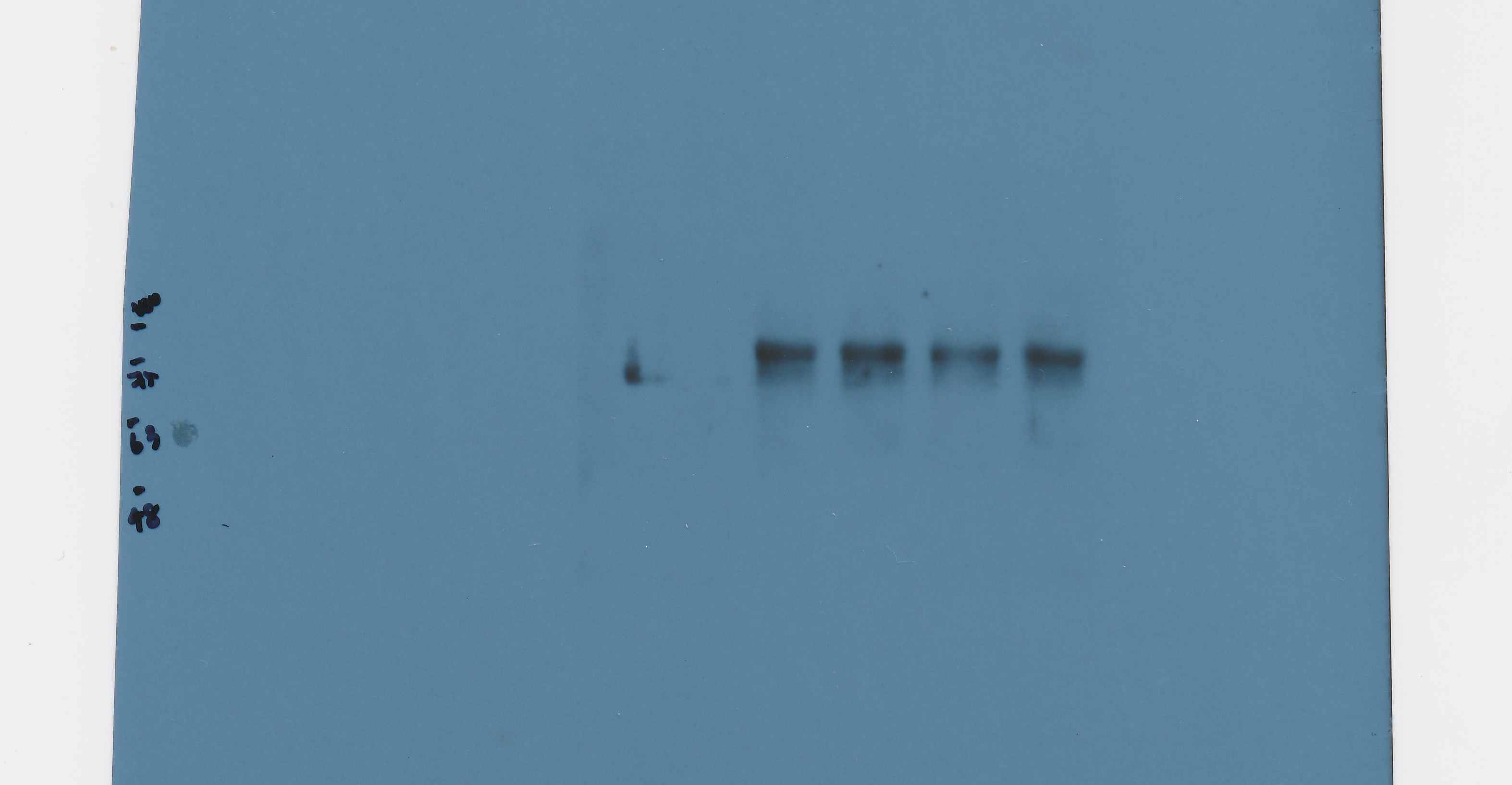

Supplement: Supplementary file 8 — Source Data Fig. 4 [file 44319_2024_64_MOESM8_ESM.zip › 4J/IP HA (p75NTR):IB p75NTR.jpg]

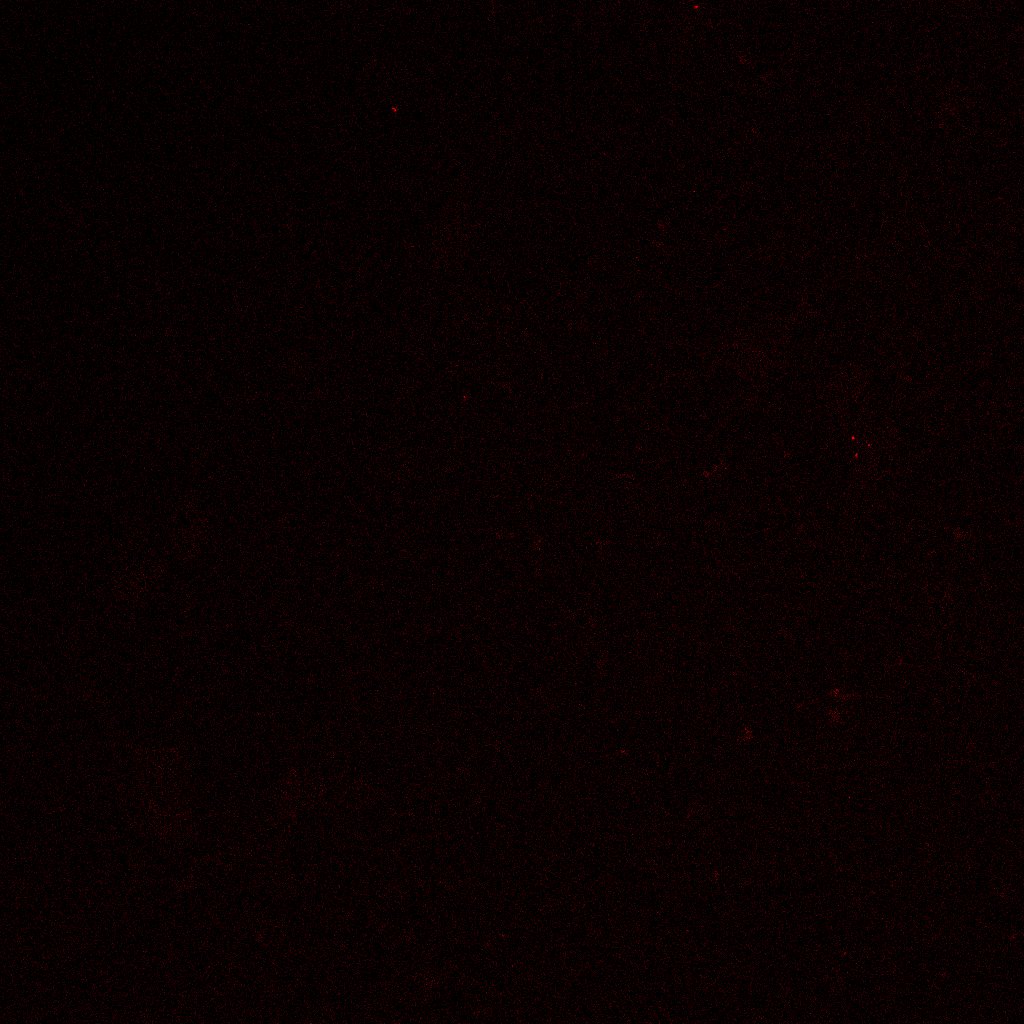

Supplement: Supplementary file 8 — Source Data Fig. 4 [file 44319_2024_64_MOESM8_ESM.zip › 4K/p75NTR K303A/K303A PLA.jpg]

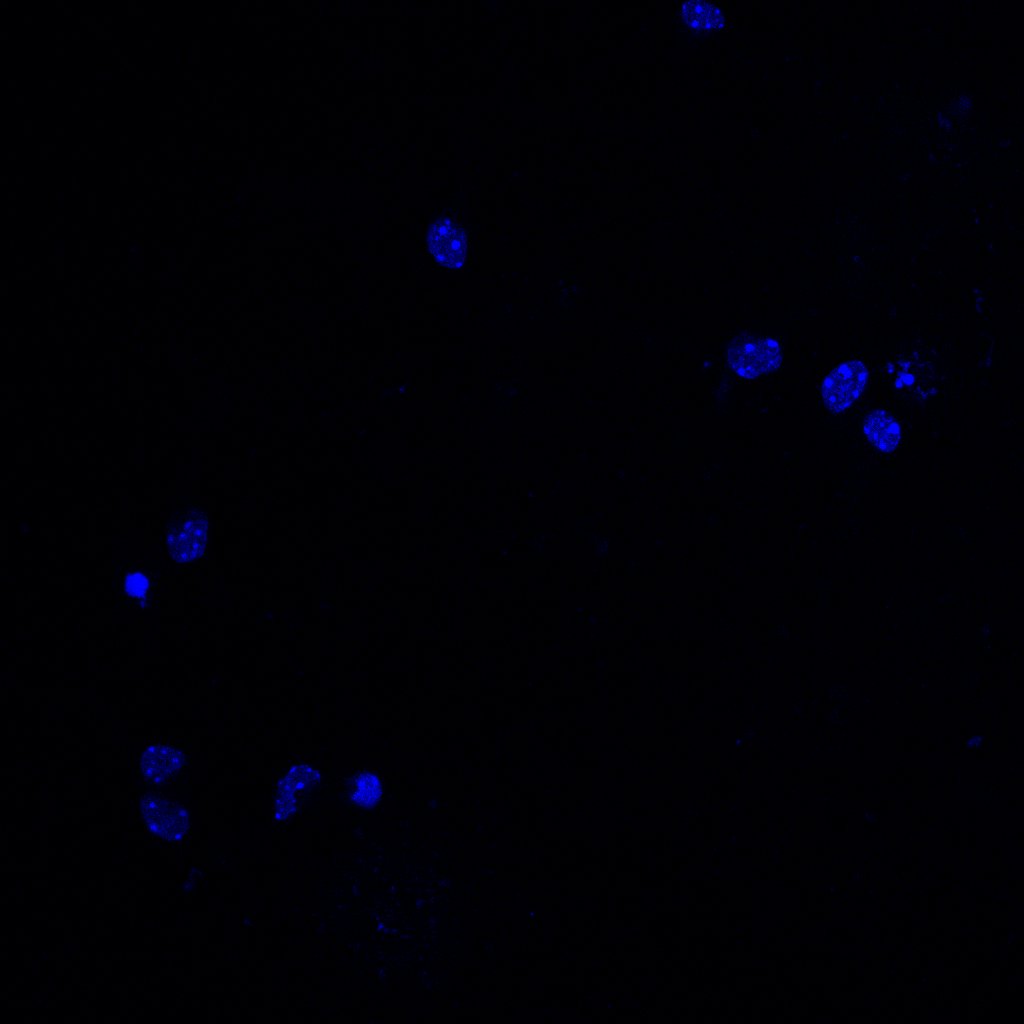

Supplement: Supplementary file 8 — Source Data Fig. 4 [file 44319_2024_64_MOESM8_ESM.zip › 4K/p75NTR K303A/K303A Dapi.jpg]

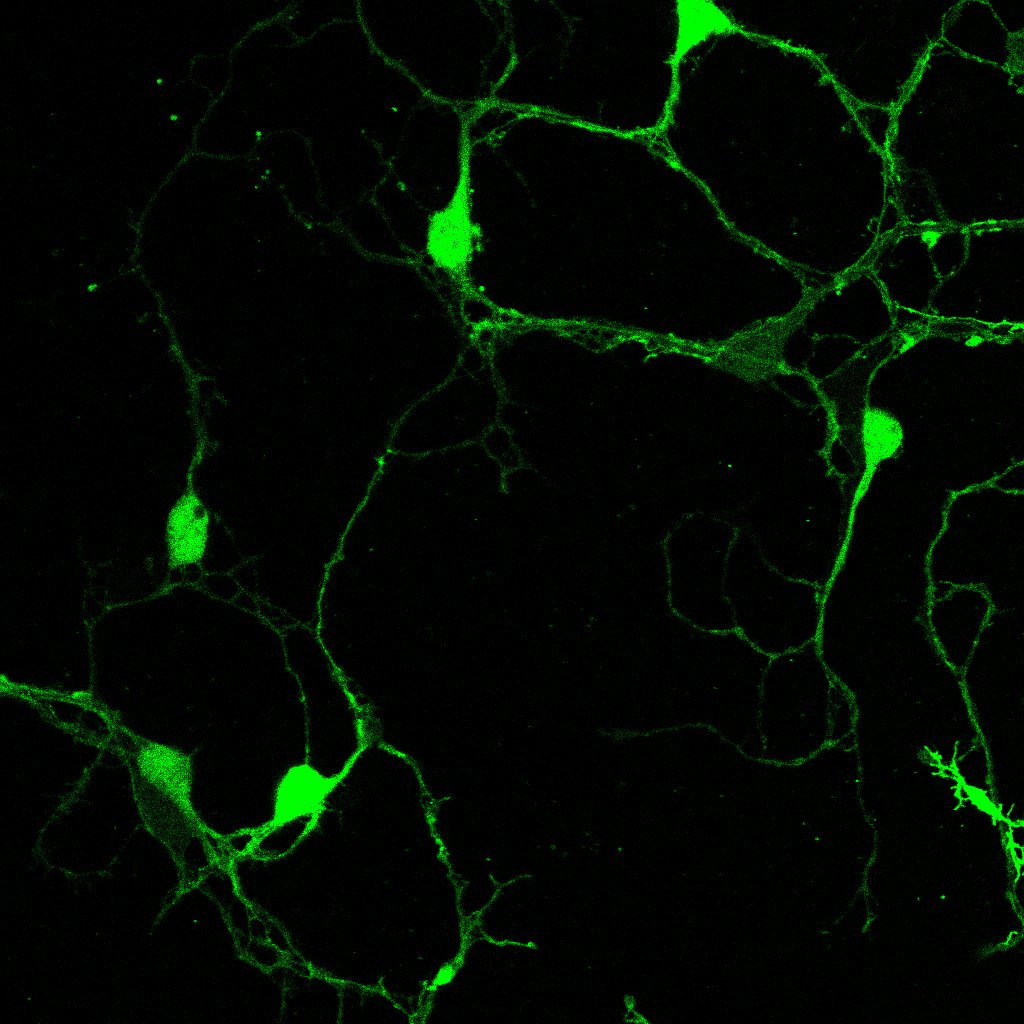

Supplement: Supplementary file 8 — Source Data Fig. 4 [file 44319_2024_64_MOESM8_ESM.zip › 4K/p75NTR K303A/K303A GFP.jpg]

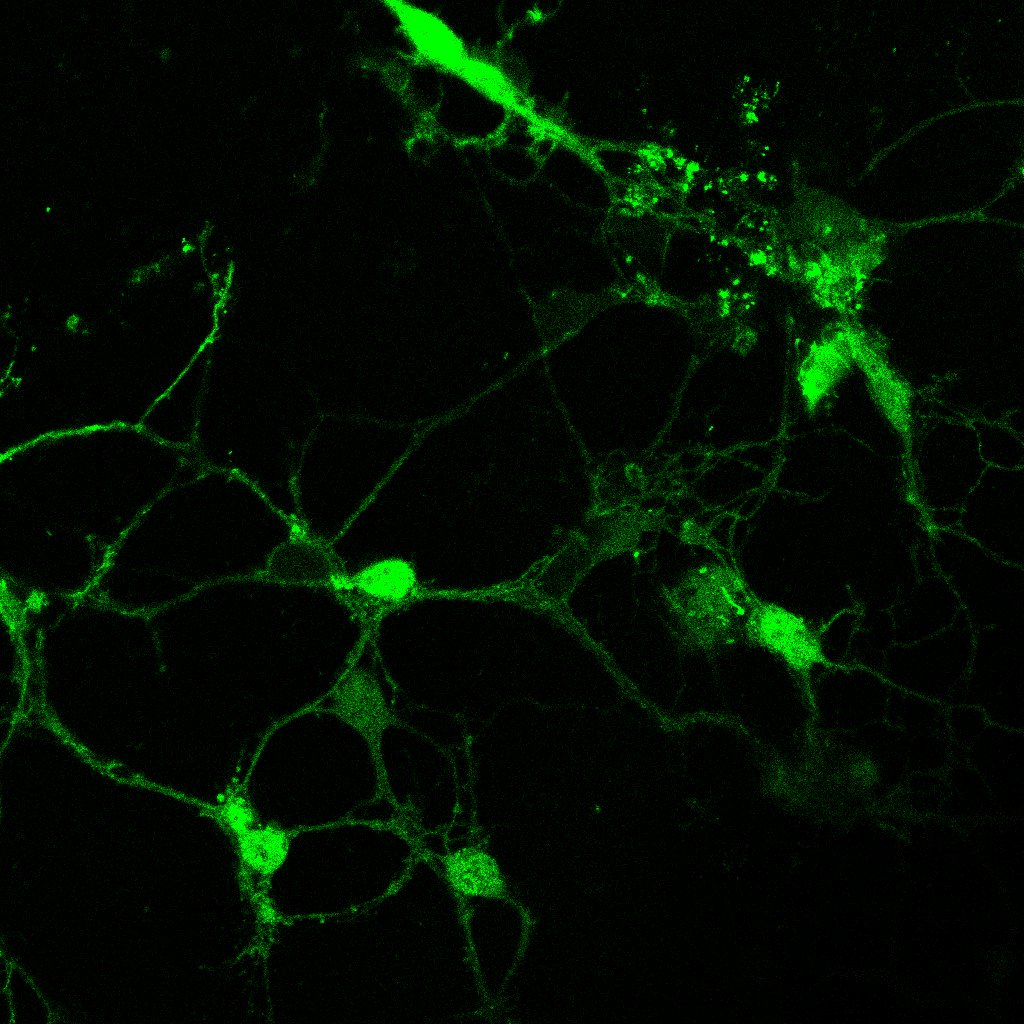

Supplement: Supplementary file 8 — Source Data Fig. 4 [file 44319_2024_64_MOESM8_ESM.zip › 4K/p75NTR WT/WT GFP.jpg]

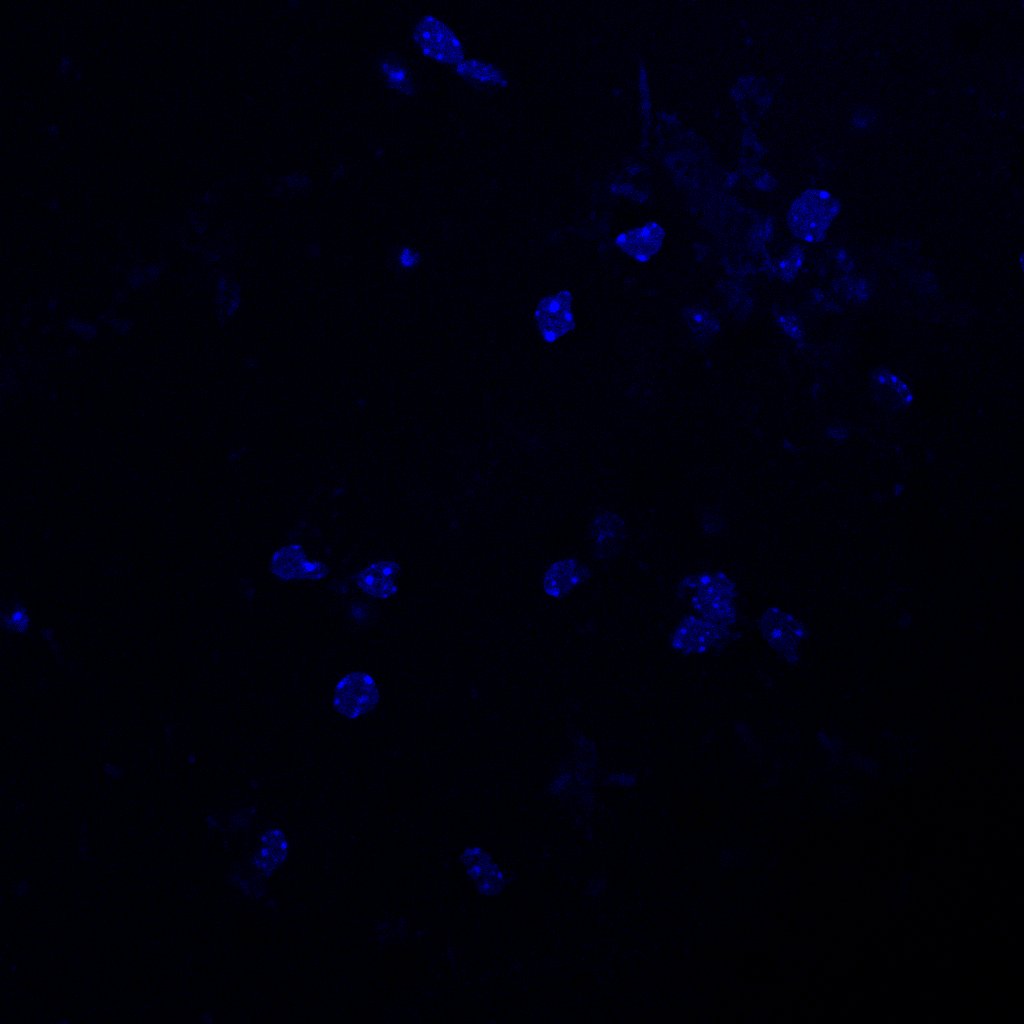

Supplement: Supplementary file 8 — Source Data Fig. 4 [file 44319_2024_64_MOESM8_ESM.zip › 4K/p75NTR WT/WT Dapi.jpg]

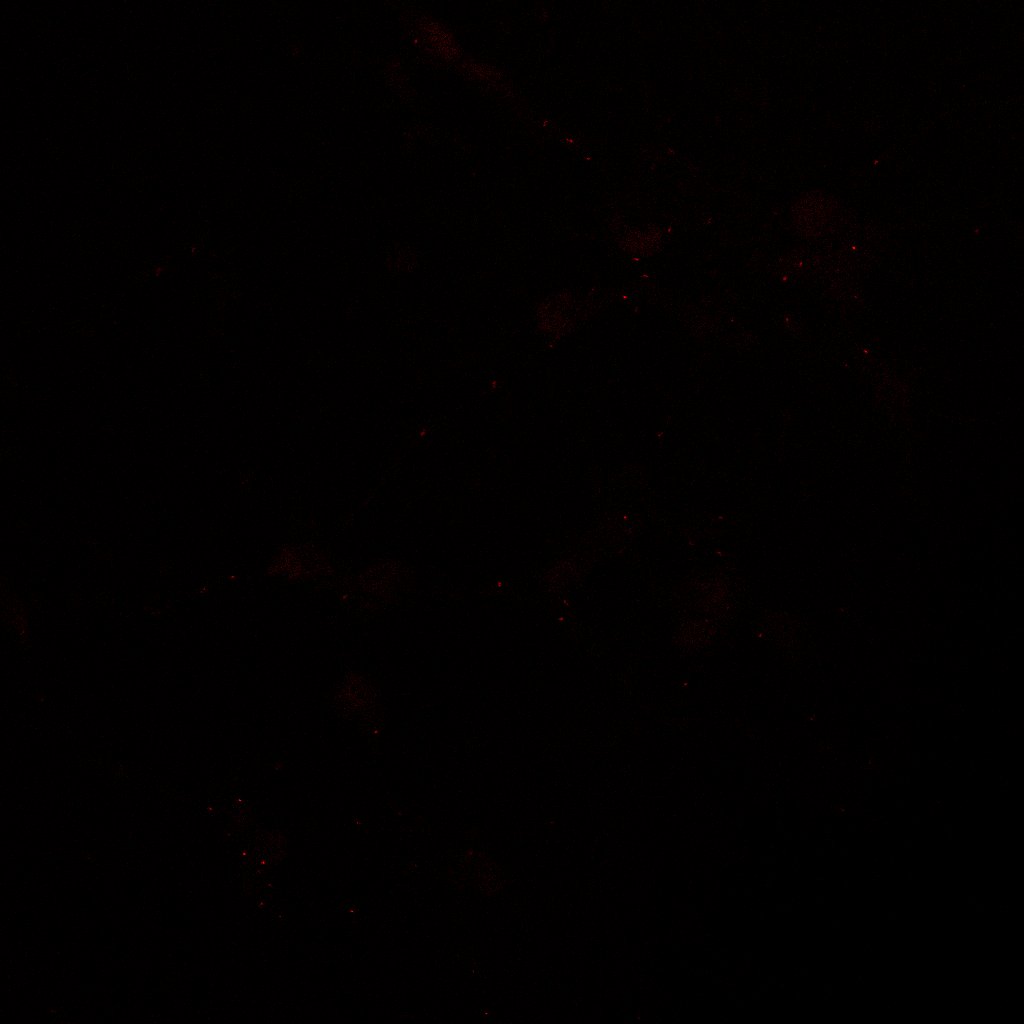

Supplement: Supplementary file 8 — Source Data Fig. 4 [file 44319_2024_64_MOESM8_ESM.zip › 4K/p75NTR WT/WT PLA.jpg]

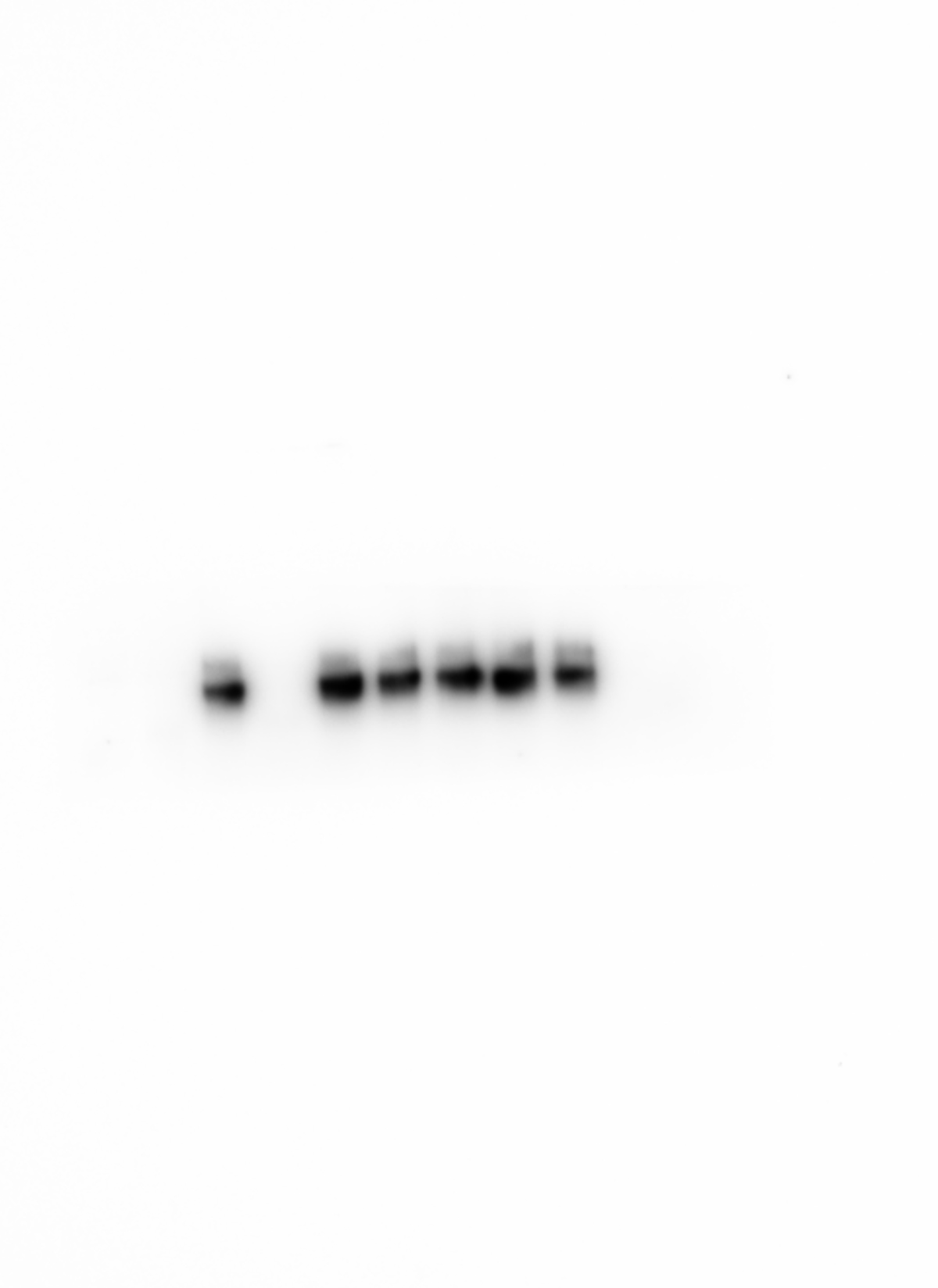

Supplement: Supplementary file 10 — Source Data Fig. 6 [file 44319_2024_64_MOESM10_ESM.zip › 6A/HA_CoIP.jpg]

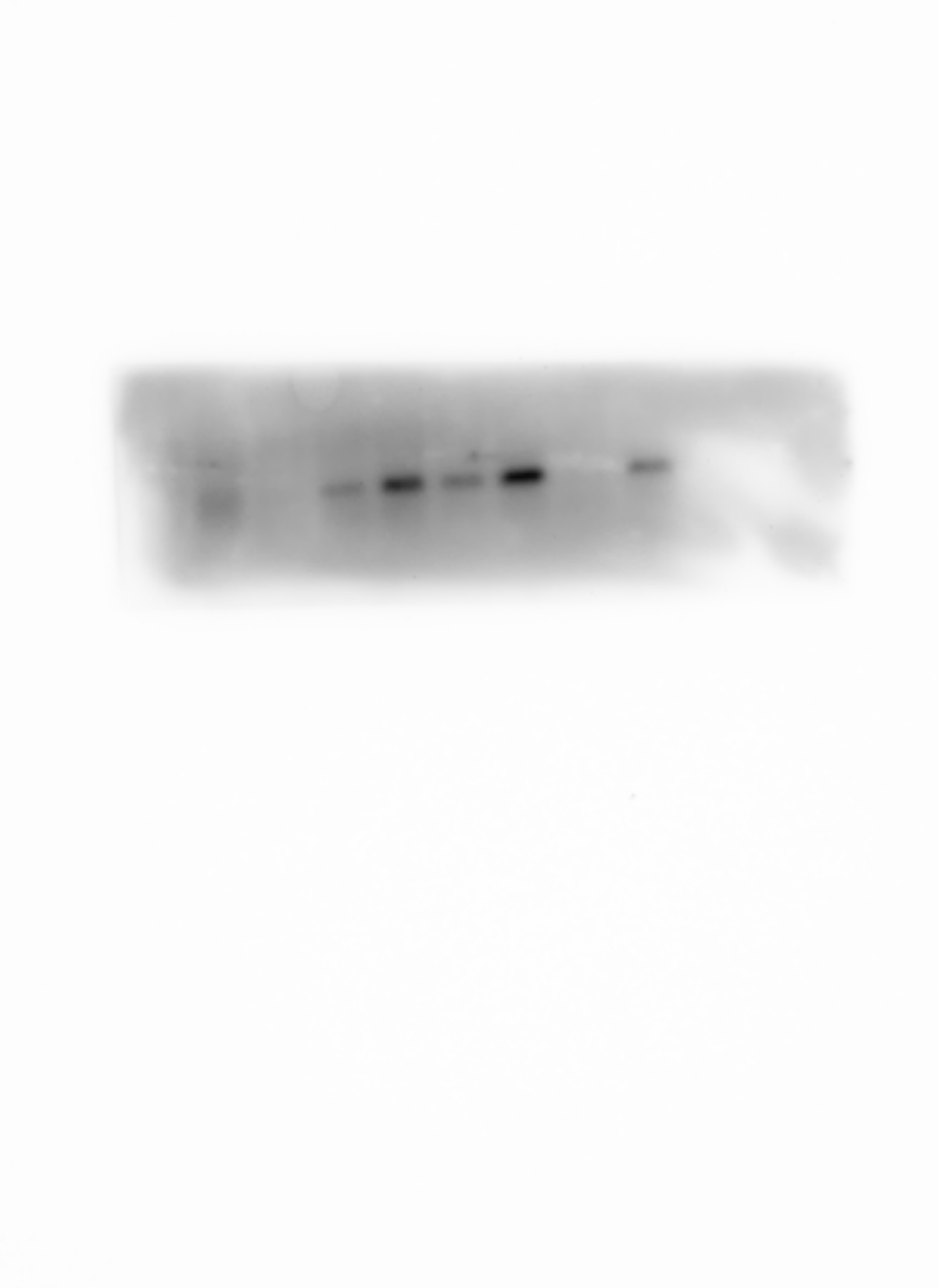

Supplement: Supplementary file 10 — Source Data Fig. 6 [file 44319_2024_64_MOESM10_ESM.zip › 6A/FLAG_CoIP.jpg]

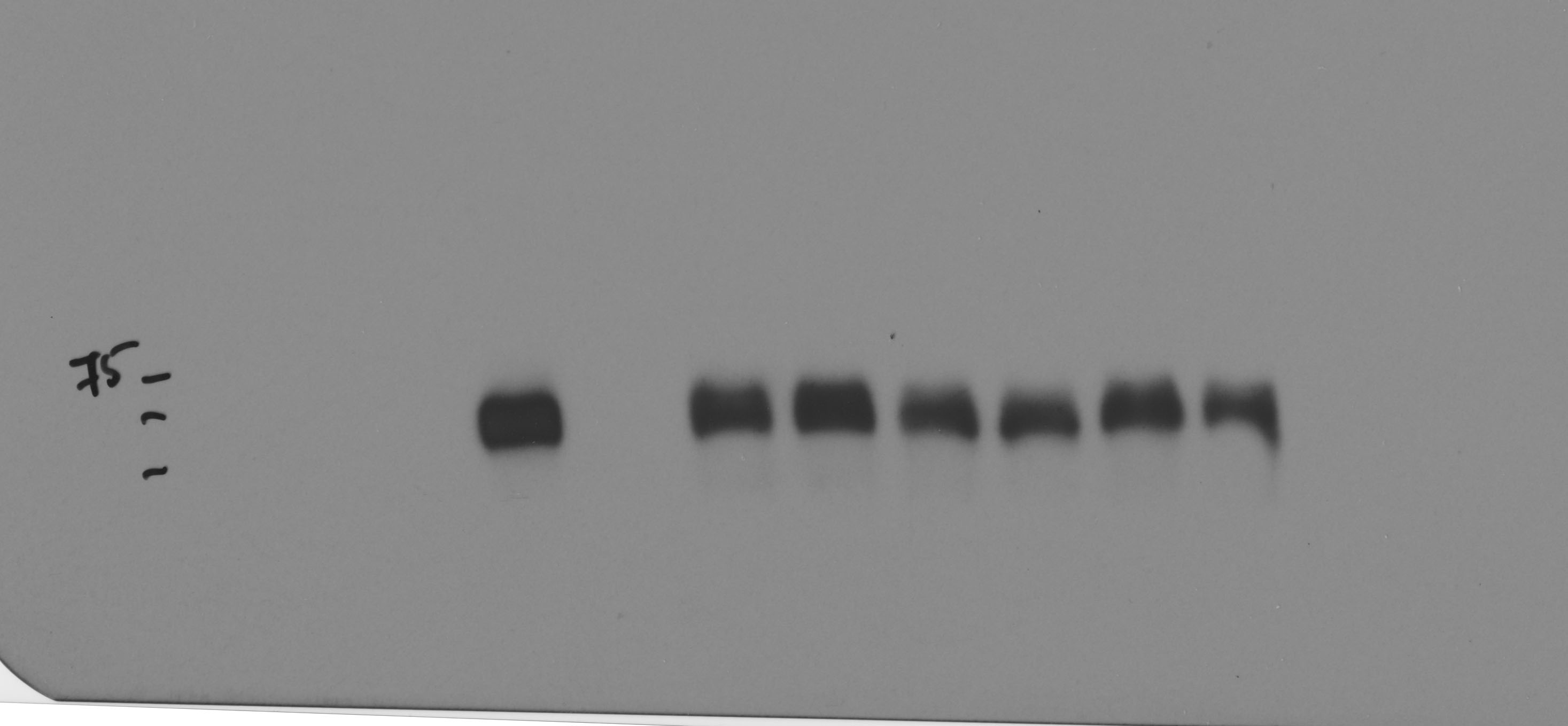

Supplement: Supplementary file 10 — Source Data Fig. 6 [file 44319_2024_64_MOESM10_ESM.zip › 6B/IP p75NTR:IB p75NTR.jpg]

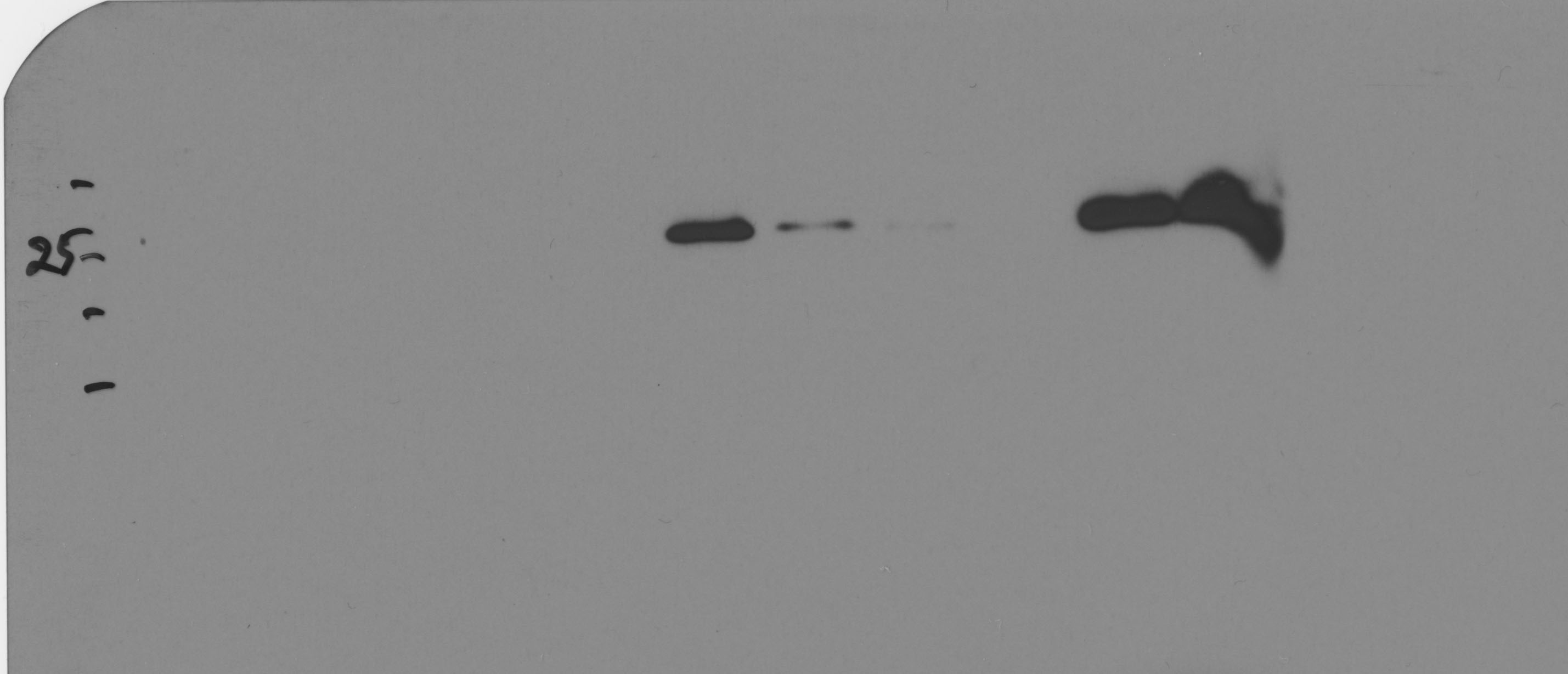

Supplement: Supplementary file 10 — Source Data Fig. 6 [file 44319_2024_64_MOESM10_ESM.zip › 6B/IP p75NTR:IB Flag (RhoGDI).jpg]

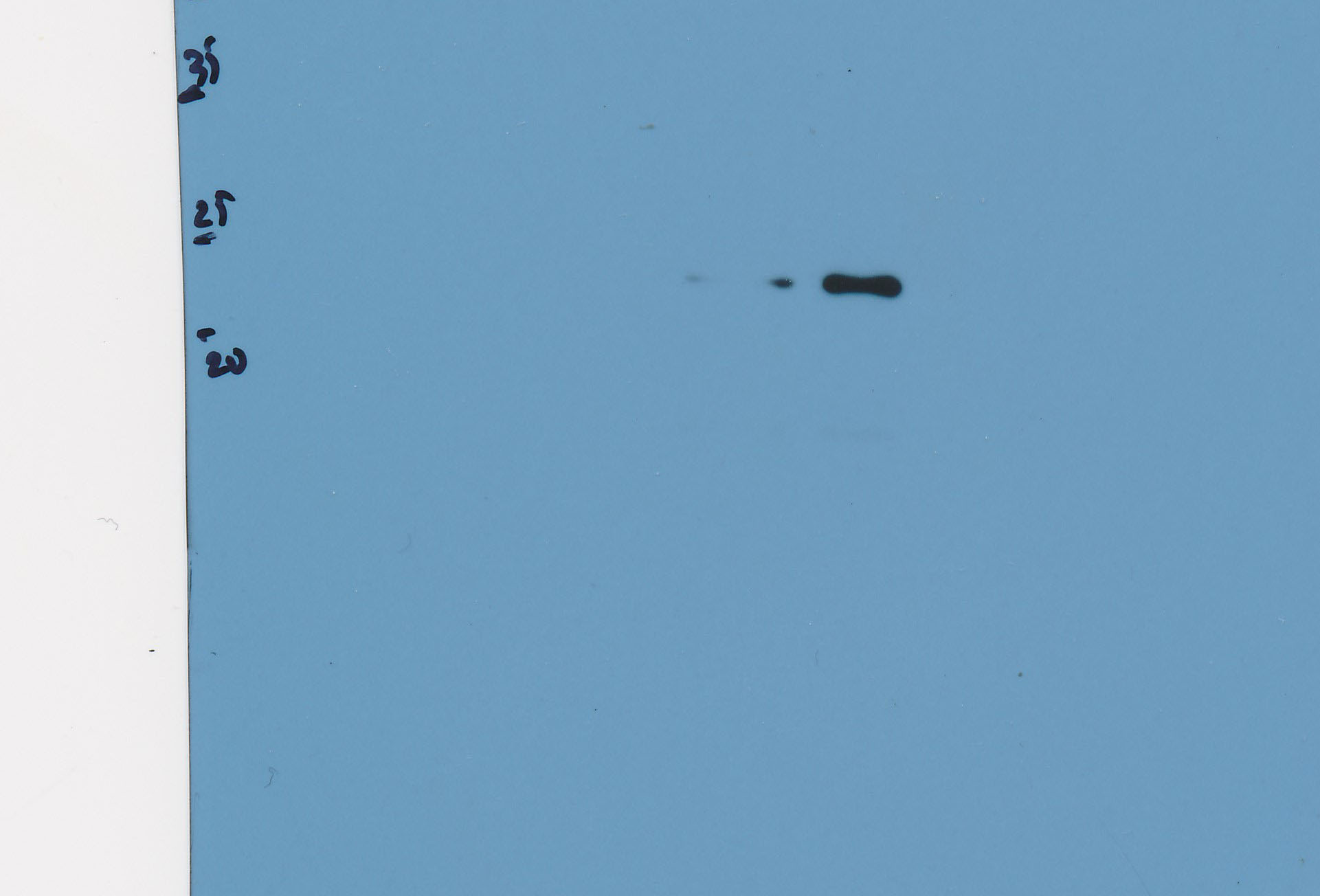

Supplement: Supplementary file 10 — Source Data Fig. 6 [file 44319_2024_64_MOESM10_ESM.zip › 6D/IP RhoGDI:IB P-Ser96 RhoGDI.jpg]

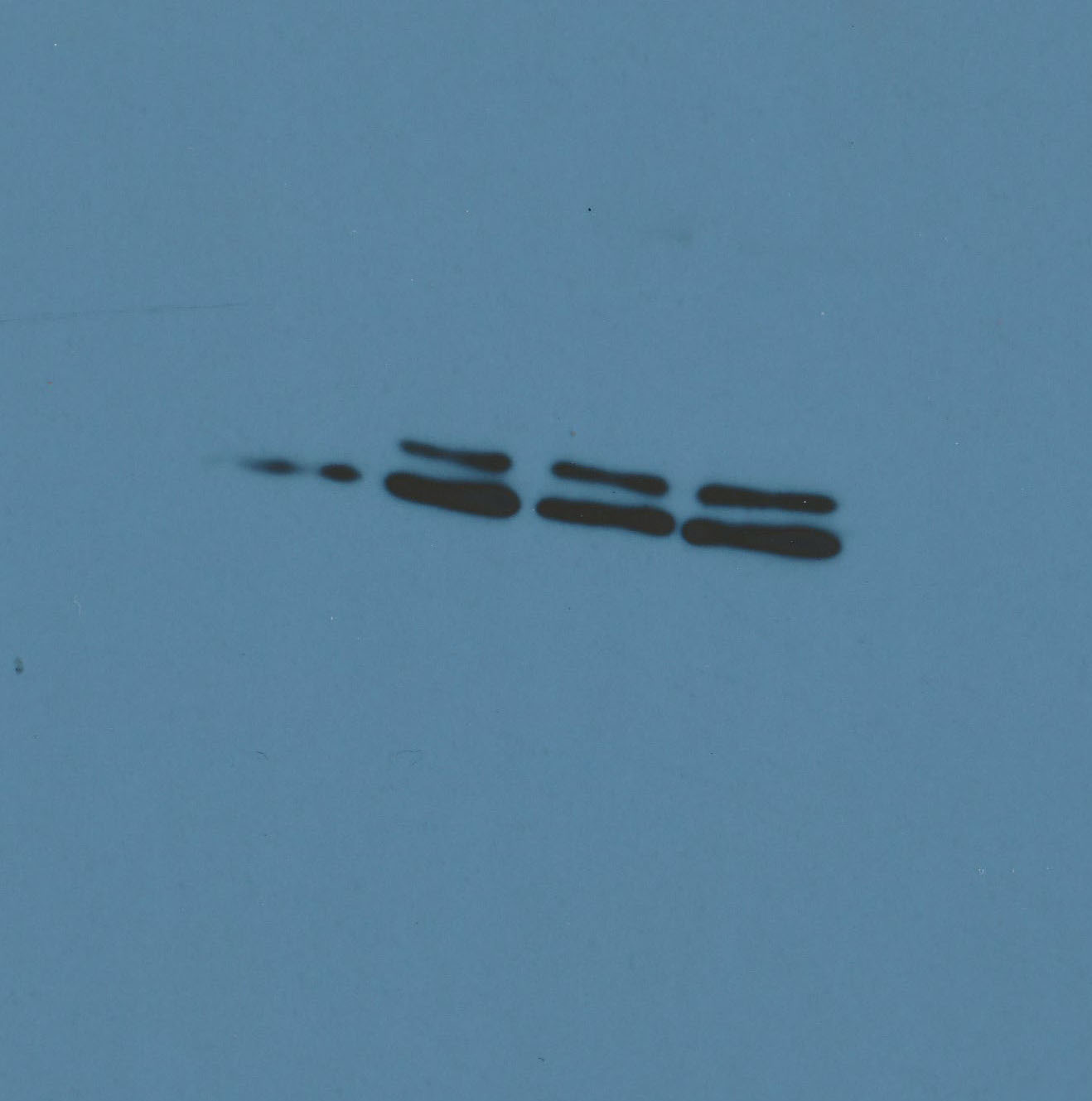

Supplement: Supplementary file 10 — Source Data Fig. 6 [file 44319_2024_64_MOESM10_ESM.zip › 6D/IP RhoGDI:IB RhoGDI.jpg]

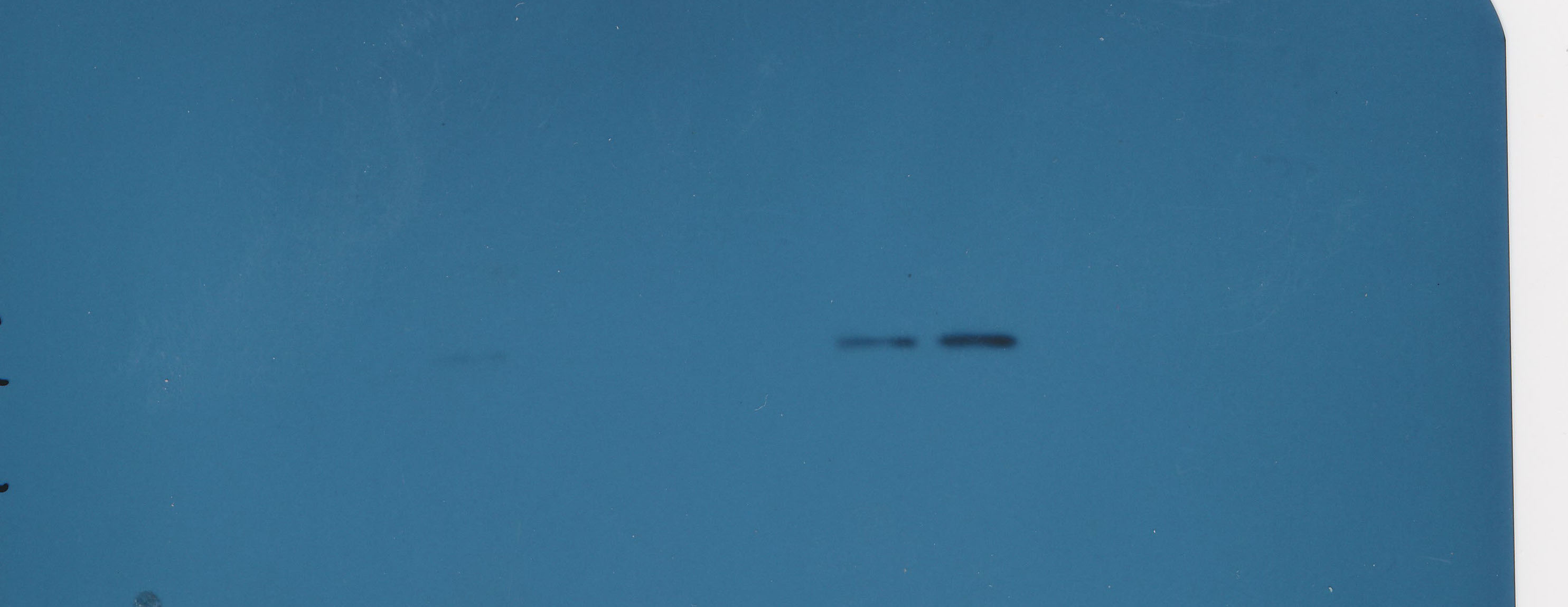

Supplement: Supplementary file 10 — Source Data Fig. 6 [file 44319_2024_64_MOESM10_ESM.zip › 6E/IP Flag (RhoGDI):IB P-Ser96 RhoGDI.jpg]

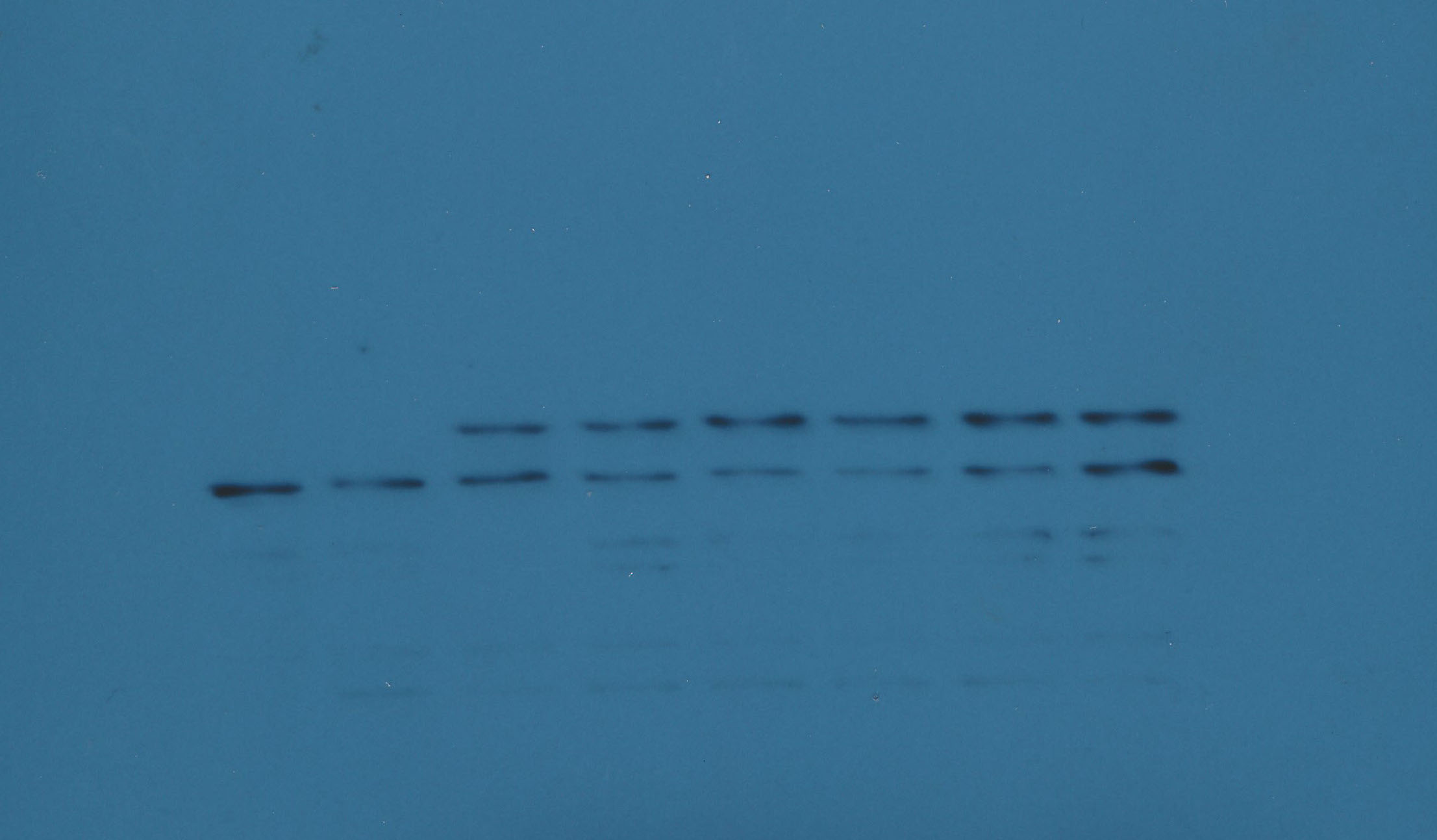

Supplement: Supplementary file 10 — Source Data Fig. 6 [file 44319_2024_64_MOESM10_ESM.zip › 6E/IP RhoGDI:IB RhoGDI.jpg]

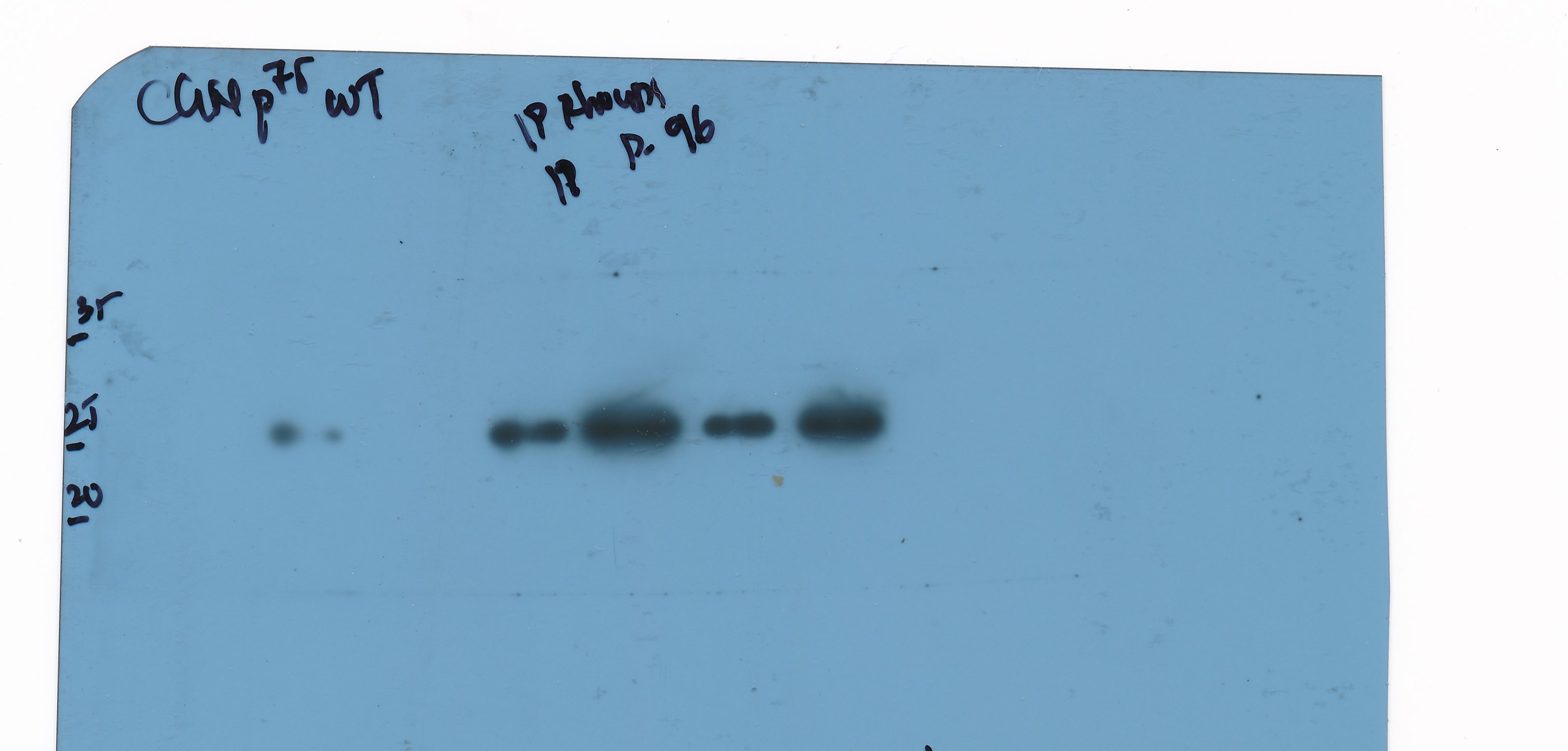

Supplement: Supplementary file 10 — Source Data Fig. 6 [file 44319_2024_64_MOESM10_ESM.zip › 6F/IP RhoGDI:IB p-Ser 96.jpg]

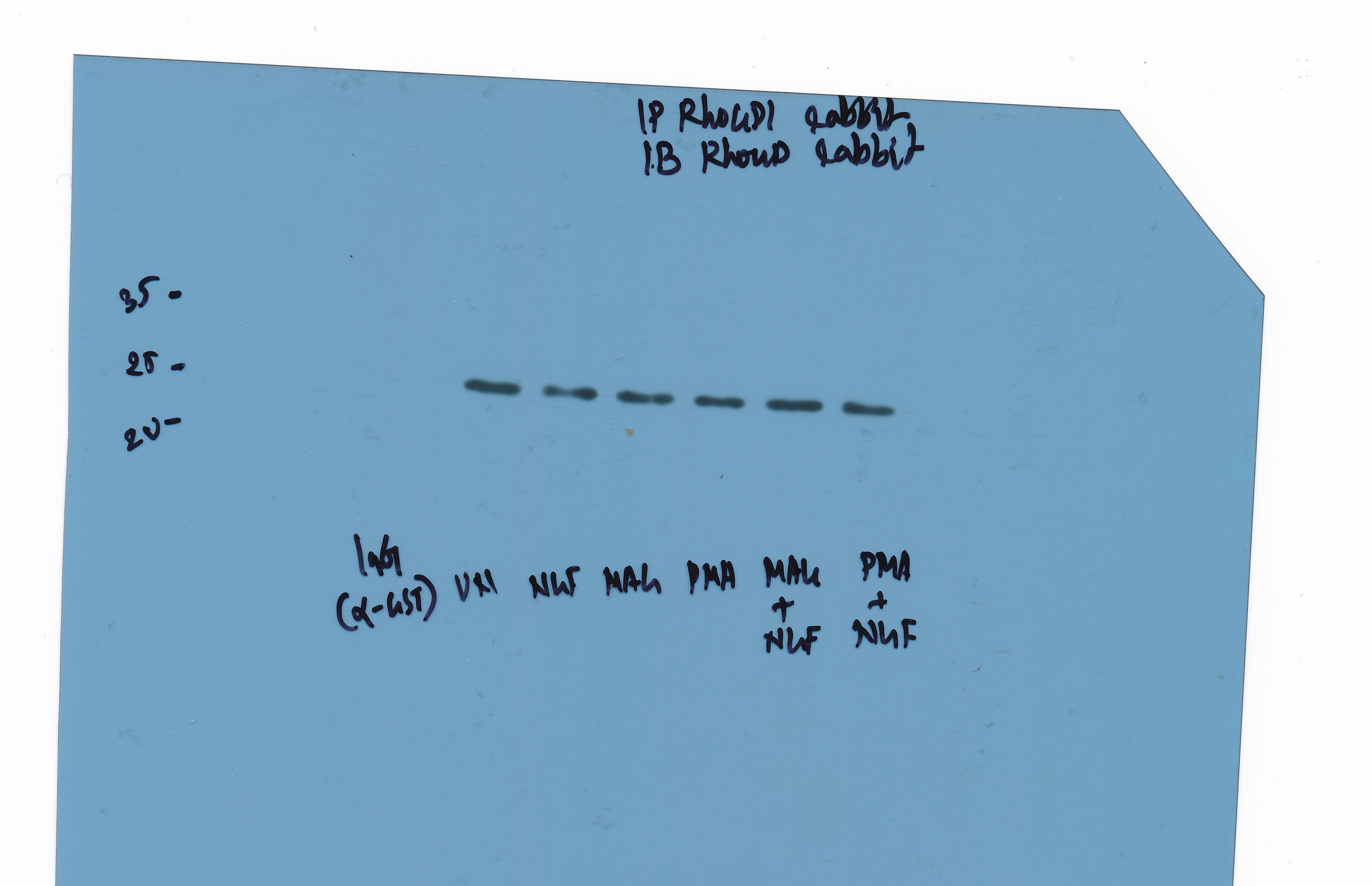

Supplement: Supplementary file 10 — Source Data Fig. 6 [file 44319_2024_64_MOESM10_ESM.zip › 6F/IP RhoGDI:IB RhoGDI.jpg]

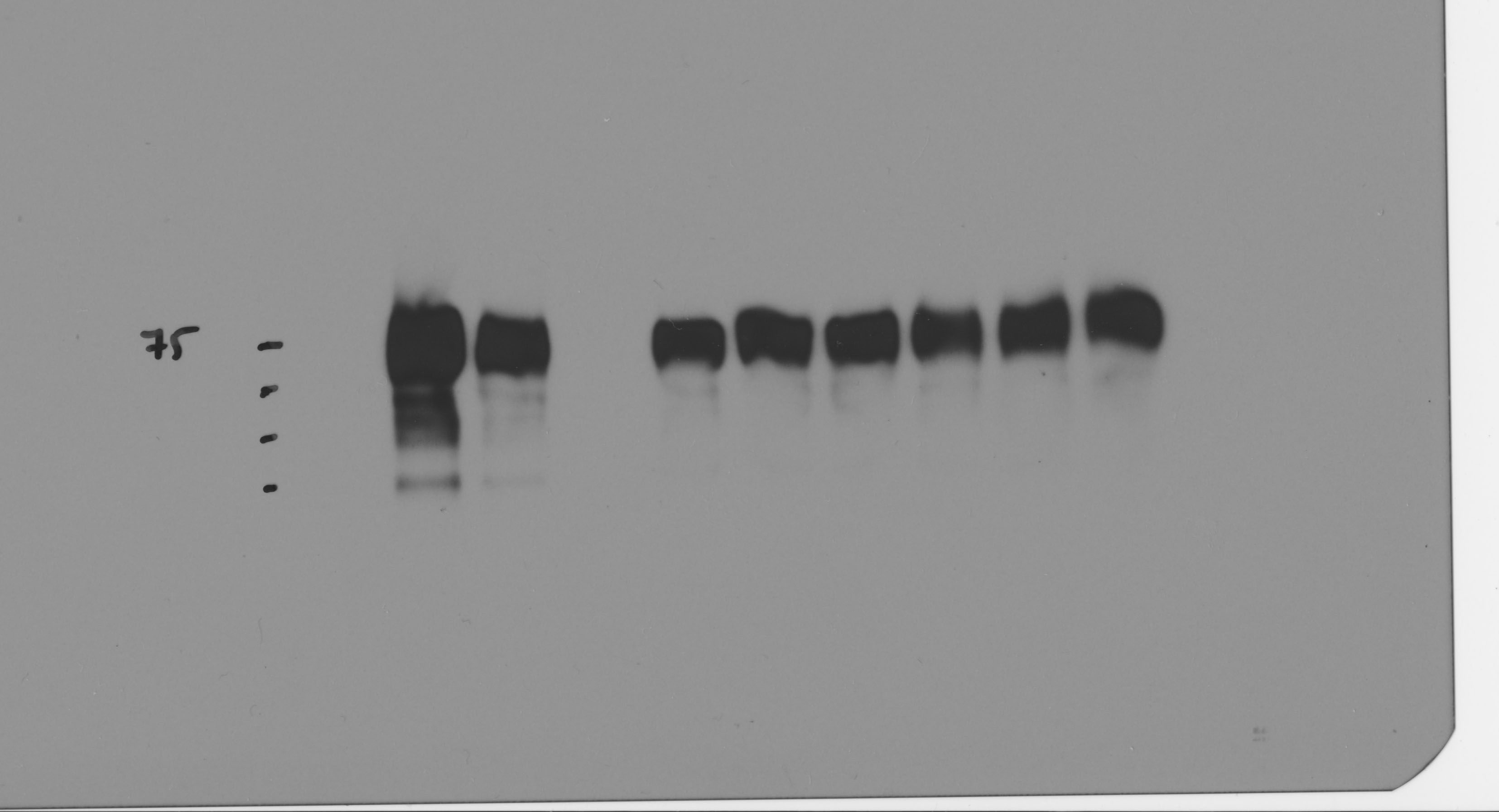

Supplement: Supplementary file 10 — Source Data Fig. 6 [file 44319_2024_64_MOESM10_ESM.zip › 6G/IP p75NTR:IB p75NTR.jpg]

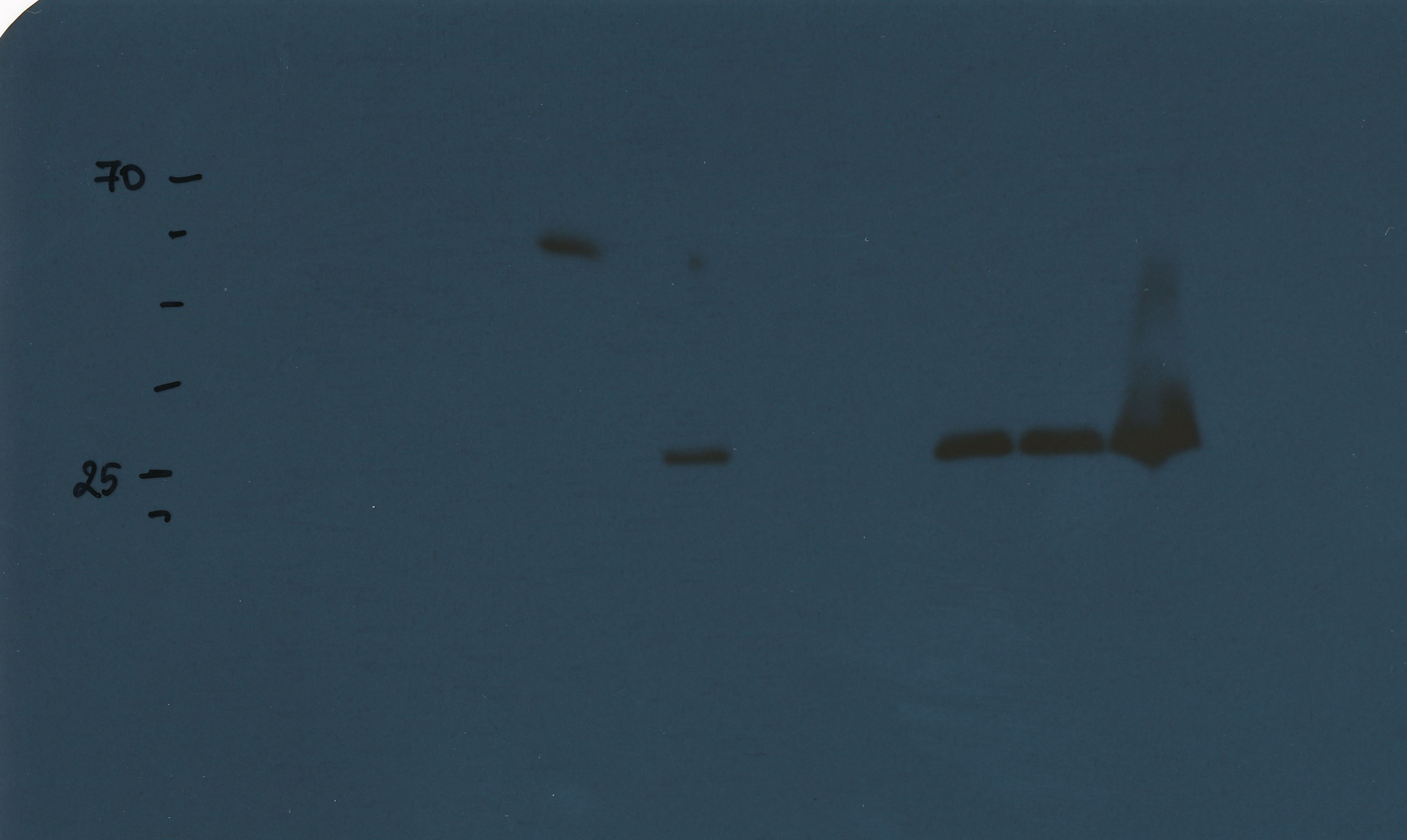

Supplement: Supplementary file 10 — Source Data Fig. 6 [file 44319_2024_64_MOESM10_ESM.zip › 6G/IP p75NTR:IB Flag (RhoGDI).jpg]

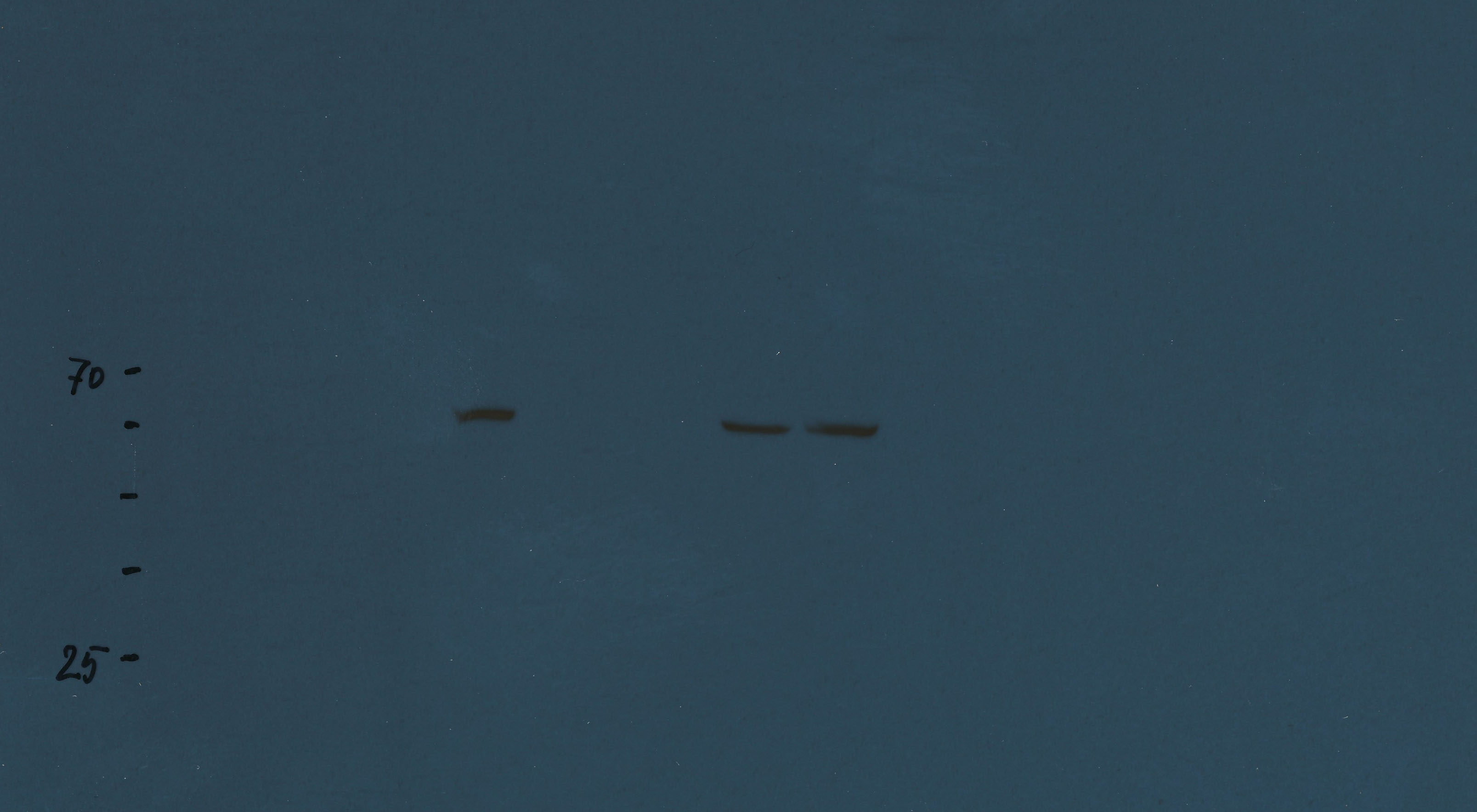

Supplement: Supplementary file 10 — Source Data Fig. 6 [file 44319_2024_64_MOESM10_ESM.zip › 6G/IP p75NTR:IB Myc (RIP2).jpg]

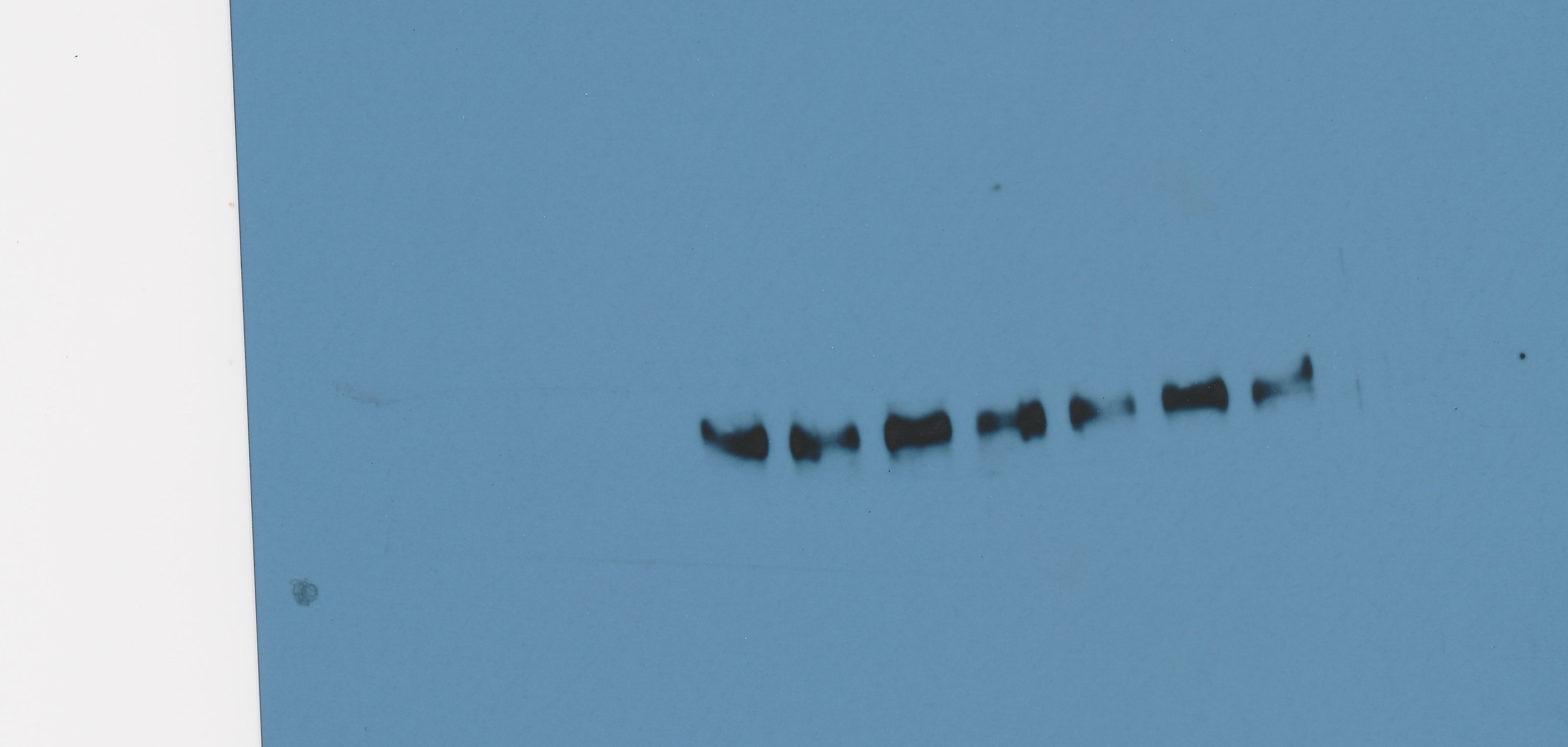

Supplement: Supplementary file 10 — Source Data Fig. 6 [file 44319_2024_64_MOESM10_ESM.zip › 6H/IP p75NTR:IB p75NTR.jpg]

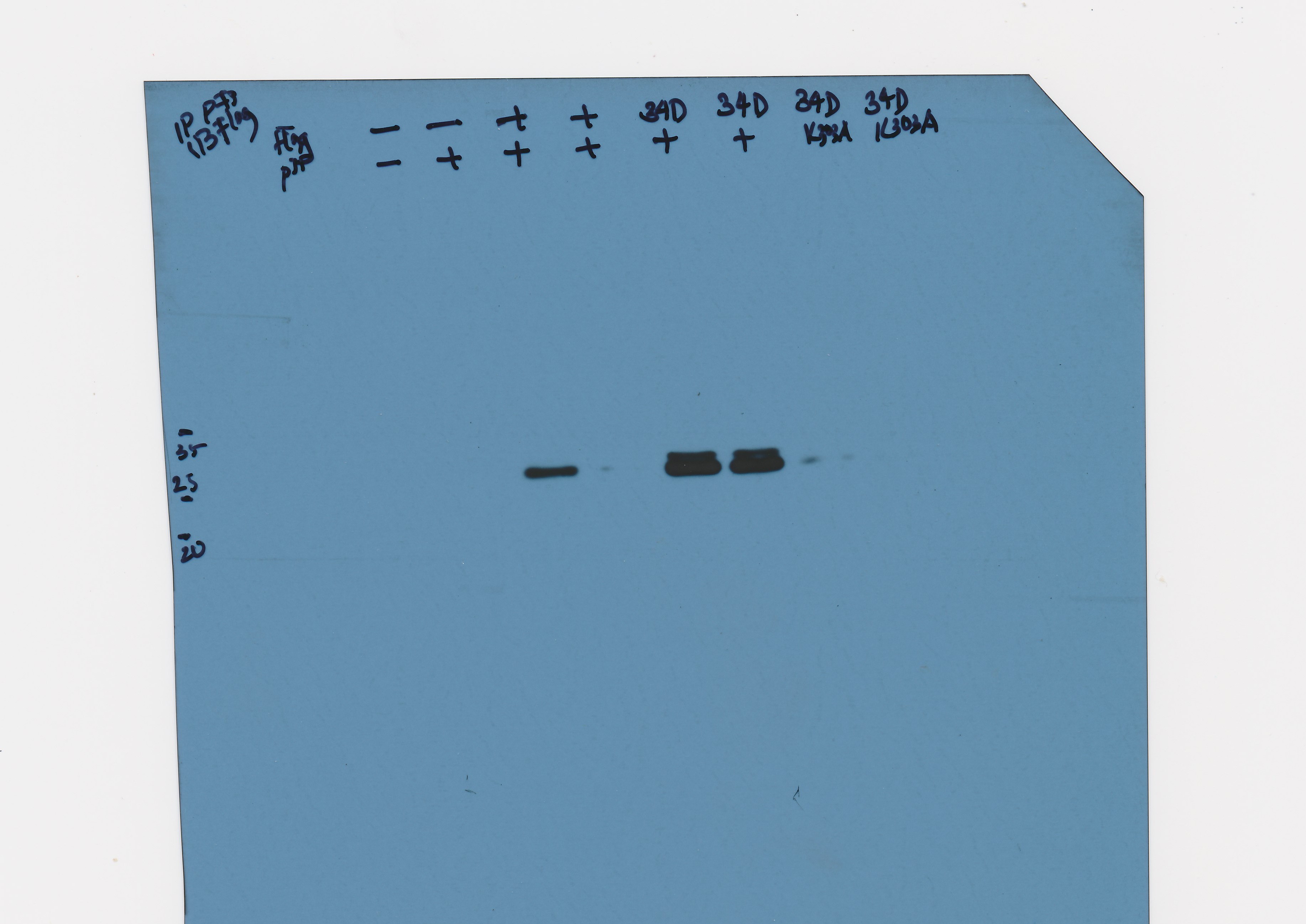

Supplement: Supplementary file 10 — Source Data Fig. 6 [file 44319_2024_64_MOESM10_ESM.zip › 6H/IP p75NTR:IB Flag (RhoGDI).jpg]

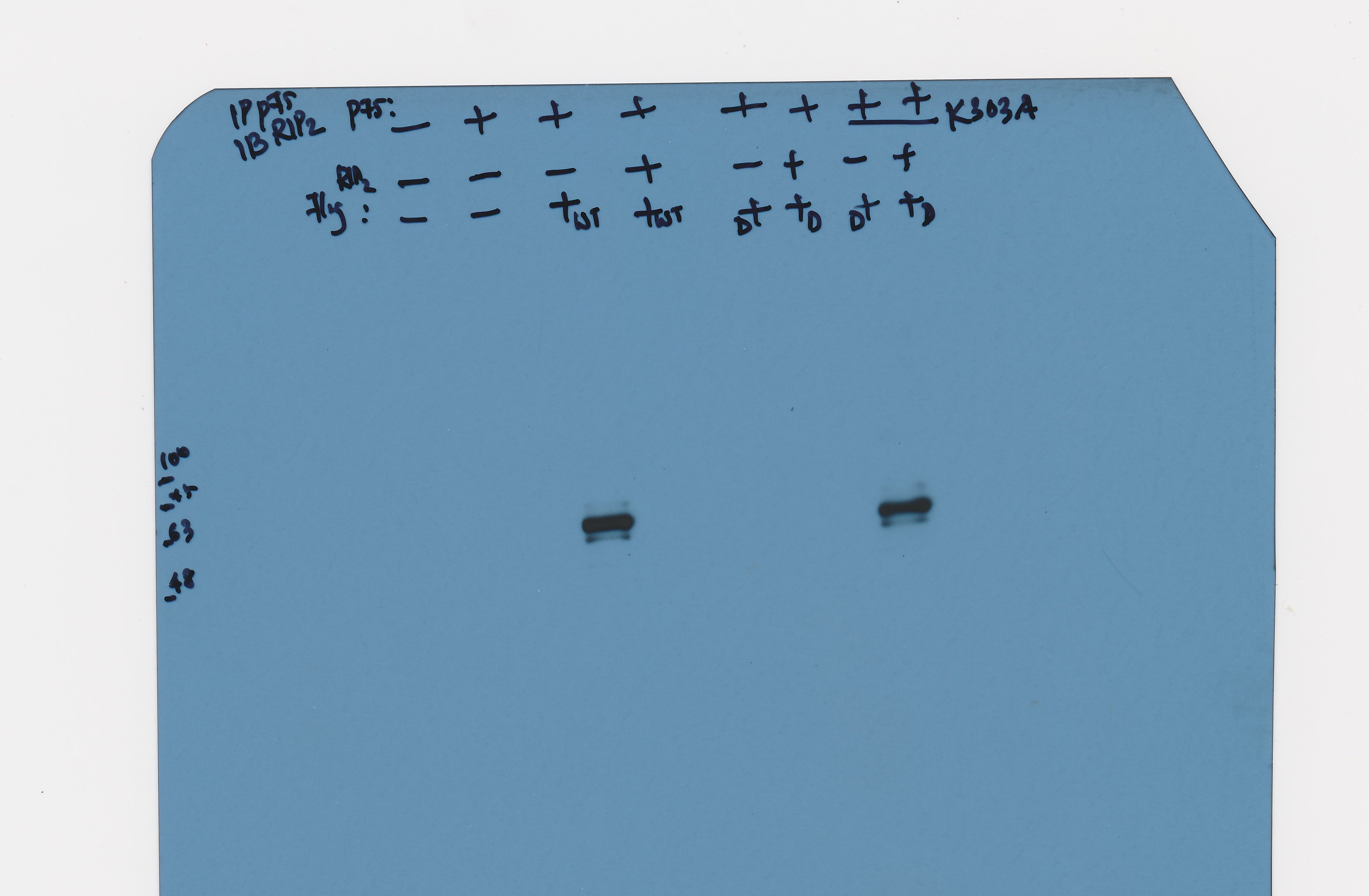

Supplement: Supplementary file 10 — Source Data Fig. 6 [file 44319_2024_64_MOESM10_ESM.zip › 6H/IP p75NTR:IB Myc (RIP2).jpg]

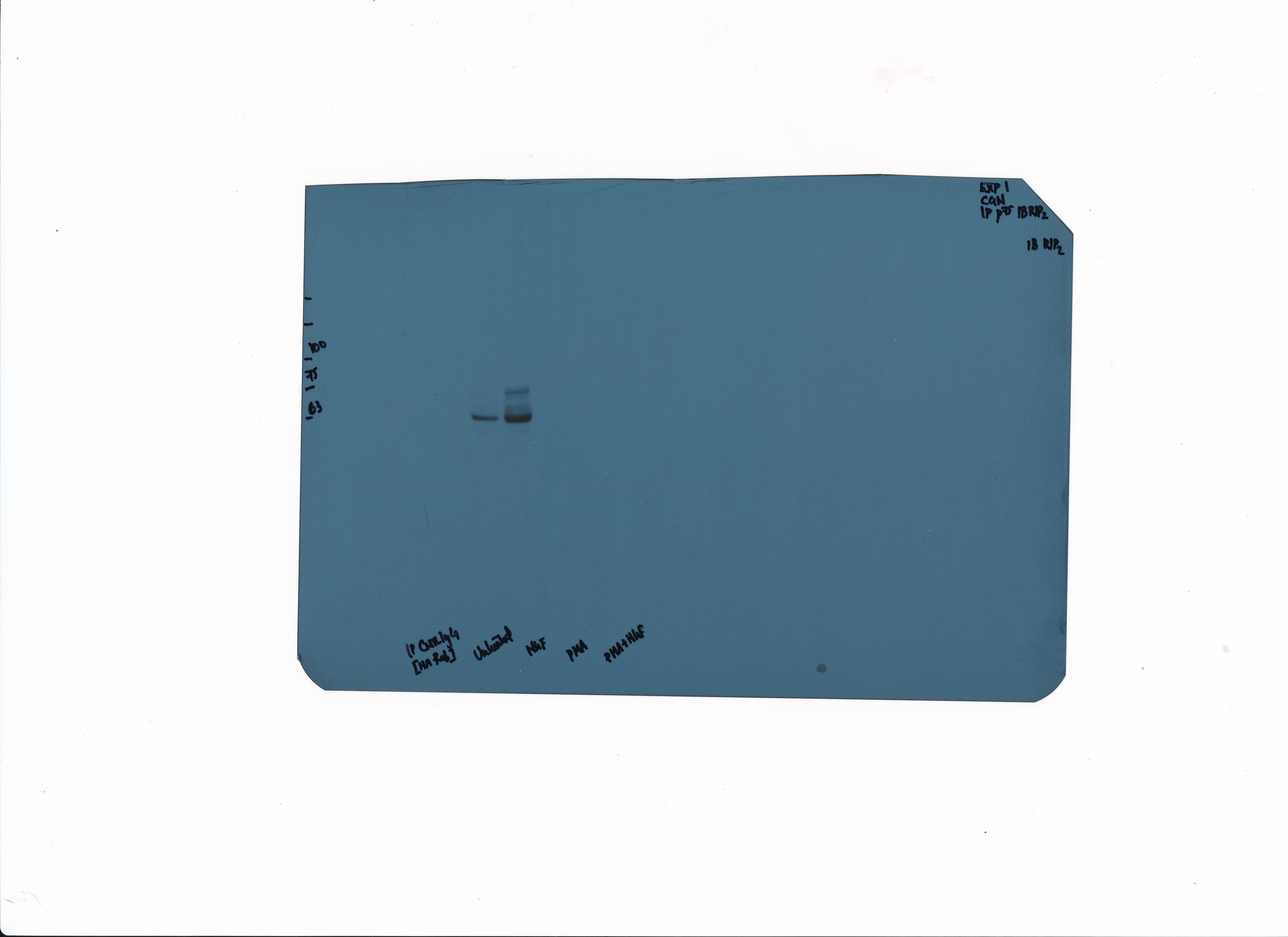

Supplement: Supplementary file 10 — Source Data Fig. 6 [file 44319_2024_64_MOESM10_ESM.zip › 6J/IP p75NTR:IB RIP2.jpg]

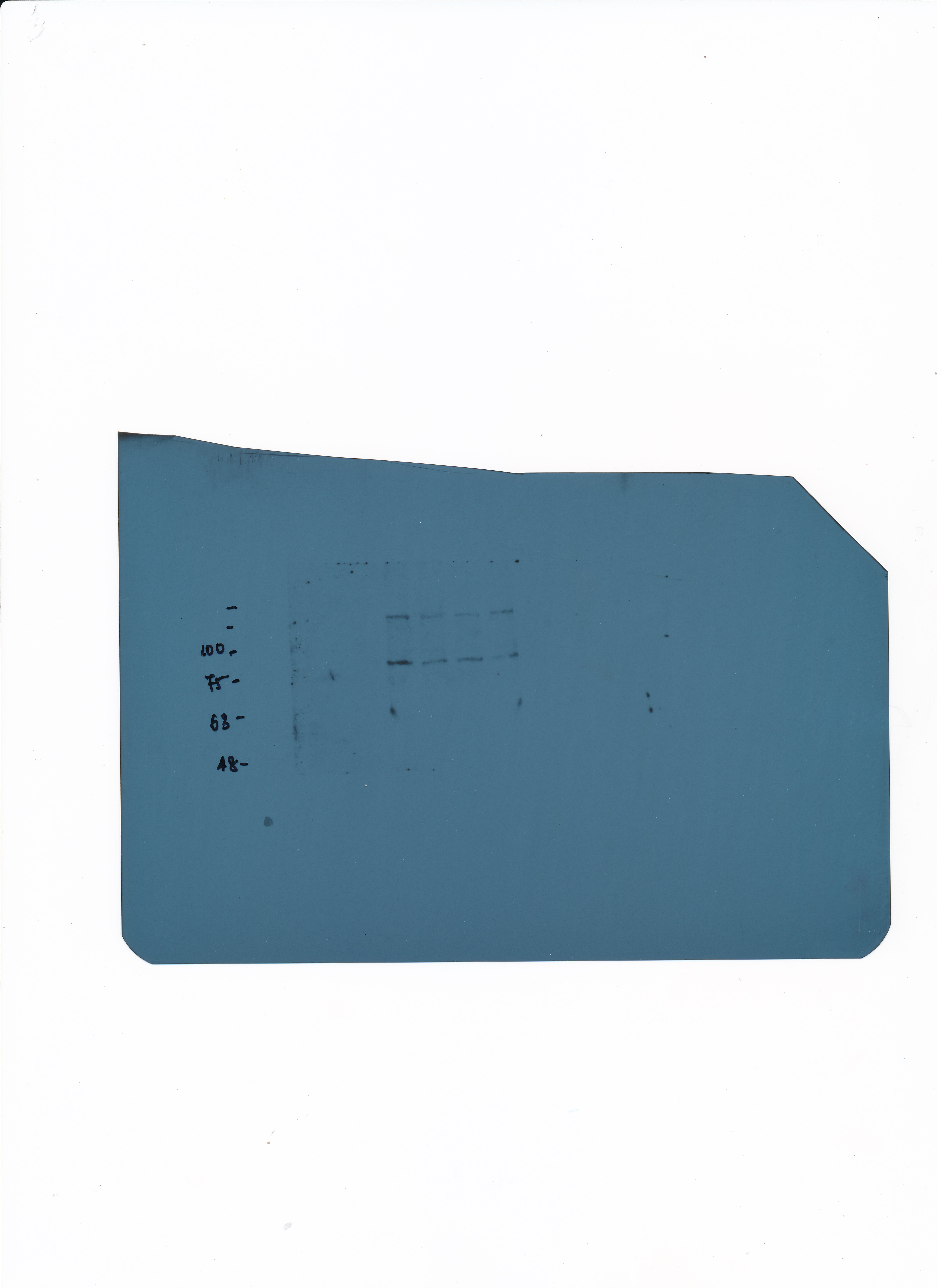

Supplement: Supplementary file 10 — Source Data Fig. 6 [file 44319_2024_64_MOESM10_ESM.zip › 6J/IP P75NTR:IB P75NTR.jpg]

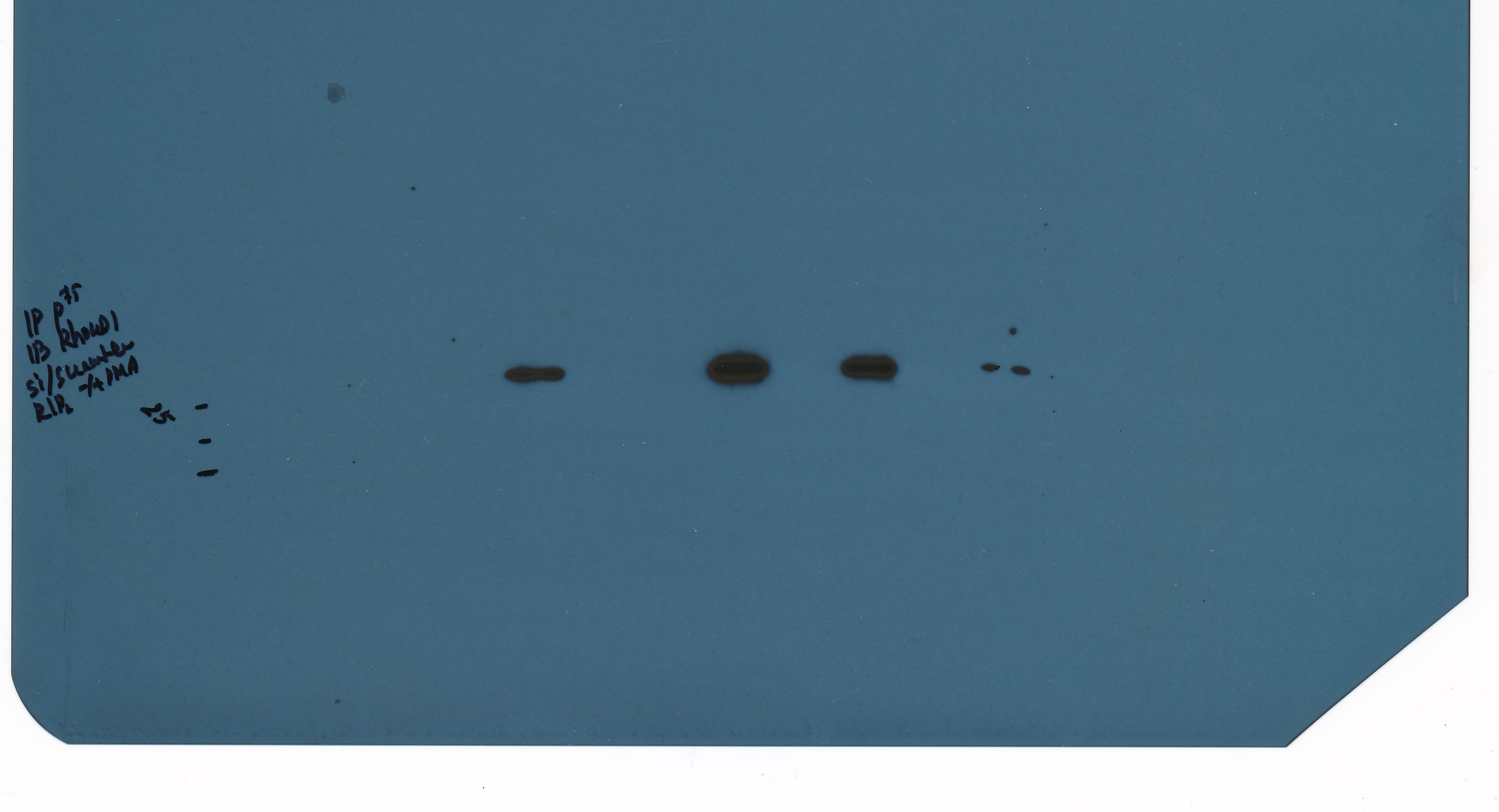

Supplement: Supplementary file 10 — Source Data Fig. 6 [file 44319_2024_64_MOESM10_ESM.zip › 6K/IP p75NTR:IB RhoGDI.jpg]

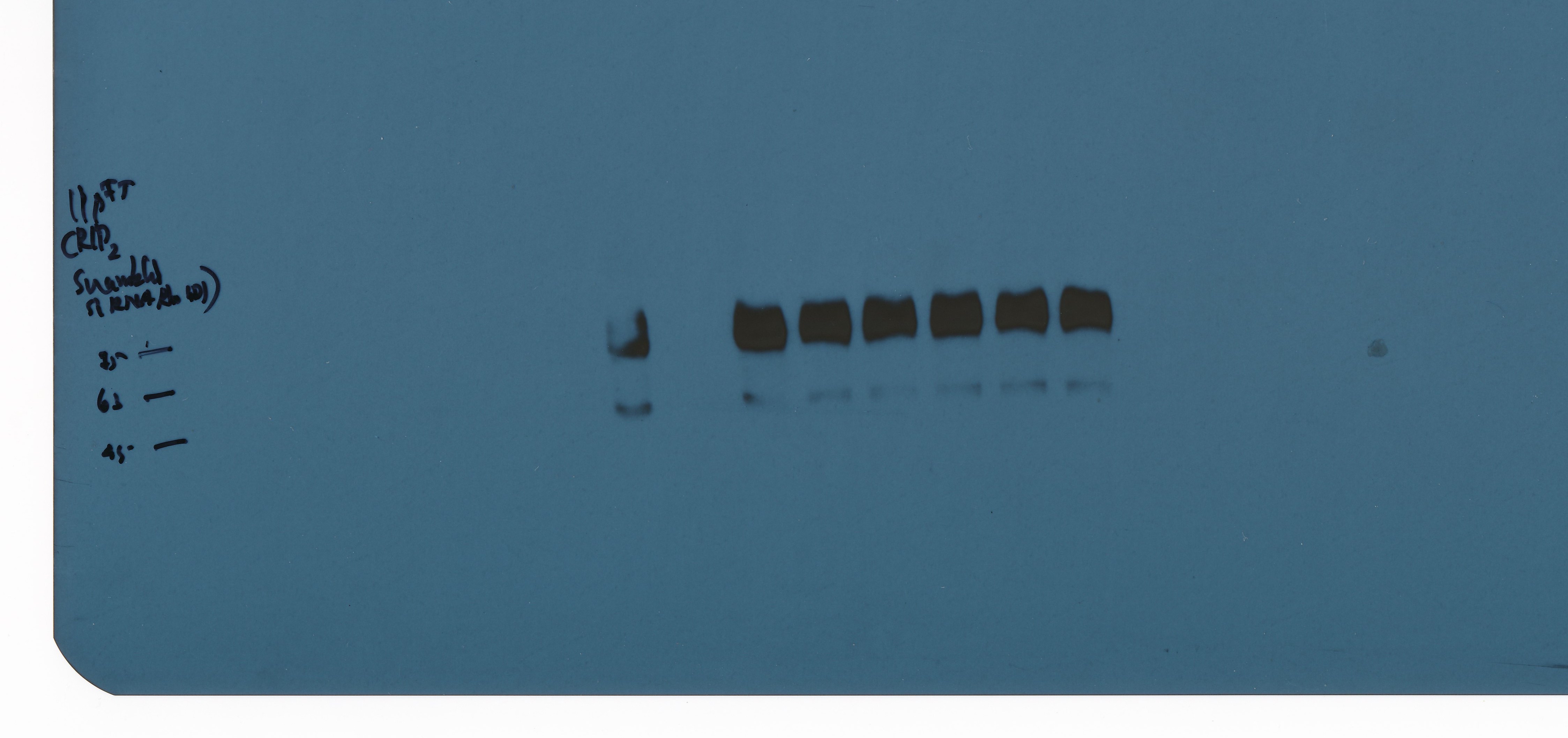

Supplement: Supplementary file 10 — Source Data Fig. 6 [file 44319_2024_64_MOESM10_ESM.zip › 6K/IP p75NTR:IB p75NTR.jpg]

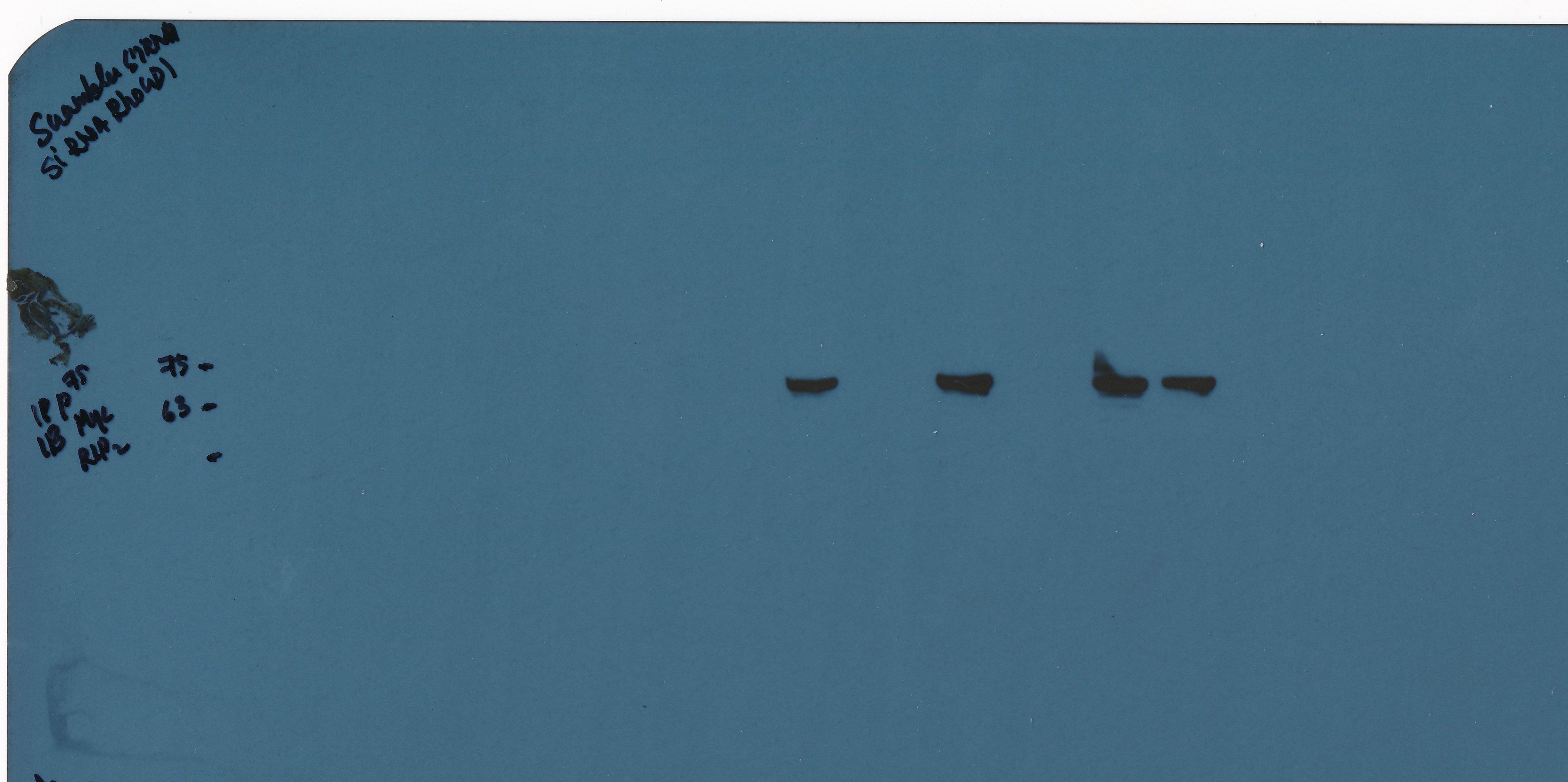

Supplement: Supplementary file 10 — Source Data Fig. 6 [file 44319_2024_64_MOESM10_ESM.zip › 6K/IP p75NTR:IB Myc (RIP2).jpg]

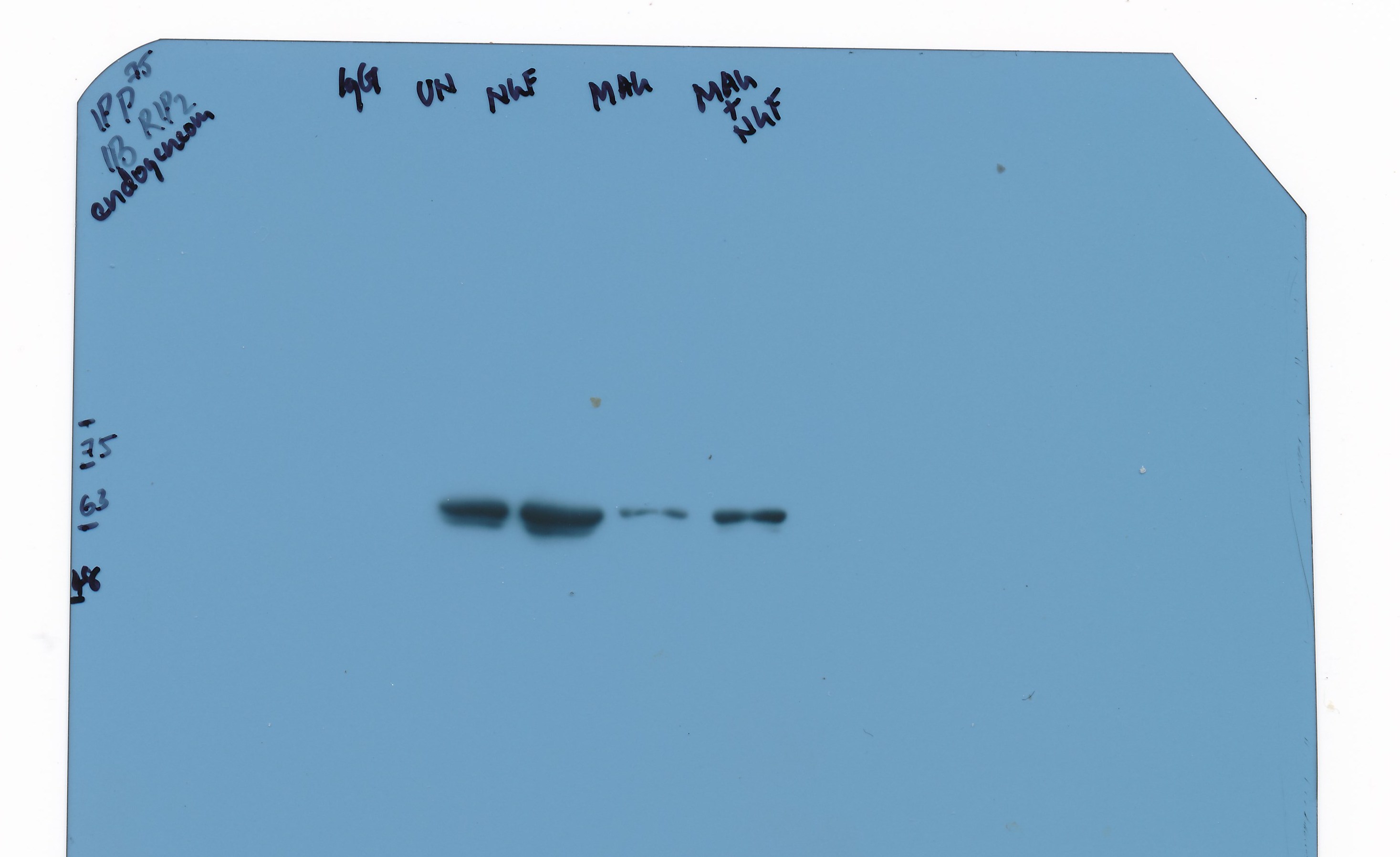

Supplement: Supplementary file 10 — Source Data Fig. 6 [file 44319_2024_64_MOESM10_ESM.zip › 6L/IP p75NTR:IB RIP2.jpg]

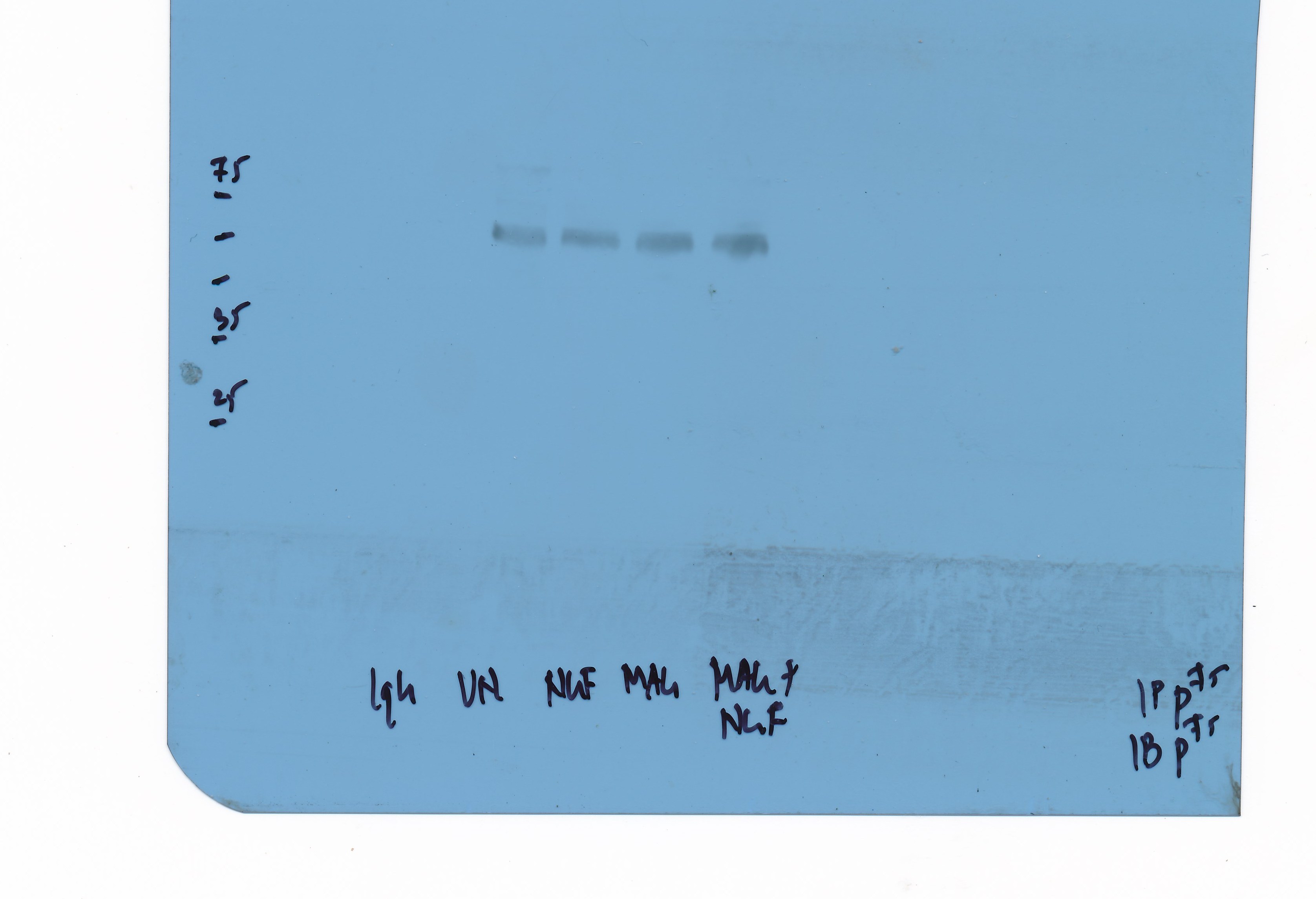

Supplement: Supplementary file 10 — Source Data Fig. 6 [file 44319_2024_64_MOESM10_ESM.zip › 6L/IP p75NTR:IB p75NTR.jpg]

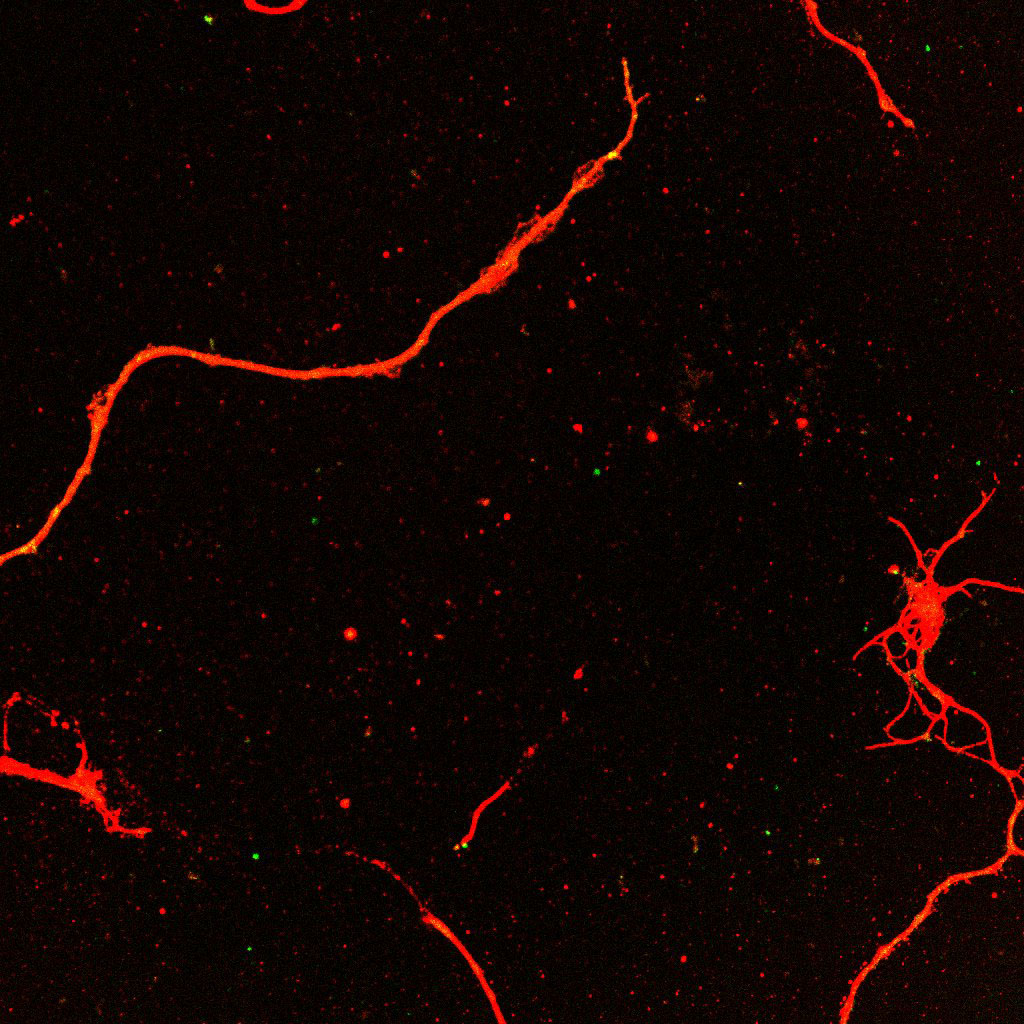

Supplement: Supplementary file 11 — Source Data Fig. 7 [file 44319_2024_64_MOESM11_ESM.zip › 7A/7A RhoGDI S34D and p75NTR WT.jpg]

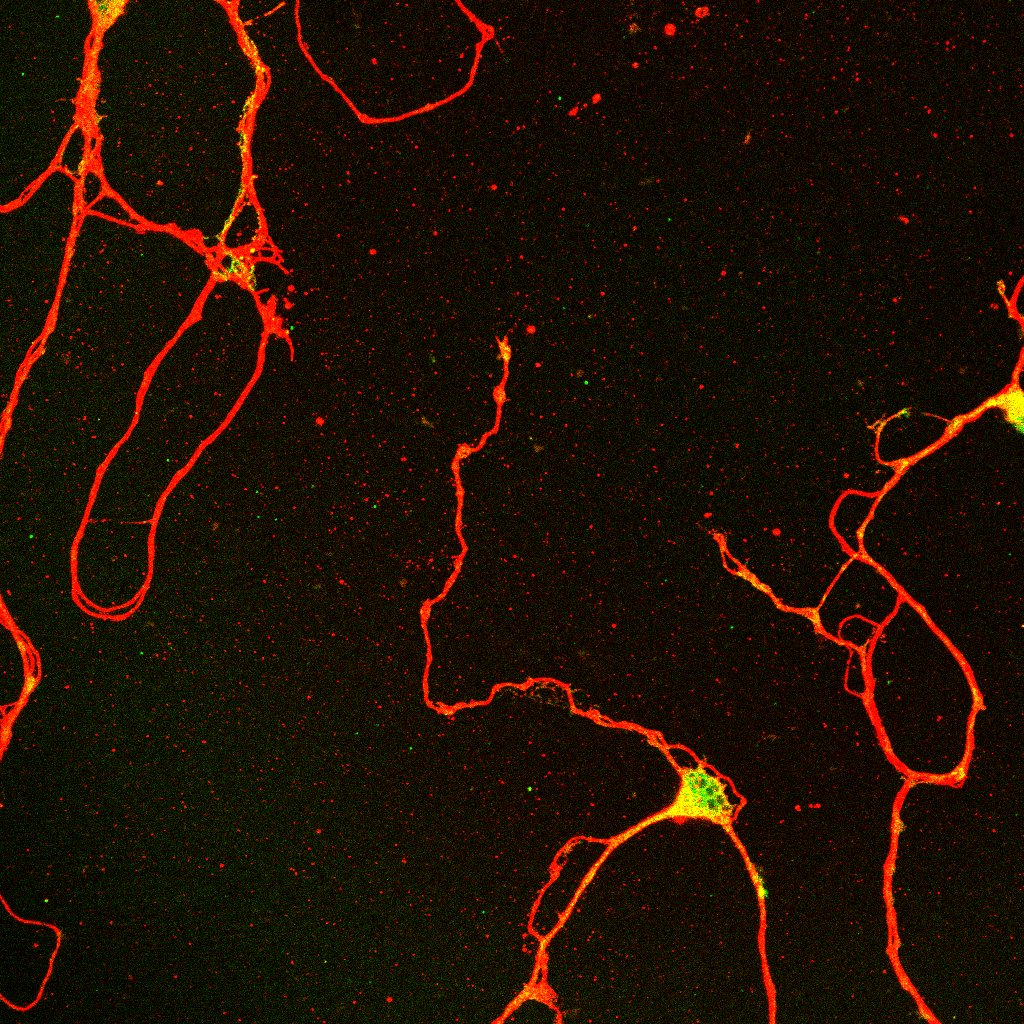

Supplement: Supplementary file 11 — Source Data Fig. 7 [file 44319_2024_64_MOESM11_ESM.zip › 7A/7A RhoGDI S34D and p75NTR KO.jpg]

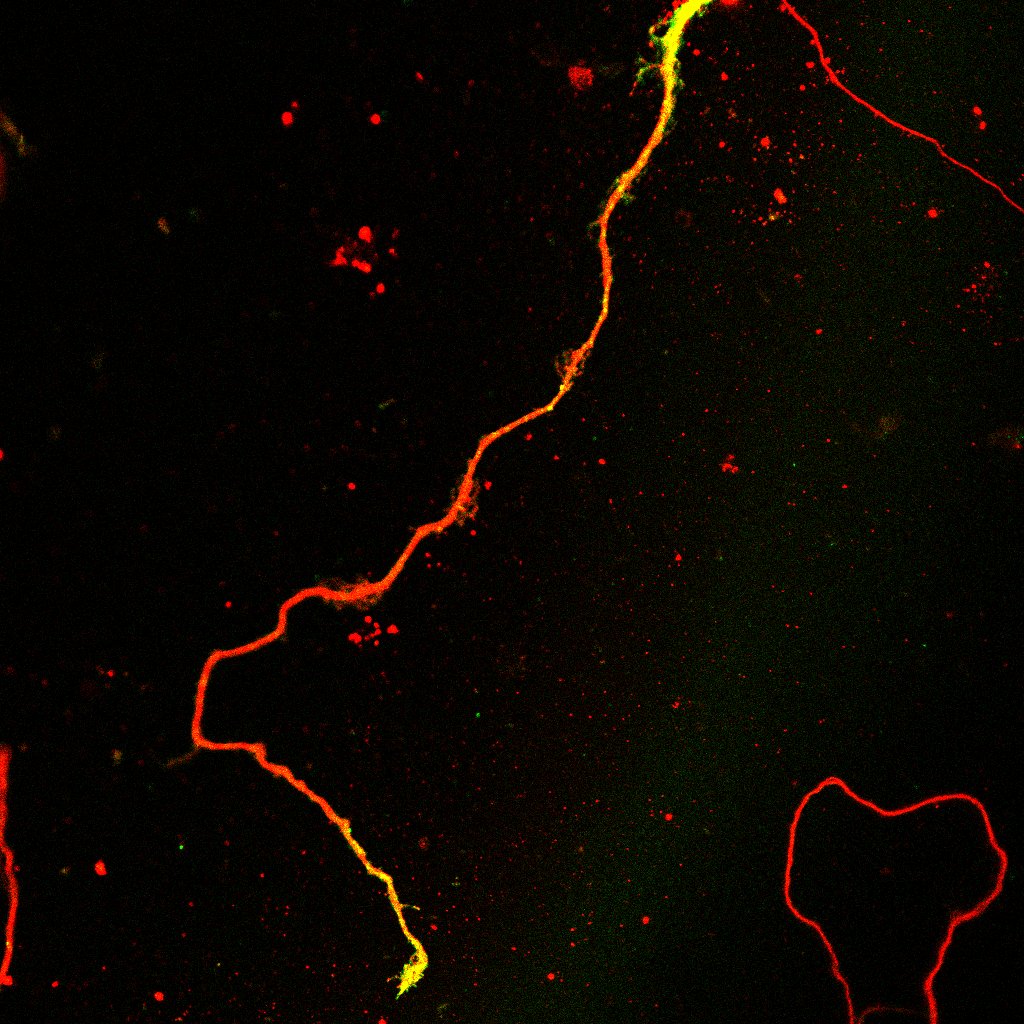

Supplement: Supplementary file 11 — Source Data Fig. 7 [file 44319_2024_64_MOESM11_ESM.zip › 7A/7A RhoGDI WT and p75NTR KO.jpg]

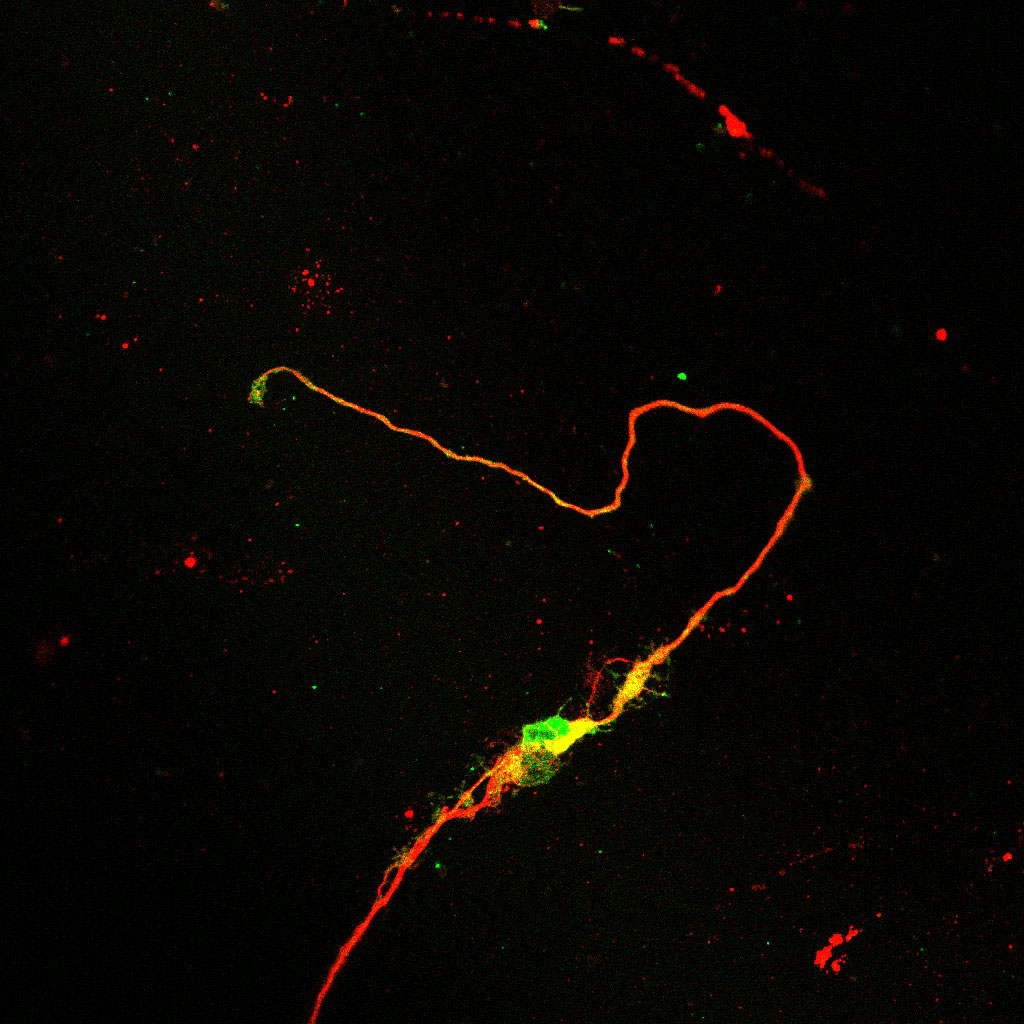

Supplement: Supplementary file 11 — Source Data Fig. 7 [file 44319_2024_64_MOESM11_ESM.zip › 7A/7A RhoGDI WT and p75NTR WT.jpg]

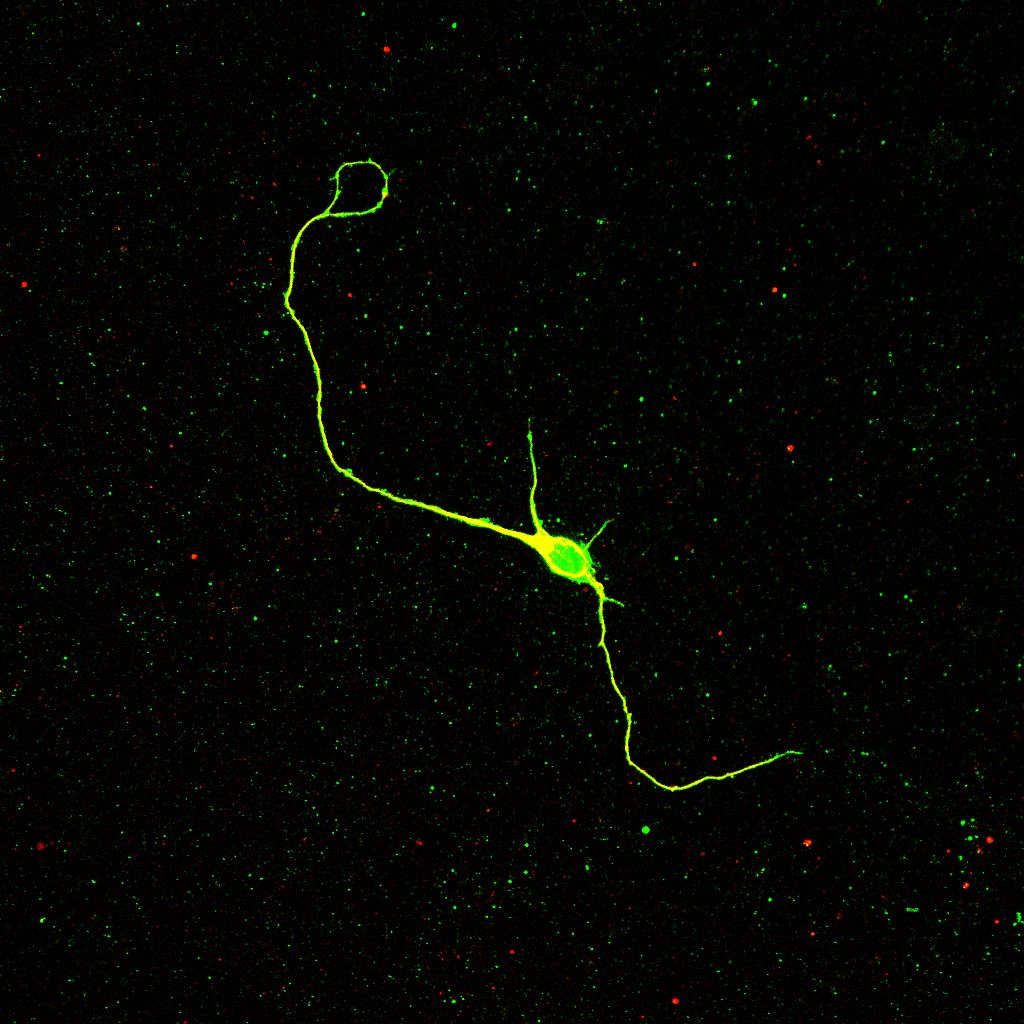

Supplement: Supplementary file 11 — Source Data Fig. 7 [file 44319_2024_64_MOESM11_ESM.zip › 7B/7B - P75NTR WT.jpg]

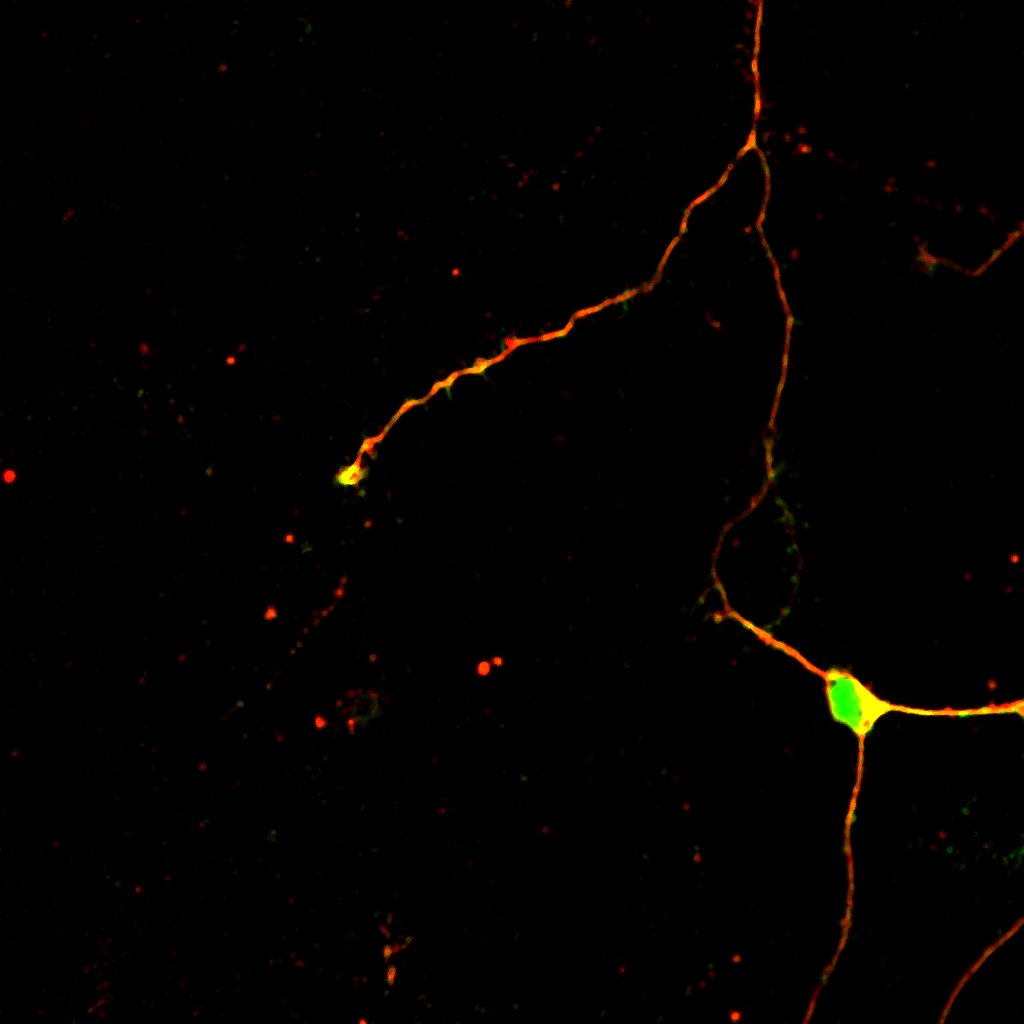

Supplement: Supplementary file 11 — Source Data Fig. 7 [file 44319_2024_64_MOESM11_ESM.zip › 7B/7B - K3030A.jpg]

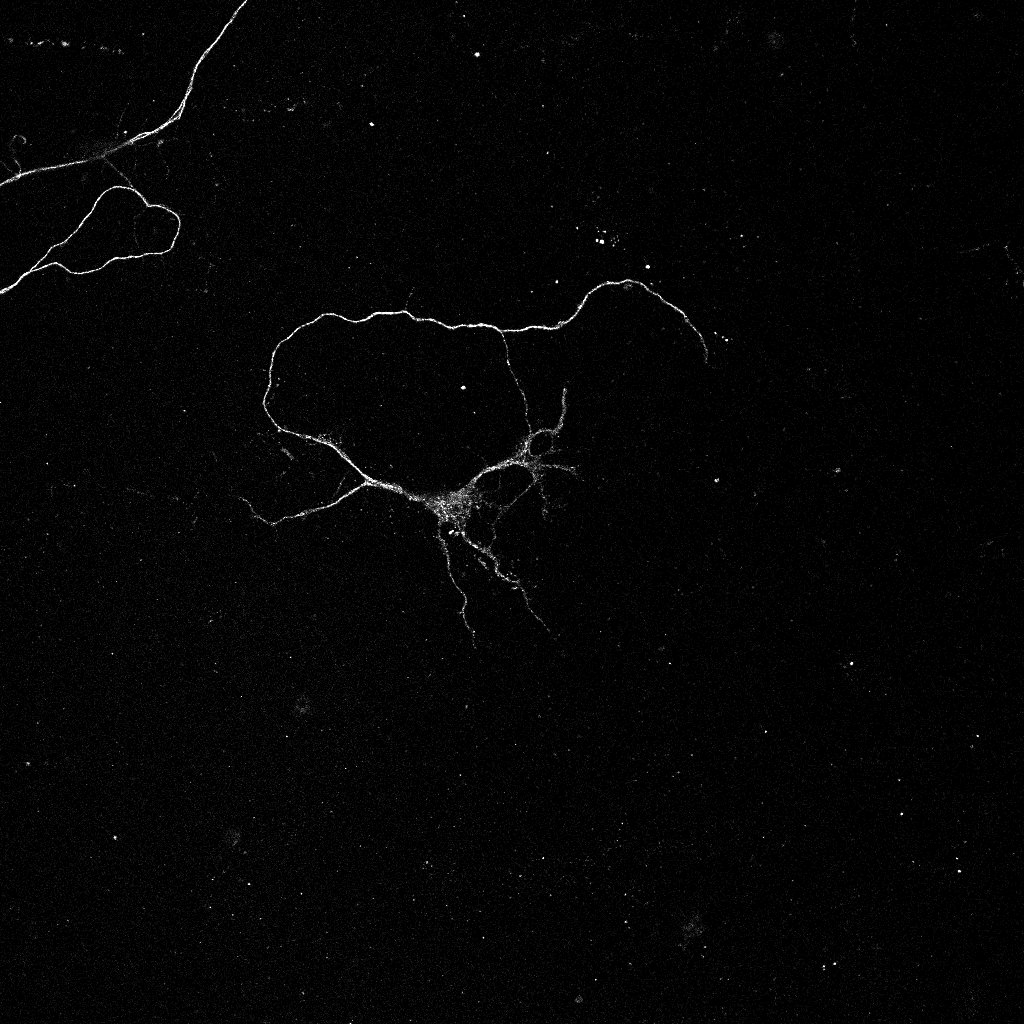

Supplement: Supplementary file 11 — Source Data Fig. 7 [file 44319_2024_64_MOESM11_ESM.zip › 7C/RhoGDI S34D: p75NTR KO.jpg]

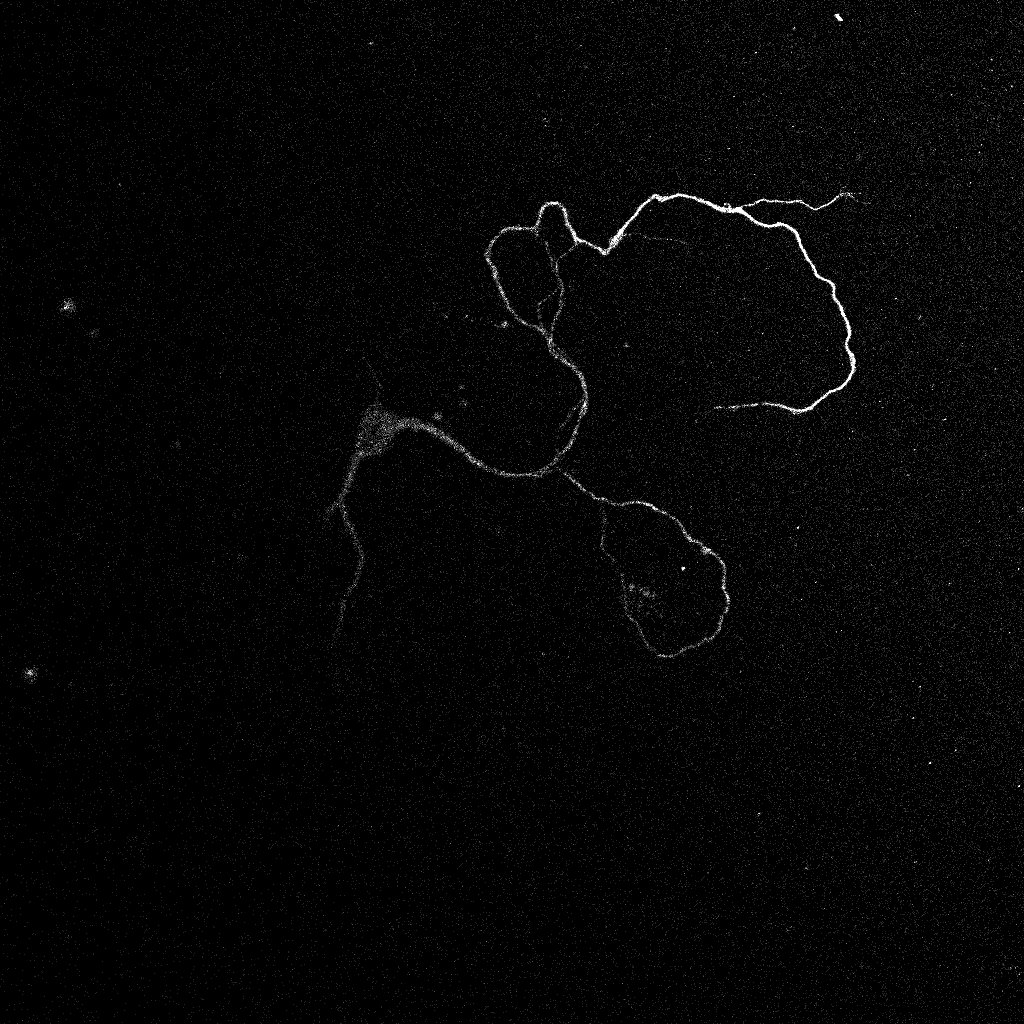

Supplement: Supplementary file 11 — Source Data Fig. 7 [file 44319_2024_64_MOESM11_ESM.zip › 7C/RhoGDI WT:p75NTR WT.jpg]

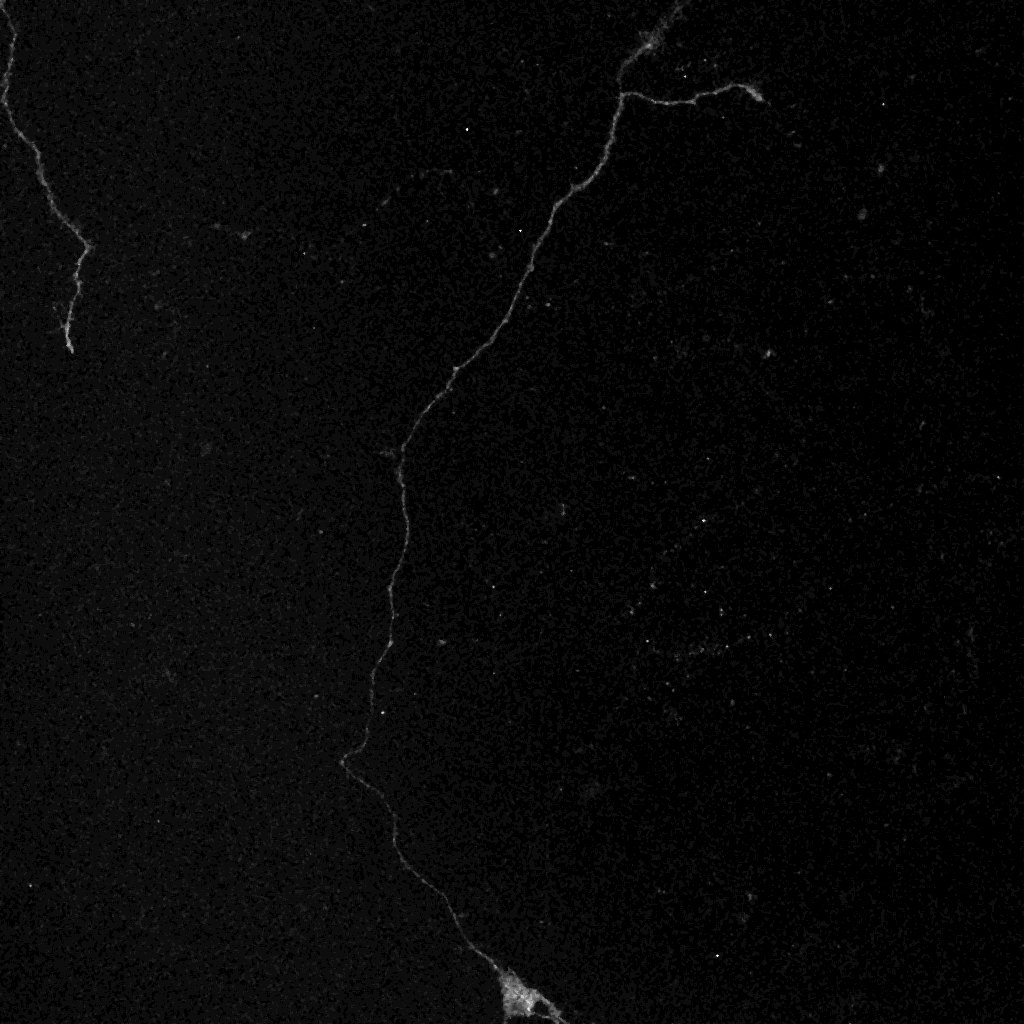

Supplement: Supplementary file 11 — Source Data Fig. 7 [file 44319_2024_64_MOESM11_ESM.zip › 7C/RhoGDI WT: p75NTR KO.jpg]

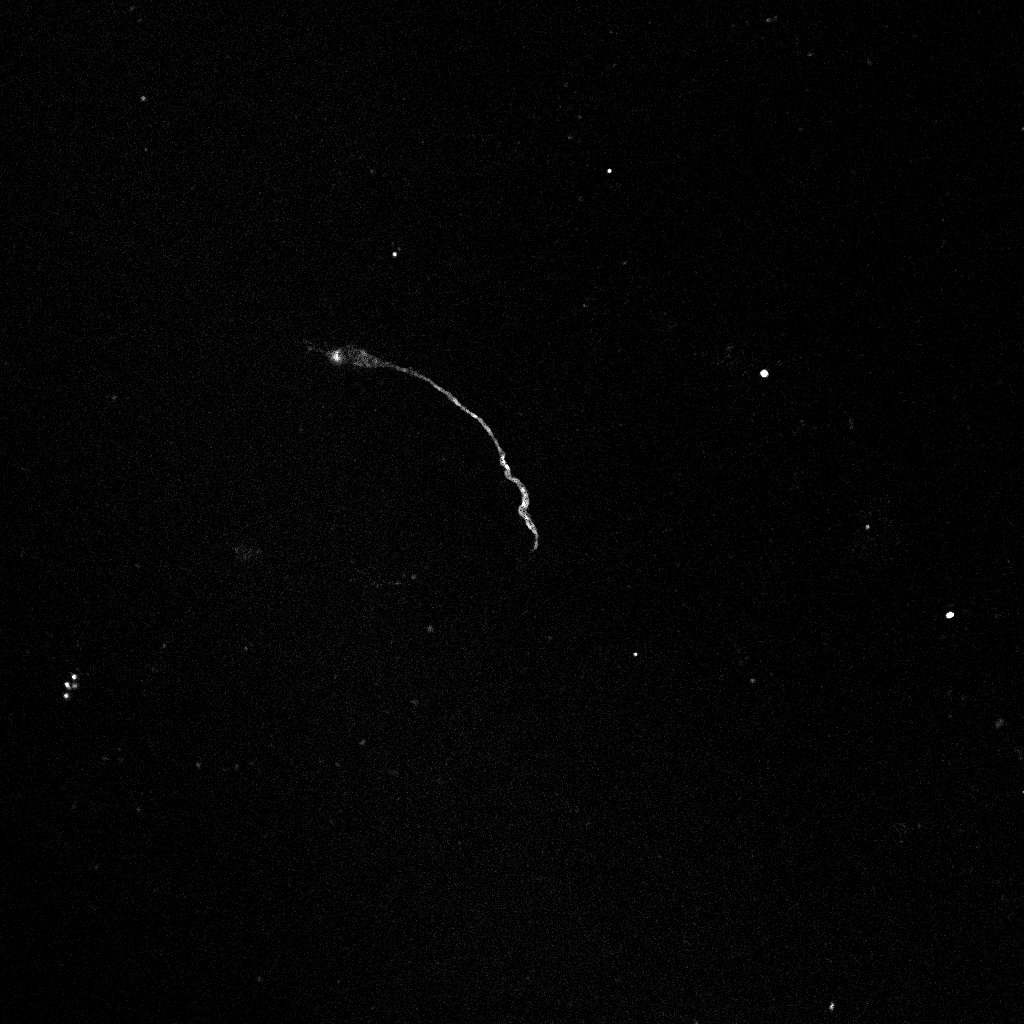

Supplement: Supplementary file 11 — Source Data Fig. 7 [file 44319_2024_64_MOESM11_ESM.zip › 7C/RhoGDI S34D:p75NTR WT.jpg]

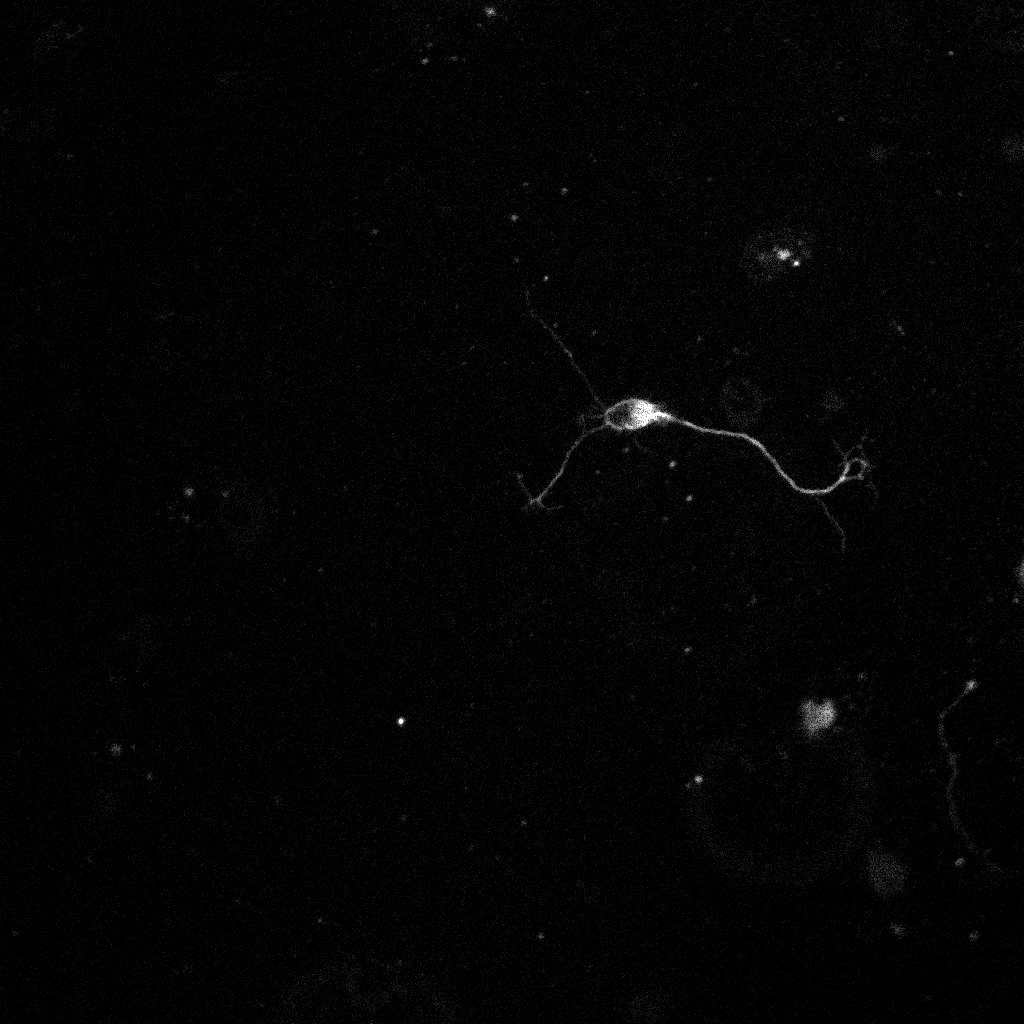

Supplement: Supplementary file 11 — Source Data Fig. 7 [file 44319_2024_64_MOESM11_ESM.zip › 7D/p75NTR WT + MAG.jpg]

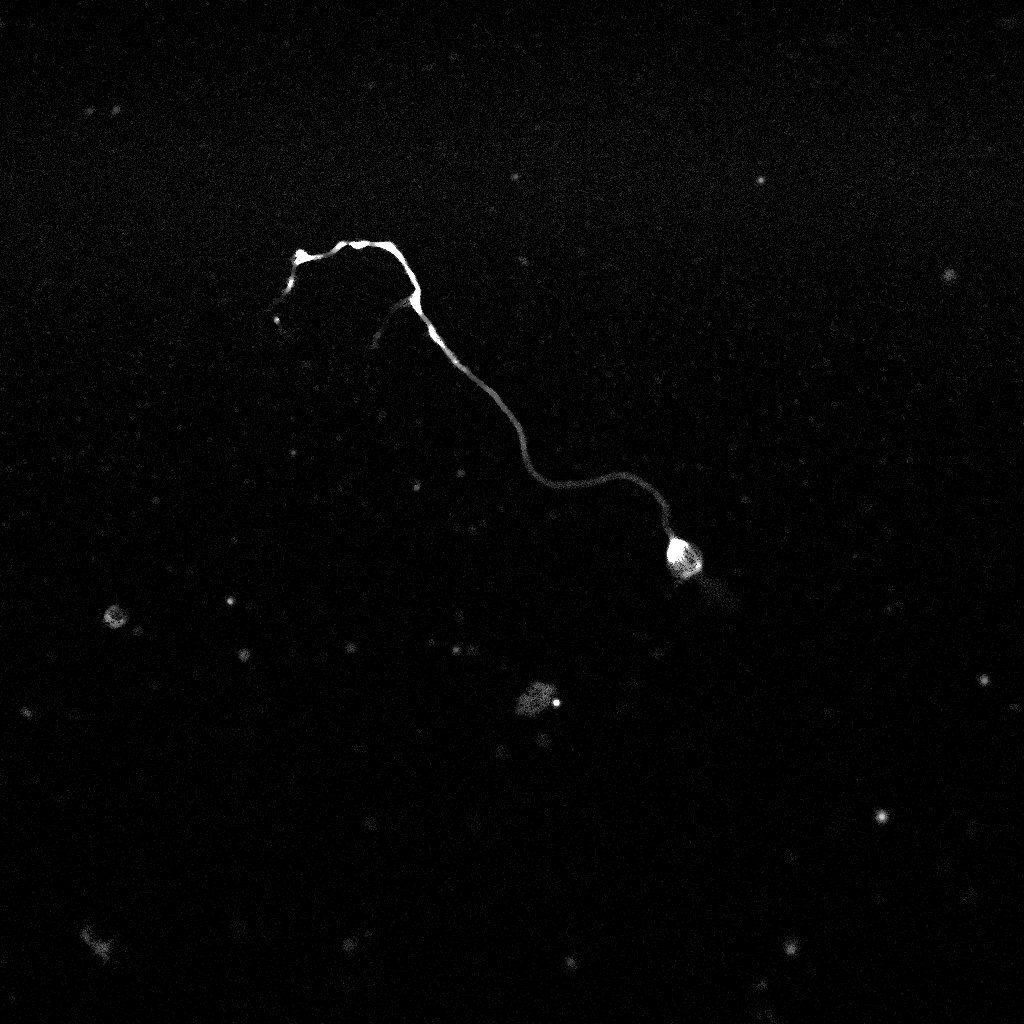

Supplement: Supplementary file 11 — Source Data Fig. 7 [file 44319_2024_64_MOESM11_ESM.zip › 7D/p75NTR K303A + MAG.jpg]

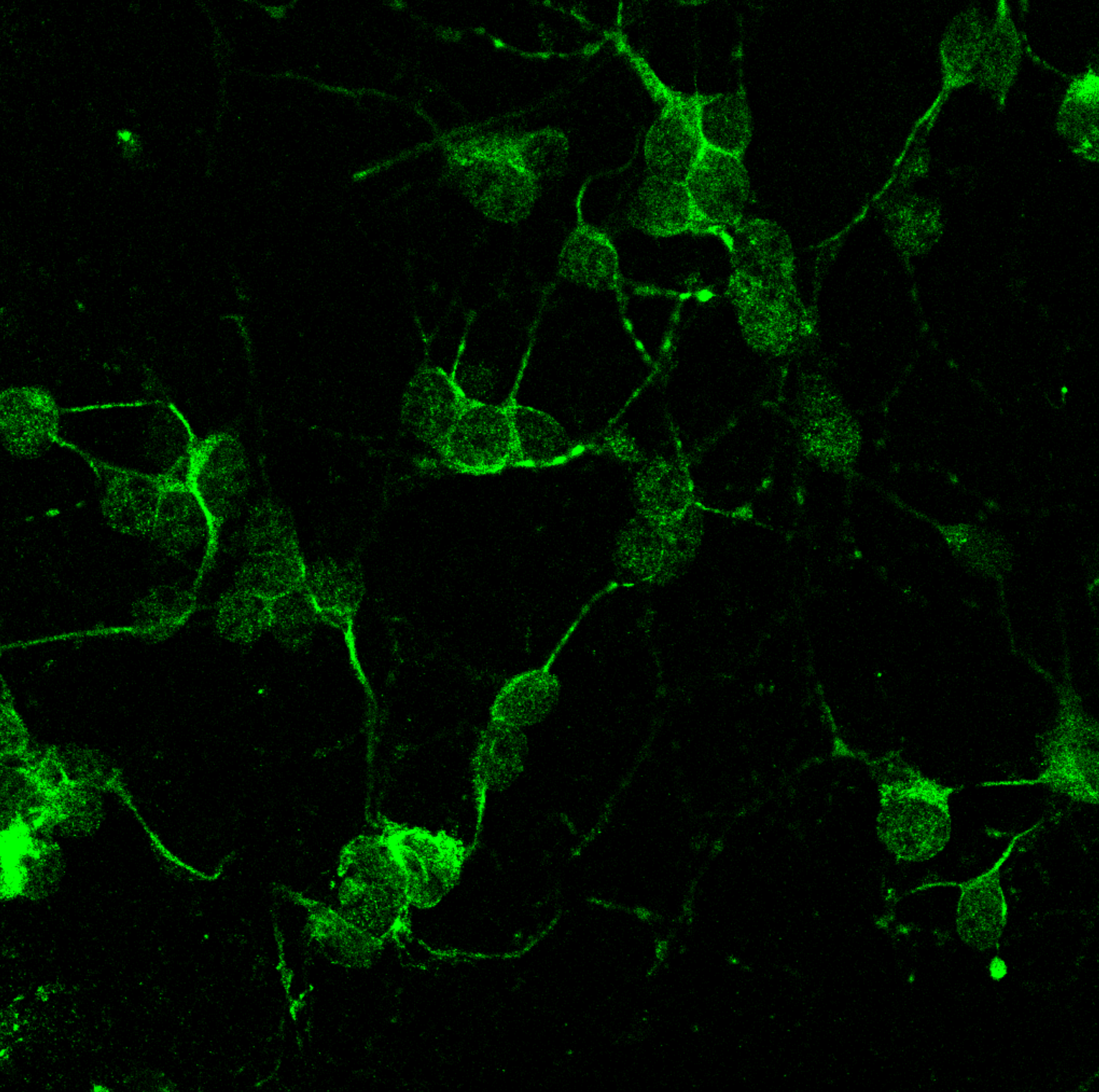

Supplement: Supplementary file 11 — Source Data Fig. 7 [file 44319_2024_64_MOESM11_ESM.zip › 7E/Fc-Control/eGFP-RhoGDI WT.png]

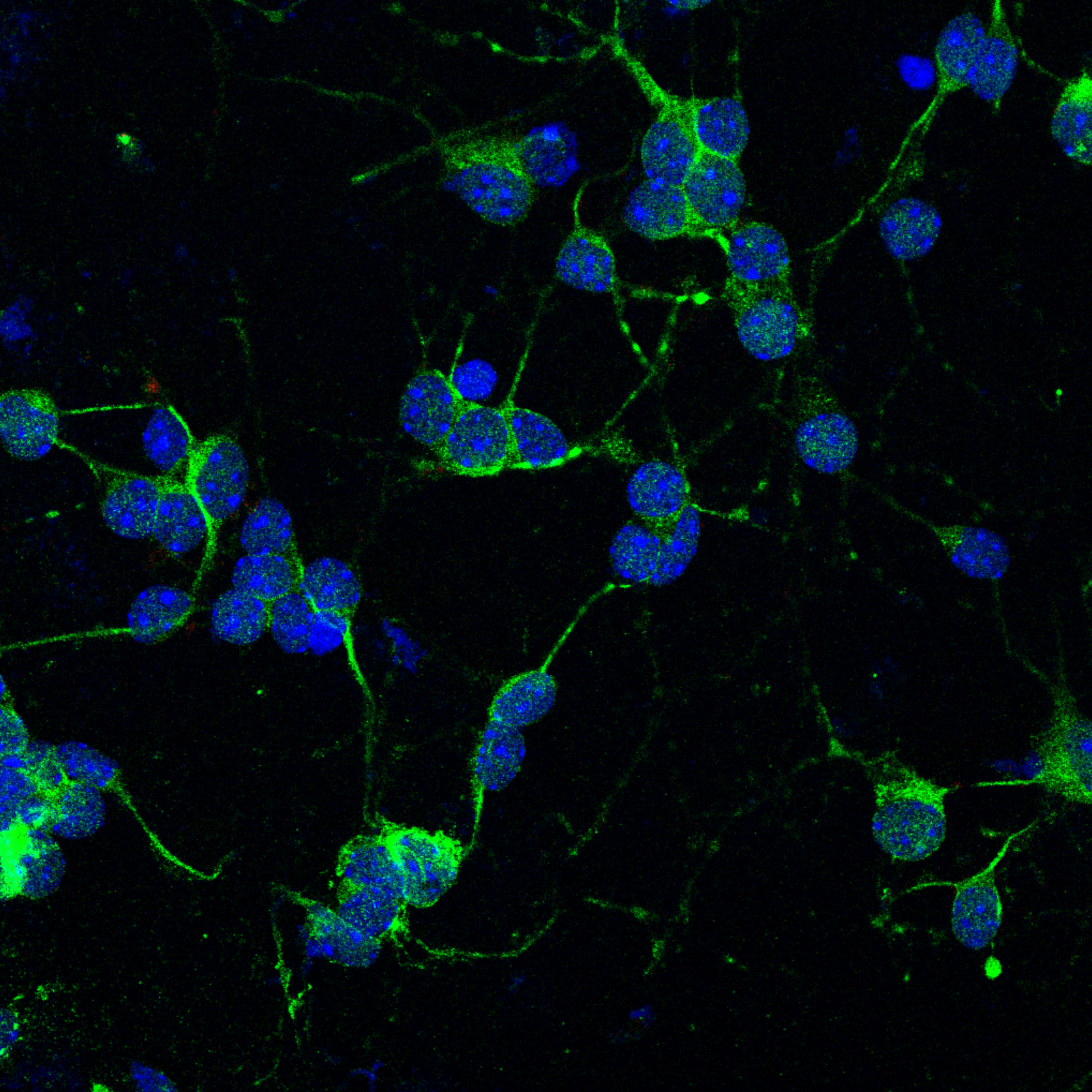

Supplement: Supplementary file 11 — Source Data Fig. 7 [file 44319_2024_64_MOESM11_ESM.zip › 7E/Fc-Control/Merge.png]

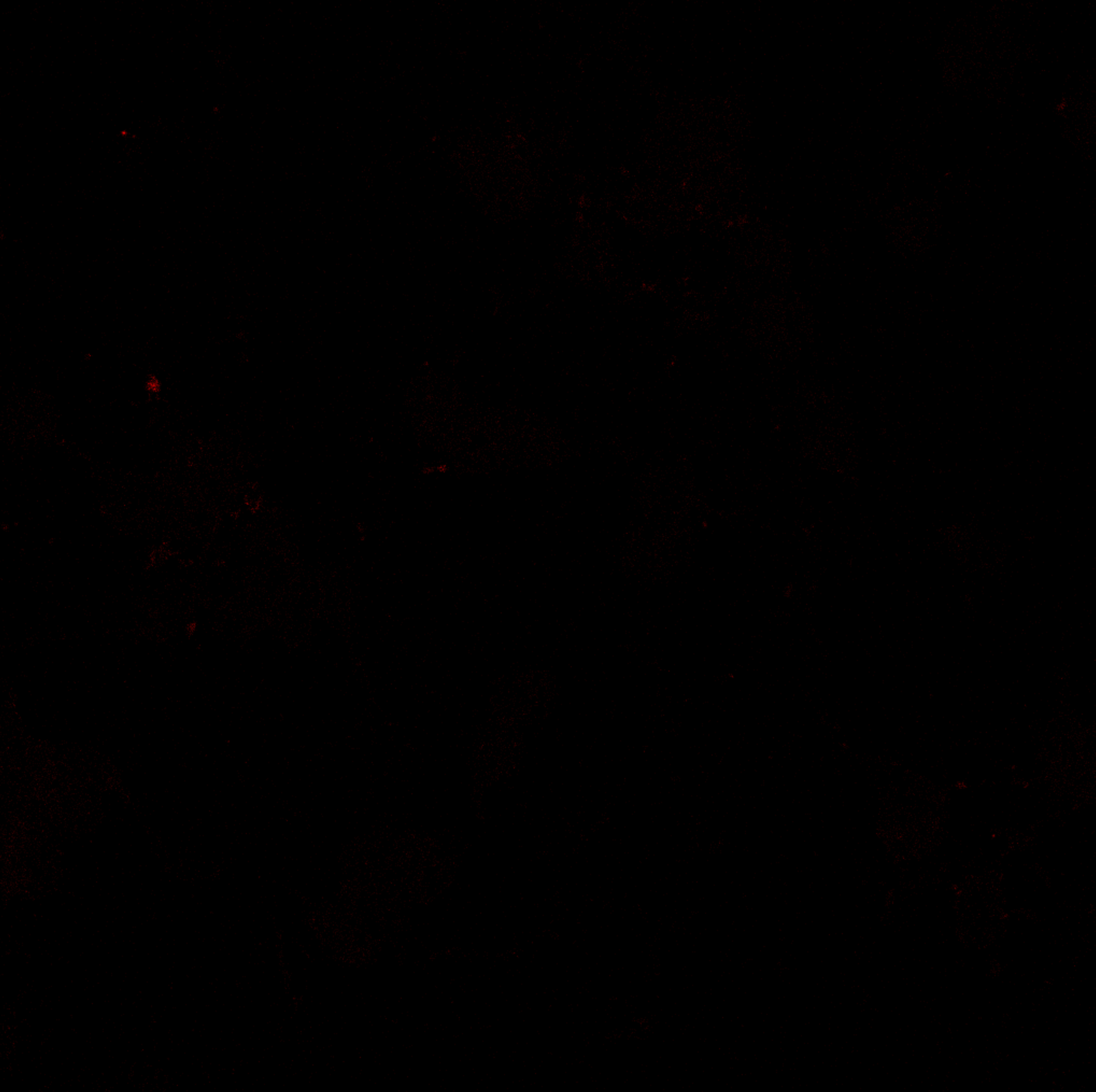

Supplement: Supplementary file 11 — Source Data Fig. 7 [file 44319_2024_64_MOESM11_ESM.zip › 7E/Fc-Control/Cleaved caspase 3.png]

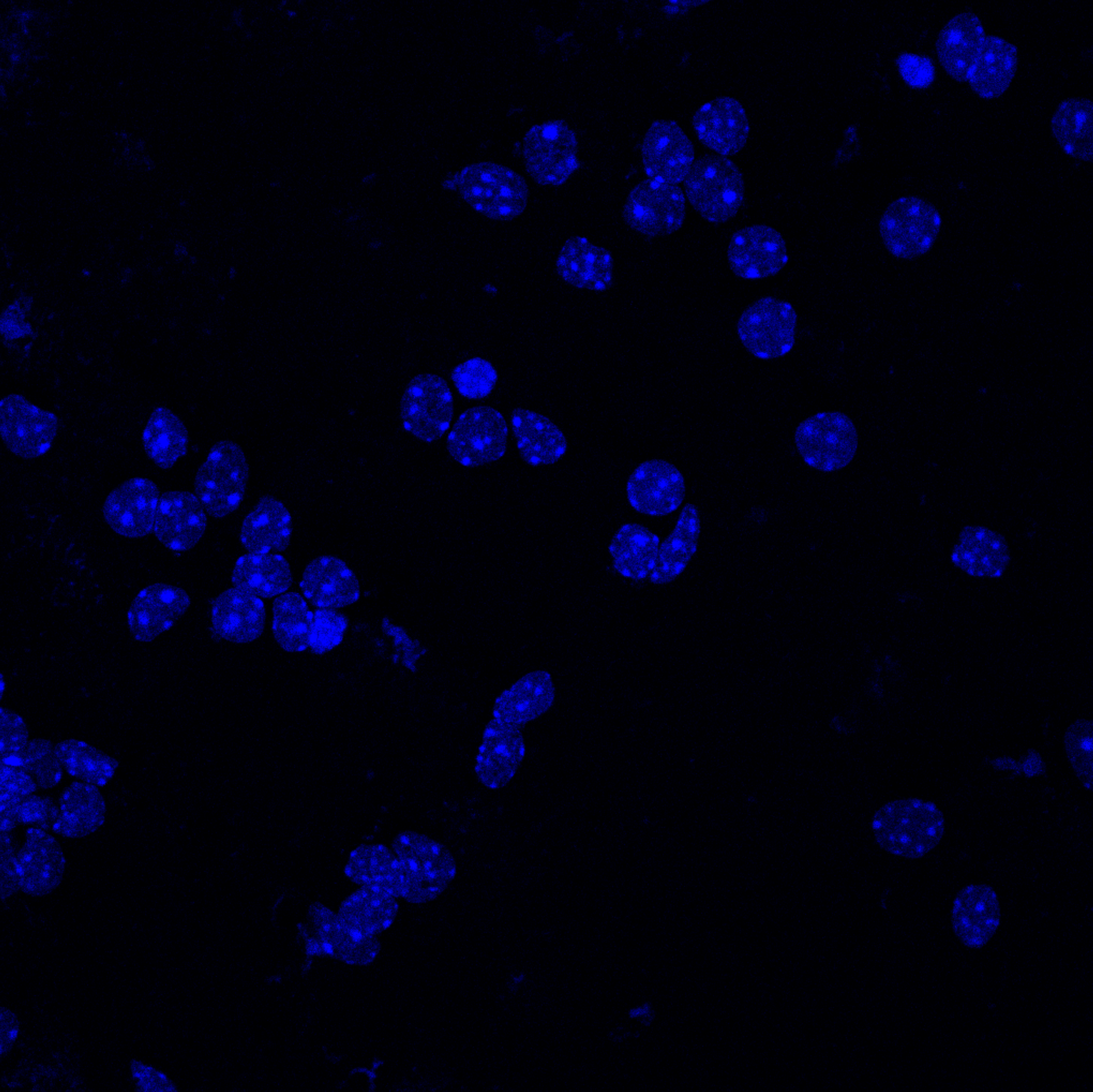

Supplement: Supplementary file 11 — Source Data Fig. 7 [file 44319_2024_64_MOESM11_ESM.zip › 7E/Fc-Control/DAPI.png]

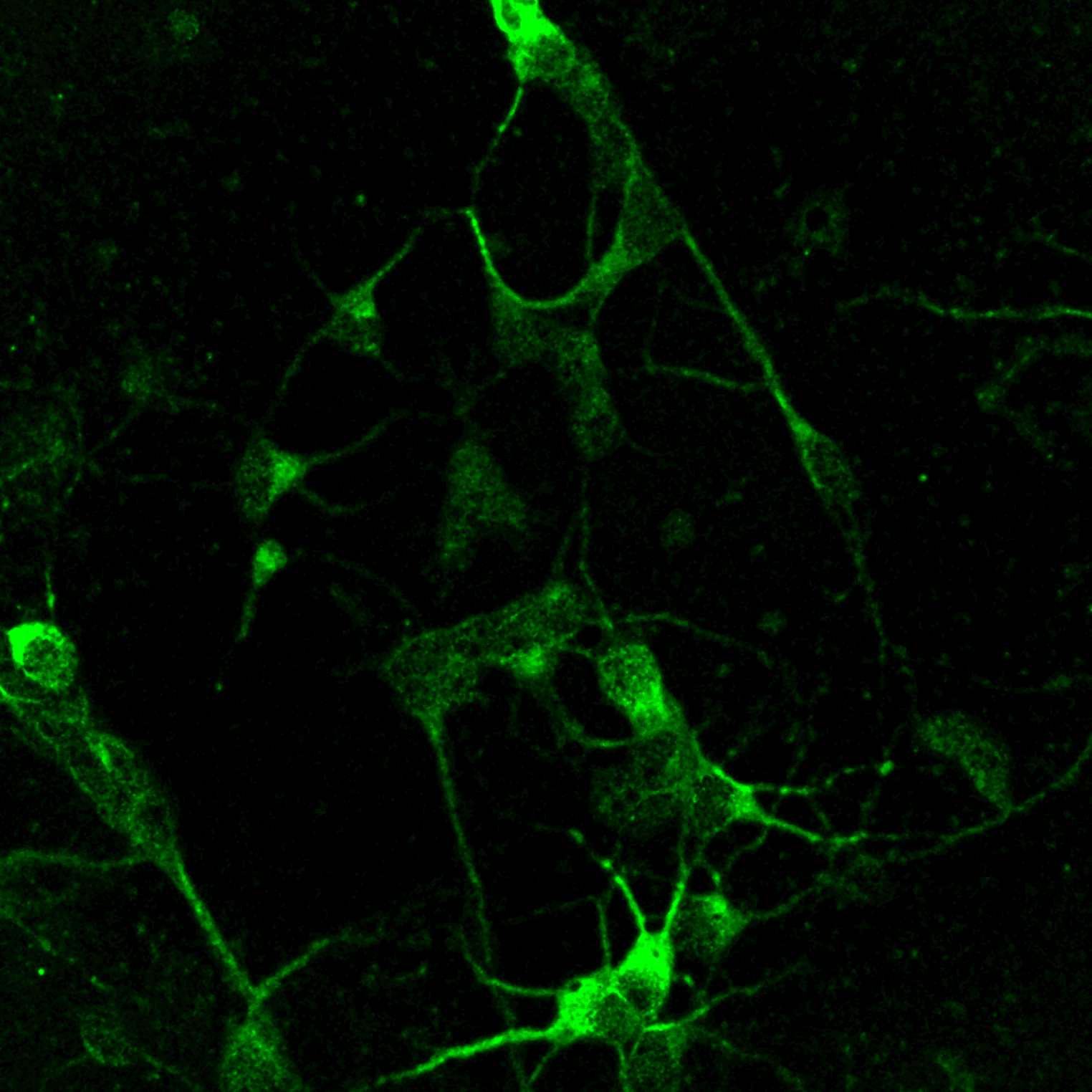

Supplement: Supplementary file 11 — Source Data Fig. 7 [file 44319_2024_64_MOESM11_ESM.zip › 7E/Fc-MAG/eGFP-RhoGDI WT.png]

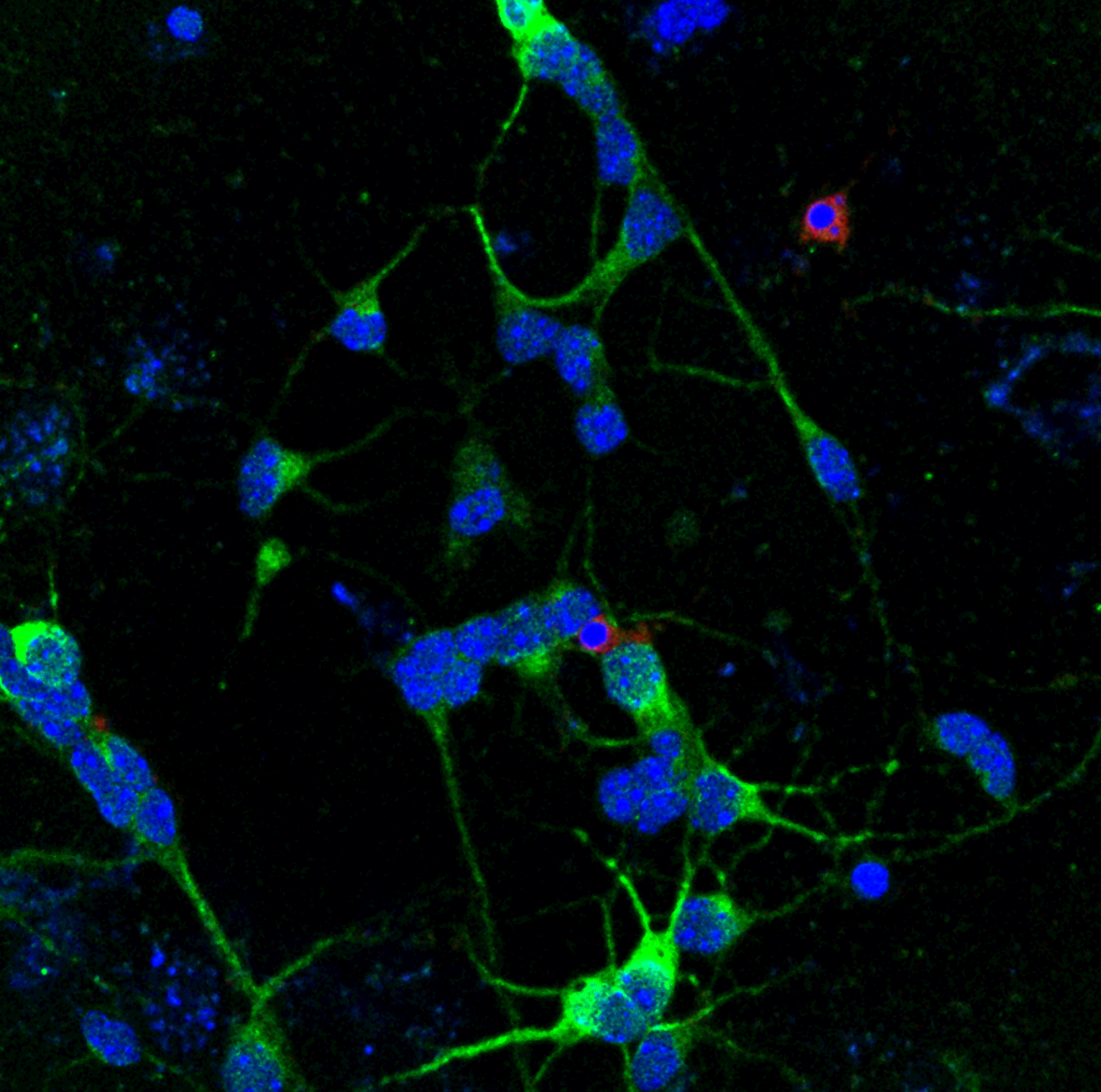

Supplement: Supplementary file 11 — Source Data Fig. 7 [file 44319_2024_64_MOESM11_ESM.zip › 7E/Fc-MAG/Merge.png]

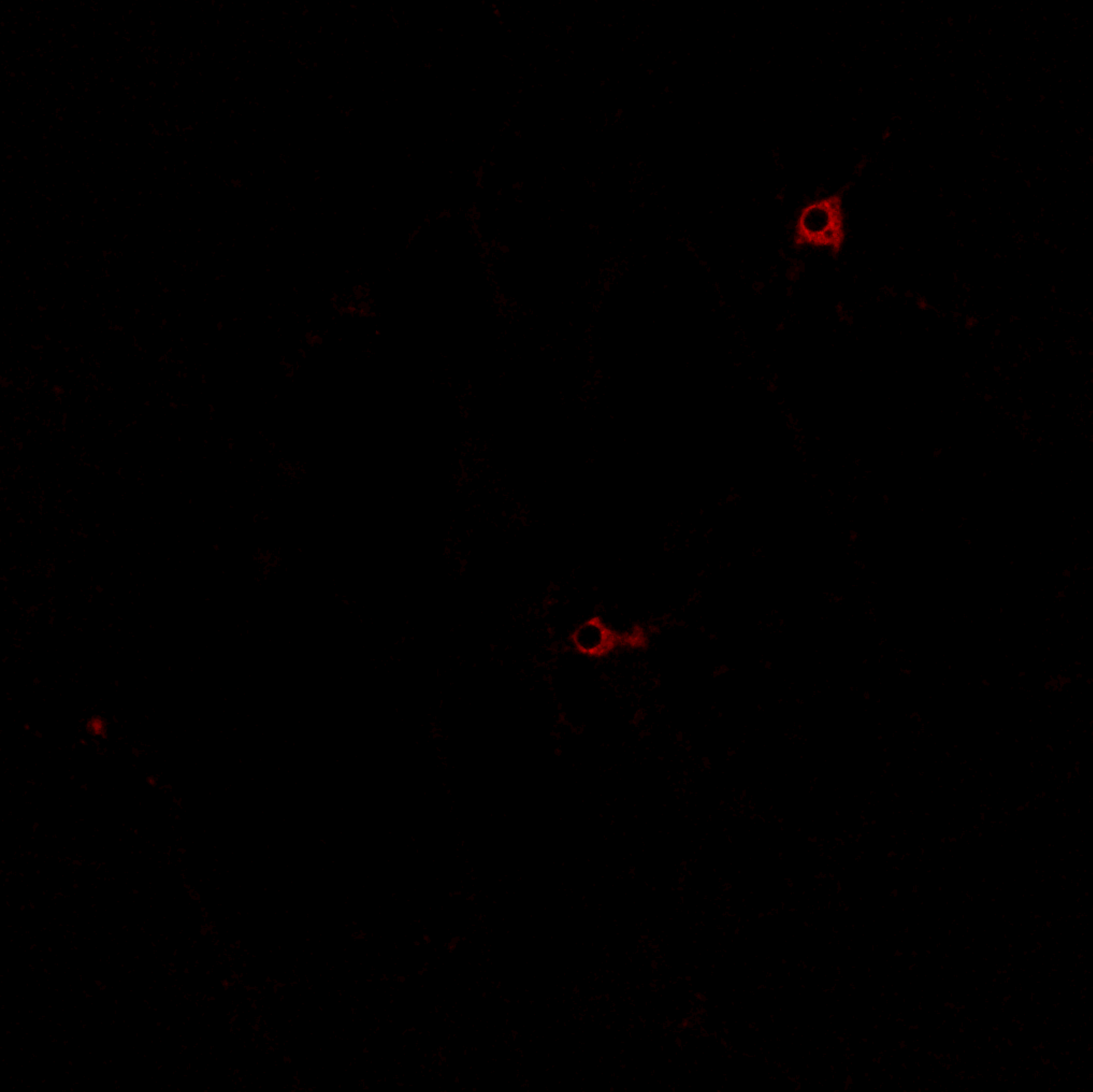

Supplement: Supplementary file 11 — Source Data Fig. 7 [file 44319_2024_64_MOESM11_ESM.zip › 7E/Fc-MAG/Cleaved caspase 3.png]

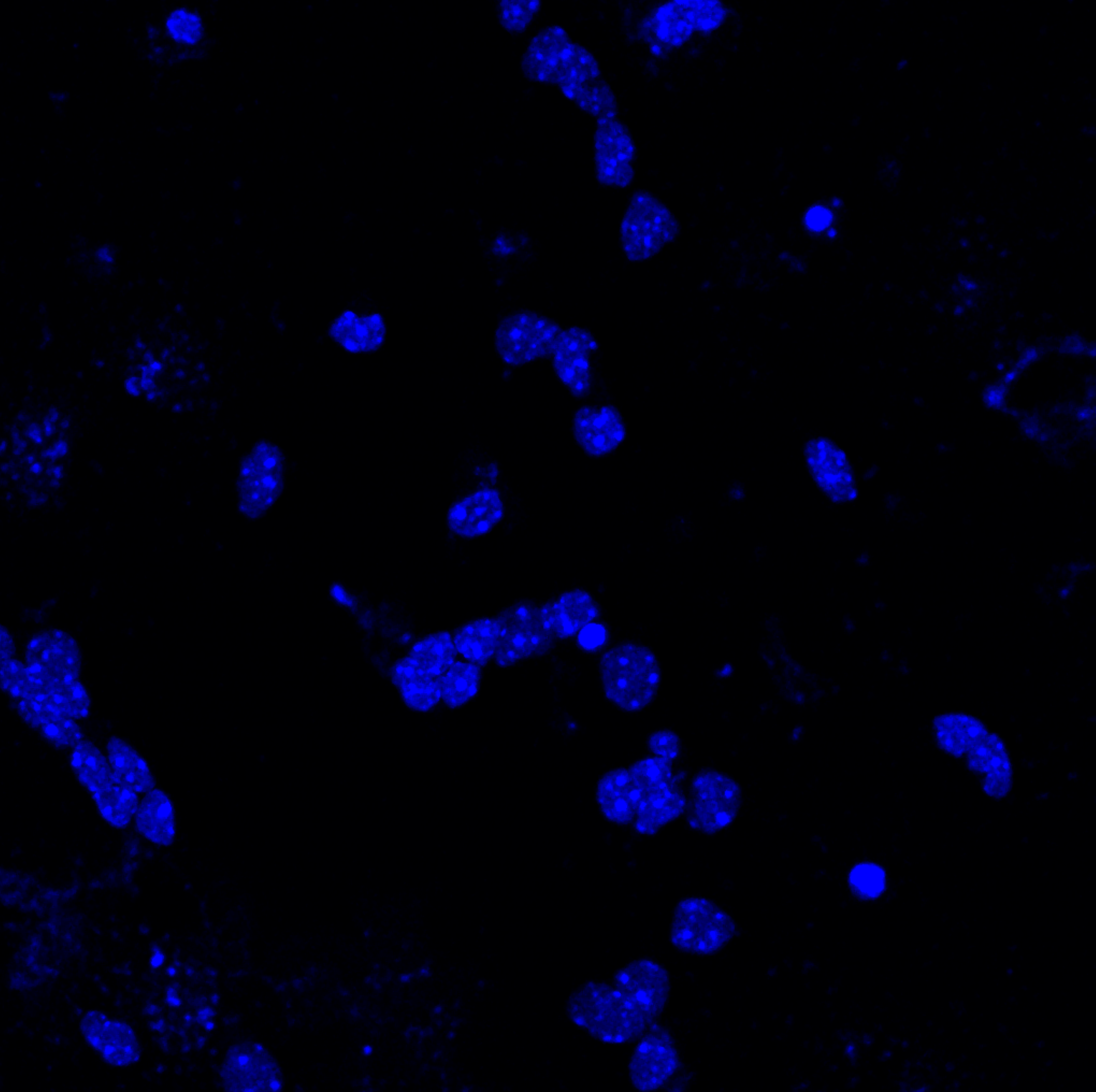

Supplement: Supplementary file 11 — Source Data Fig. 7 [file 44319_2024_64_MOESM11_ESM.zip › 7E/Fc-MAG/DAPI.png]

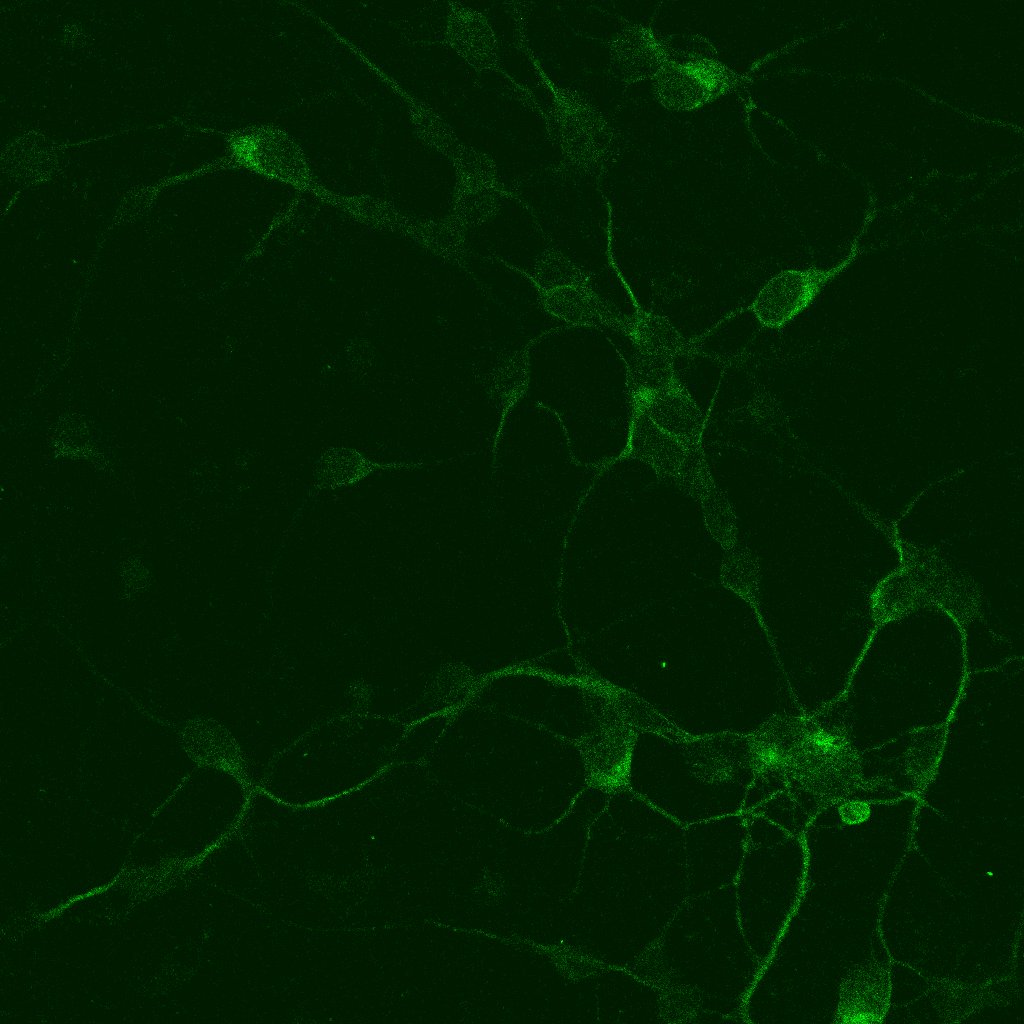

Supplement: Supplementary file 11 — Source Data Fig. 7 [file 44319_2024_64_MOESM11_ESM.zip › 7G/Fc-Control/eGFP-RhoGDI S34D.jpg]

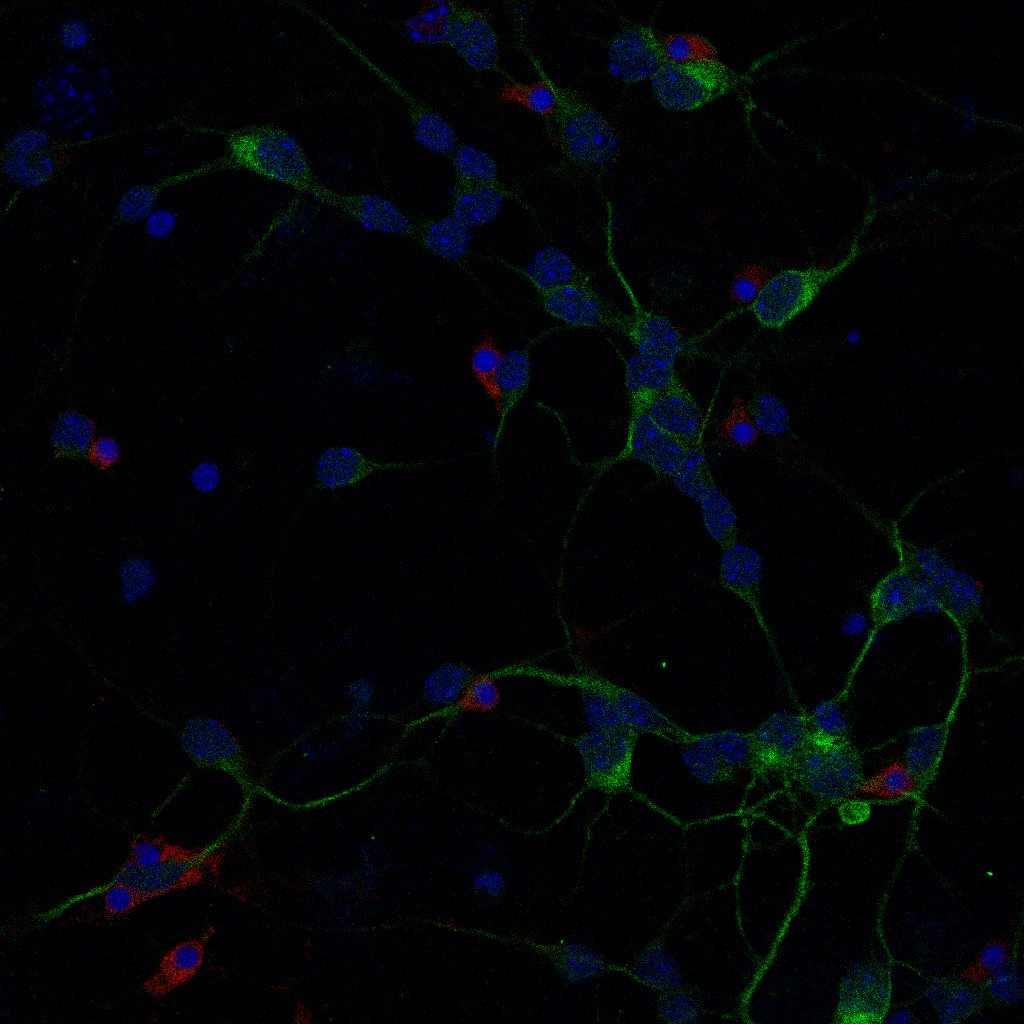

Supplement: Supplementary file 11 — Source Data Fig. 7 [file 44319_2024_64_MOESM11_ESM.zip › 7G/Fc-Control/Merge.jpg]
