# Supplementary figures and images for: RhoGDI phosphorylation by PKC promotes its interaction with death receptor p75NTR to gate axon growth and neuron survival (part 2 of 3)
Source: EMBO Rep. 2024 Jan 22;25(3):30. doi: 10.1038/s44319-024-00064-2 (PMC10933337; doi:10.1038/s44319-024-00064-2)

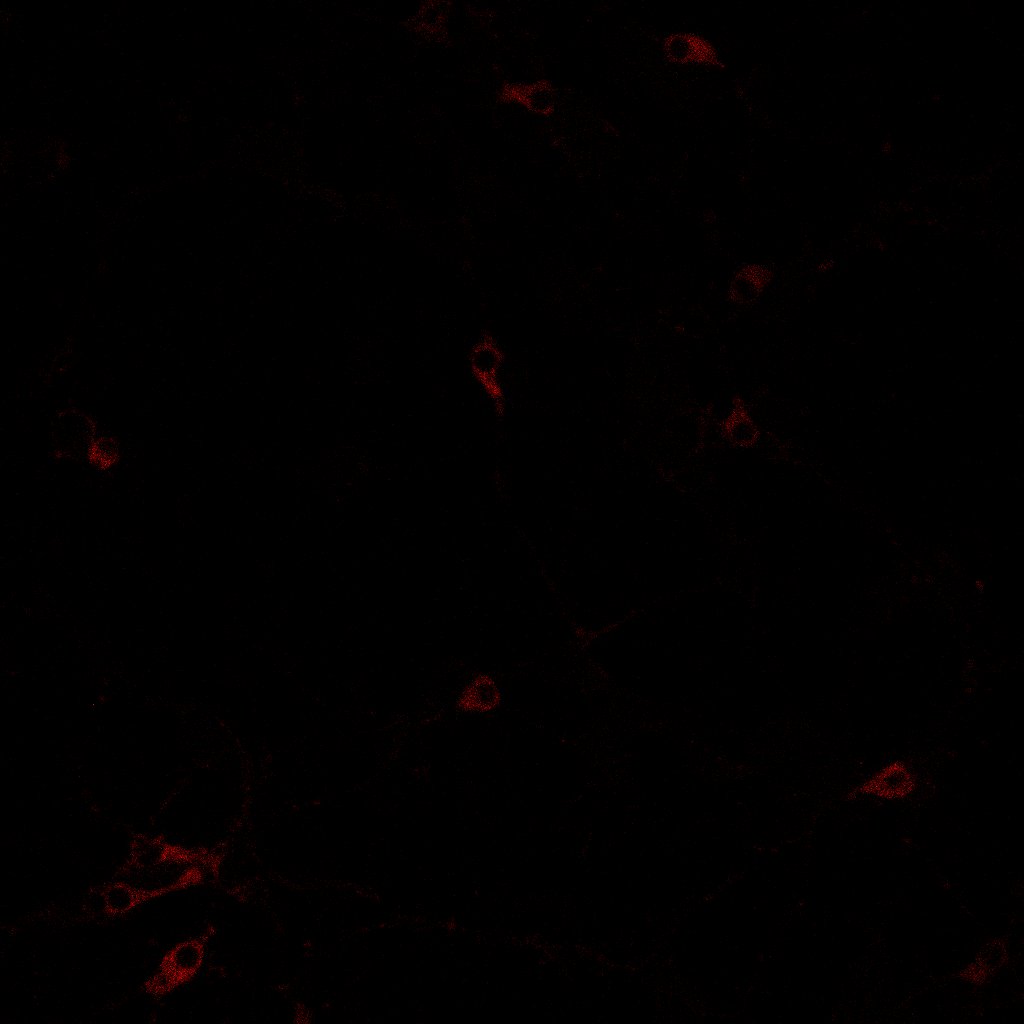

Supplement: Supplementary file 11 — Source Data Fig. 7 [file 44319_2024_64_MOESM11_ESM.zip › 7G/Fc-Control/Cleaved caspase 3.jpg]

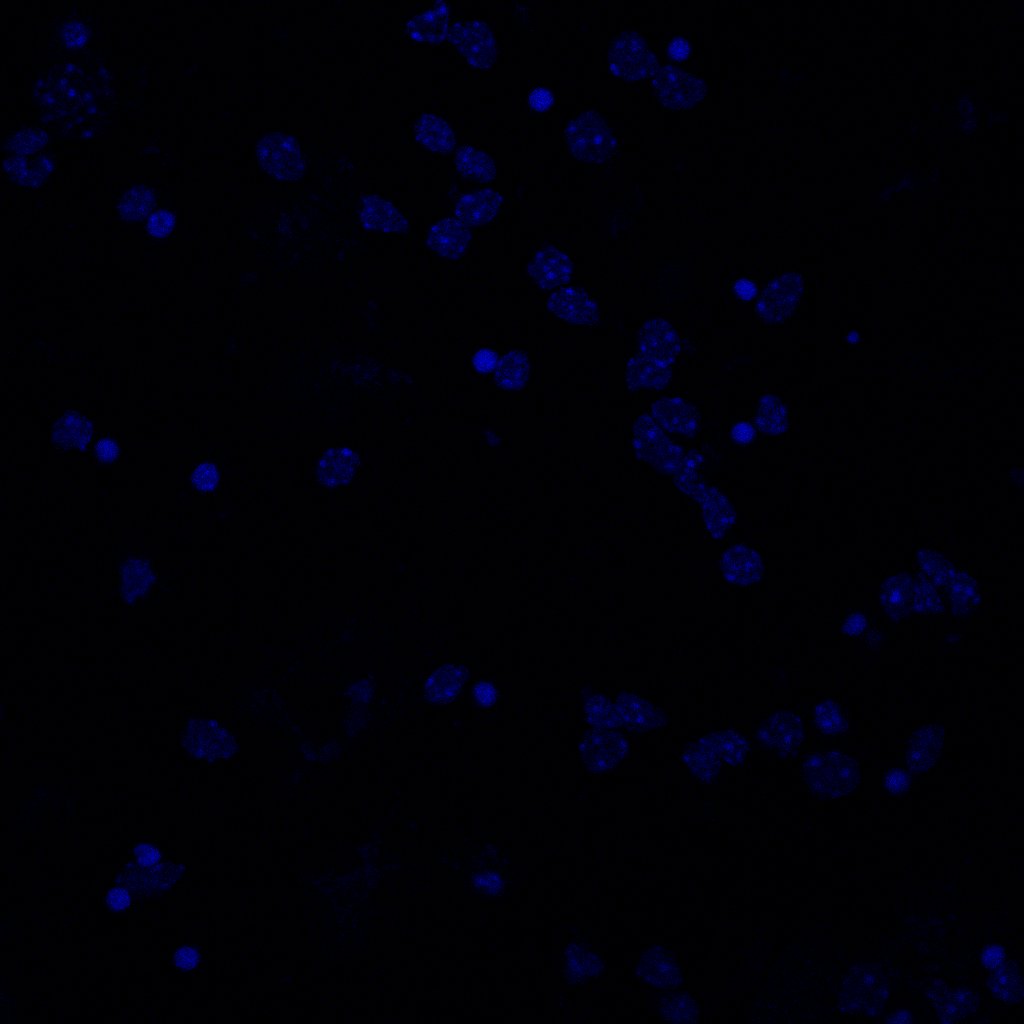

Supplement: Supplementary file 11 — Source Data Fig. 7 [file 44319_2024_64_MOESM11_ESM.zip › 7G/Fc-Control/DAPI.jpg]

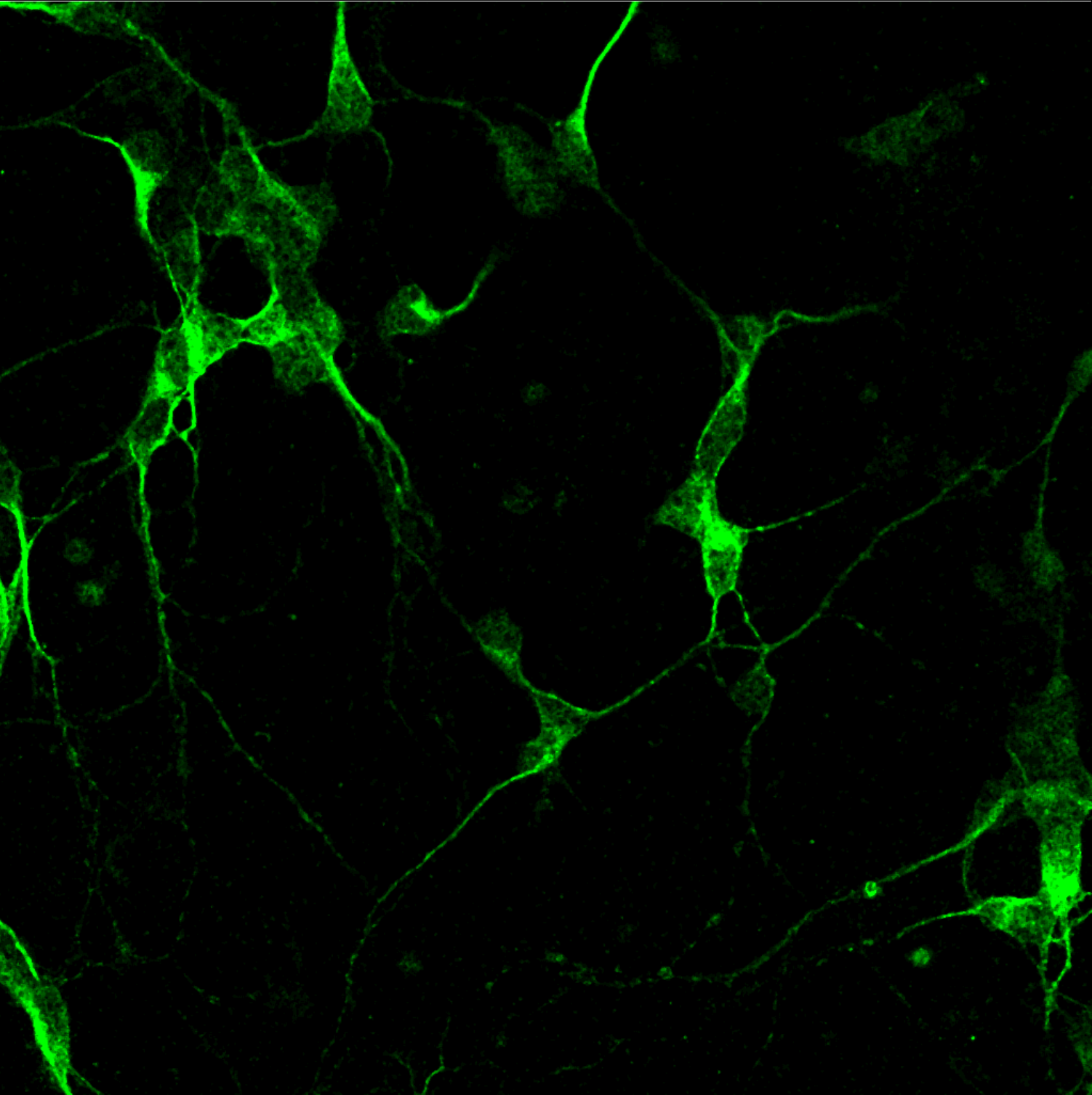

Supplement: Supplementary file 11 — Source Data Fig. 7 [file 44319_2024_64_MOESM11_ESM.zip › 7G/Fc-MAG/eGFP-RhoGDI S34D.png]

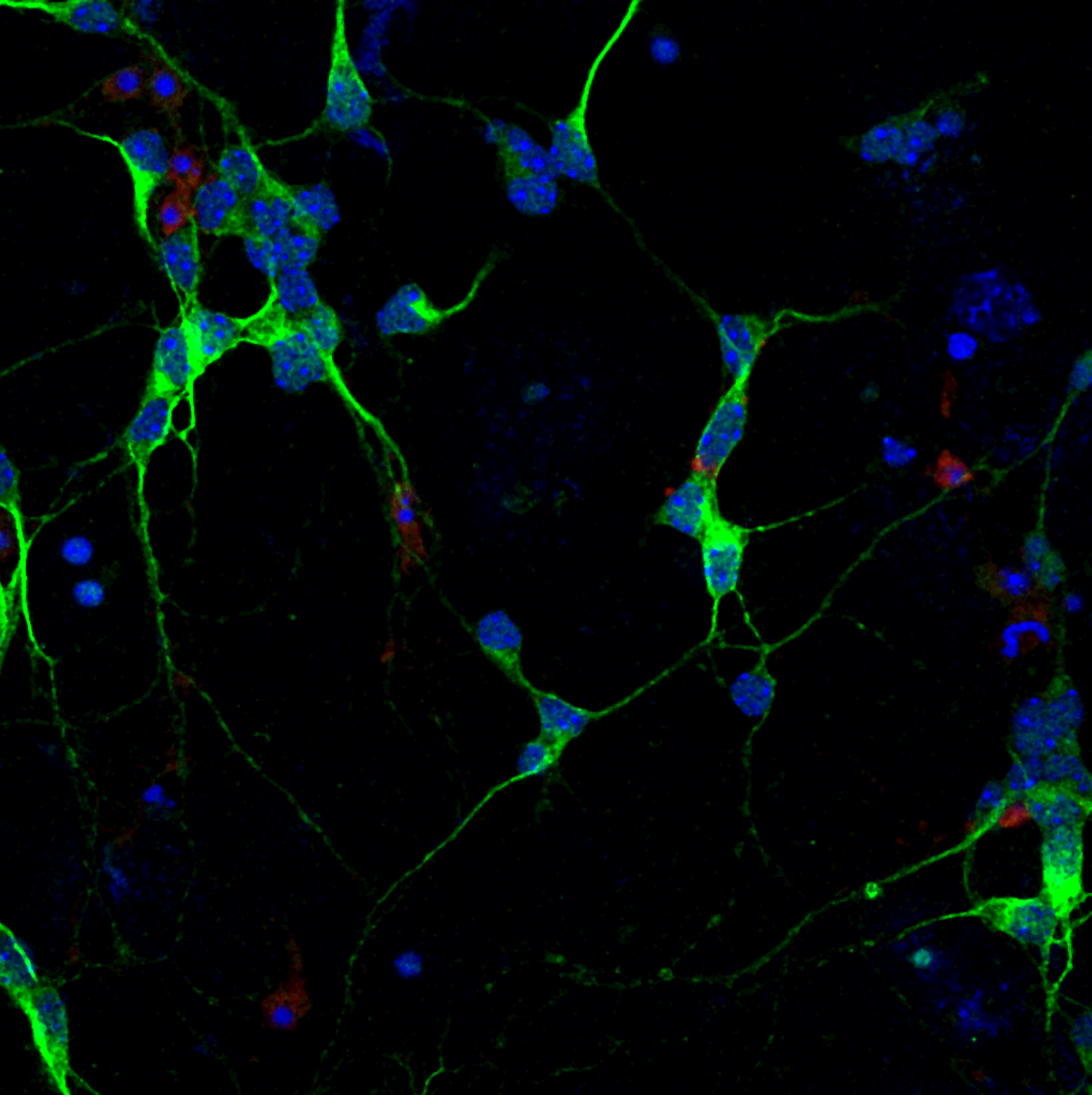

Supplement: Supplementary file 11 — Source Data Fig. 7 [file 44319_2024_64_MOESM11_ESM.zip › 7G/Fc-MAG/Merge.png]

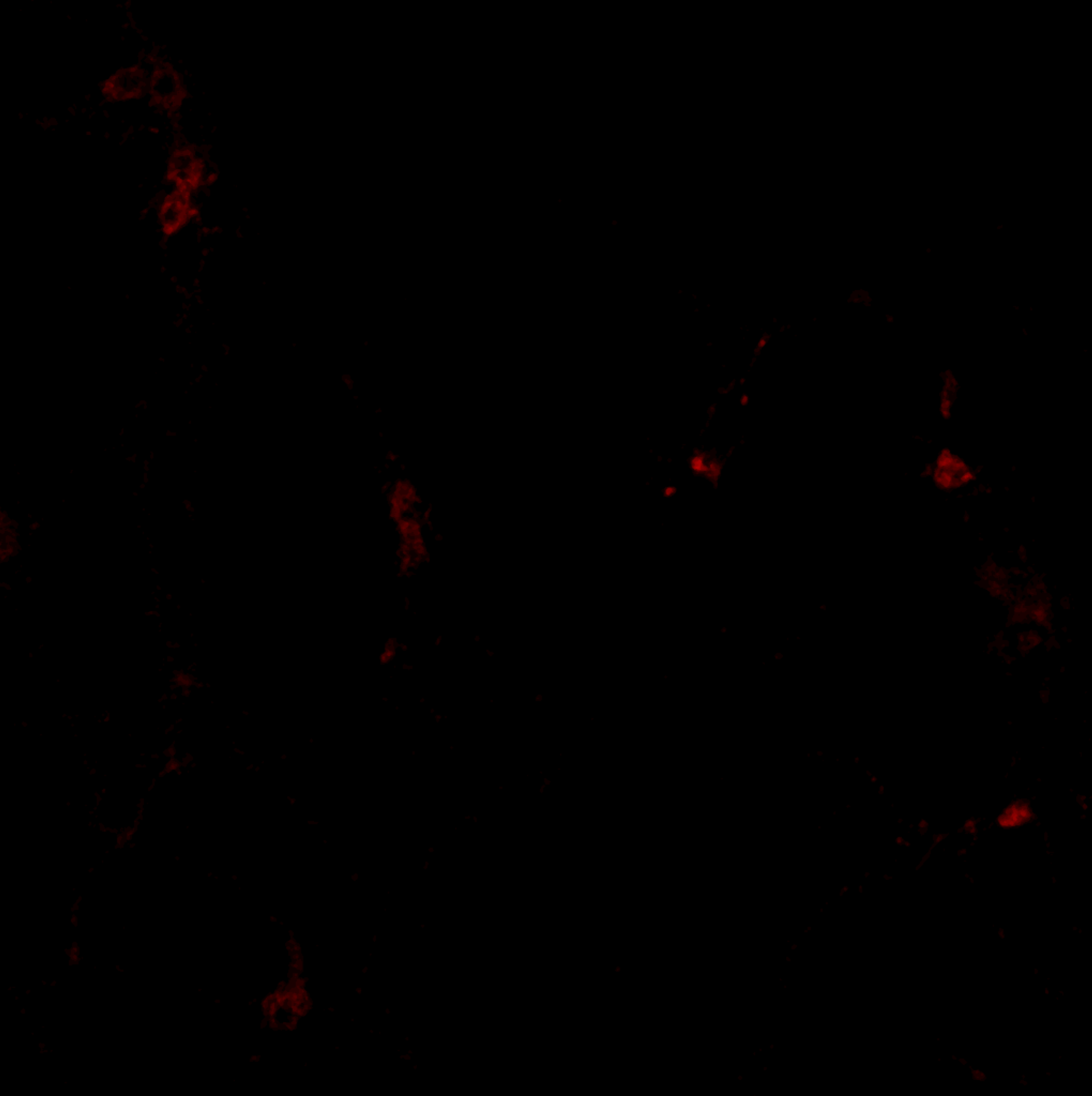

Supplement: Supplementary file 11 — Source Data Fig. 7 [file 44319_2024_64_MOESM11_ESM.zip › 7G/Fc-MAG/Cleaved caspase 3.png]

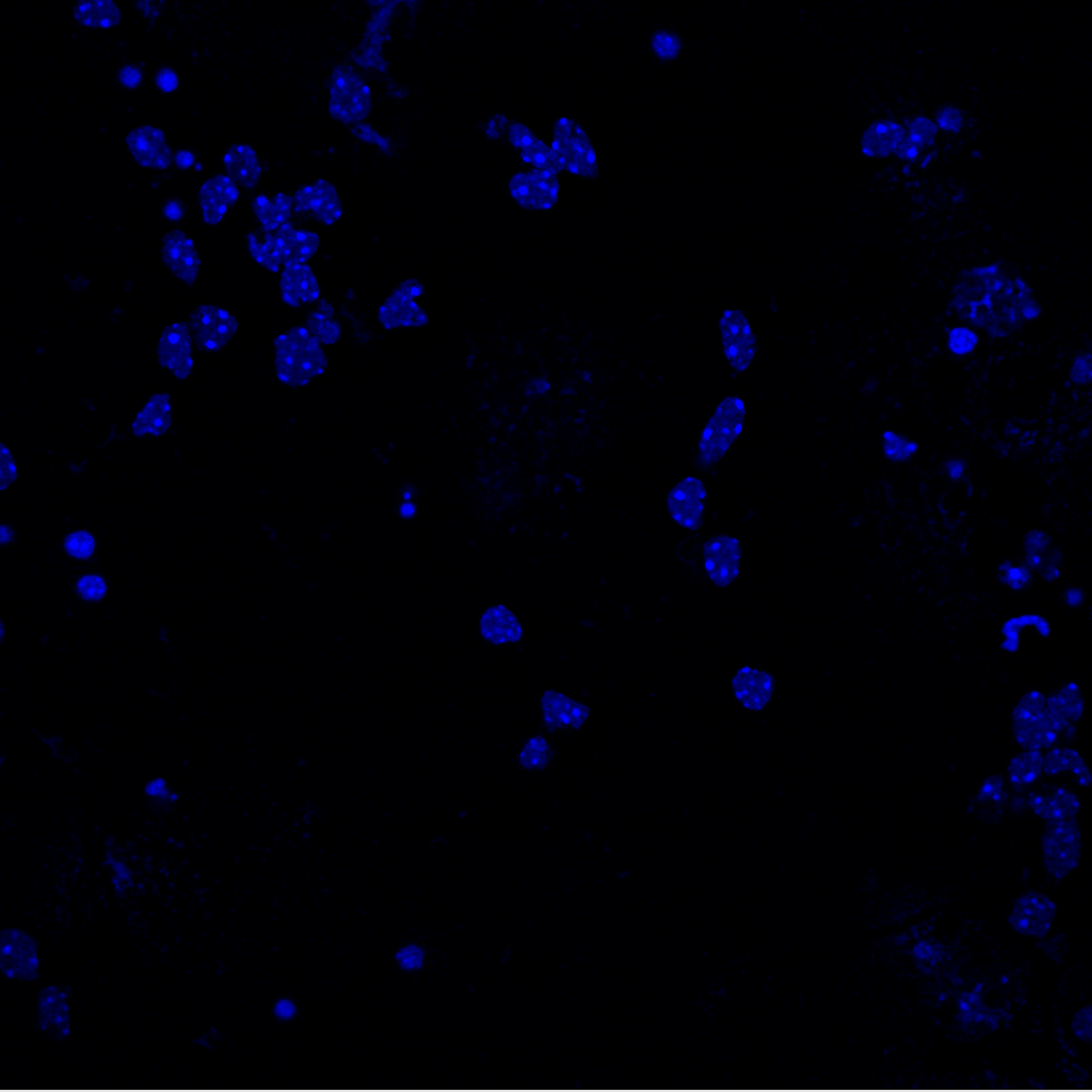

Supplement: Supplementary file 11 — Source Data Fig. 7 [file 44319_2024_64_MOESM11_ESM.zip › 7G/Fc-MAG/DAPI.png]

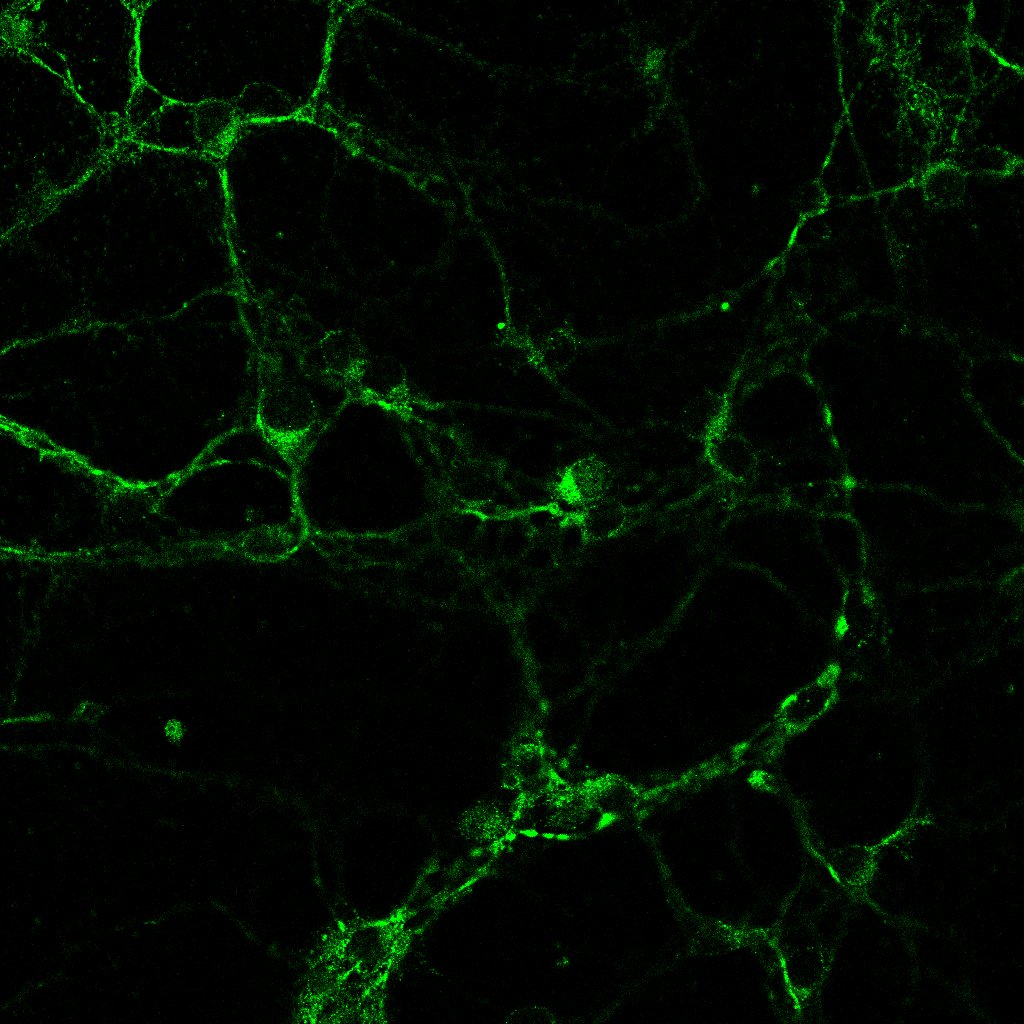

Supplement: Supplementary file 11 — Source Data Fig. 7 [file 44319_2024_64_MOESM11_ESM.zip › 7H/p75NTR K303A/eGFP-p75NTR.jpg]

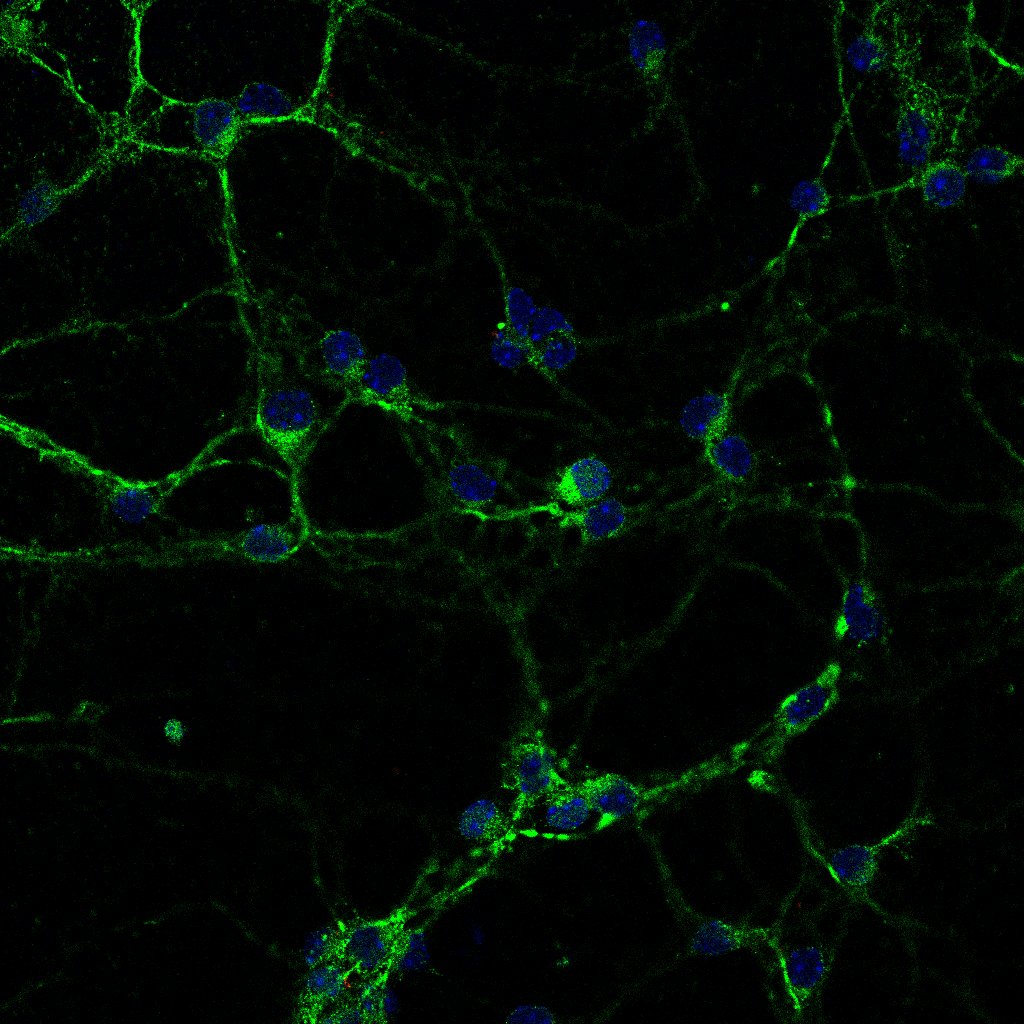

Supplement: Supplementary file 11 — Source Data Fig. 7 [file 44319_2024_64_MOESM11_ESM.zip › 7H/p75NTR K303A/Merge.jpg]

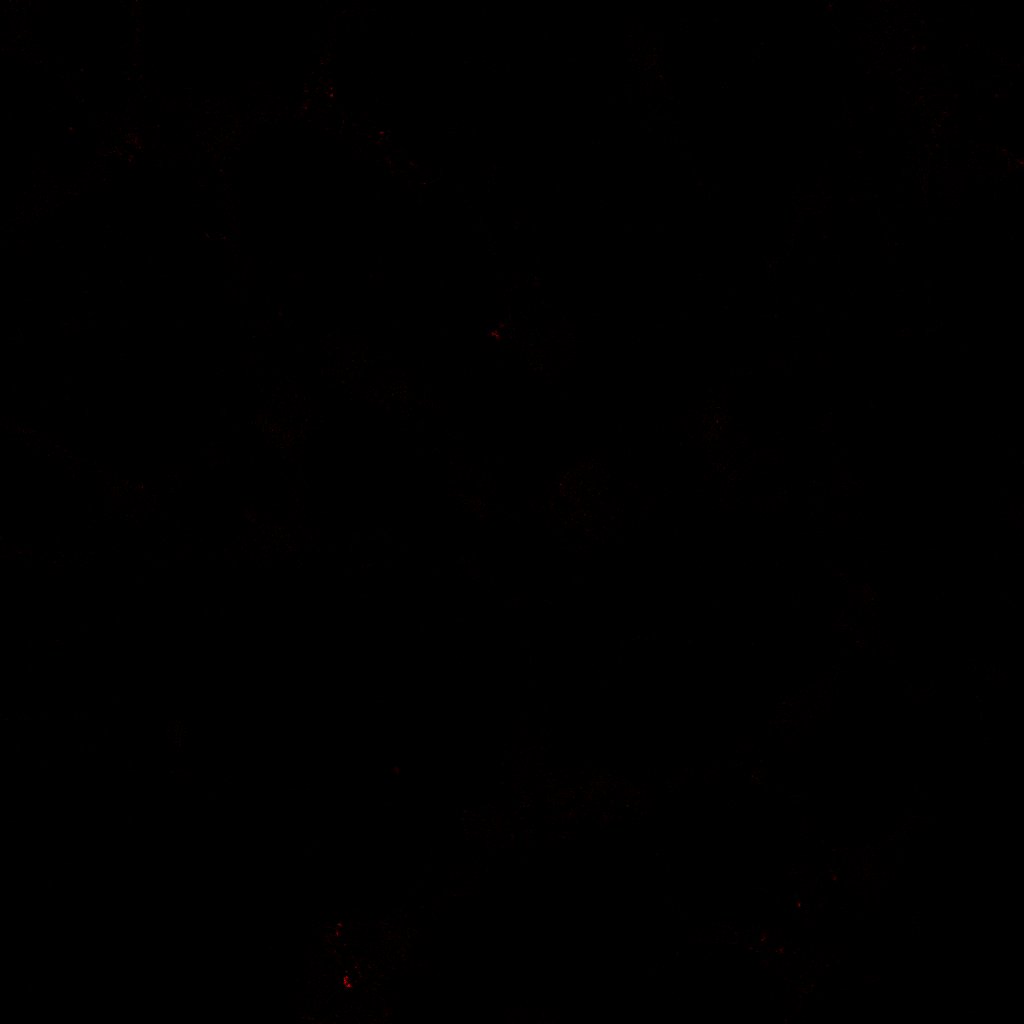

Supplement: Supplementary file 11 — Source Data Fig. 7 [file 44319_2024_64_MOESM11_ESM.zip › 7H/p75NTR K303A/Cleaved caspase 3.jpg]

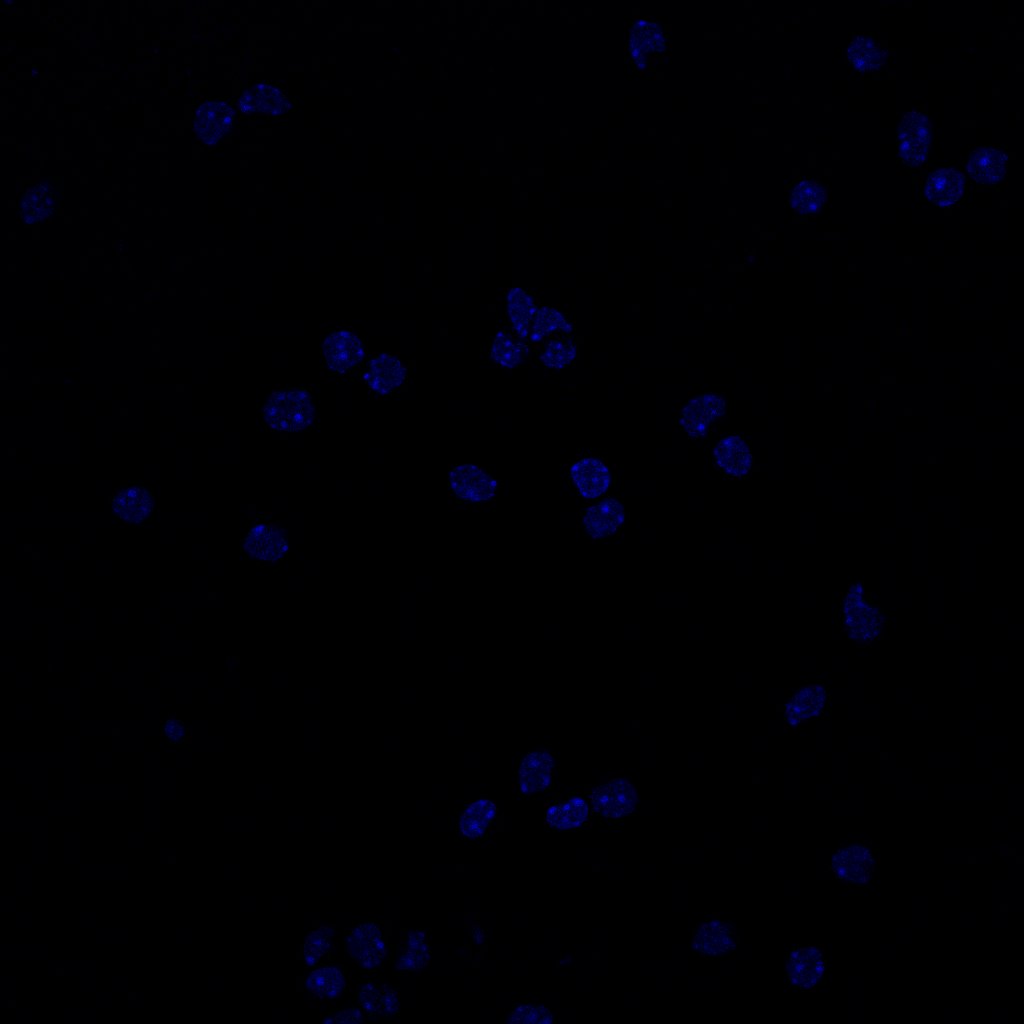

Supplement: Supplementary file 11 — Source Data Fig. 7 [file 44319_2024_64_MOESM11_ESM.zip › 7H/p75NTR K303A/DAPI.jpg]

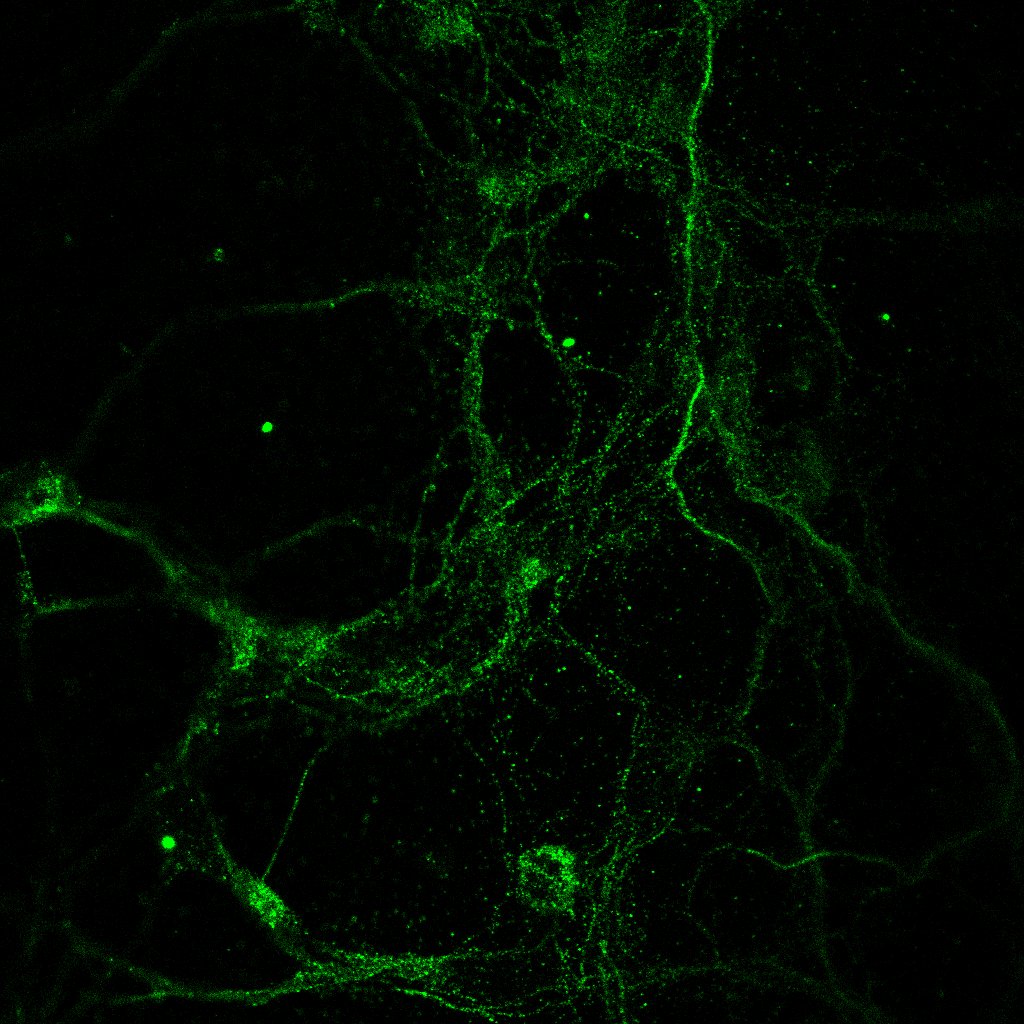

Supplement: Supplementary file 11 — Source Data Fig. 7 [file 44319_2024_64_MOESM11_ESM.zip › 7H/p75NTR WT/eGFP-p75NTR.jpg]

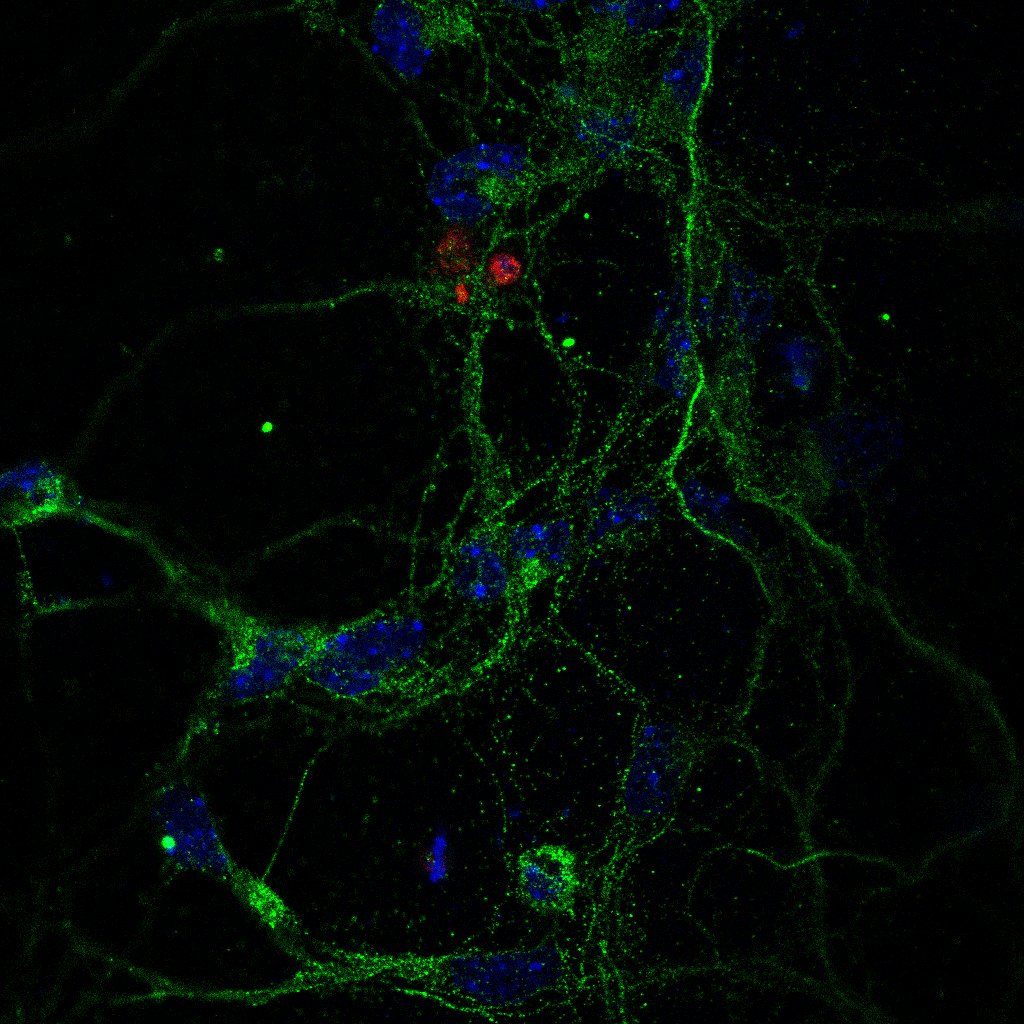

Supplement: Supplementary file 11 — Source Data Fig. 7 [file 44319_2024_64_MOESM11_ESM.zip › 7H/p75NTR WT/Merge.jpg]

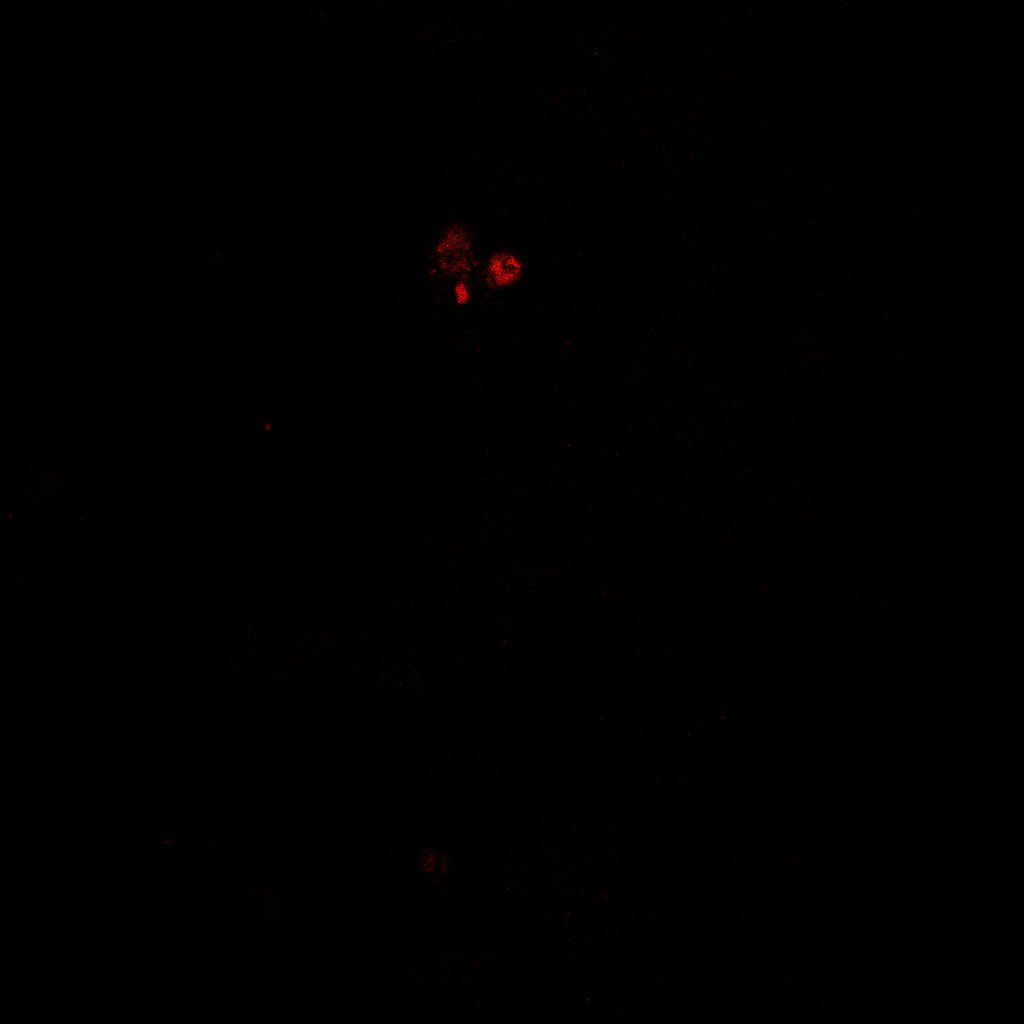

Supplement: Supplementary file 11 — Source Data Fig. 7 [file 44319_2024_64_MOESM11_ESM.zip › 7H/p75NTR WT/Cleaved caspase 3.jpg]

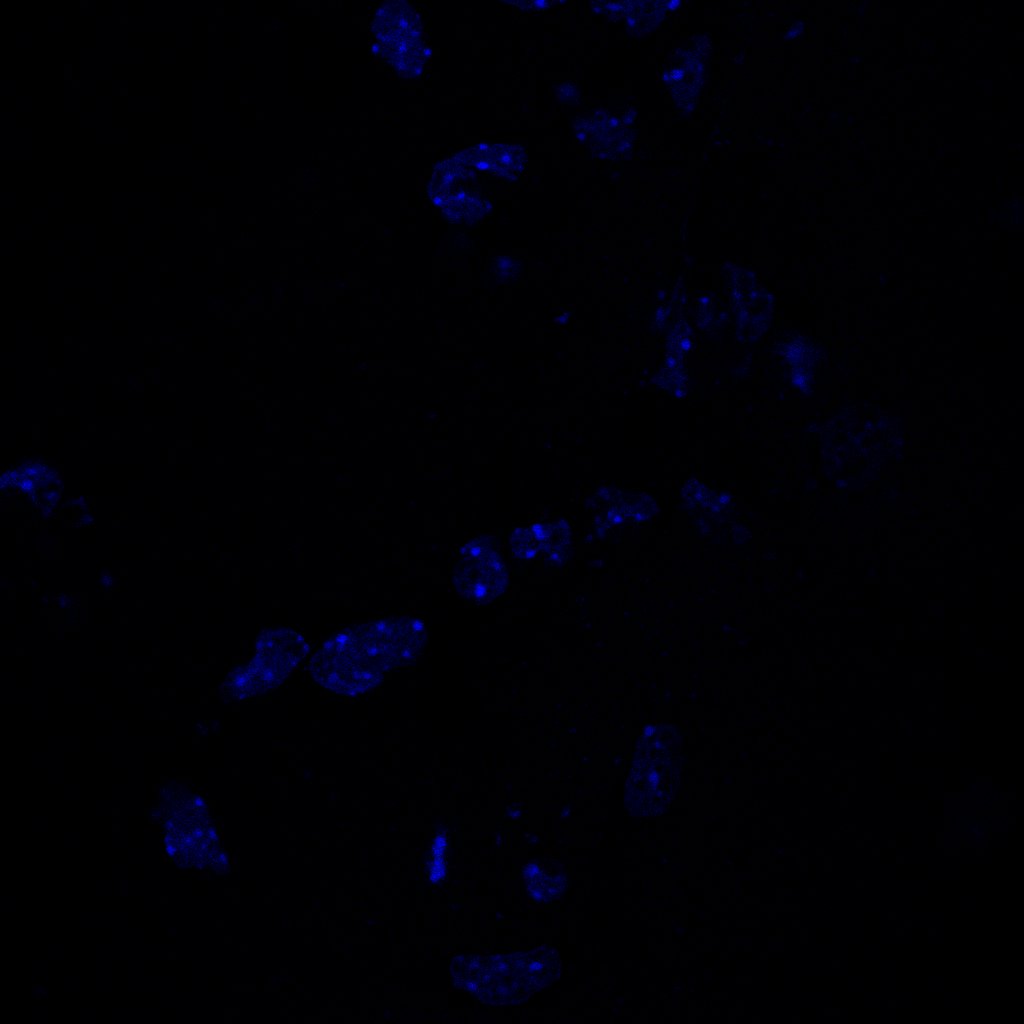

Supplement: Supplementary file 11 — Source Data Fig. 7 [file 44319_2024_64_MOESM11_ESM.zip › 7H/p75NTR WT/DAPI.jpg]

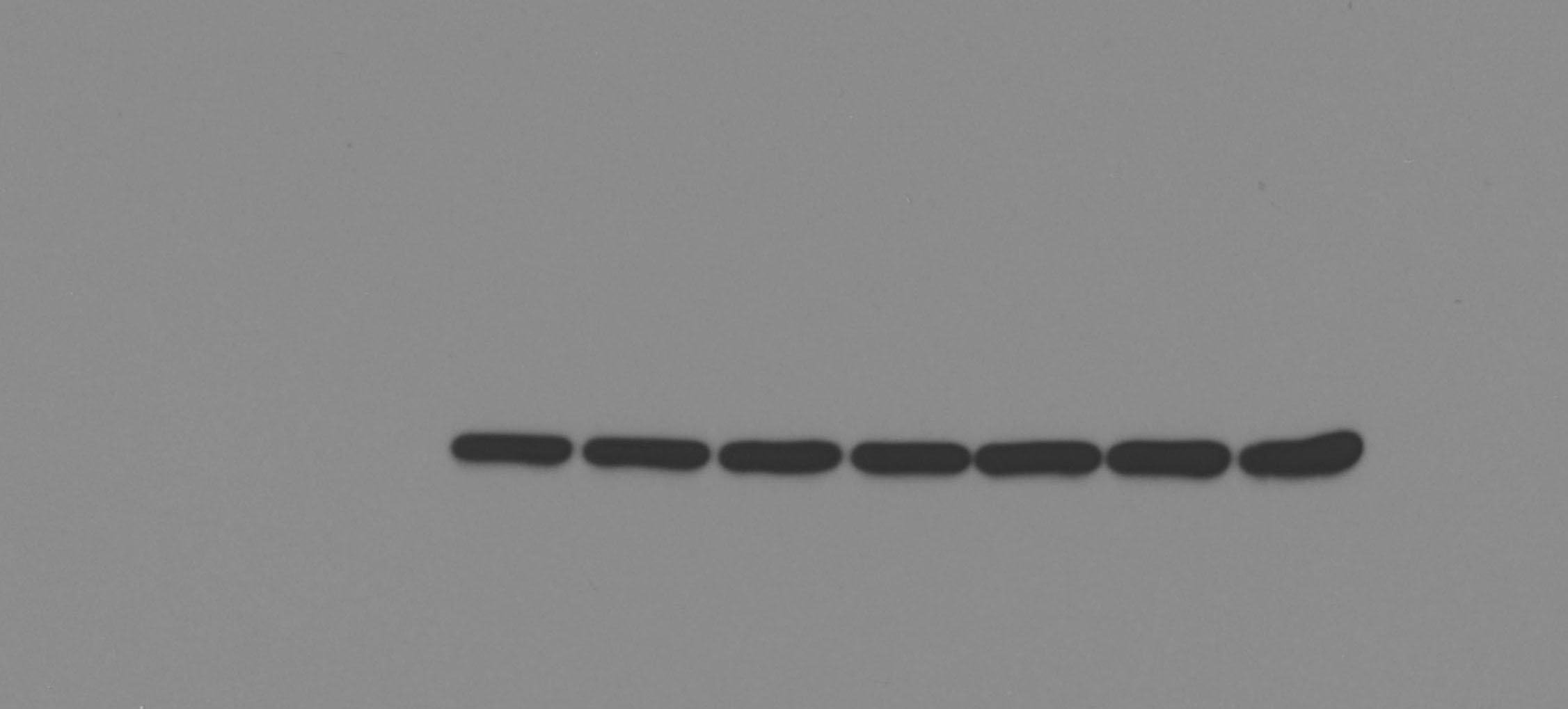

Supplement: Supplementary file 12 — Appendix Source Data [file 44319_2024_64_MOESM12_ESM.zip › Figure S5/5B/WCL IB Flag (RhoGDI).jpg]

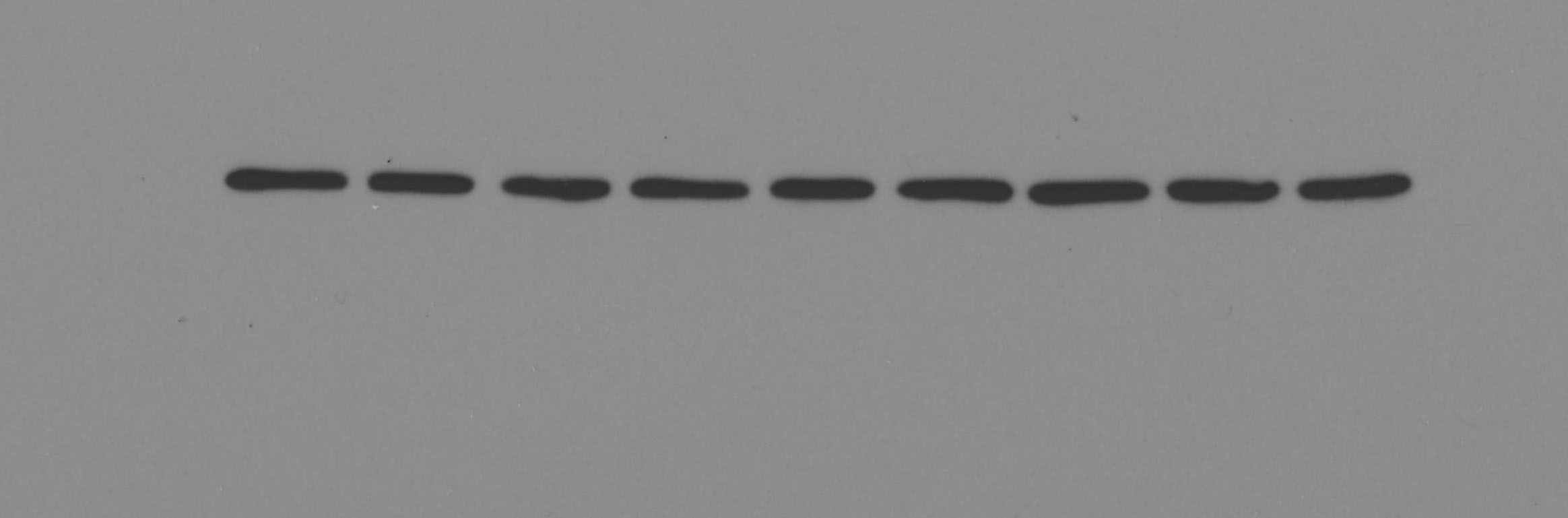

Supplement: Supplementary file 12 — Appendix Source Data [file 44319_2024_64_MOESM12_ESM.zip › Figure S5/5B/WCL IB GAPDH.jpg]

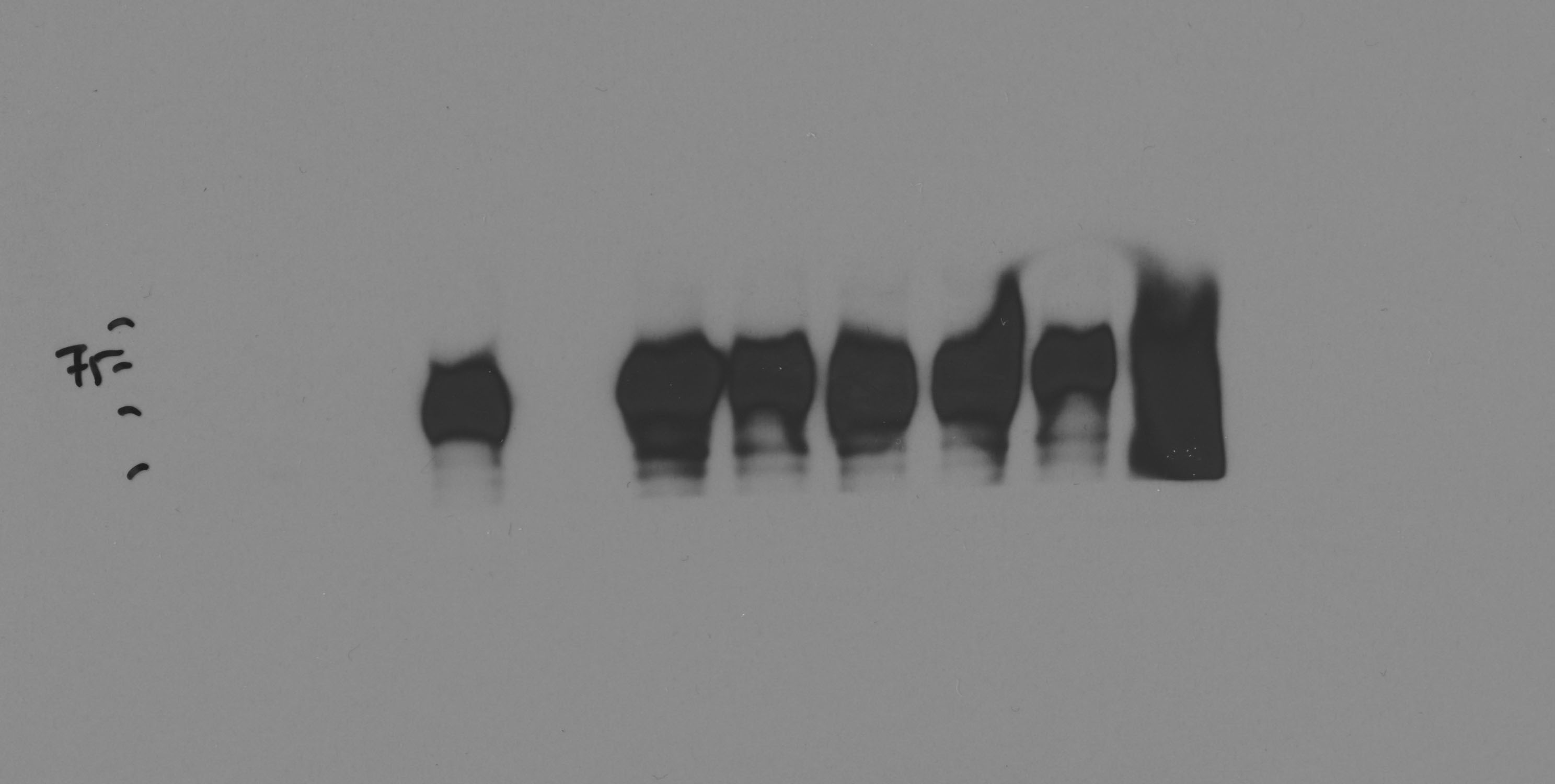

Supplement: Supplementary file 12 — Appendix Source Data [file 44319_2024_64_MOESM12_ESM.zip › Figure S5/5B/WCL IB p75NTR.jpg]

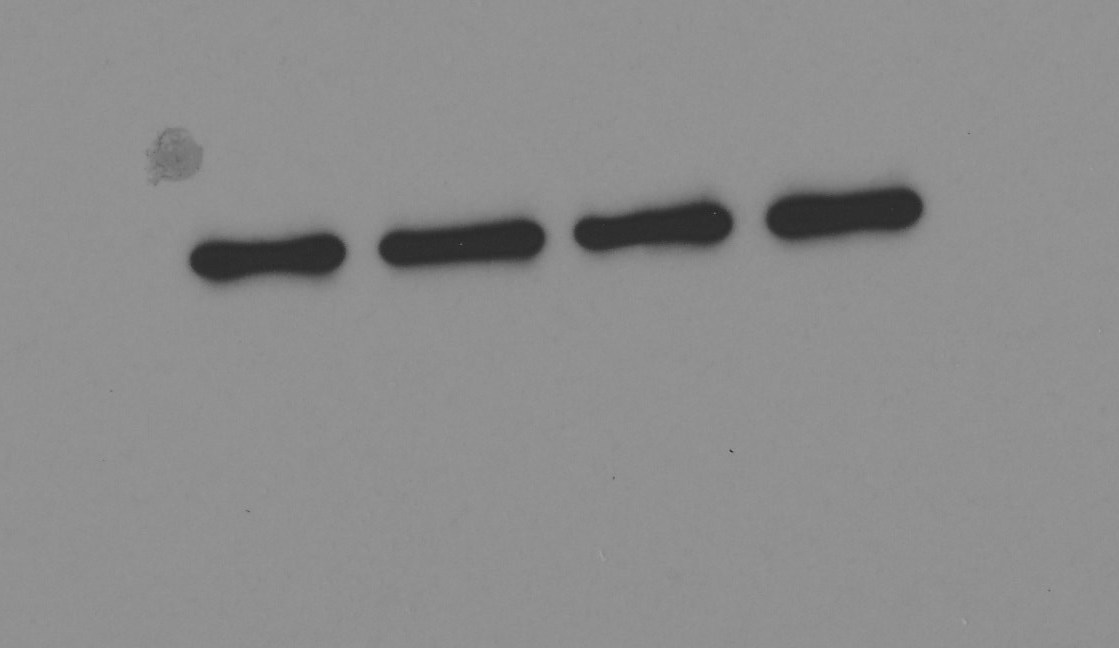

Supplement: Supplementary file 12 — Appendix Source Data [file 44319_2024_64_MOESM12_ESM.zip › Figure S5/5C/WCL IB GAPDH.jpg]

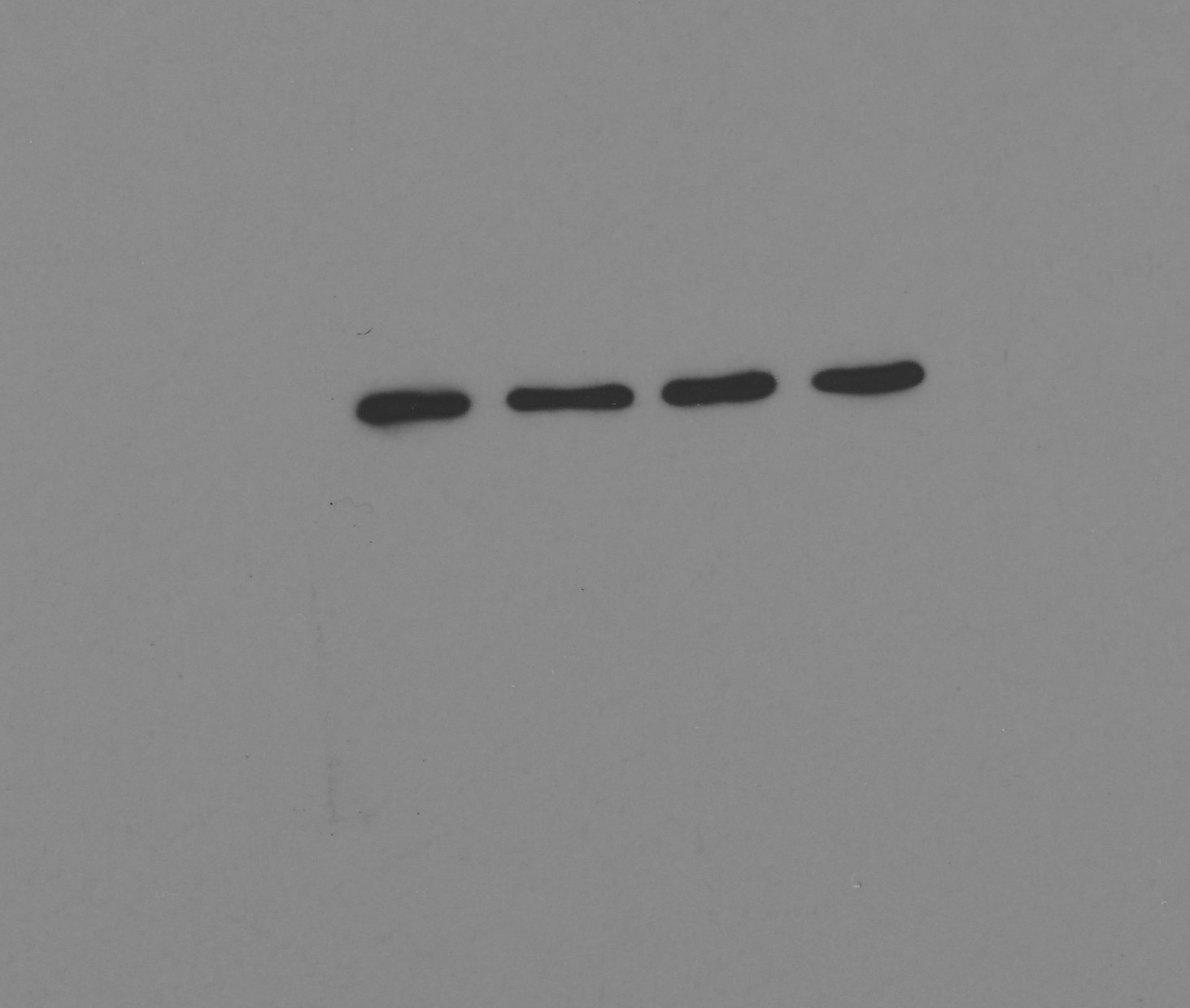

Supplement: Supplementary file 12 — Appendix Source Data [file 44319_2024_64_MOESM12_ESM.zip › Figure S5/5C/WCL IB RhoGDI.jpg]

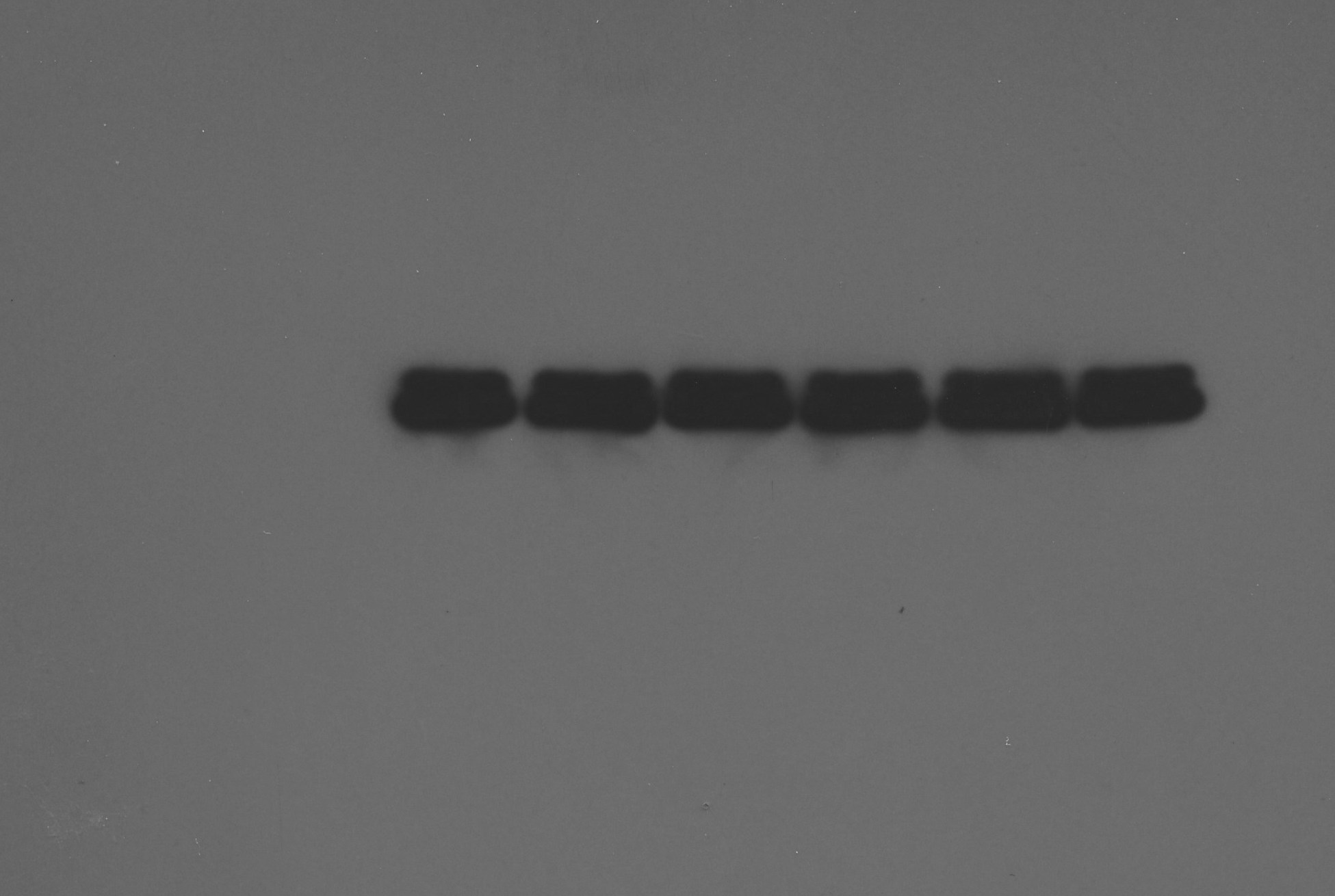

Supplement: Supplementary file 12 — Appendix Source Data [file 44319_2024_64_MOESM12_ESM.zip › Figure S5/5D/WCL IB Flag (RhoGDI).jpg]

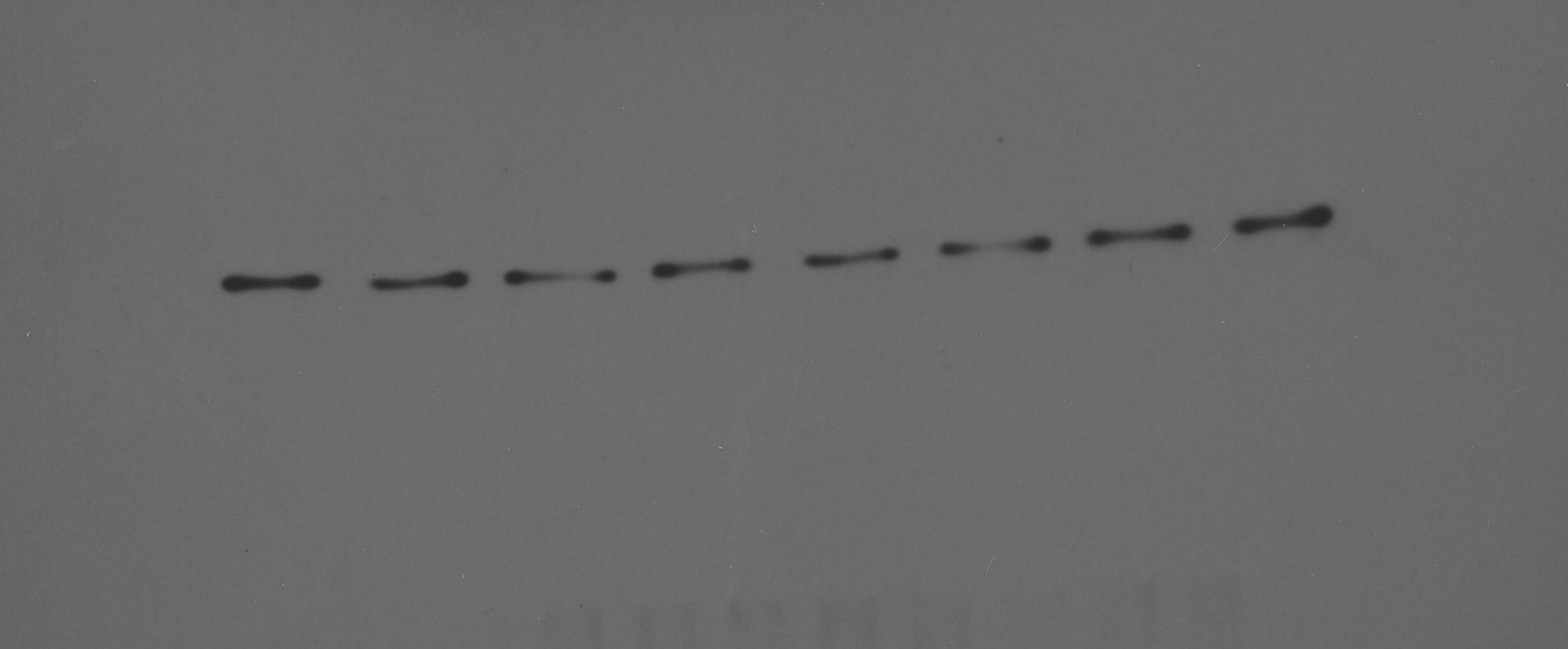

Supplement: Supplementary file 12 — Appendix Source Data [file 44319_2024_64_MOESM12_ESM.zip › Figure S5/5D/WCL IB GAPDH.jpg]

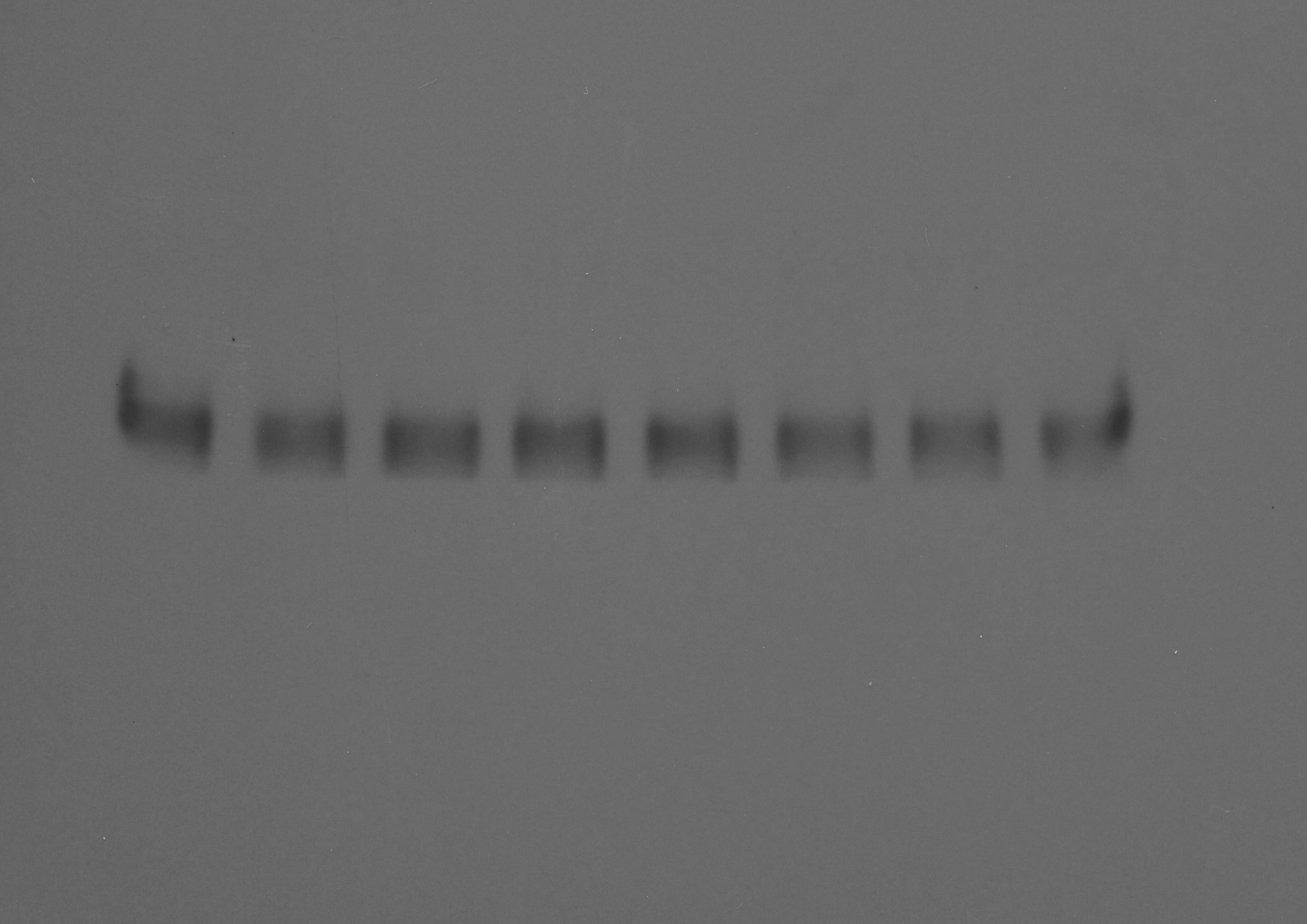

Supplement: Supplementary file 12 — Appendix Source Data [file 44319_2024_64_MOESM12_ESM.zip › Figure S5/5D/WCL IB p75NTR.jpg]

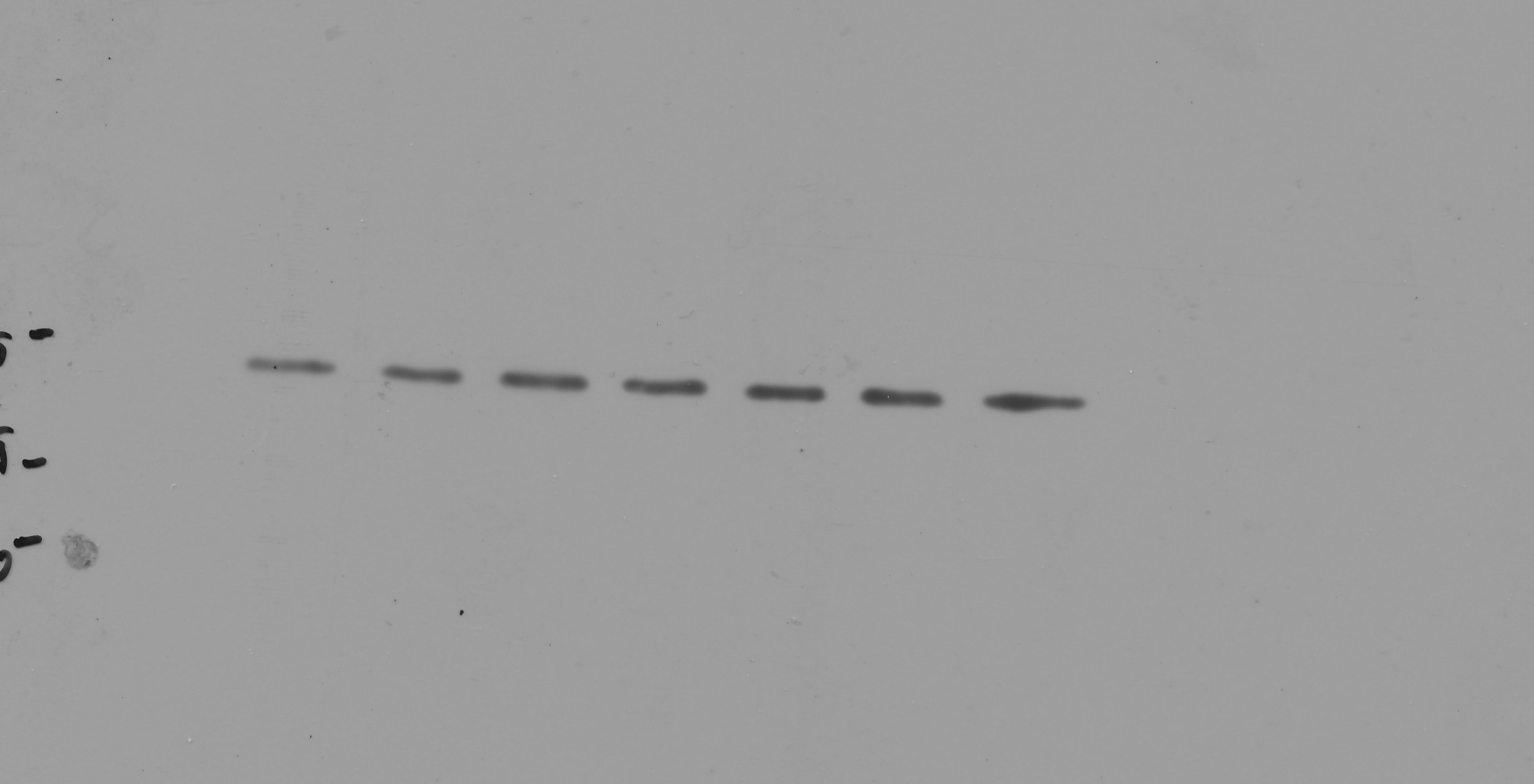

Supplement: Supplementary file 12 — Appendix Source Data [file 44319_2024_64_MOESM12_ESM.zip › Figure S5/5E/WCL IB GAPDH.jpg]

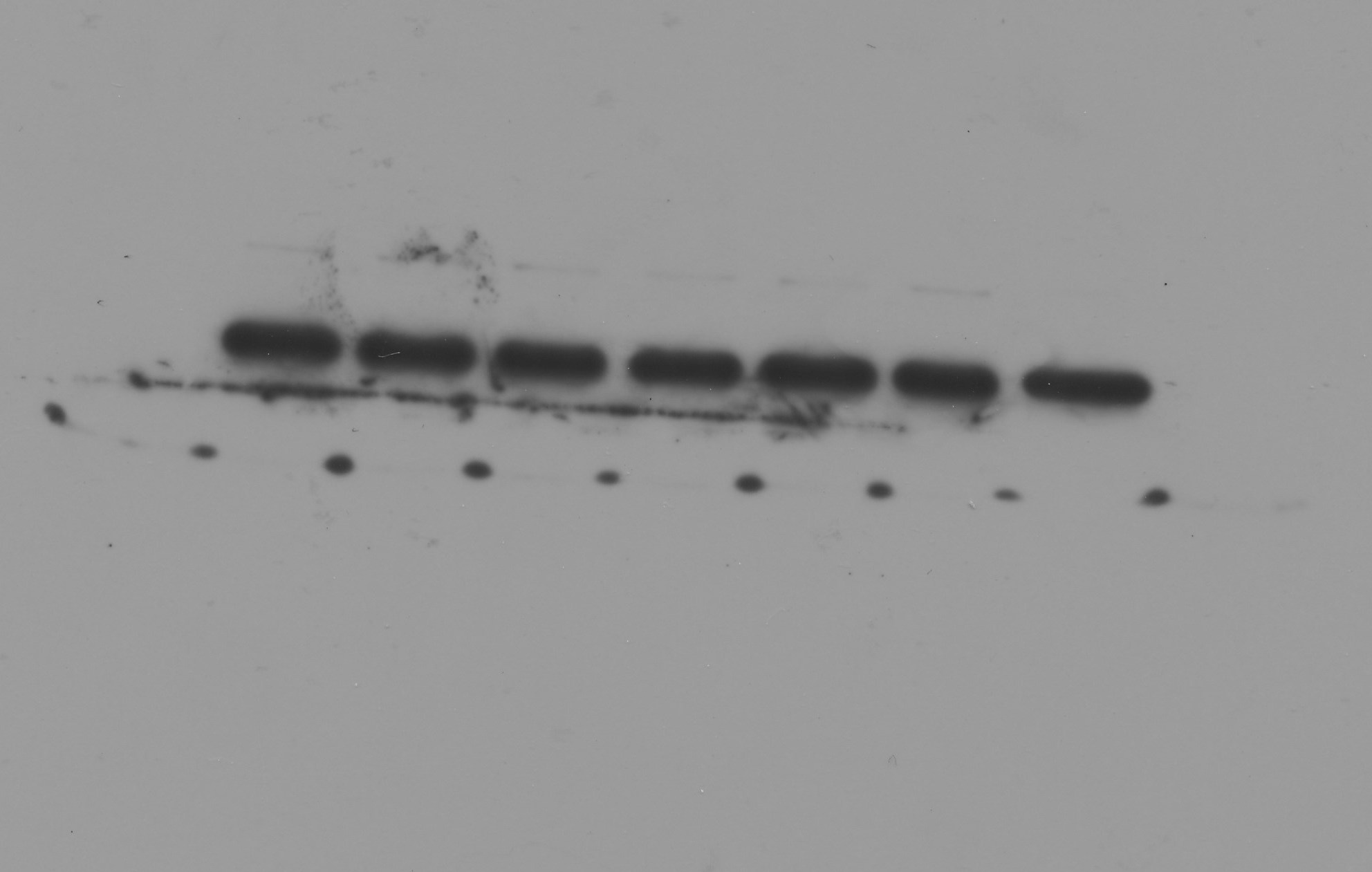

Supplement: Supplementary file 12 — Appendix Source Data [file 44319_2024_64_MOESM12_ESM.zip › Figure S5/5E/WCL IB RhoGDI.jpg]

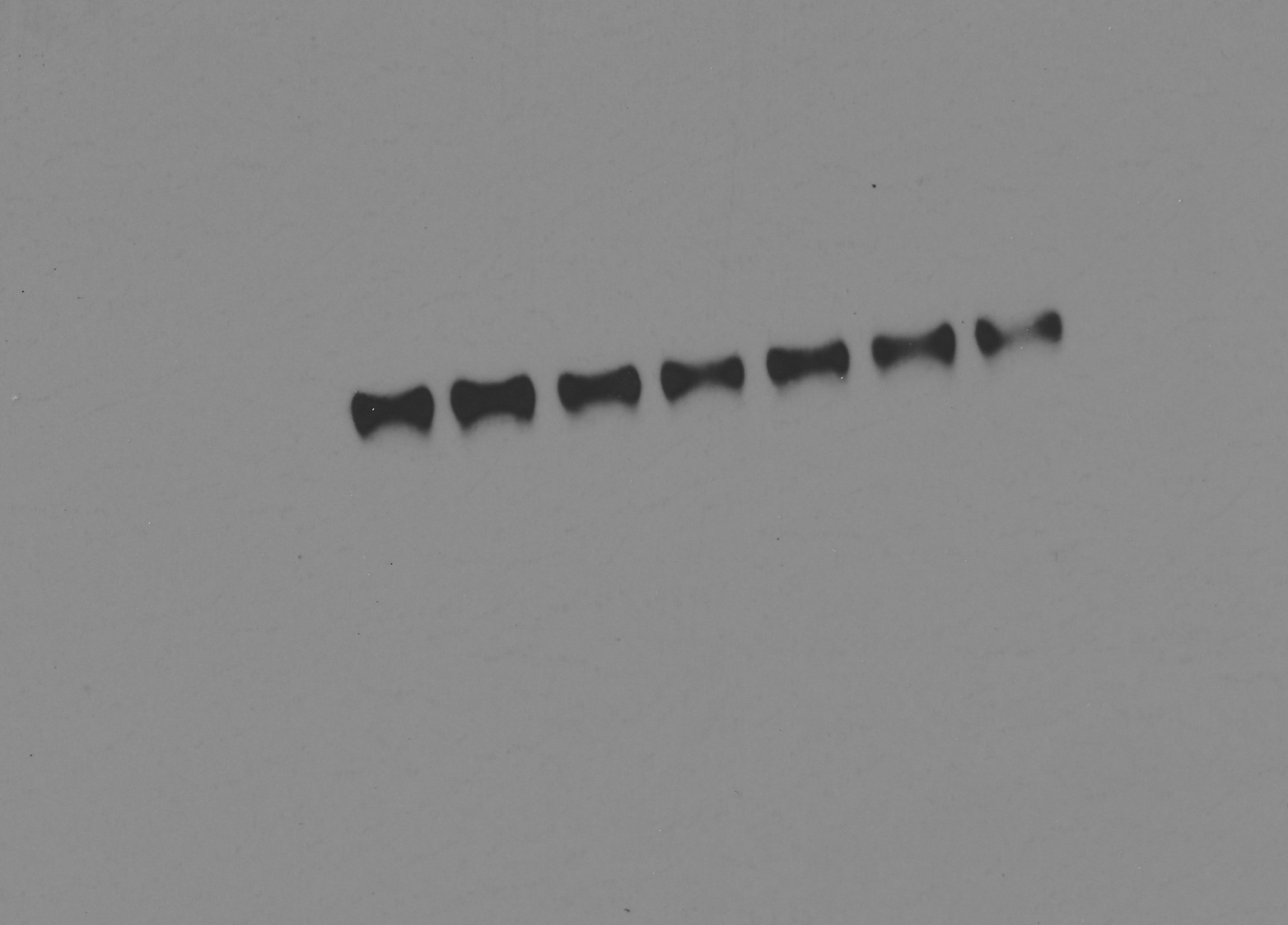

Supplement: Supplementary file 12 — Appendix Source Data [file 44319_2024_64_MOESM12_ESM.zip › Figure S5/5F/WCL IB Flag (RhoGDI).jpg]

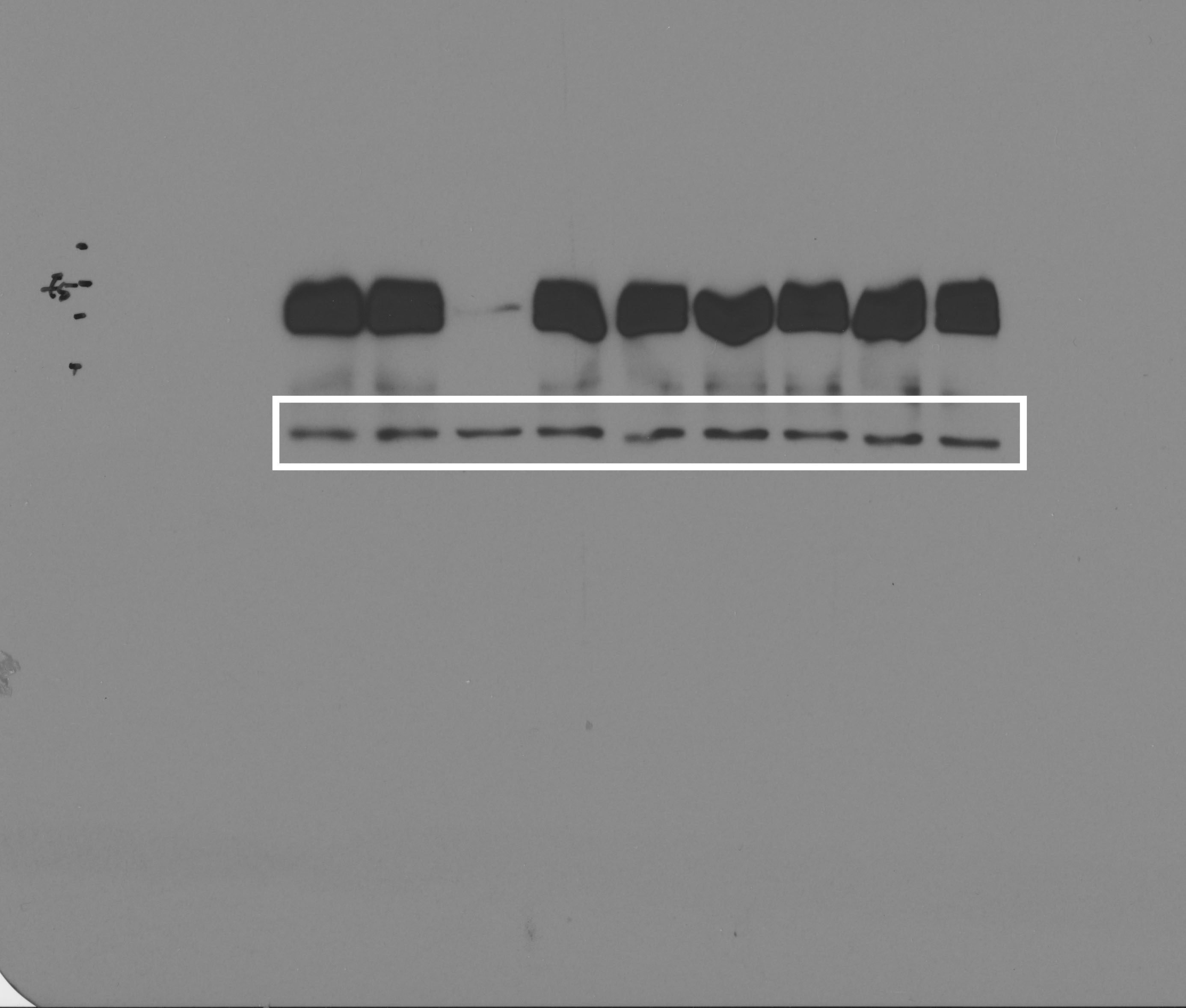

Supplement: Supplementary file 12 — Appendix Source Data [file 44319_2024_64_MOESM12_ESM.zip › Figure S5/5F/WCL IB GAPDH.jpg]

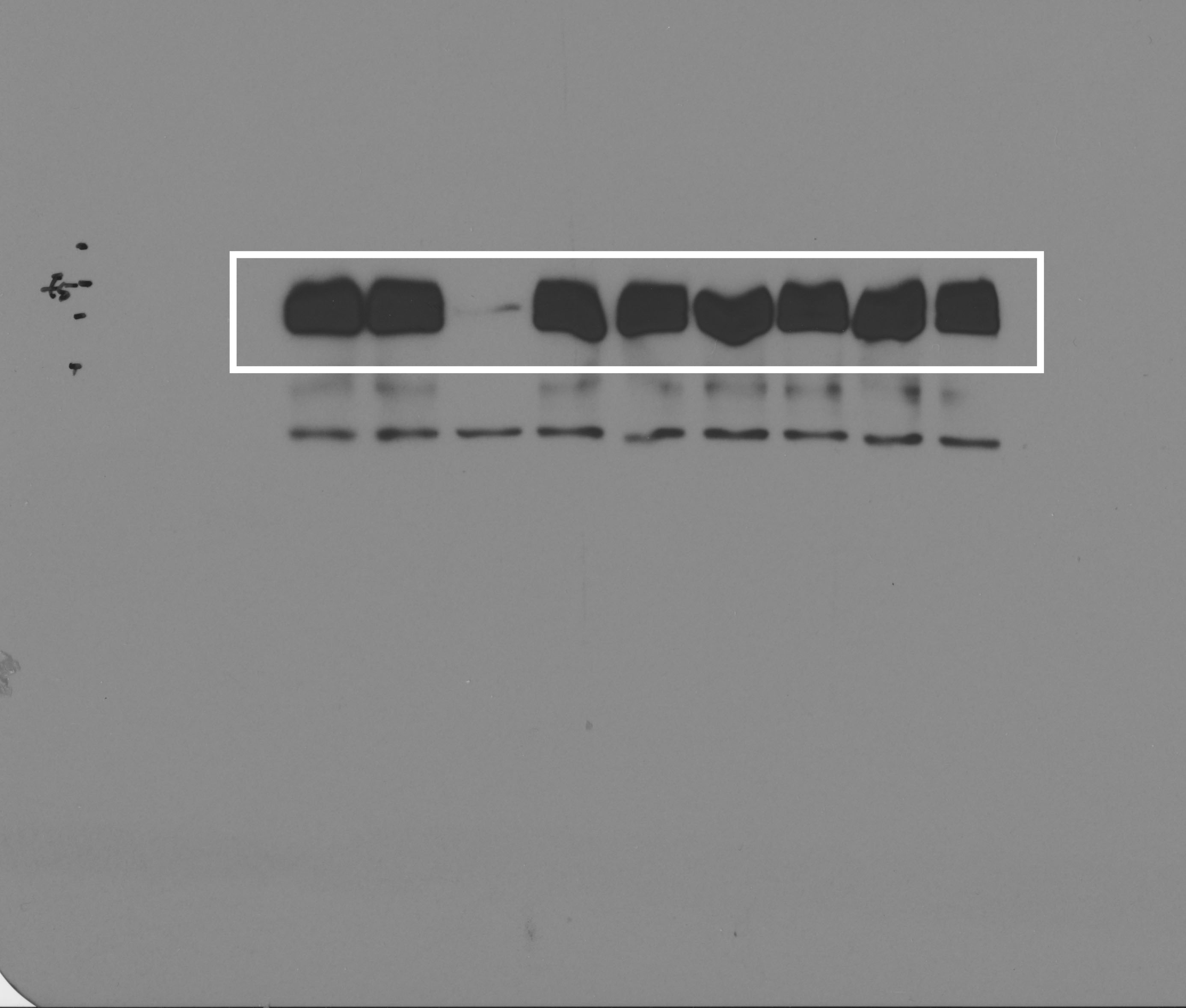

Supplement: Supplementary file 12 — Appendix Source Data [file 44319_2024_64_MOESM12_ESM.zip › Figure S5/5F/WCL IB p75NTR.jpg]

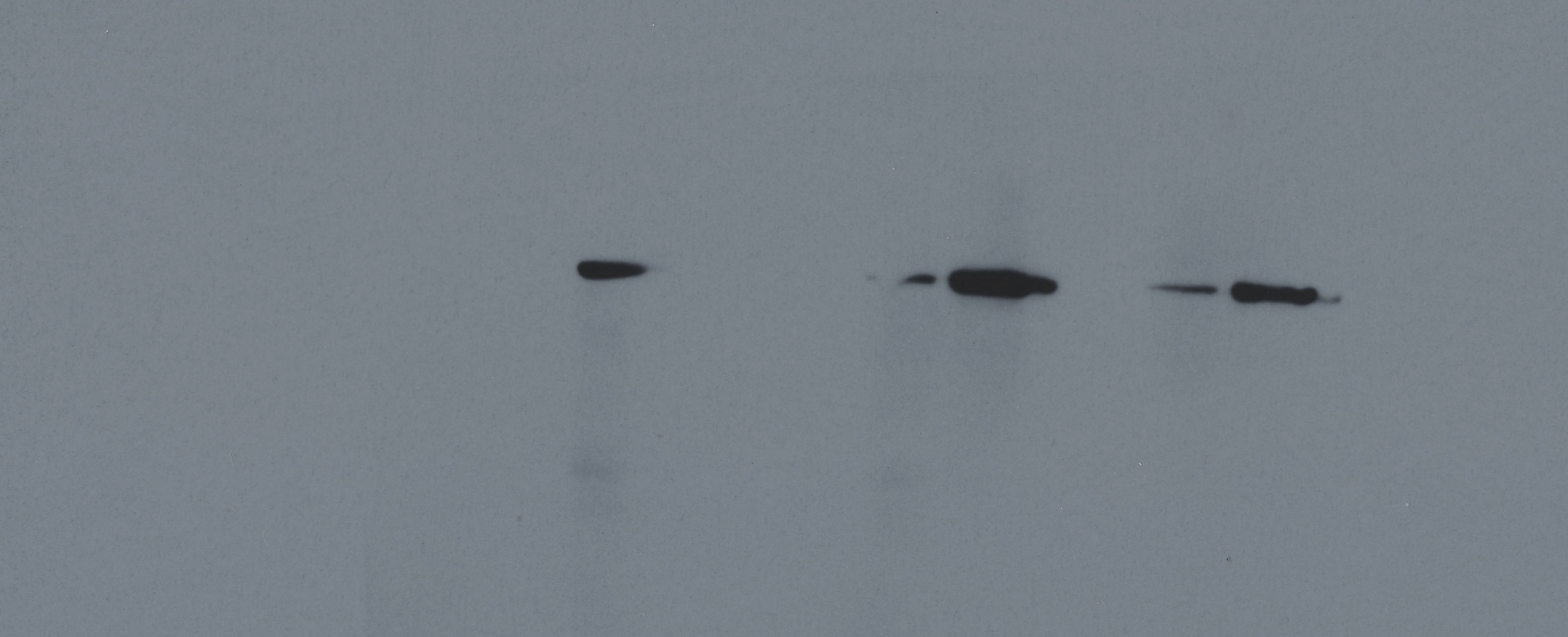

Supplement: Supplementary file 12 — Appendix Source Data [file 44319_2024_64_MOESM12_ESM.zip › Figure S5/5F/WCL IB RIP2.jpg]

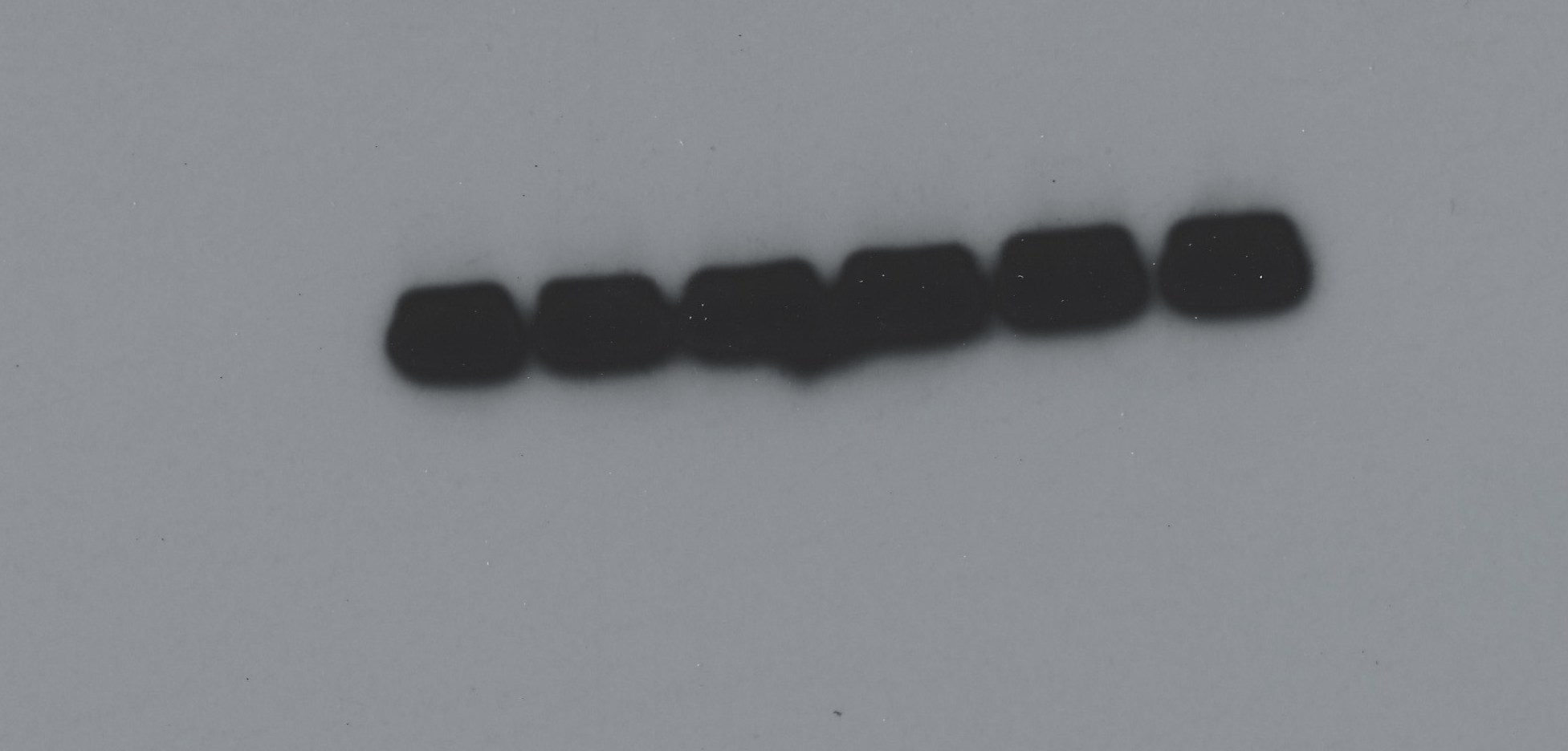

Supplement: Supplementary file 12 — Appendix Source Data [file 44319_2024_64_MOESM12_ESM.zip › Figure S5/5G/WCL IB Flag (RhoGDI).jpg]

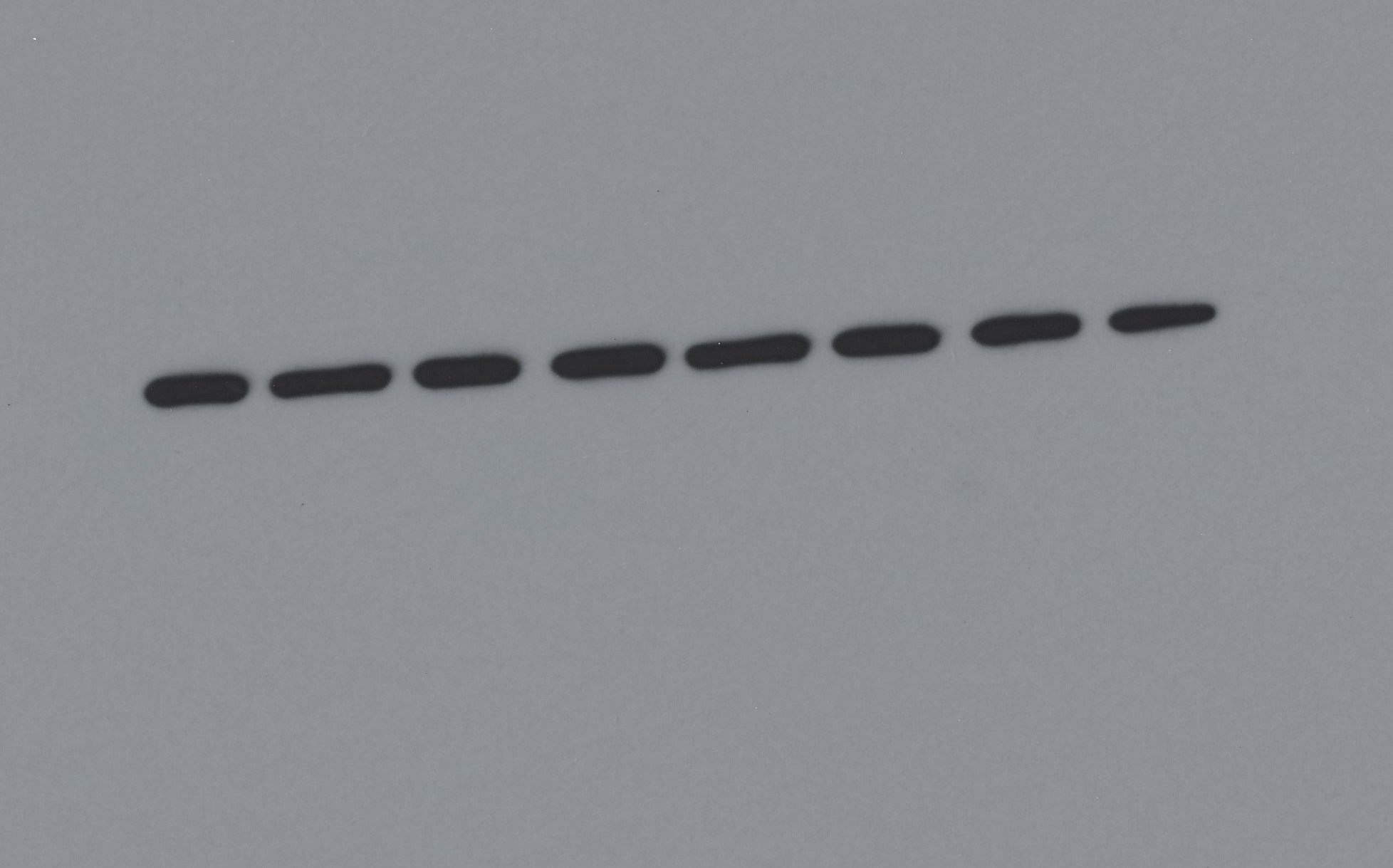

Supplement: Supplementary file 12 — Appendix Source Data [file 44319_2024_64_MOESM12_ESM.zip › Figure S5/5G/WCL IB GAPDH.jpg]

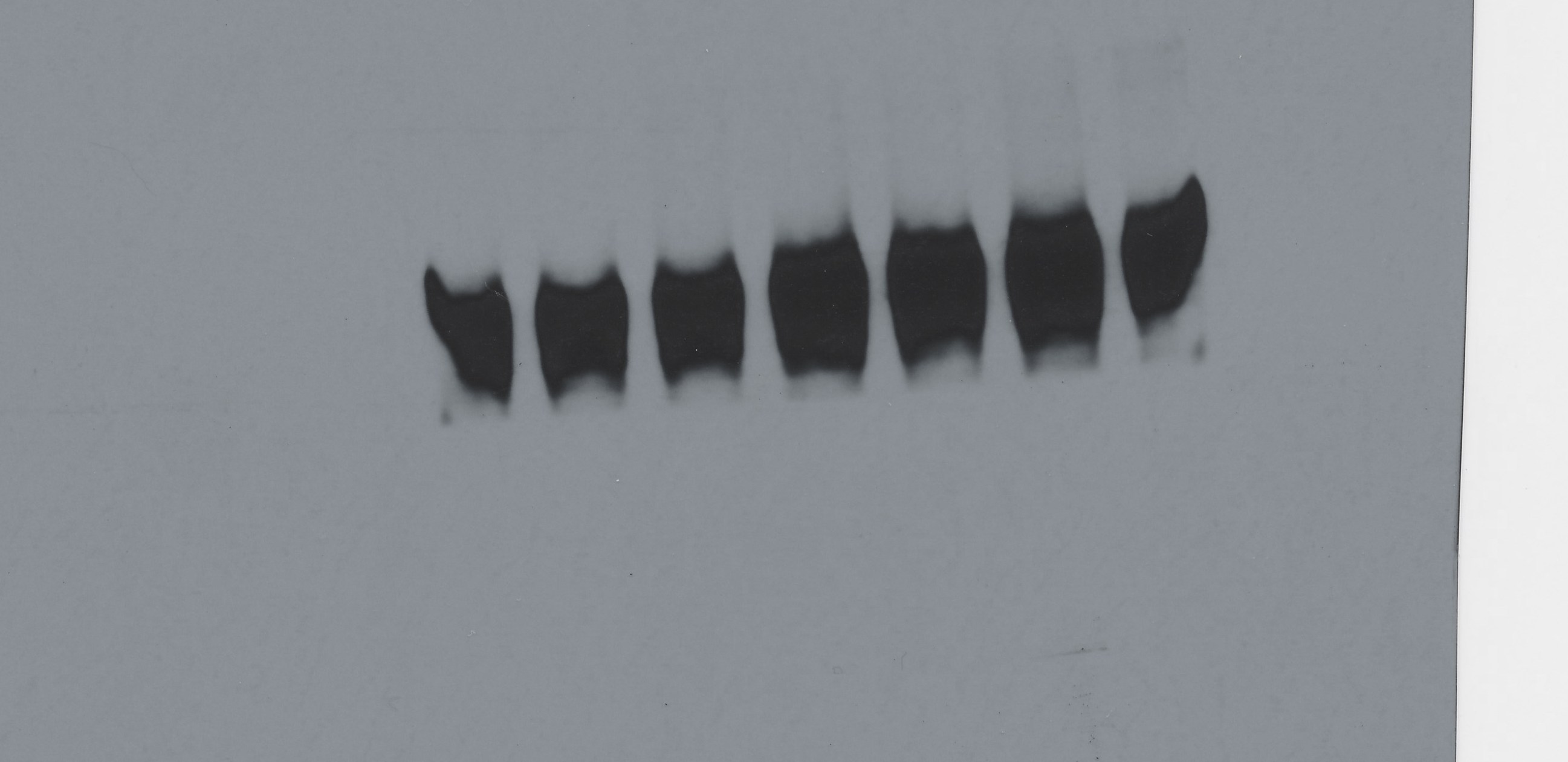

Supplement: Supplementary file 12 — Appendix Source Data [file 44319_2024_64_MOESM12_ESM.zip › Figure S5/5G/WCL IB p75NTR.jpg]

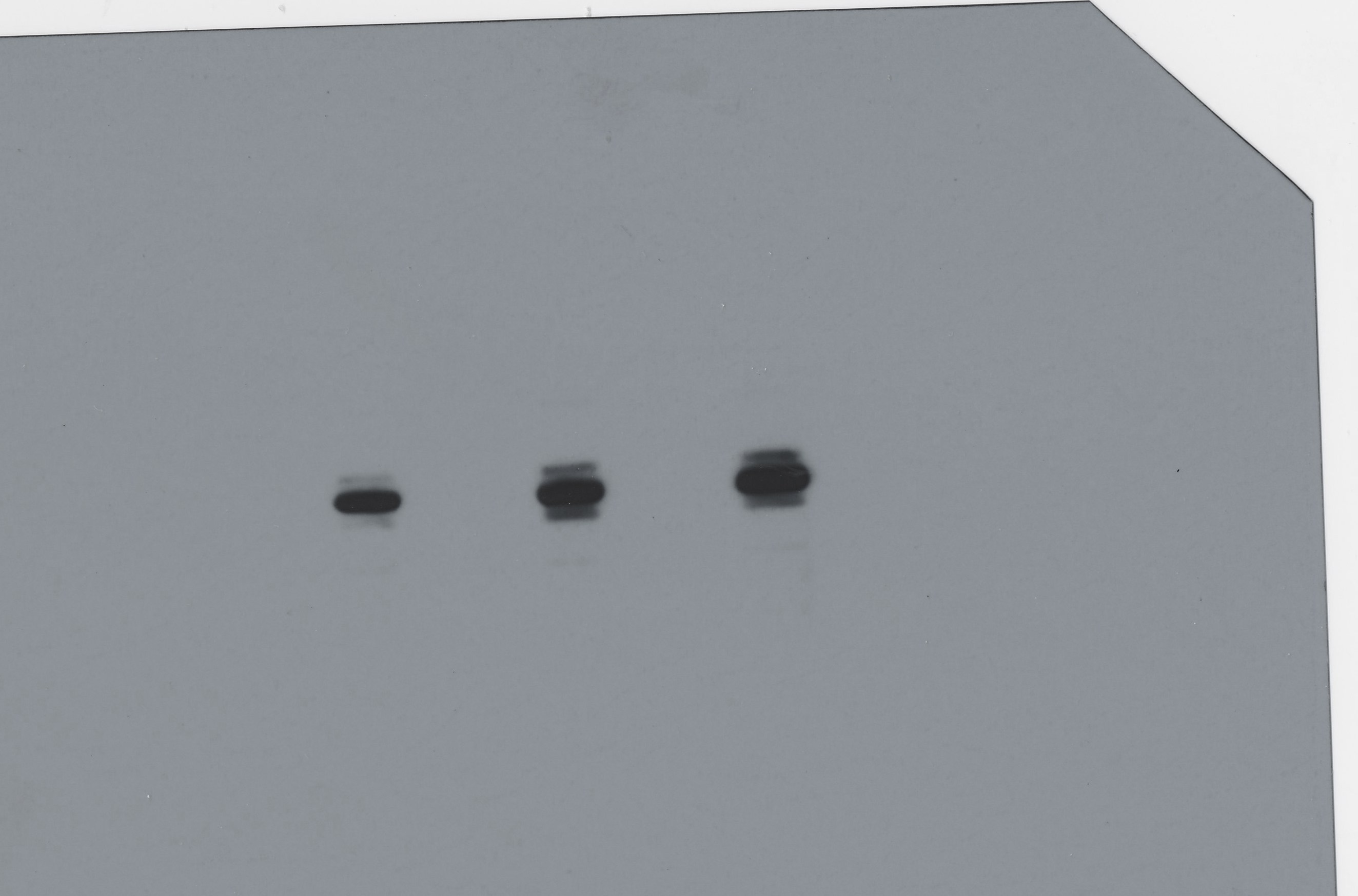

Supplement: Supplementary file 12 — Appendix Source Data [file 44319_2024_64_MOESM12_ESM.zip › Figure S5/5G/WCL IB RIP2.jpg]

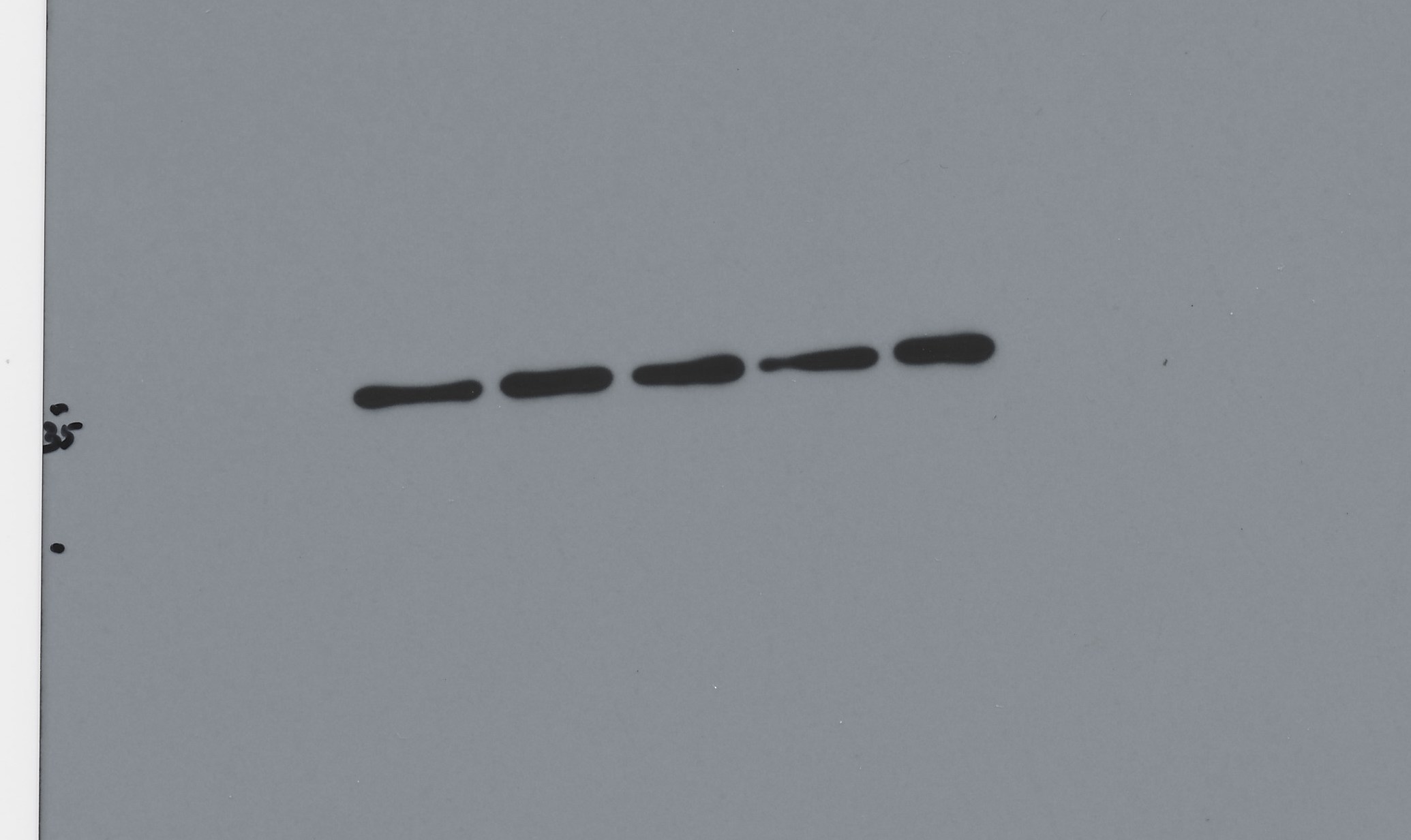

Supplement: Supplementary file 12 — Appendix Source Data [file 44319_2024_64_MOESM12_ESM.zip › Figure S5/5H/WCL IB GAPDH.jpg]

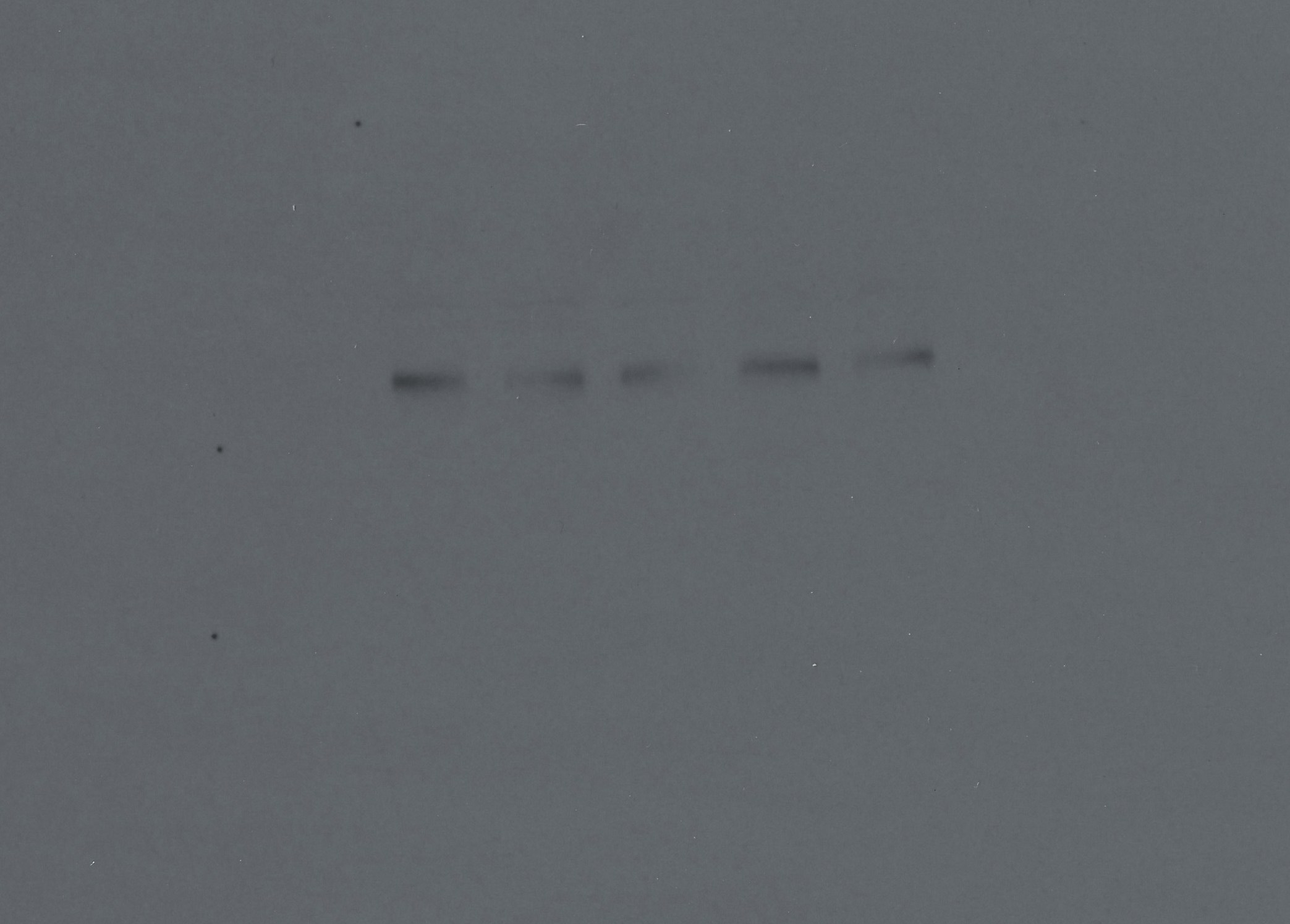

Supplement: Supplementary file 12 — Appendix Source Data [file 44319_2024_64_MOESM12_ESM.zip › Figure S5/5H/WCL IB p75NTR.jpg]

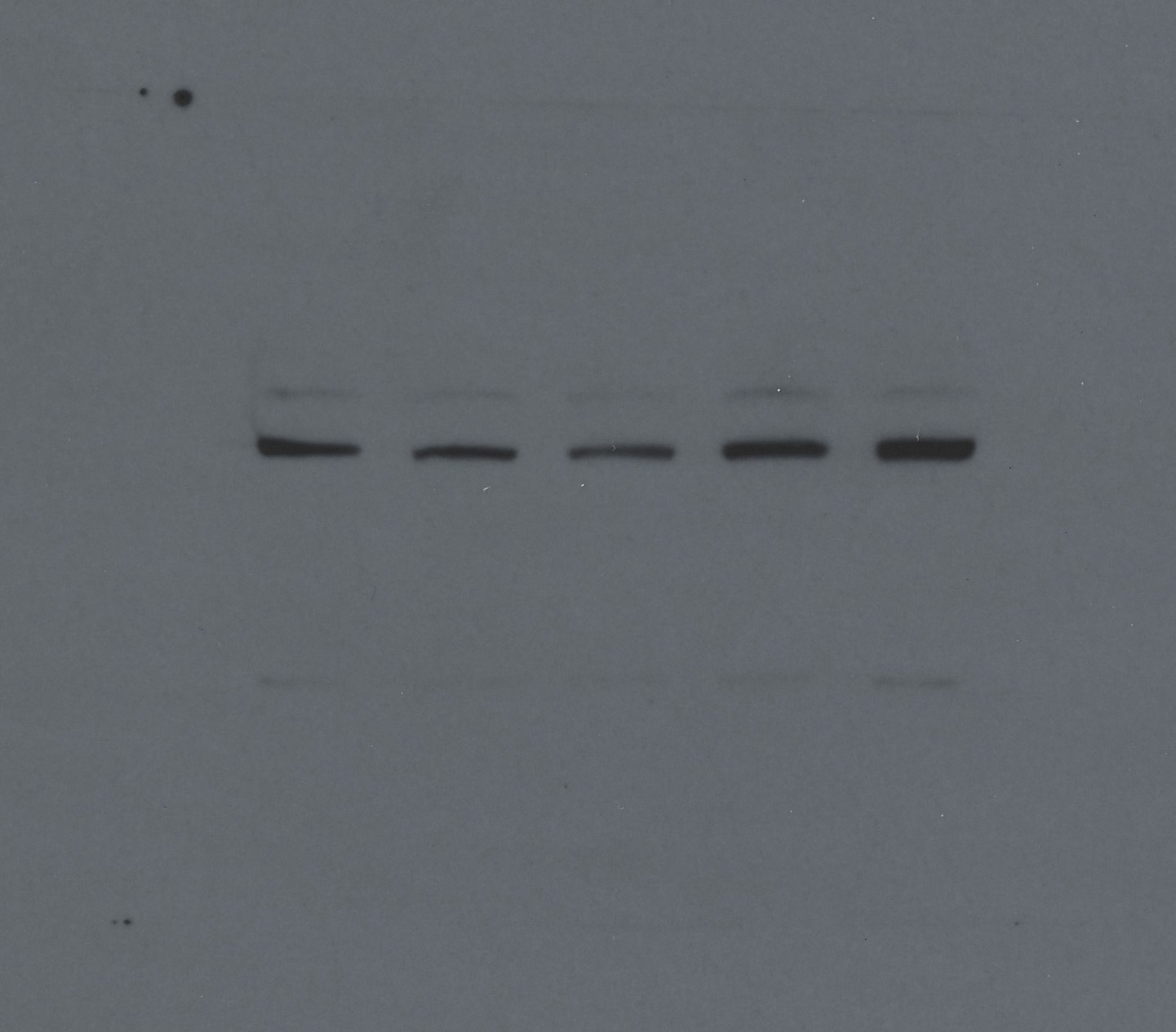

Supplement: Supplementary file 12 — Appendix Source Data [file 44319_2024_64_MOESM12_ESM.zip › Figure S5/5H/WCL IB RIP2.jpg]

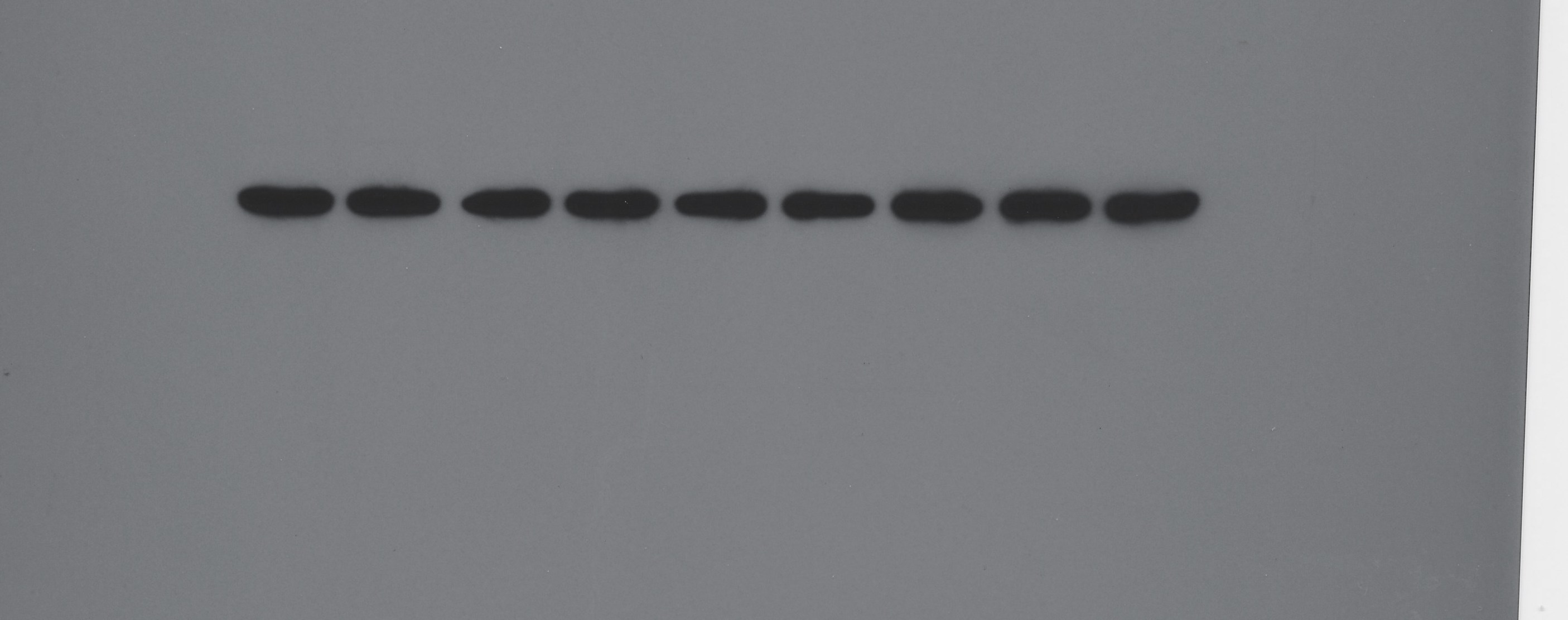

Supplement: Supplementary file 12 — Appendix Source Data [file 44319_2024_64_MOESM12_ESM.zip › Figure S5/5I/WCL IB GAPDH.jpg]

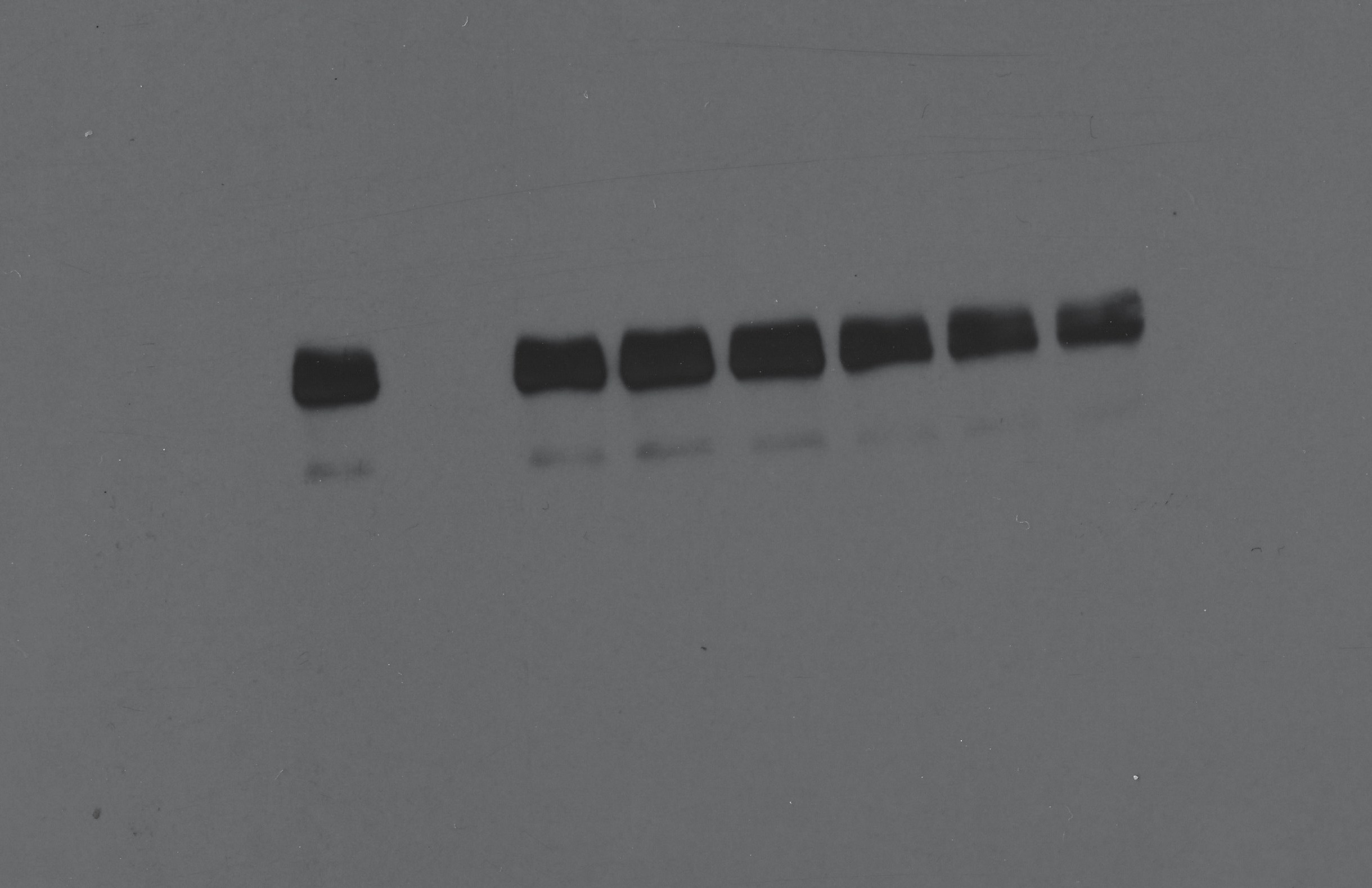

Supplement: Supplementary file 12 — Appendix Source Data [file 44319_2024_64_MOESM12_ESM.zip › Figure S5/5I/WCL IB p75NTR.jpg]

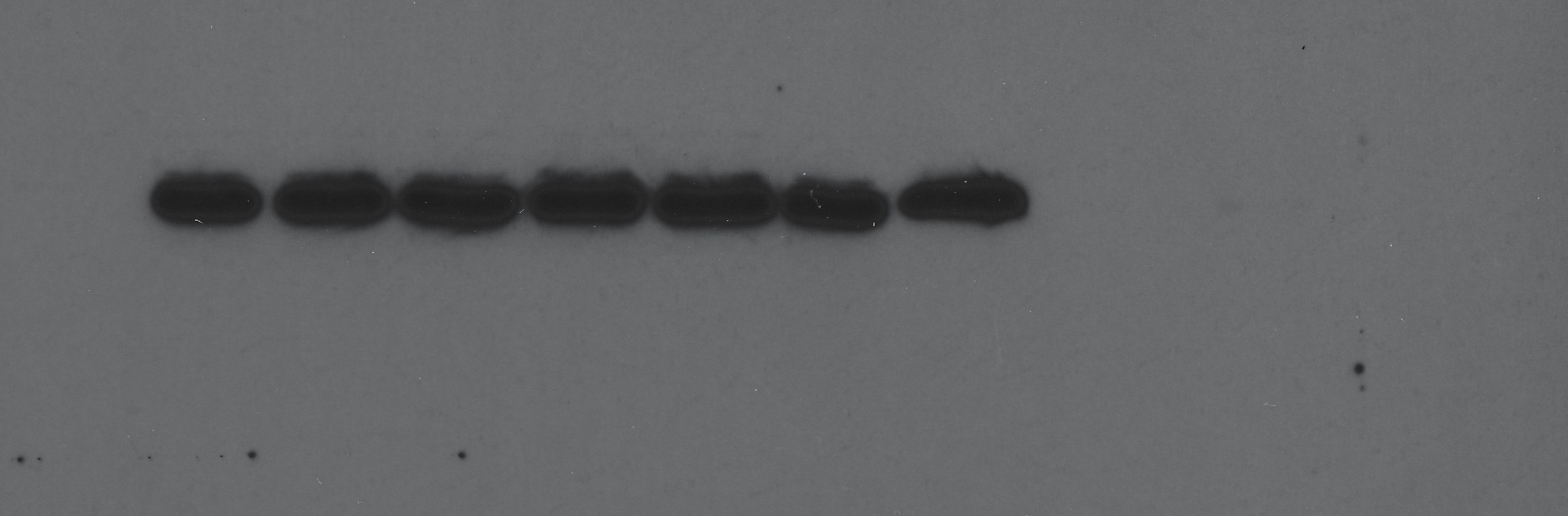

Supplement: Supplementary file 12 — Appendix Source Data [file 44319_2024_64_MOESM12_ESM.zip › Figure S5/5I/WCL IB RhoGDI.jpg]

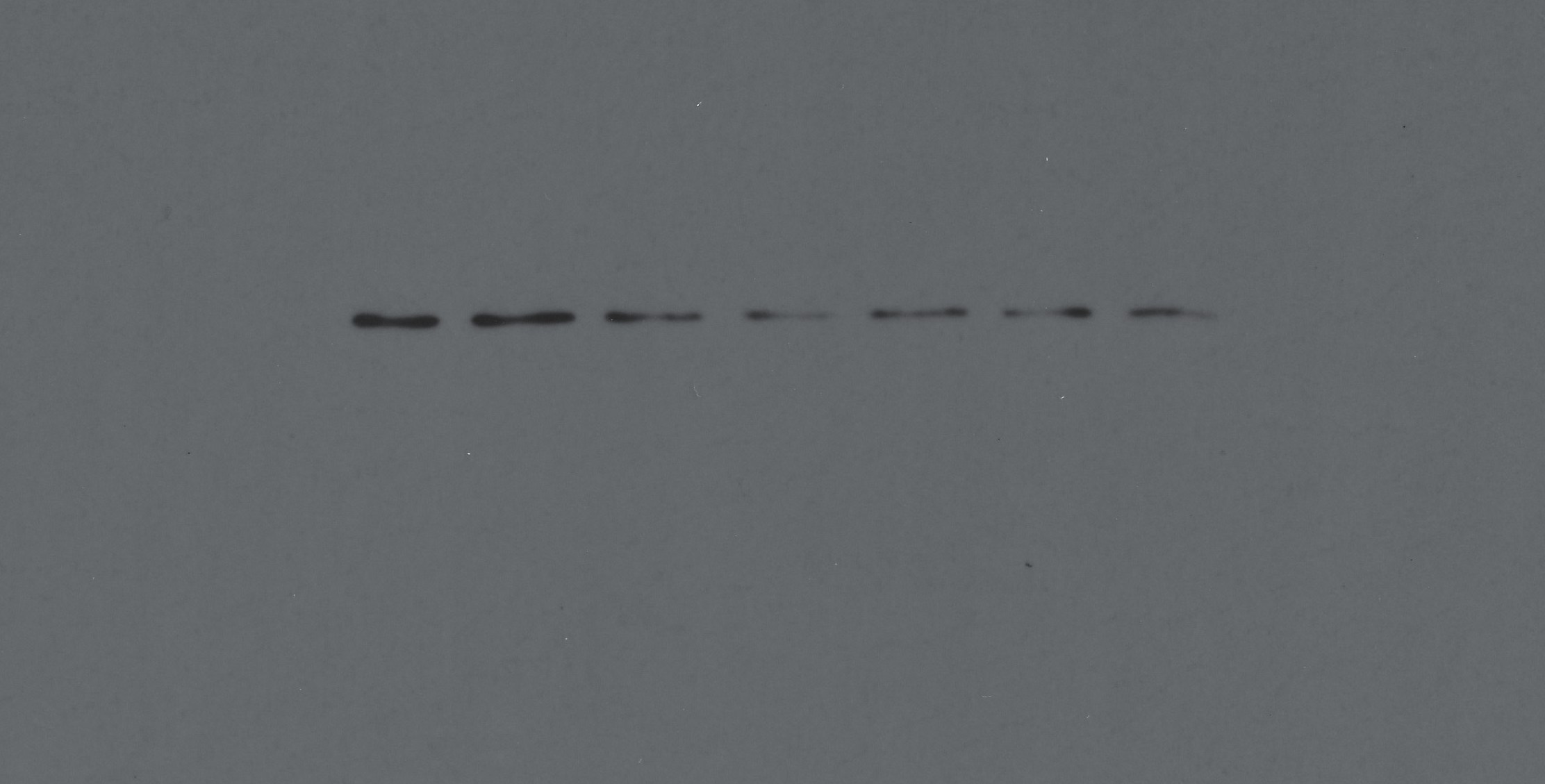

Supplement: Supplementary file 12 — Appendix Source Data [file 44319_2024_64_MOESM12_ESM.zip › Figure S5/5I/WCL IB RIP2.jpg]

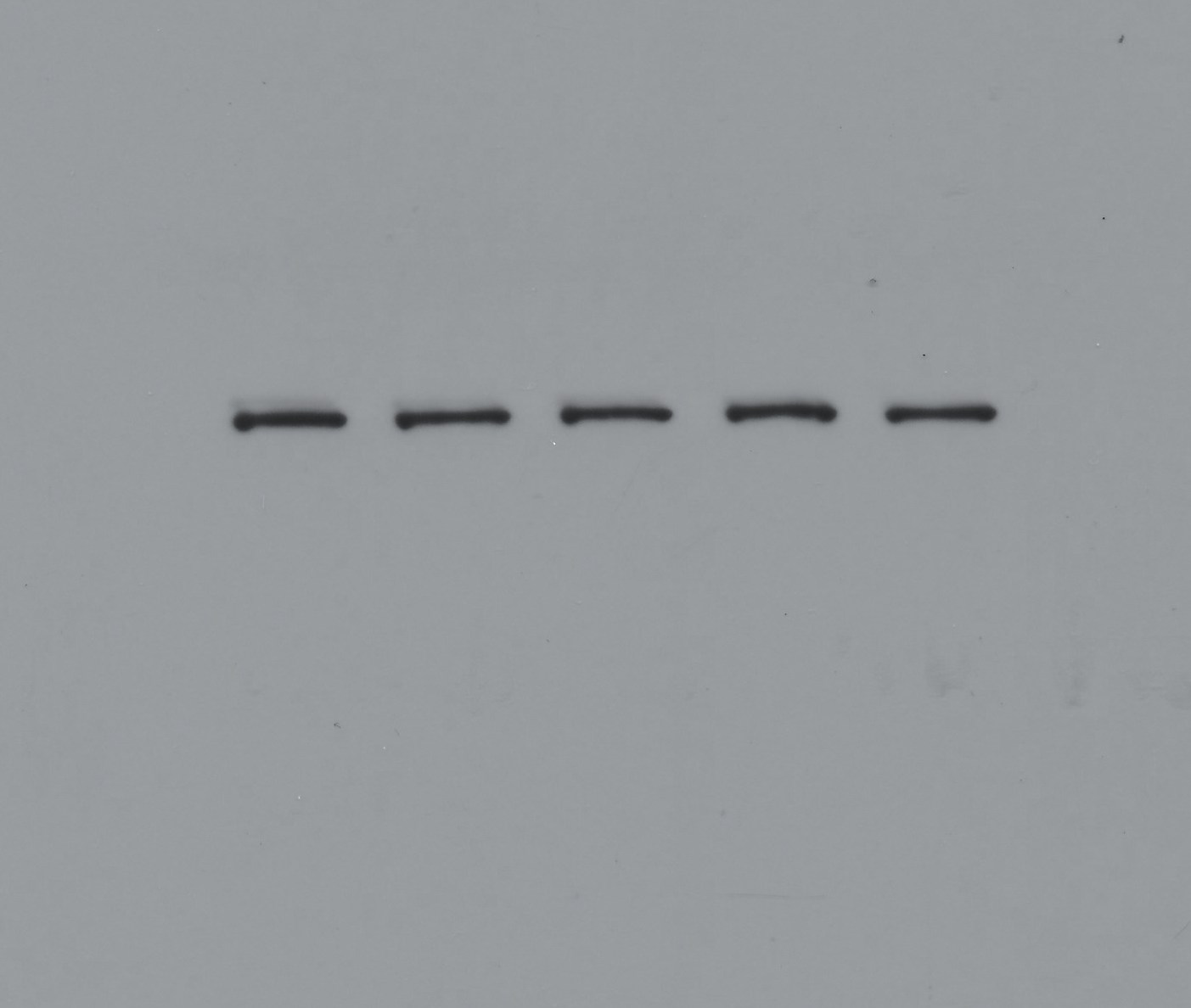

Supplement: Supplementary file 12 — Appendix Source Data [file 44319_2024_64_MOESM12_ESM.zip › Figure S5/5J/WCL IB GAPDH.jpg]

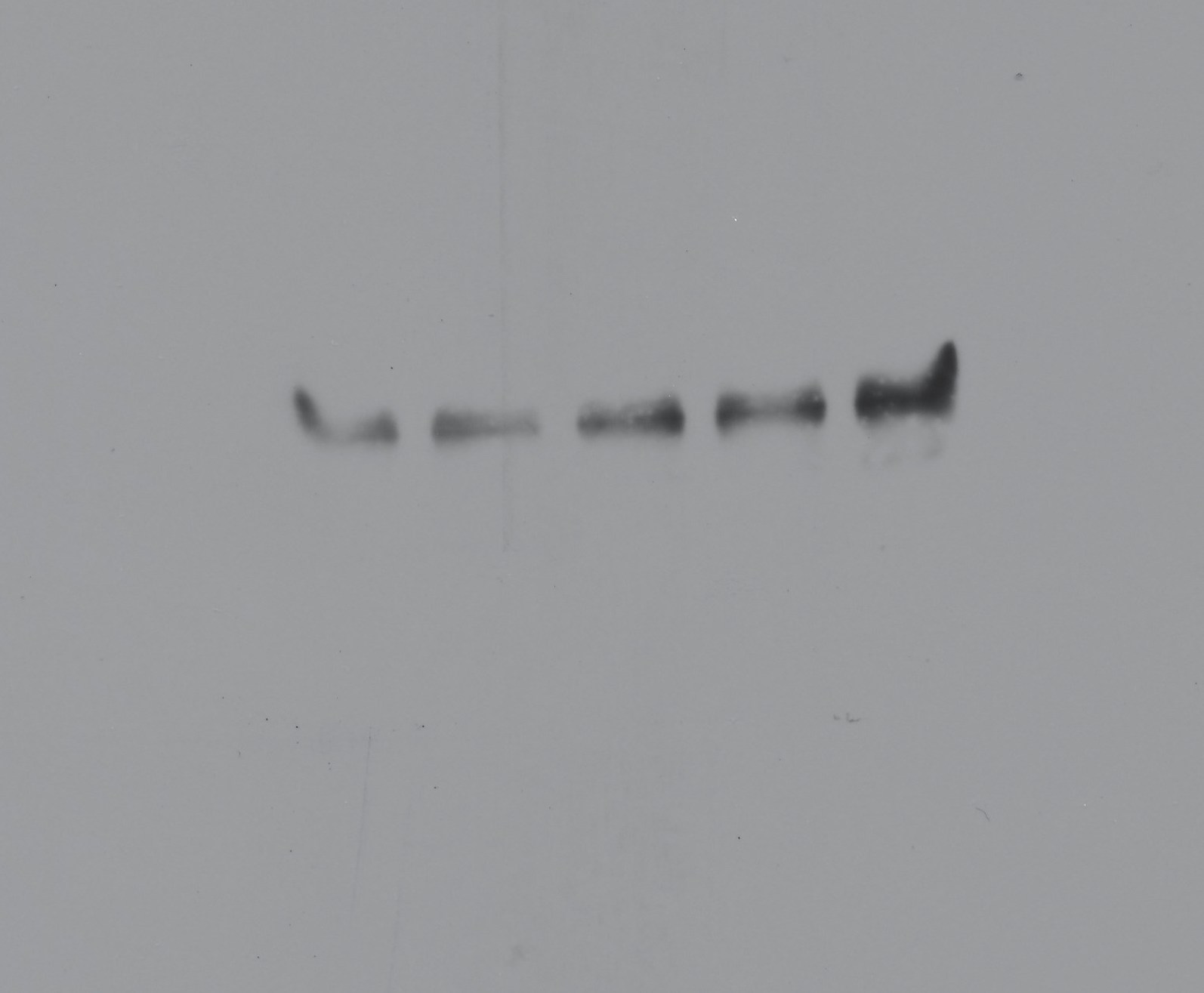

Supplement: Supplementary file 12 — Appendix Source Data [file 44319_2024_64_MOESM12_ESM.zip › Figure S5/5J/WCL IB p75NTR.jpg]

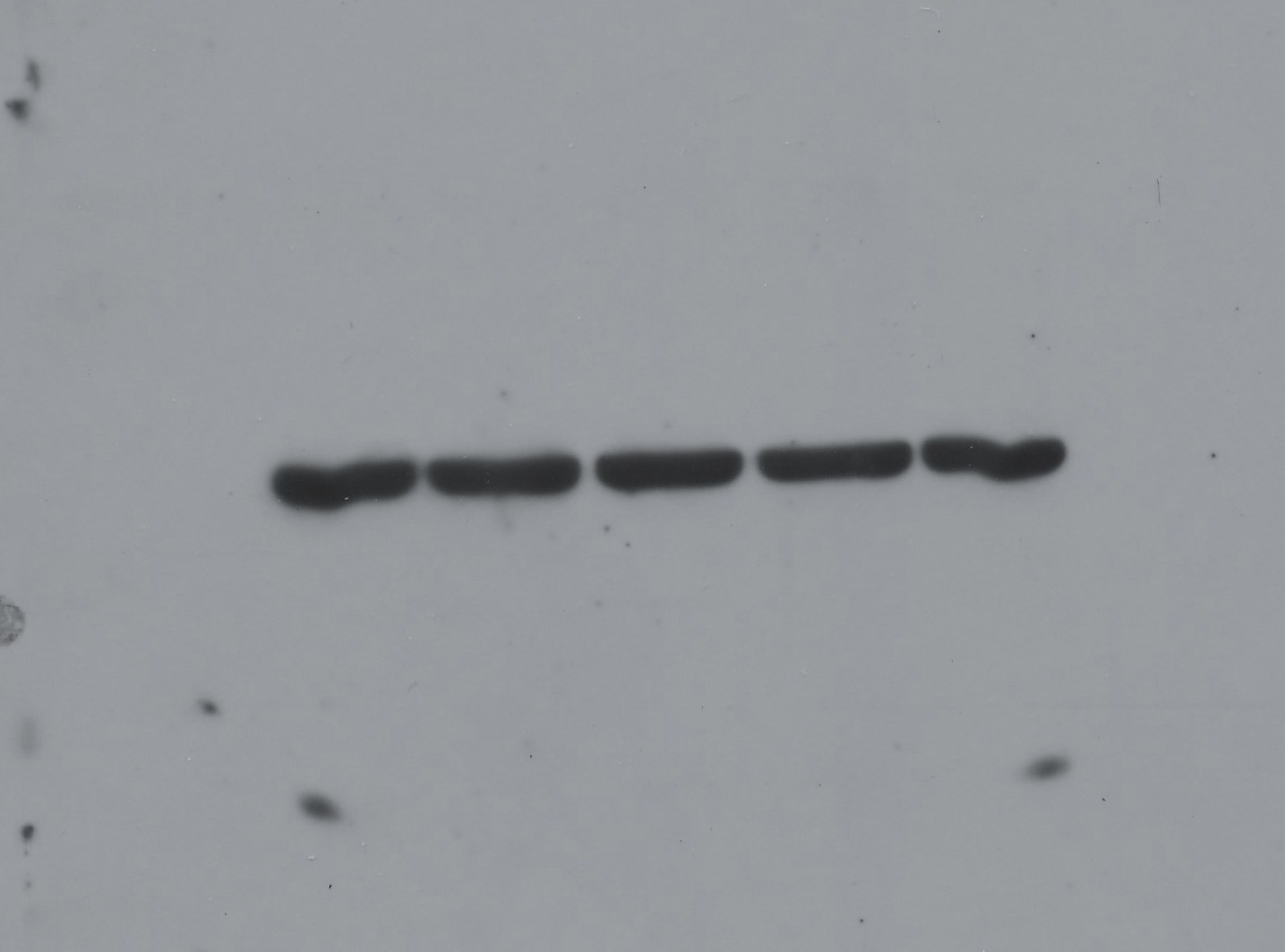

Supplement: Supplementary file 12 — Appendix Source Data [file 44319_2024_64_MOESM12_ESM.zip › Figure S5/5J/WCL IB RIP2.jpg]

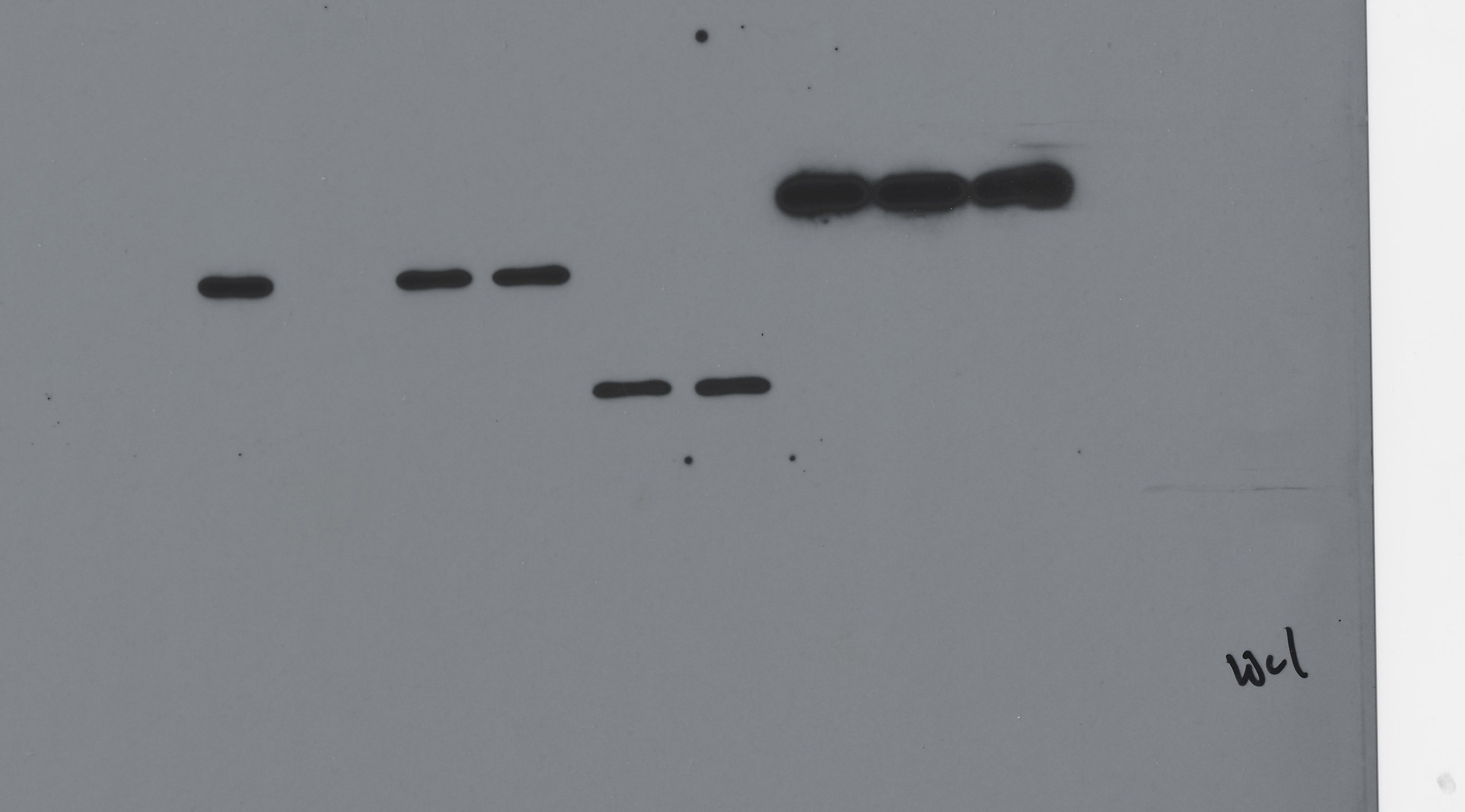

Supplement: Supplementary file 12 — Appendix Source Data [file 44319_2024_64_MOESM12_ESM.zip › Figure S4/4A/WCL IB Flag (RhoGDI).jpg]

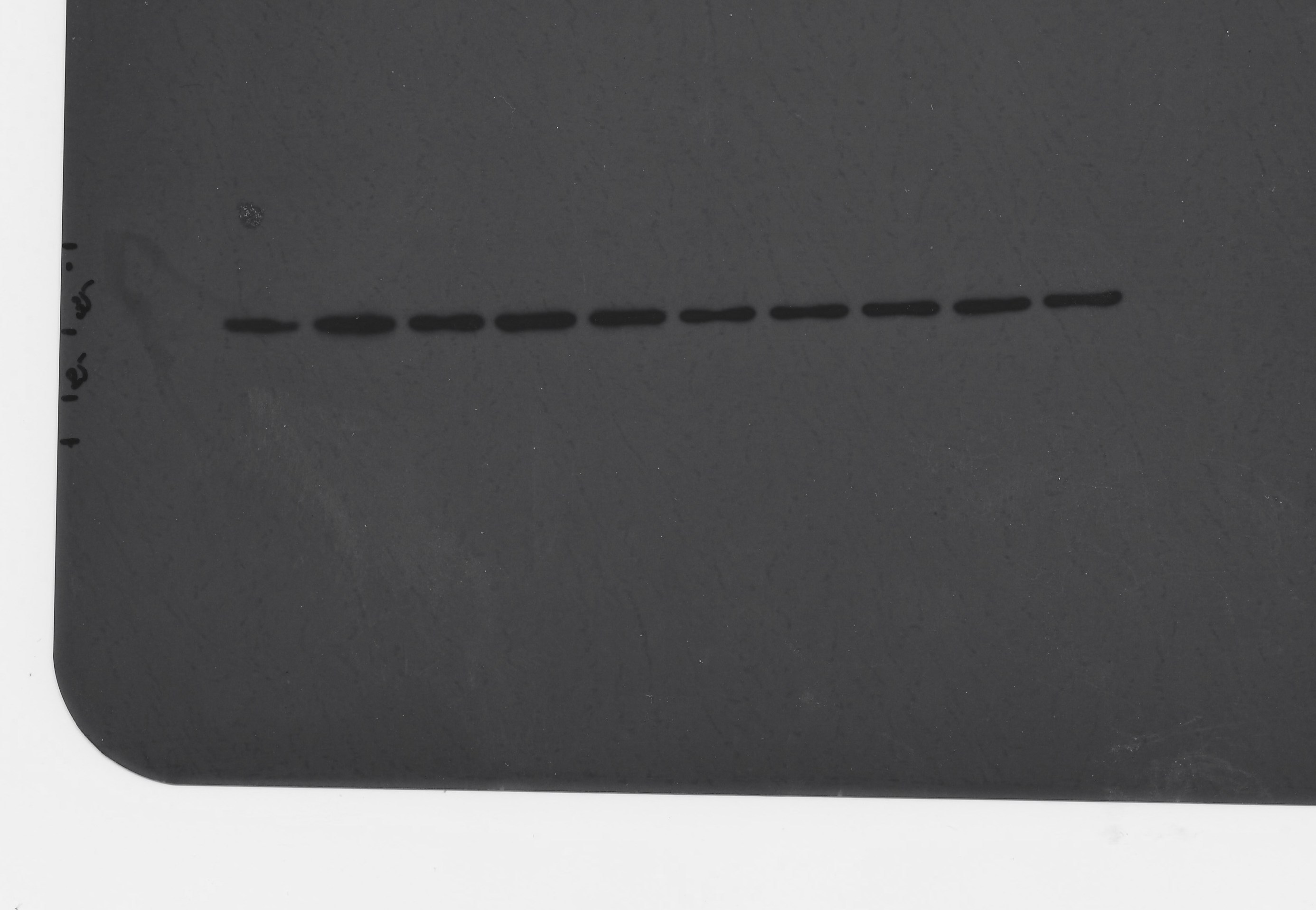

Supplement: Supplementary file 12 — Appendix Source Data [file 44319_2024_64_MOESM12_ESM.zip › Figure S4/4A/WCL IB GAPDH.jpg]

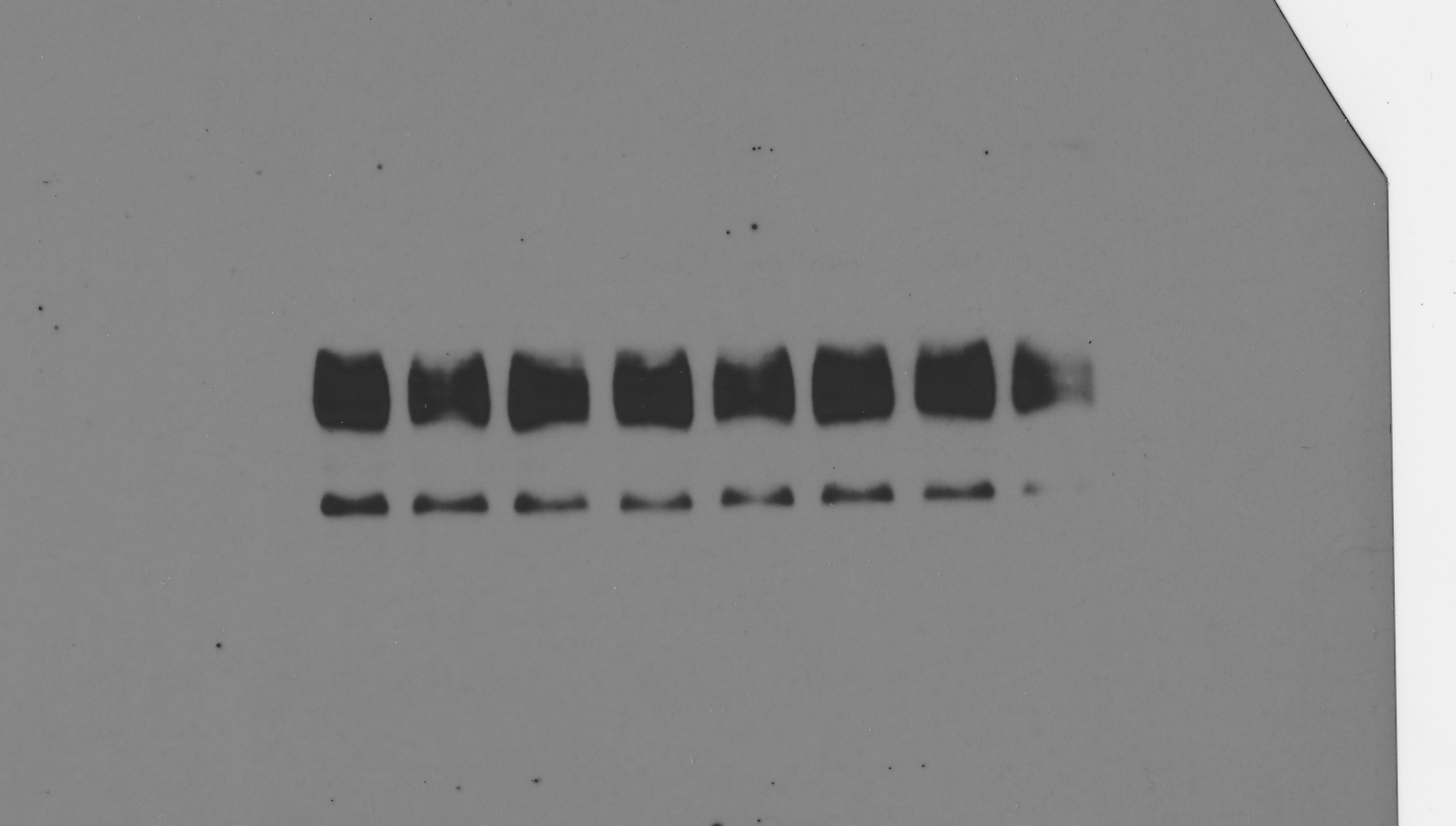

Supplement: Supplementary file 12 — Appendix Source Data [file 44319_2024_64_MOESM12_ESM.zip › Figure S4/4A/WCL IB p75NTR.jpg]

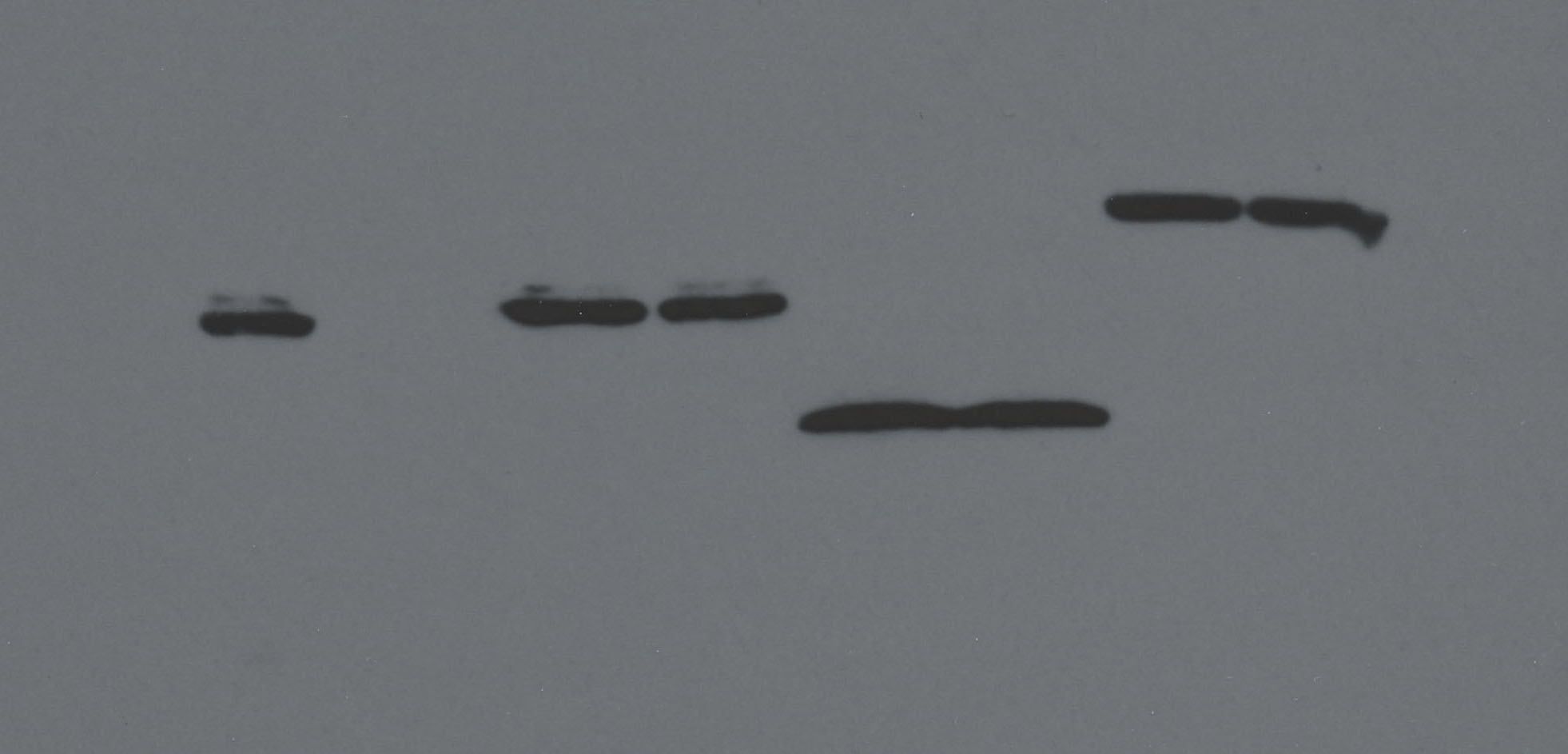

Supplement: Supplementary file 12 — Appendix Source Data [file 44319_2024_64_MOESM12_ESM.zip › Figure S4/4B/WCL IB Flag (RhoGDI).jpg]

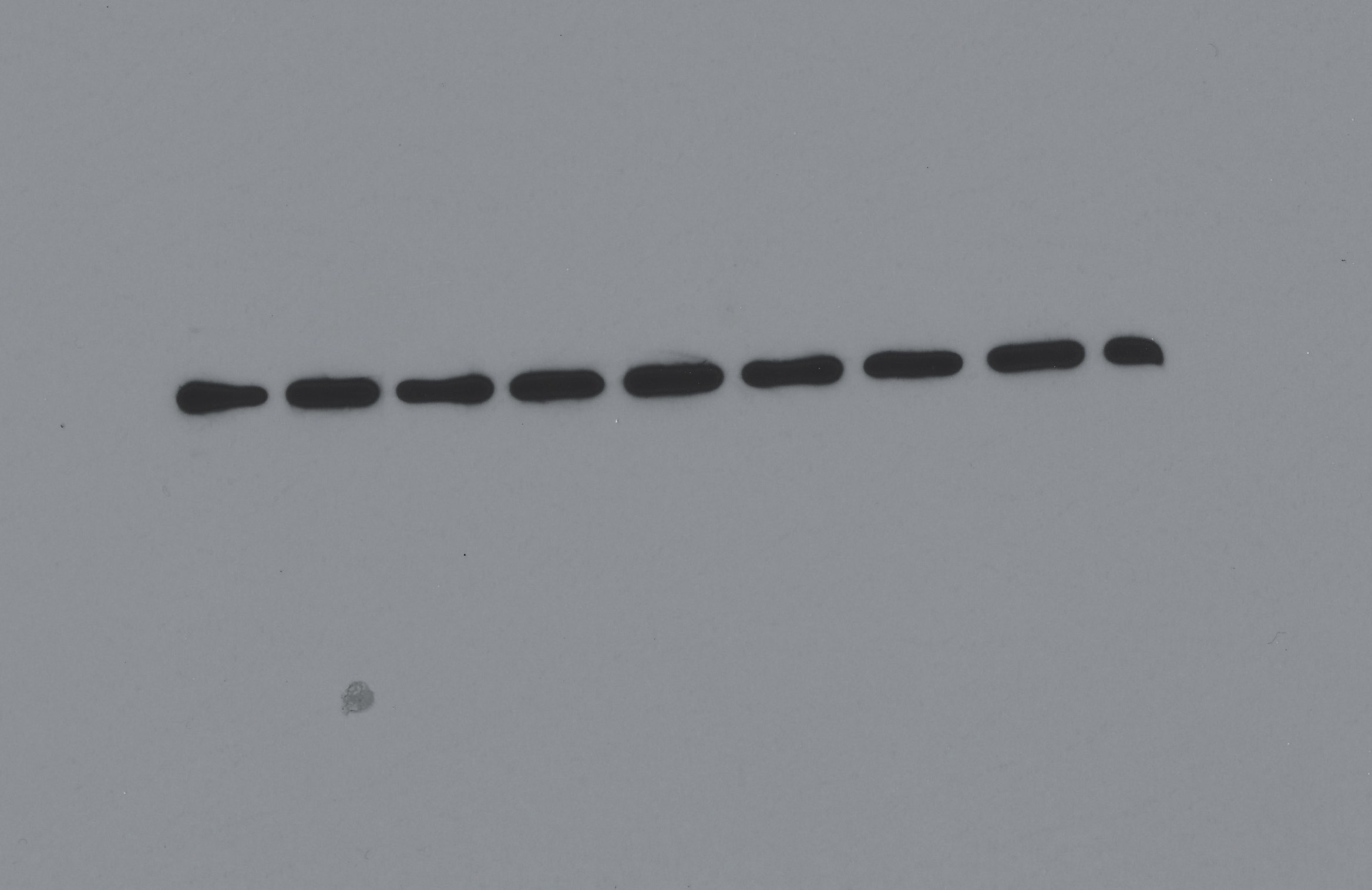

Supplement: Supplementary file 12 — Appendix Source Data [file 44319_2024_64_MOESM12_ESM.zip › Figure S4/4B/WCL IB GAPDH.jpg]

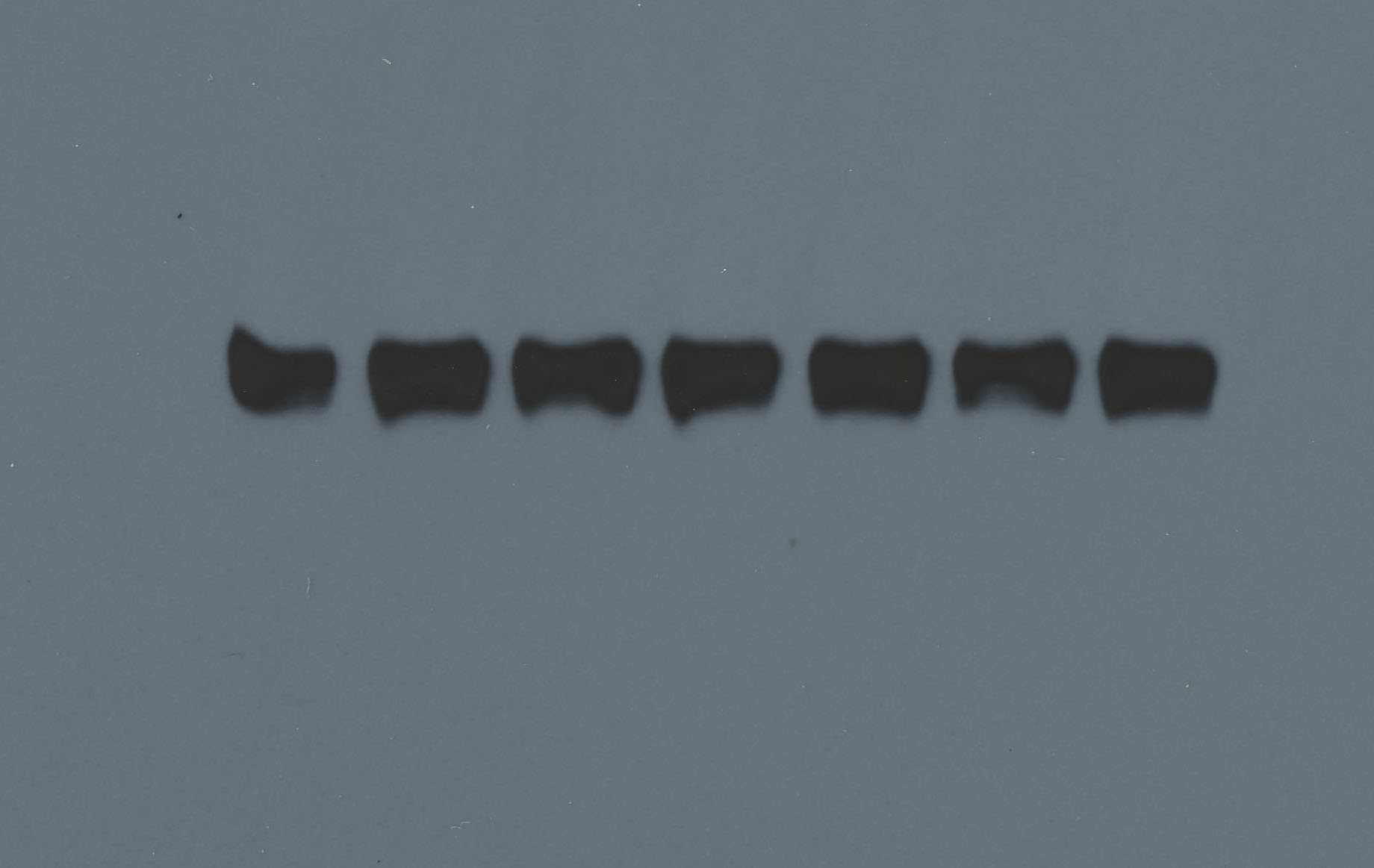

Supplement: Supplementary file 12 — Appendix Source Data [file 44319_2024_64_MOESM12_ESM.zip › Figure S4/4B/WCL IB p75NTR.jpg]

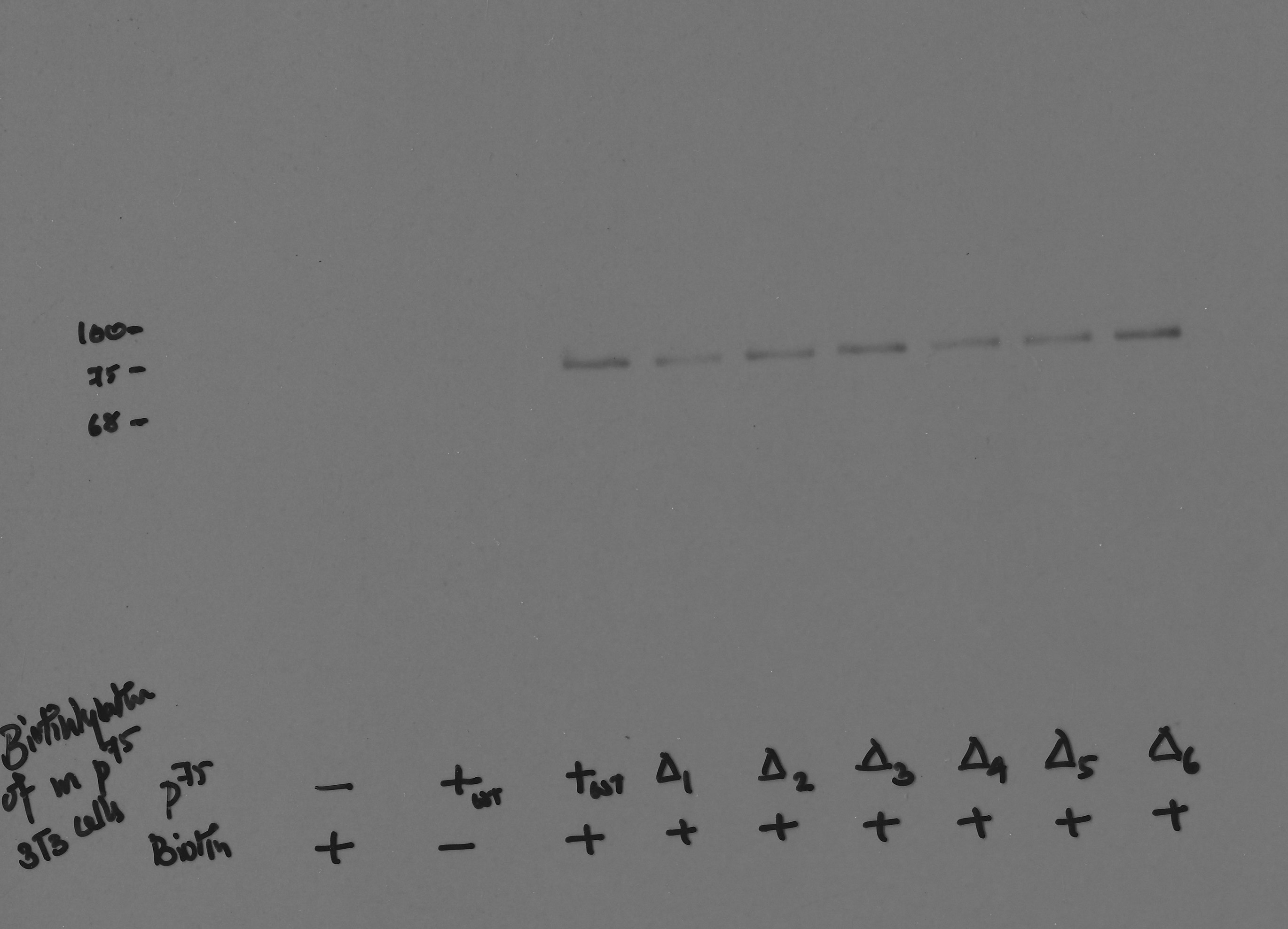

Supplement: Supplementary file 12 — Appendix Source Data [file 44319_2024_64_MOESM12_ESM.zip › Figure S4/4C/IP p75NTR blot Neutravidin HRP.jpg]

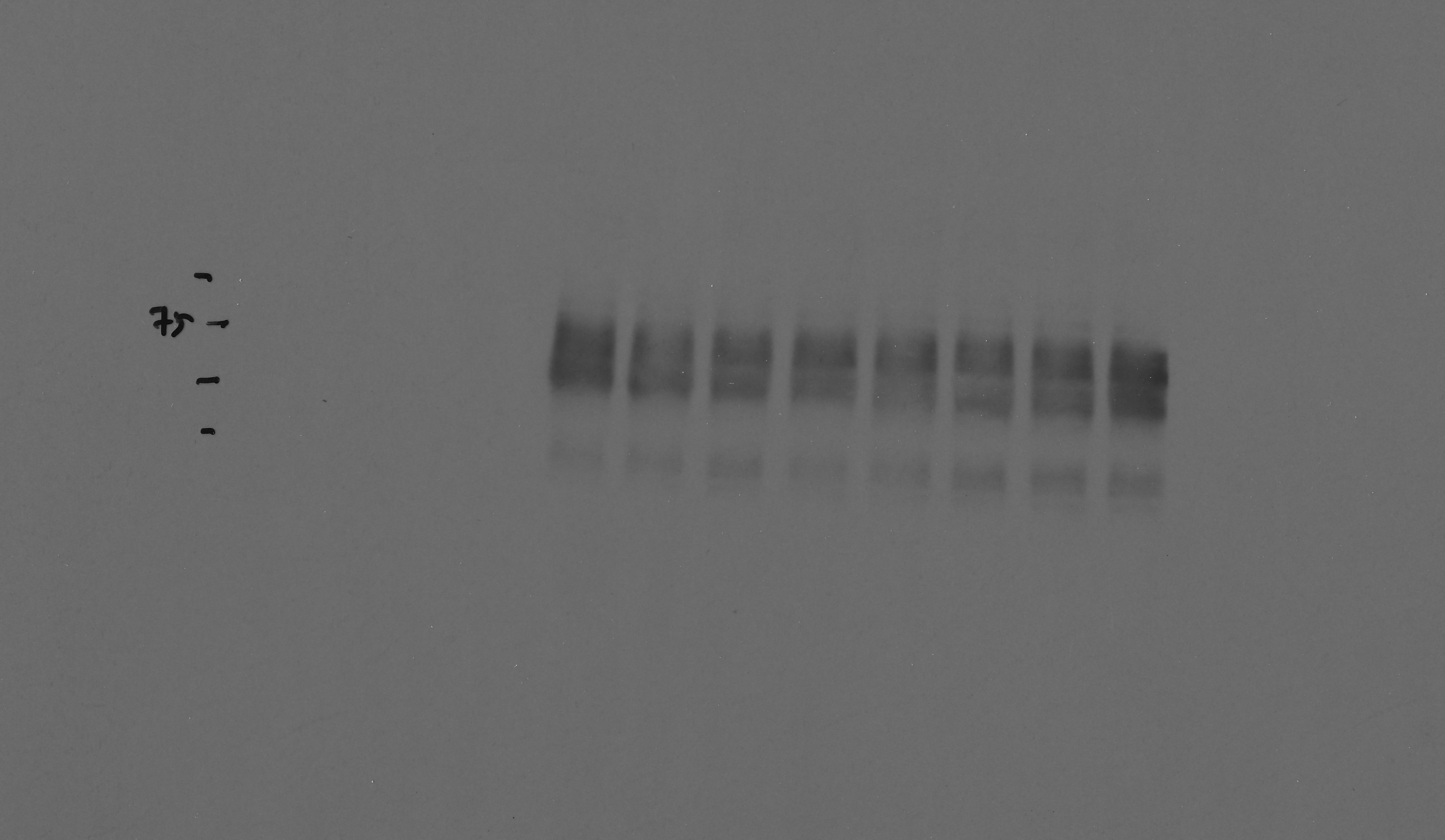

Supplement: Supplementary file 12 — Appendix Source Data [file 44319_2024_64_MOESM12_ESM.zip › Figure S4/4C/IP p75NTR IB p75NTR.jpg]

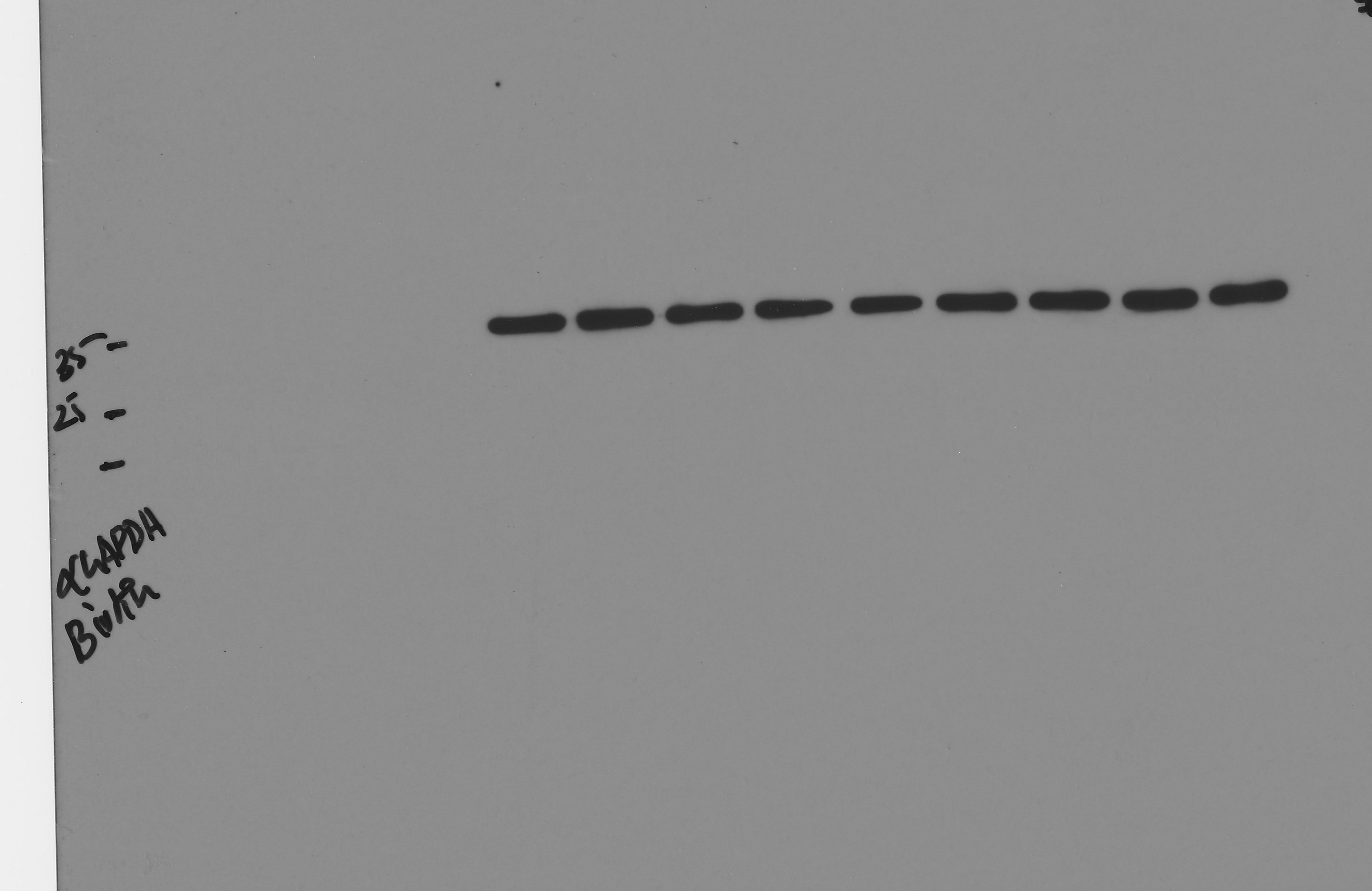

Supplement: Supplementary file 12 — Appendix Source Data [file 44319_2024_64_MOESM12_ESM.zip › Figure S4/4C/WCL GAPDH.jpg]

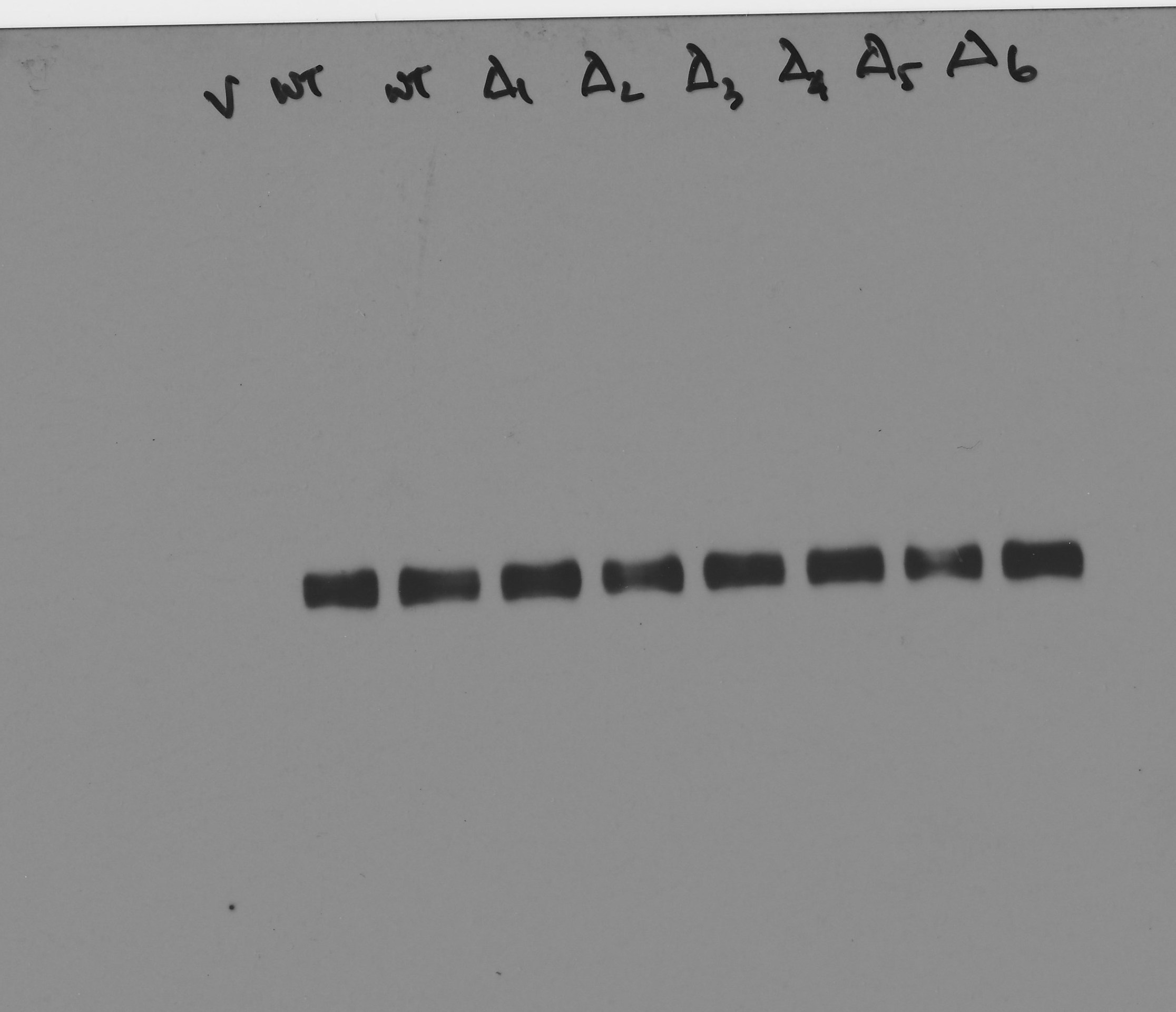

Supplement: Supplementary file 12 — Appendix Source Data [file 44319_2024_64_MOESM12_ESM.zip › Figure S4/4C/WCL p75NTR.jpg]

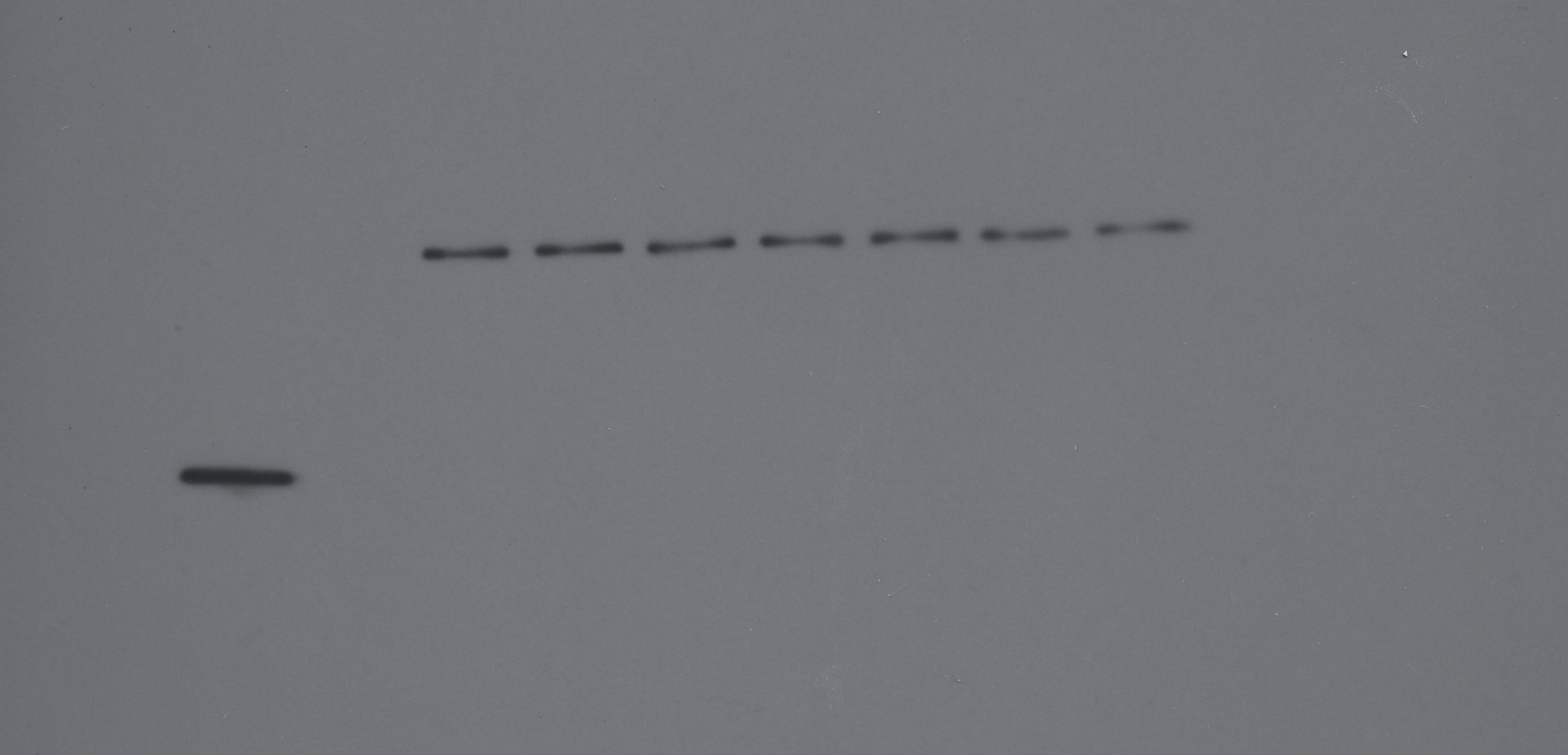

Supplement: Supplementary file 12 — Appendix Source Data [file 44319_2024_64_MOESM12_ESM.zip › Figure S4/4D/WCL HA (p75NTR Juxtamembrane).jpg]

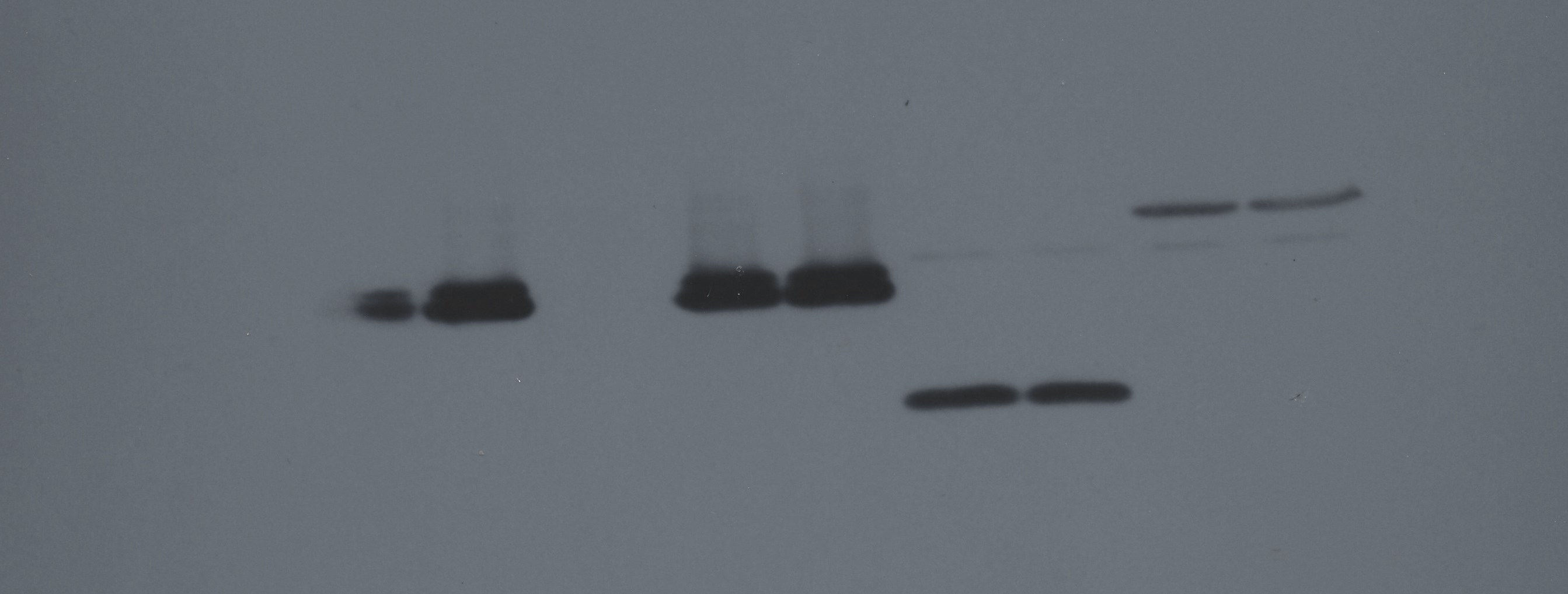

Supplement: Supplementary file 12 — Appendix Source Data [file 44319_2024_64_MOESM12_ESM.zip › Figure S4/4D/WCL IB Flag (RhoGDI).jpg]

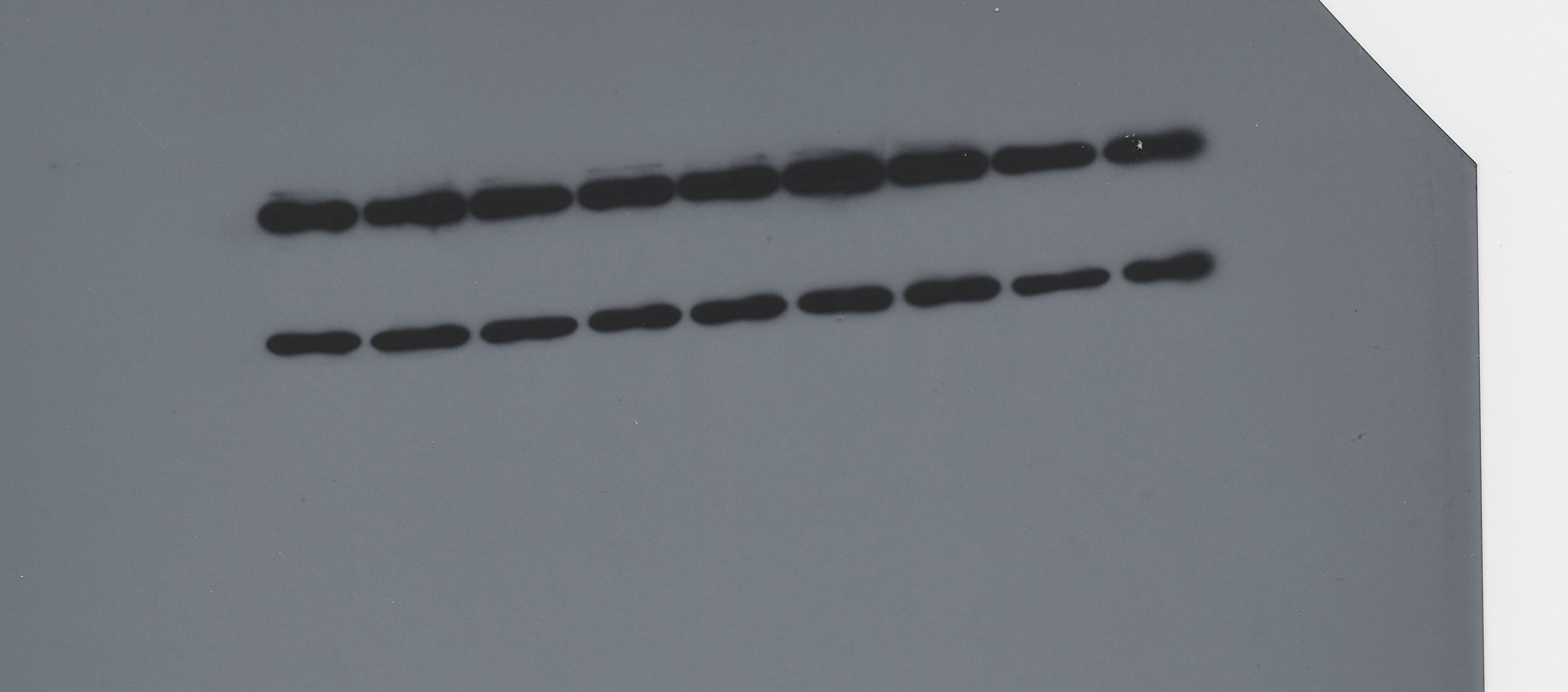

Supplement: Supplementary file 12 — Appendix Source Data [file 44319_2024_64_MOESM12_ESM.zip › Figure S4/4D/WCL IB GAPDH.jpg]

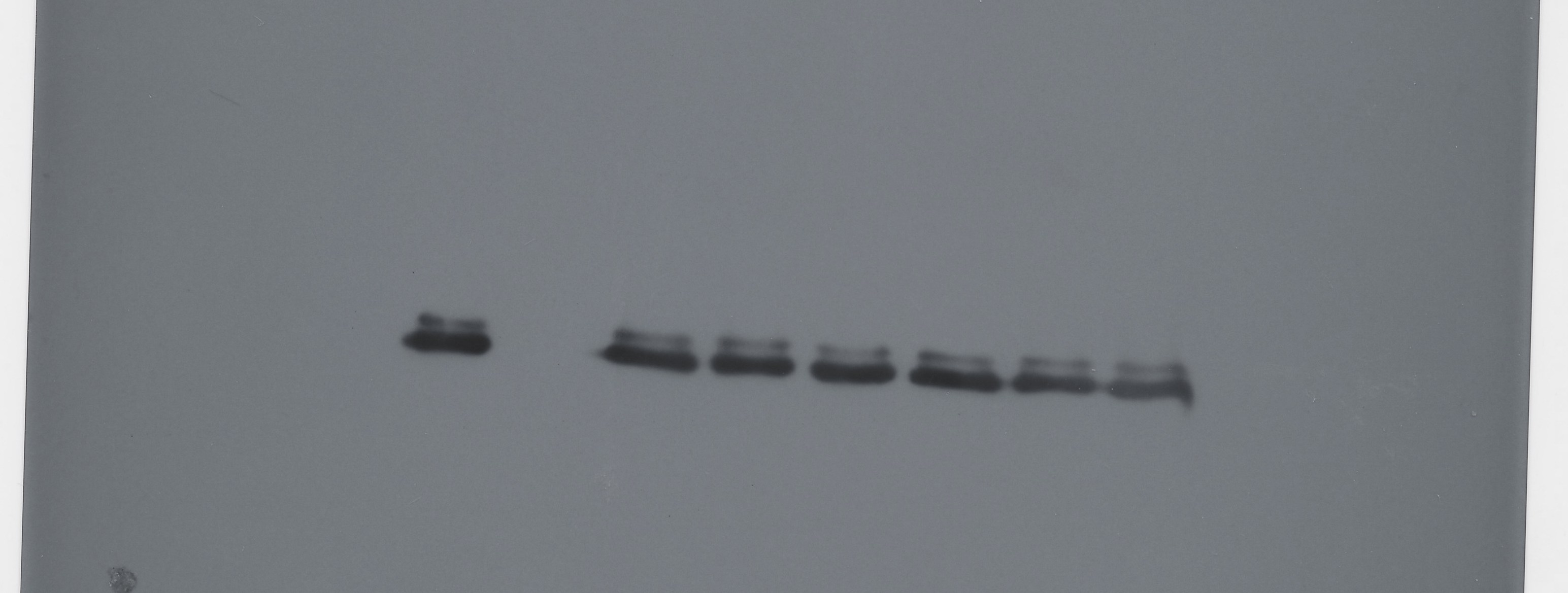

Supplement: Supplementary file 12 — Appendix Source Data [file 44319_2024_64_MOESM12_ESM.zip › Figure S4/4E/WCL IB Flag (RhoGDI).jpg]

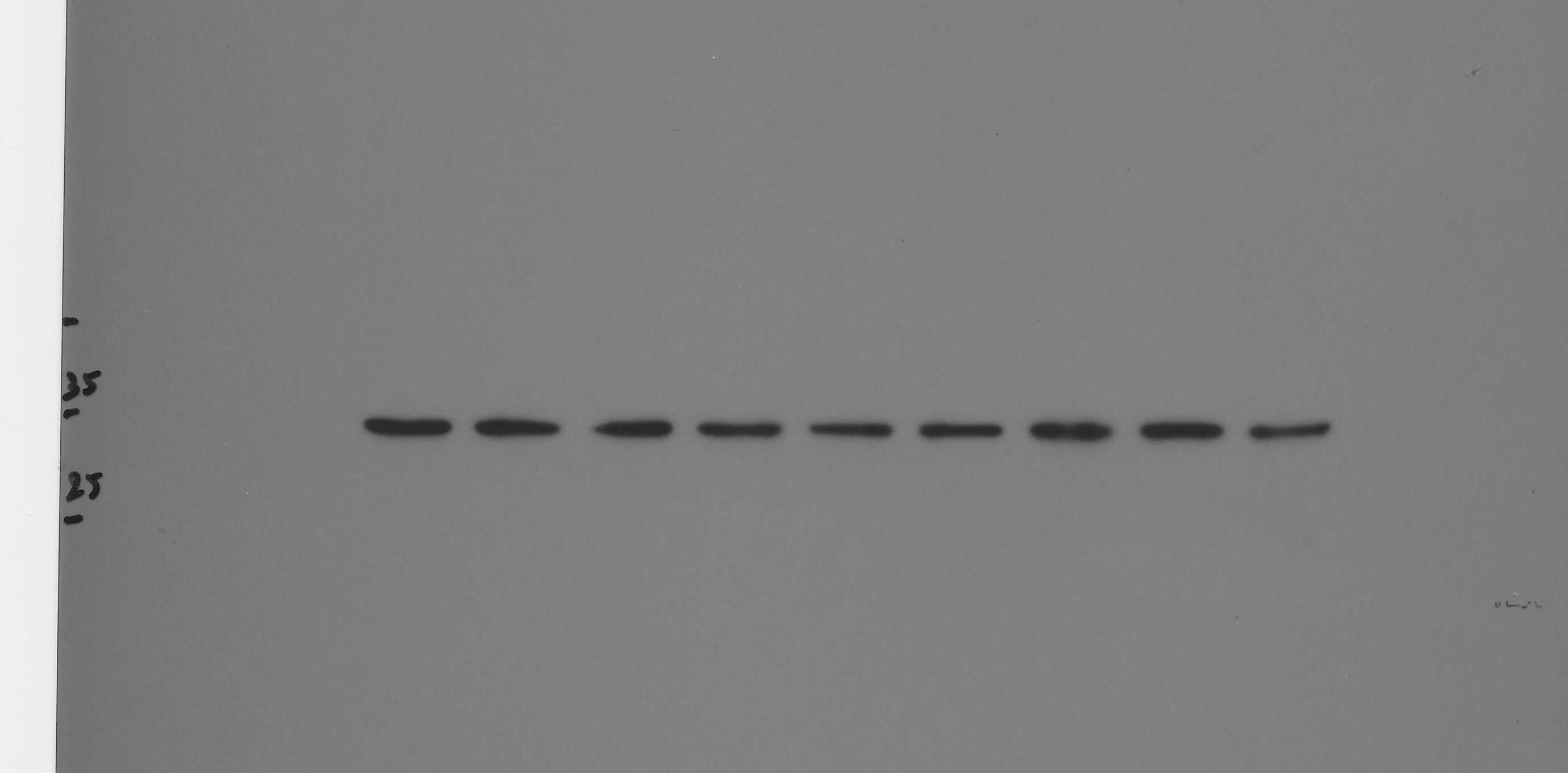

Supplement: Supplementary file 12 — Appendix Source Data [file 44319_2024_64_MOESM12_ESM.zip › Figure S4/4E/WCL IB GAPDH.jpg]

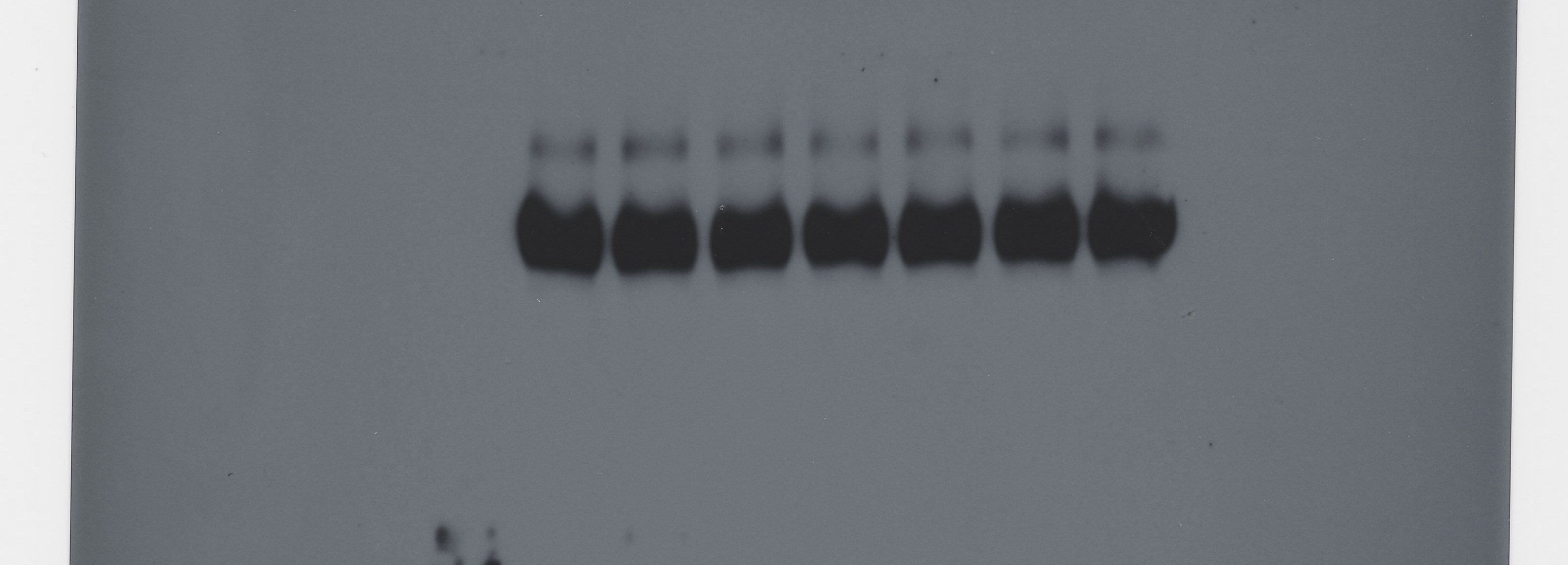

Supplement: Supplementary file 12 — Appendix Source Data [file 44319_2024_64_MOESM12_ESM.zip › Figure S4/4E/WCL IB p75NTR.jpg]

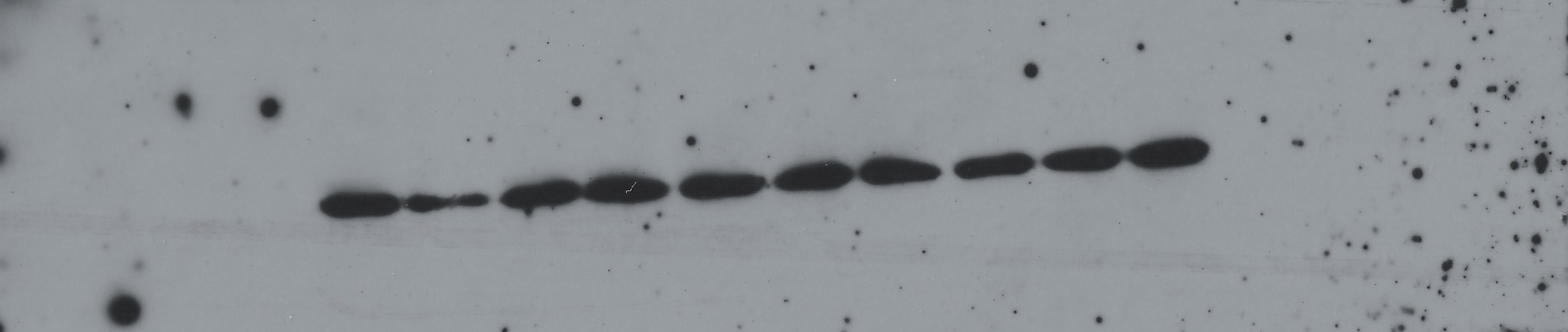

Supplement: Supplementary file 12 — Appendix Source Data [file 44319_2024_64_MOESM12_ESM.zip › Figure S4/4F/WCL IB Flag (RhoGDI).jpg]

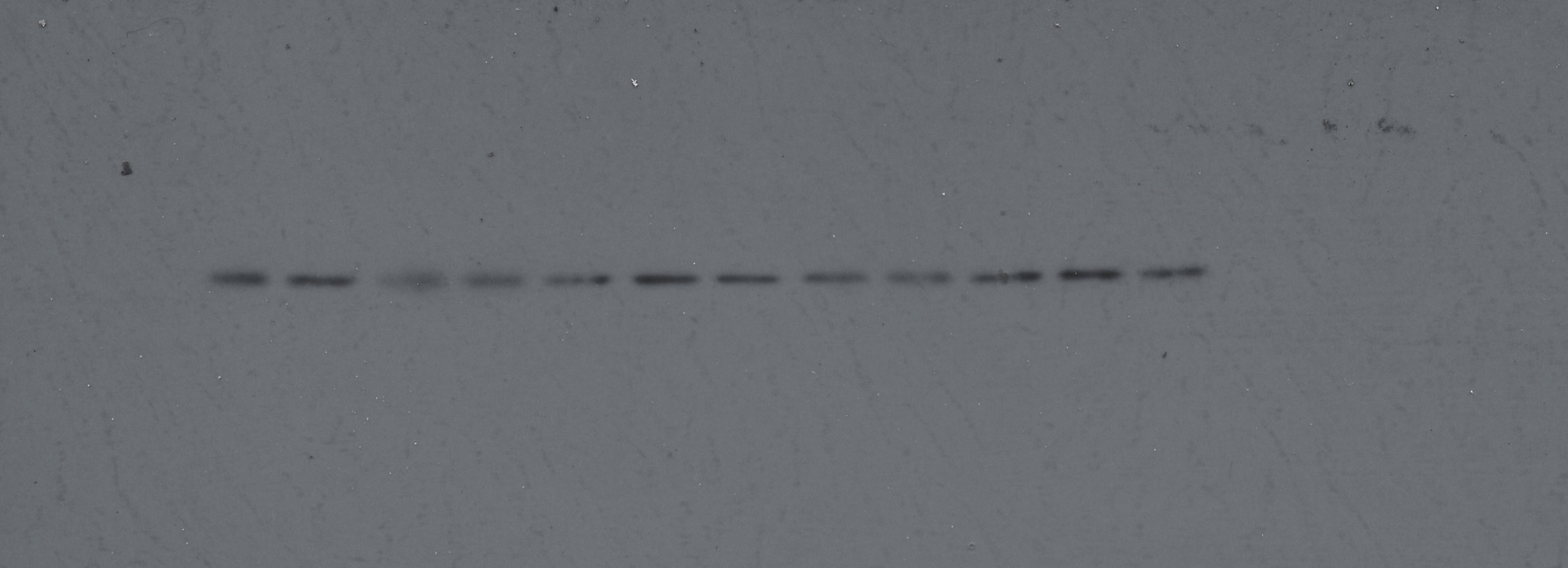

Supplement: Supplementary file 12 — Appendix Source Data [file 44319_2024_64_MOESM12_ESM.zip › Figure S4/4F/WCL IB GAPDH.jpg]

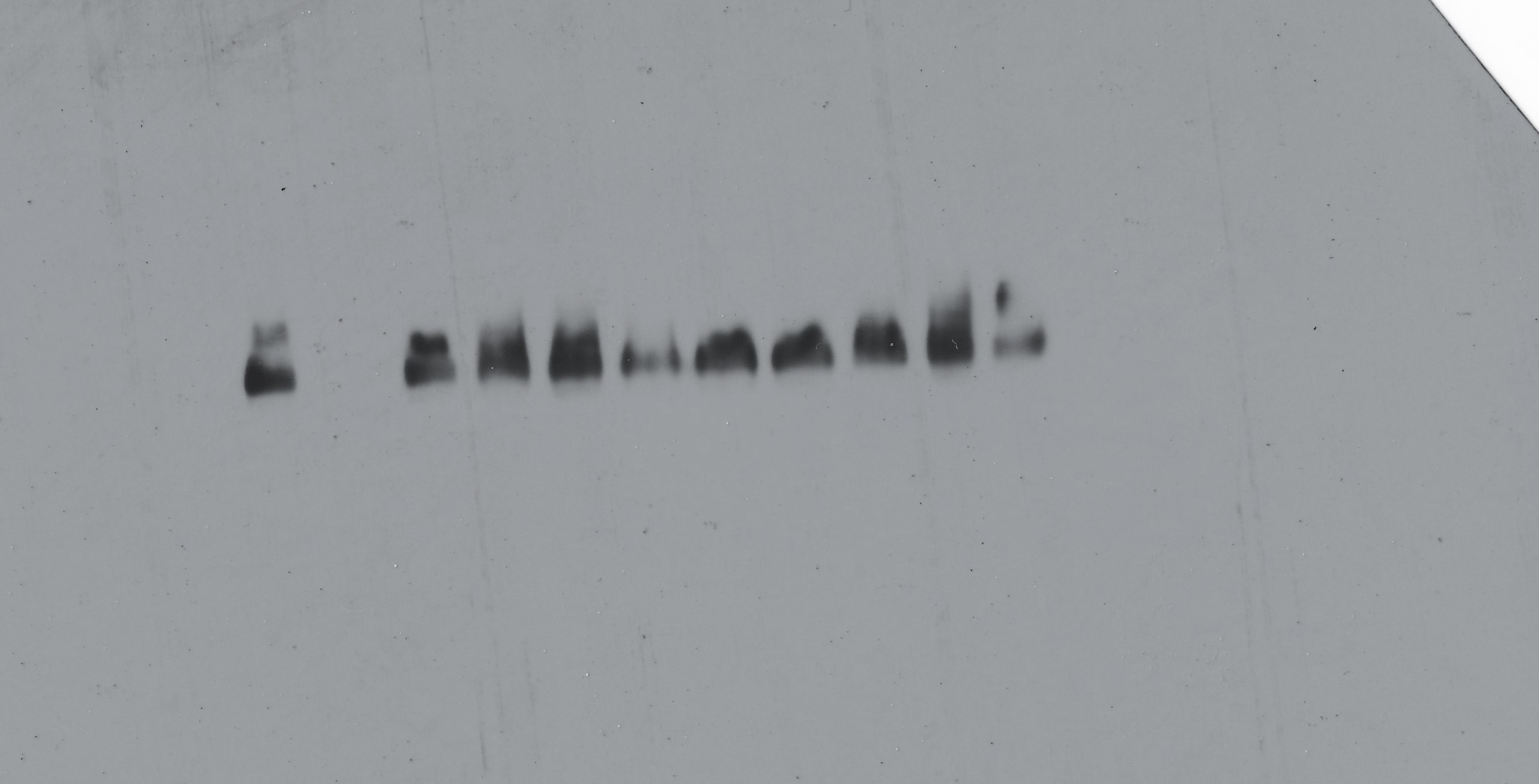

Supplement: Supplementary file 12 — Appendix Source Data [file 44319_2024_64_MOESM12_ESM.zip › Figure S4/4F/WCL IB p75NTR.jpg]

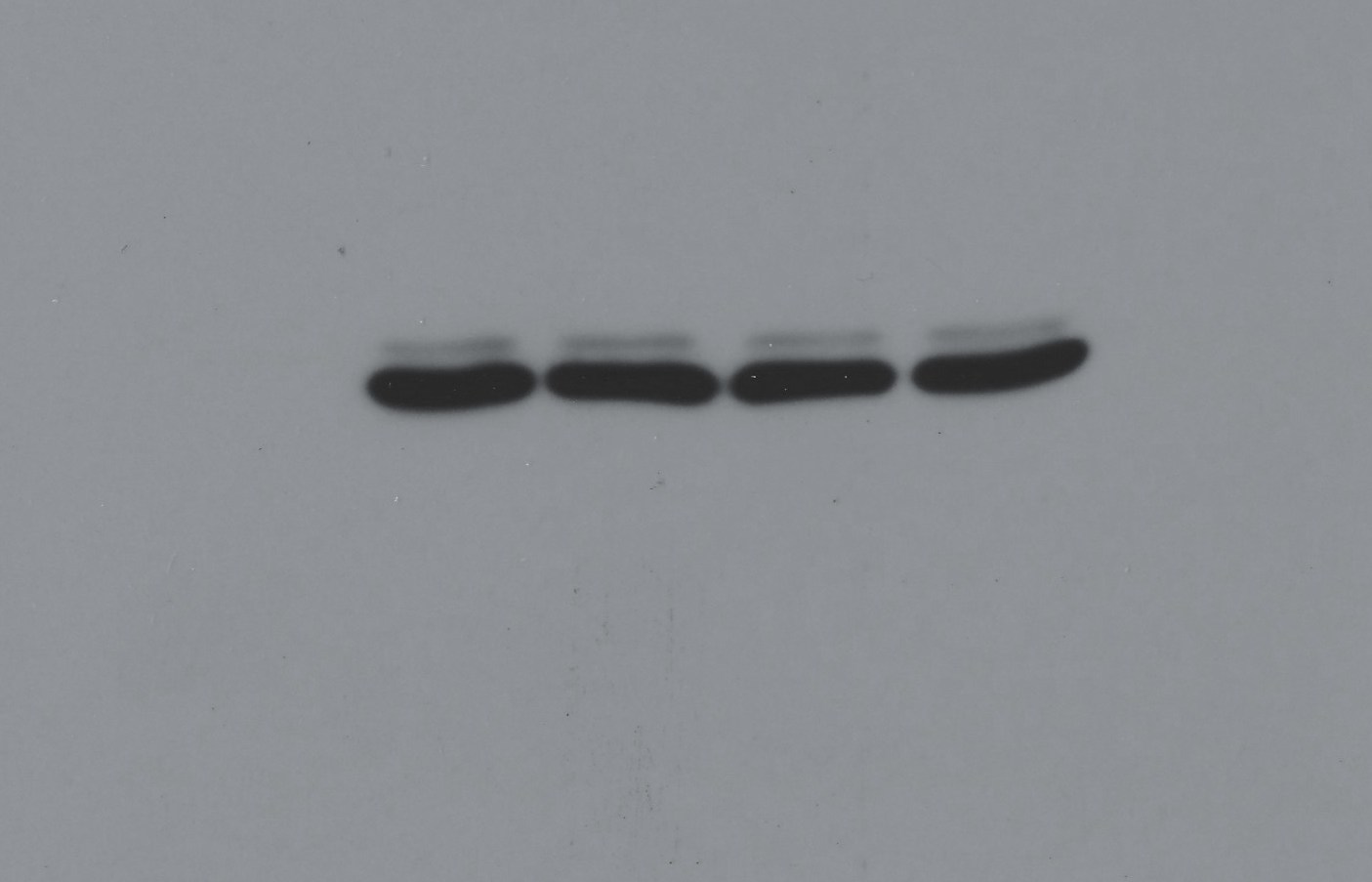

Supplement: Supplementary file 12 — Appendix Source Data [file 44319_2024_64_MOESM12_ESM.zip › Figure S4/4G/WCL IB Flag (RhoGDI).jpg]

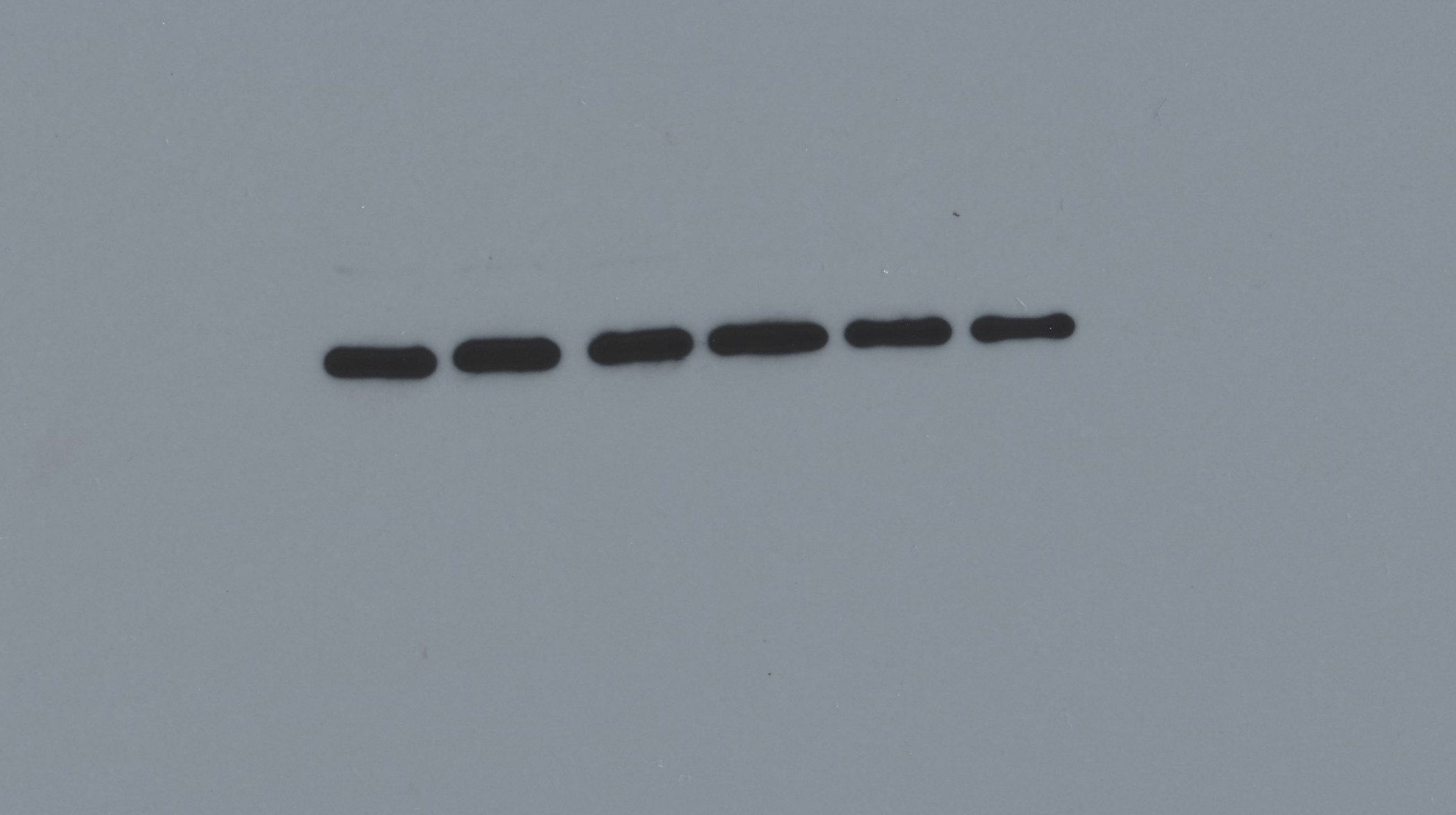

Supplement: Supplementary file 12 — Appendix Source Data [file 44319_2024_64_MOESM12_ESM.zip › Figure S4/4G/WCL IB GAPDH.jpg]

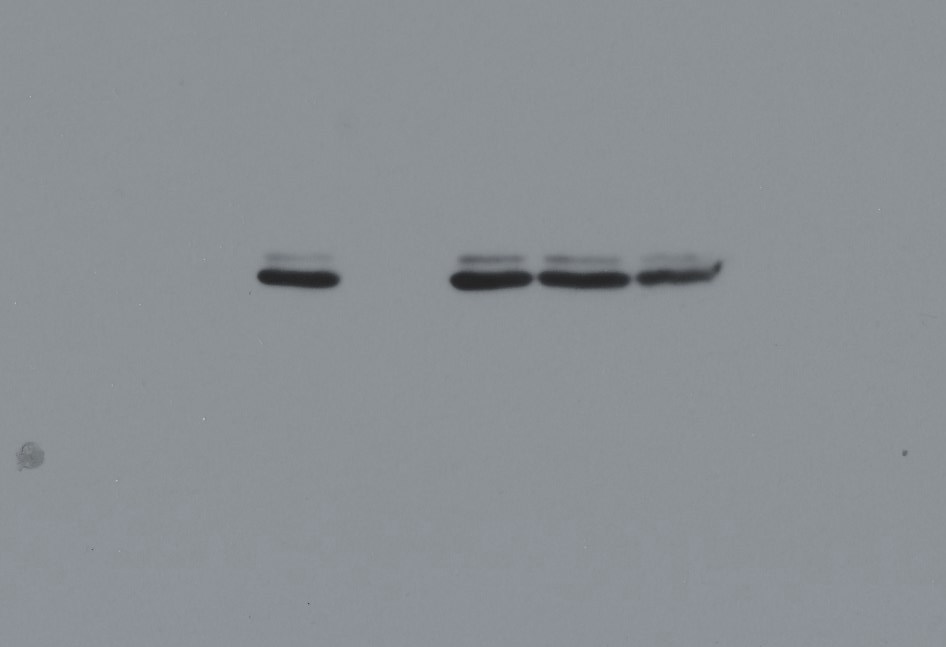

Supplement: Supplementary file 12 — Appendix Source Data [file 44319_2024_64_MOESM12_ESM.zip › Figure S4/4G/WCL IB HA (RhoA).jpg]

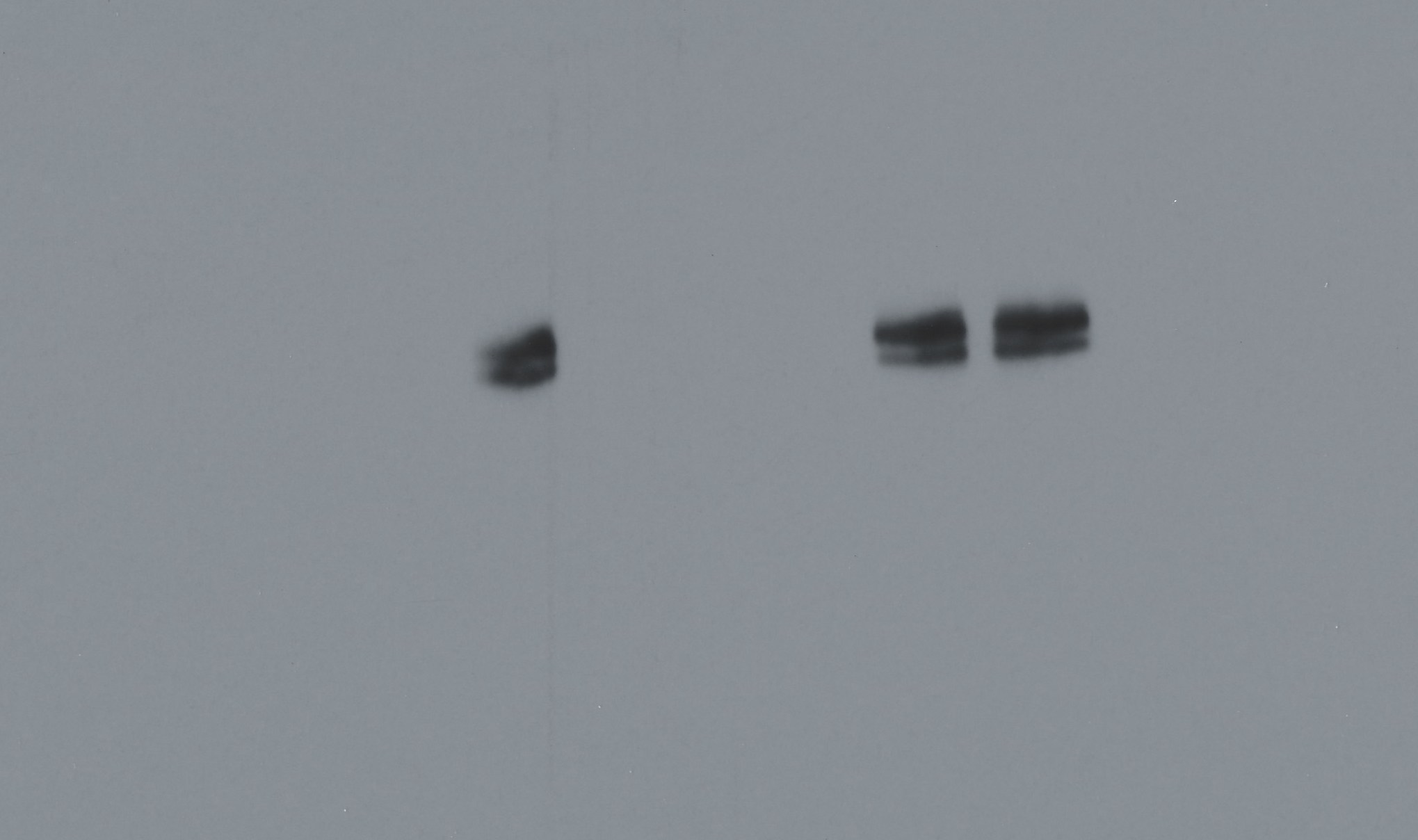

Supplement: Supplementary file 12 — Appendix Source Data [file 44319_2024_64_MOESM12_ESM.zip › Figure S4/4G/WCL IB p75NTR.jpg]

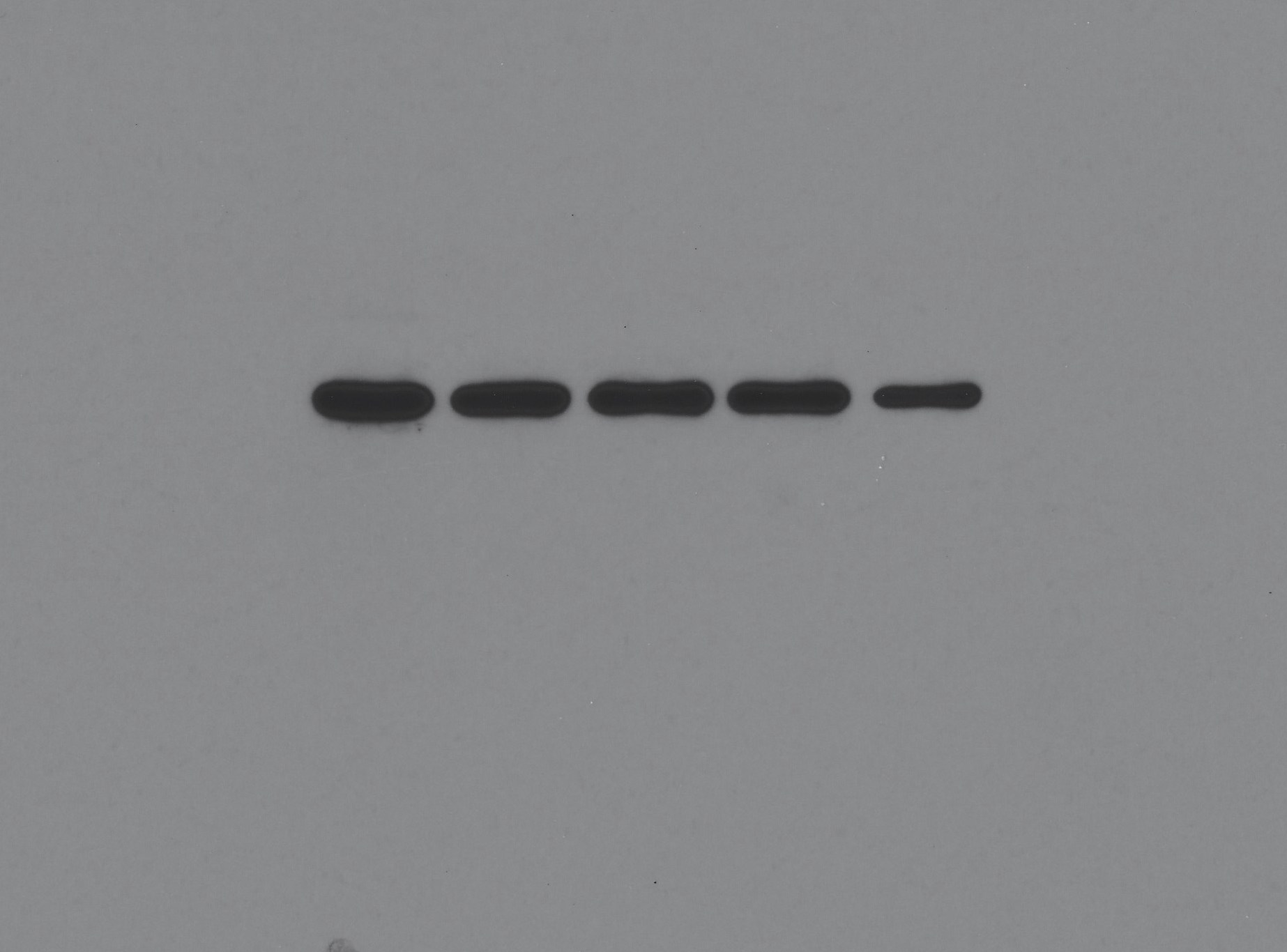

Supplement: Supplementary file 12 — Appendix Source Data [file 44319_2024_64_MOESM12_ESM.zip › Figure S4/4H/WCL IB GAPDH.jpg]

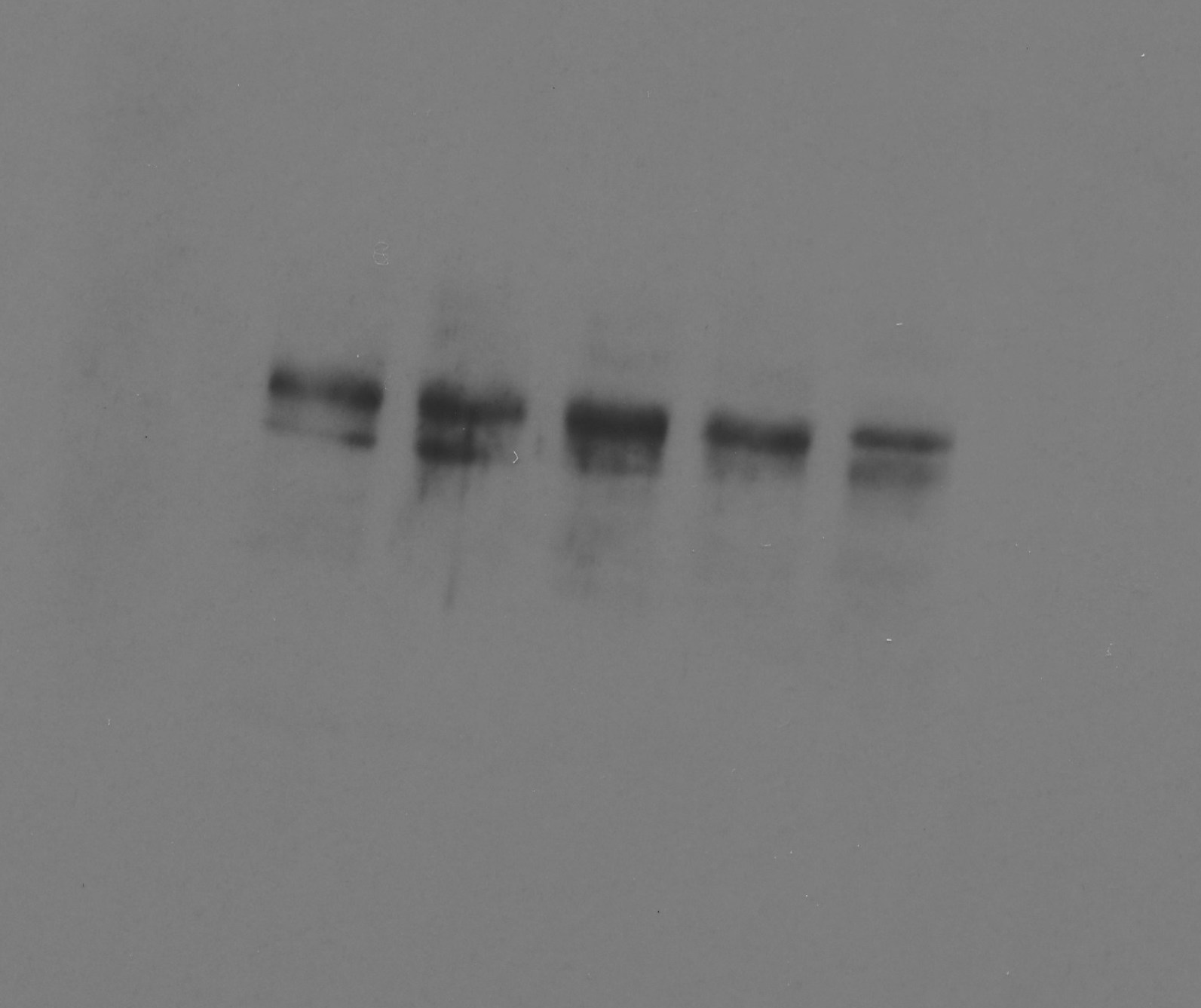

Supplement: Supplementary file 12 — Appendix Source Data [file 44319_2024_64_MOESM12_ESM.zip › Figure S4/4H/WCL IB p75NTR.jpg]

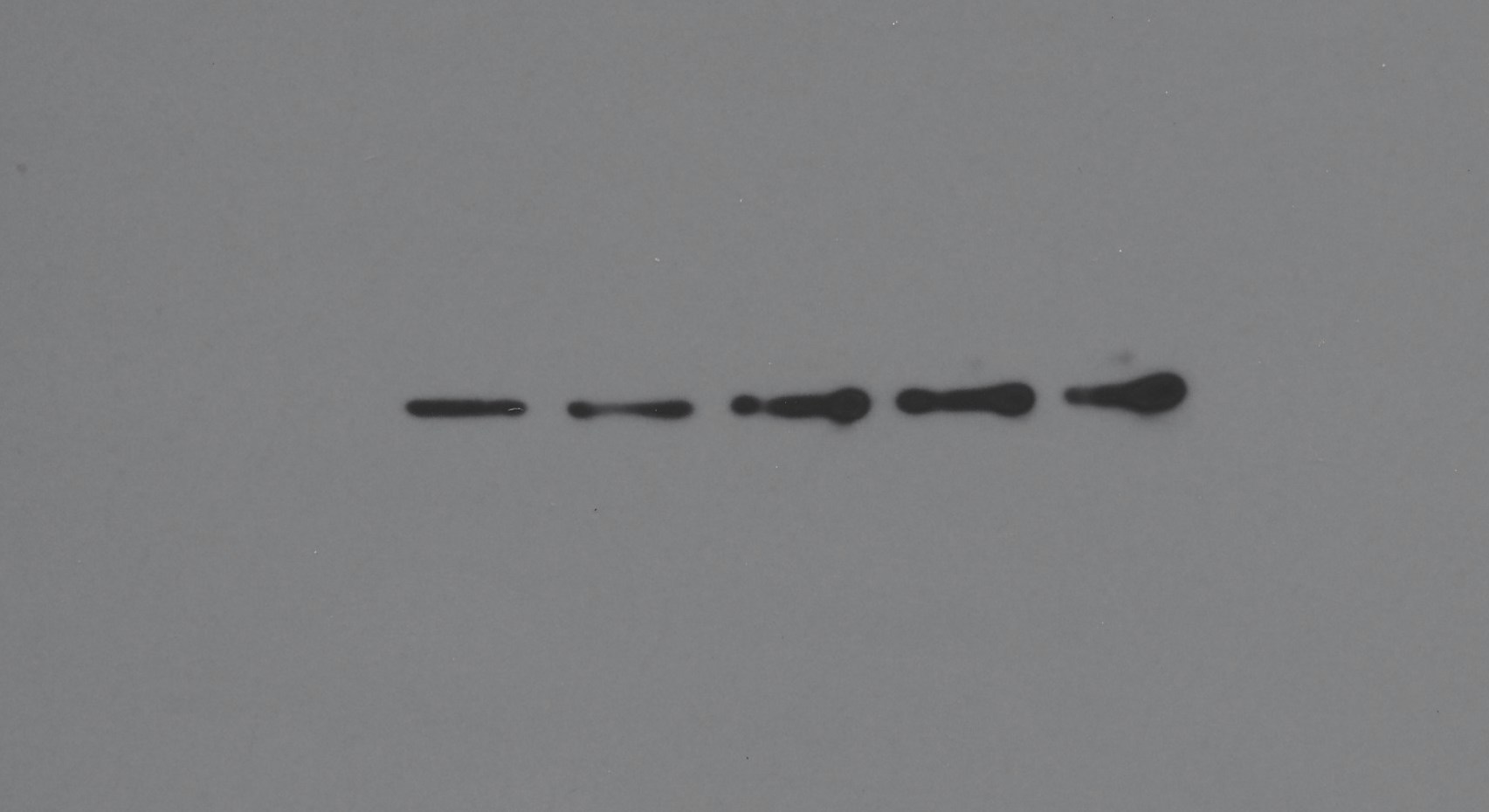

Supplement: Supplementary file 12 — Appendix Source Data [file 44319_2024_64_MOESM12_ESM.zip › Figure S4/4H/WCL IB RhoGDI.jpg]

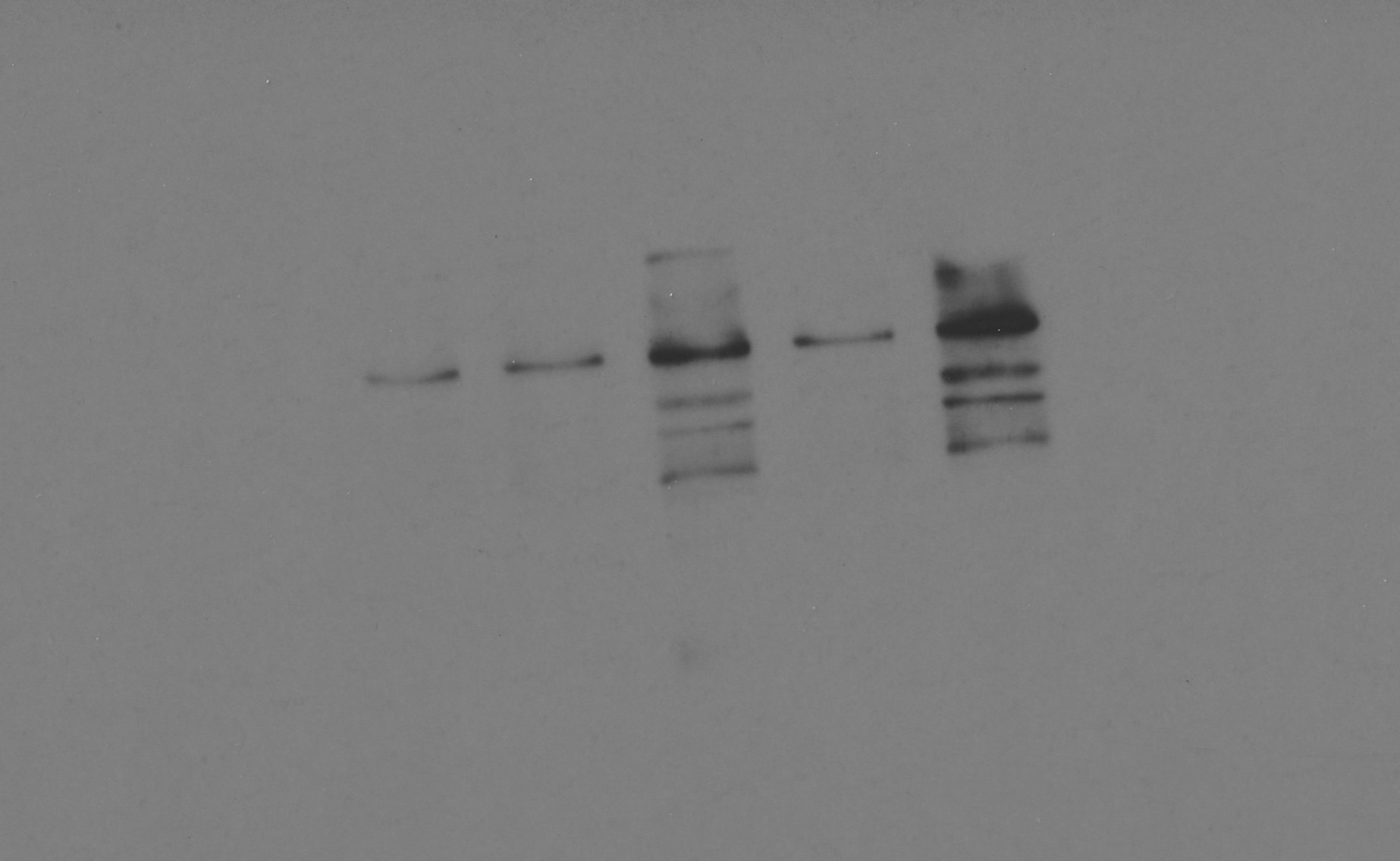

Supplement: Supplementary file 12 — Appendix Source Data [file 44319_2024_64_MOESM12_ESM.zip › Figure S4/4H/WCL phospho (Ser) PKC alpha substrate antibody.jpg]

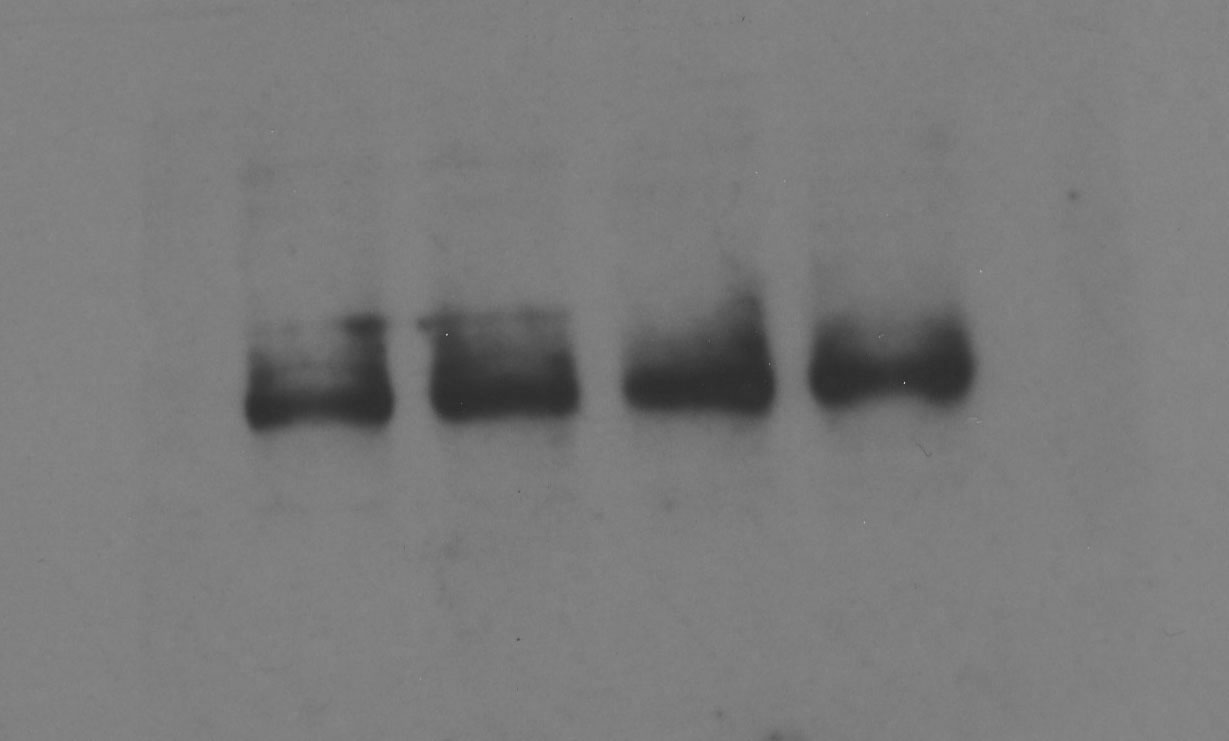

Supplement: Supplementary file 12 — Appendix Source Data [file 44319_2024_64_MOESM12_ESM.zip › Figure S1/1A/WCL - IB p75NTR.jpg]

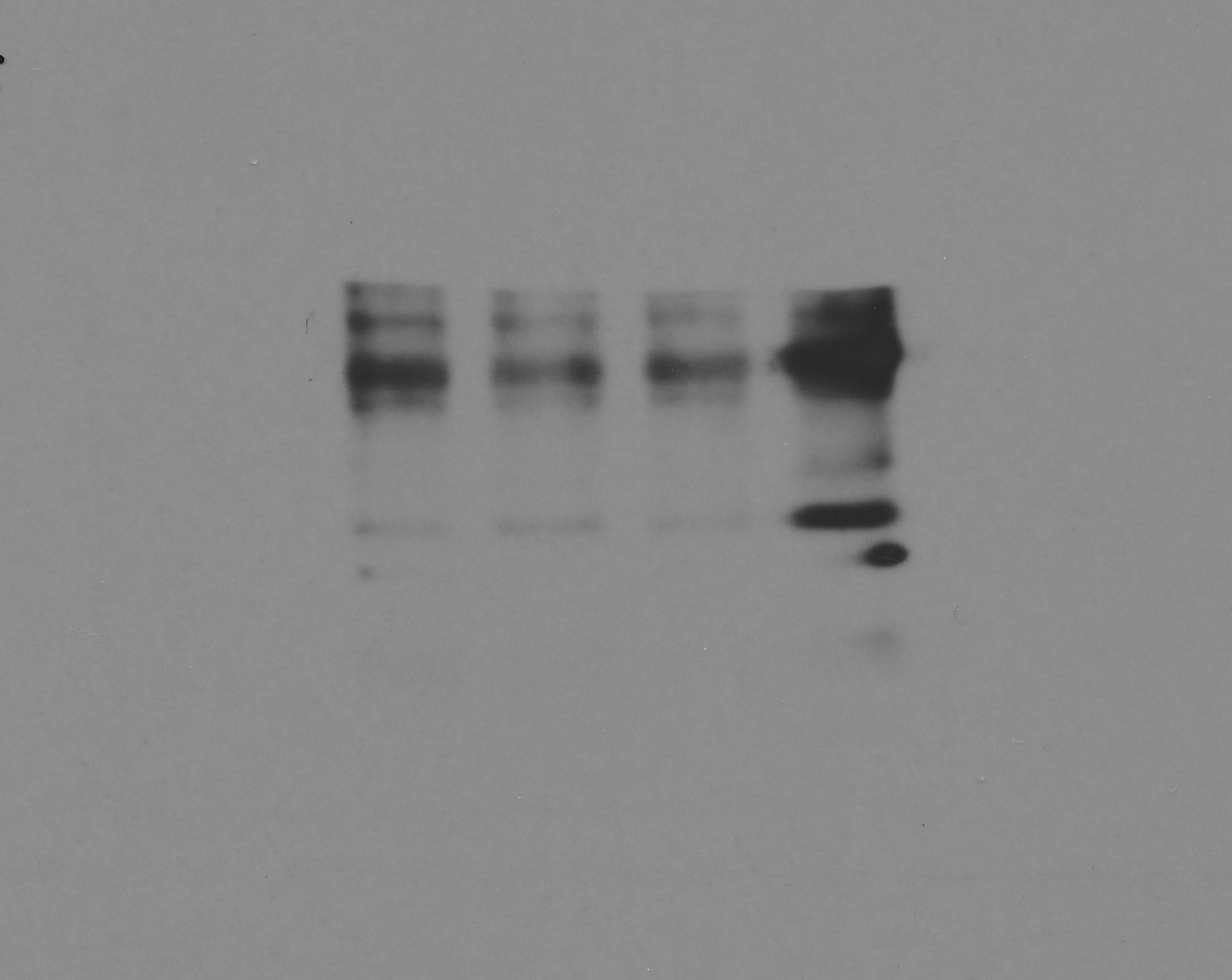

Supplement: Supplementary file 12 — Appendix Source Data [file 44319_2024_64_MOESM12_ESM.zip › Figure S1/1A/WCL - IB phospho (Ser) PKC-a substrate antibody.jpg]

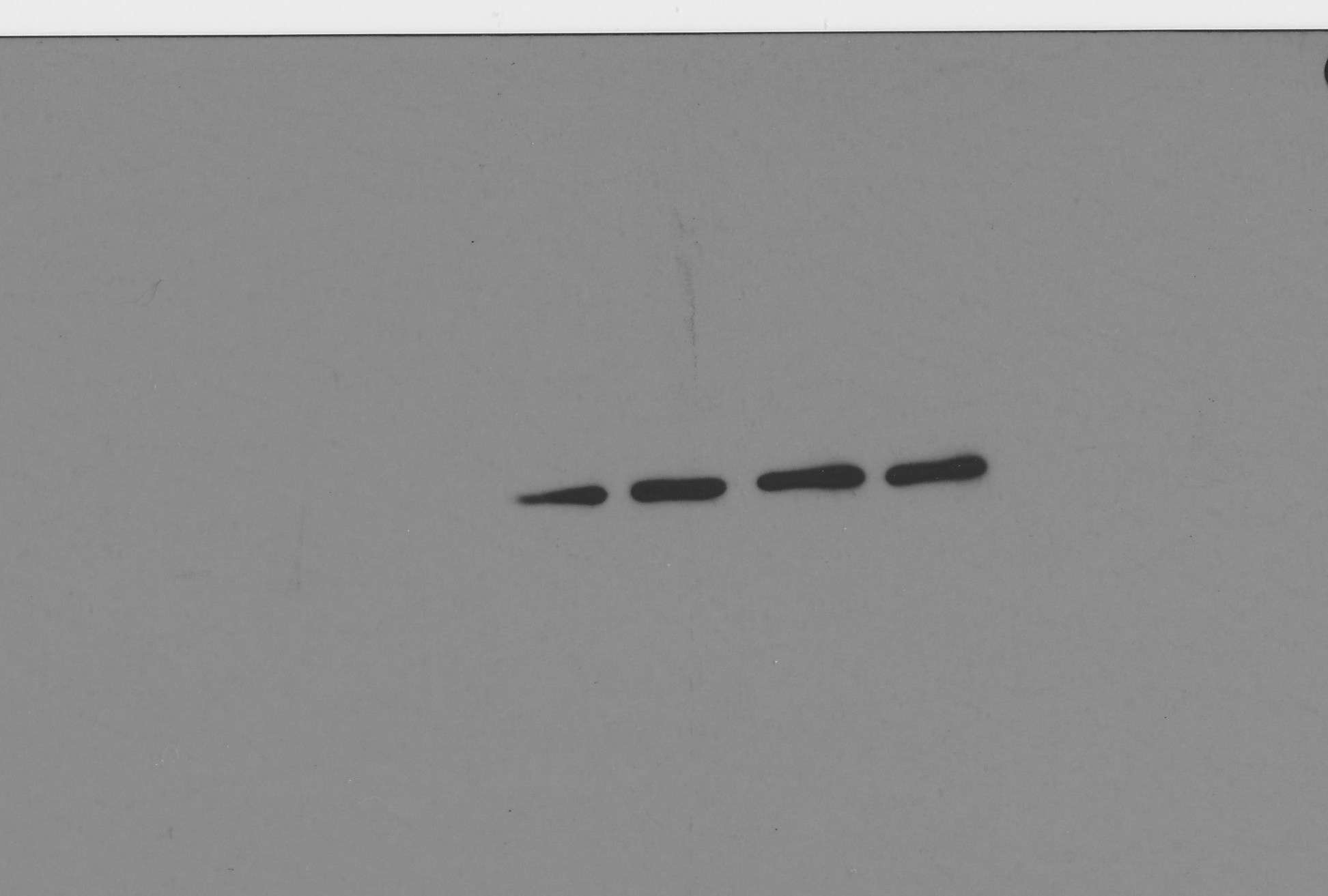

Supplement: Supplementary file 12 — Appendix Source Data [file 44319_2024_64_MOESM12_ESM.zip › Figure S1/1A/WCL IB GAPDH.jpeg]

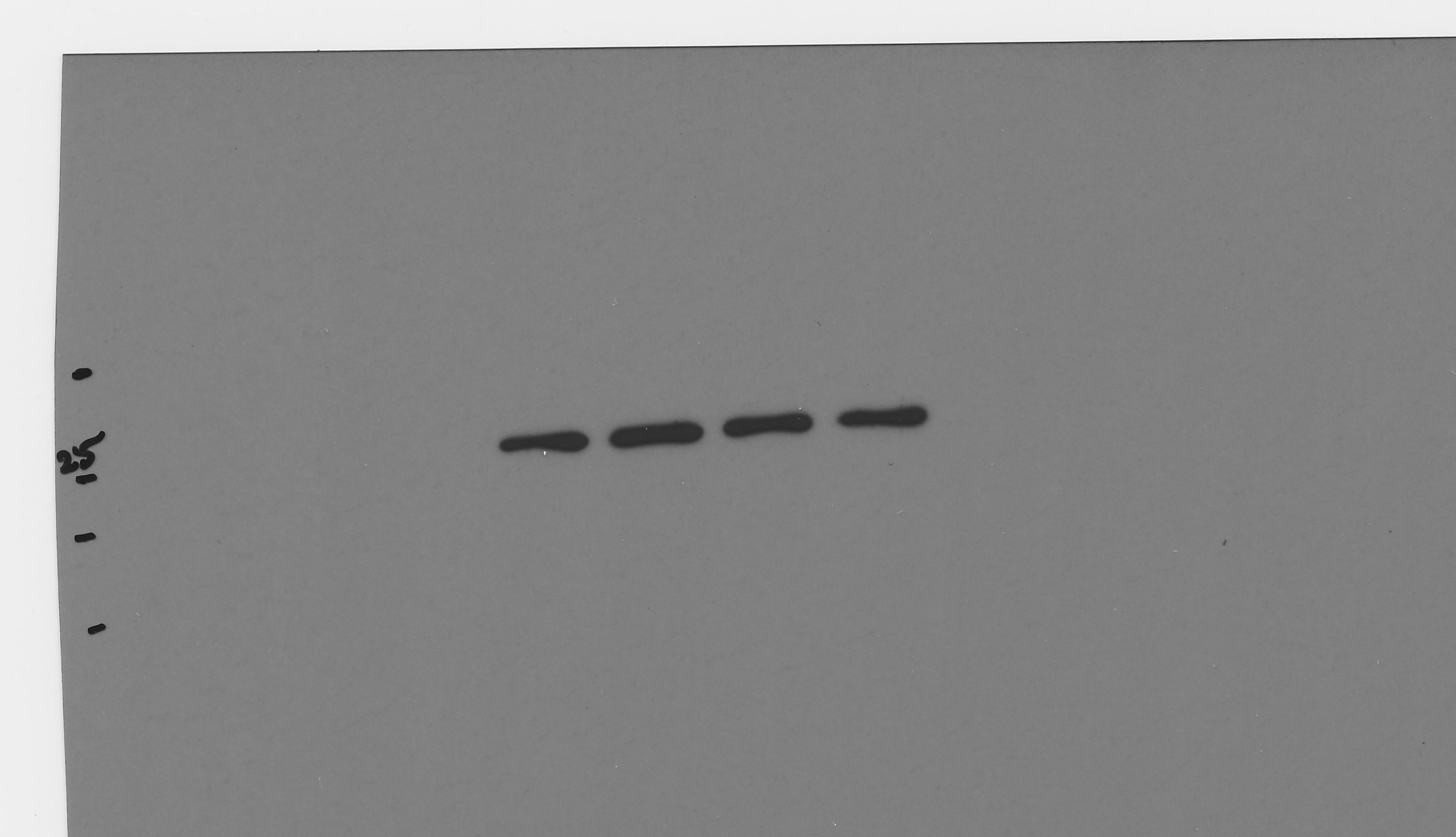

Supplement: Supplementary file 12 — Appendix Source Data [file 44319_2024_64_MOESM12_ESM.zip › Figure S1/1A/WCL IB RhoGDI.jpg]

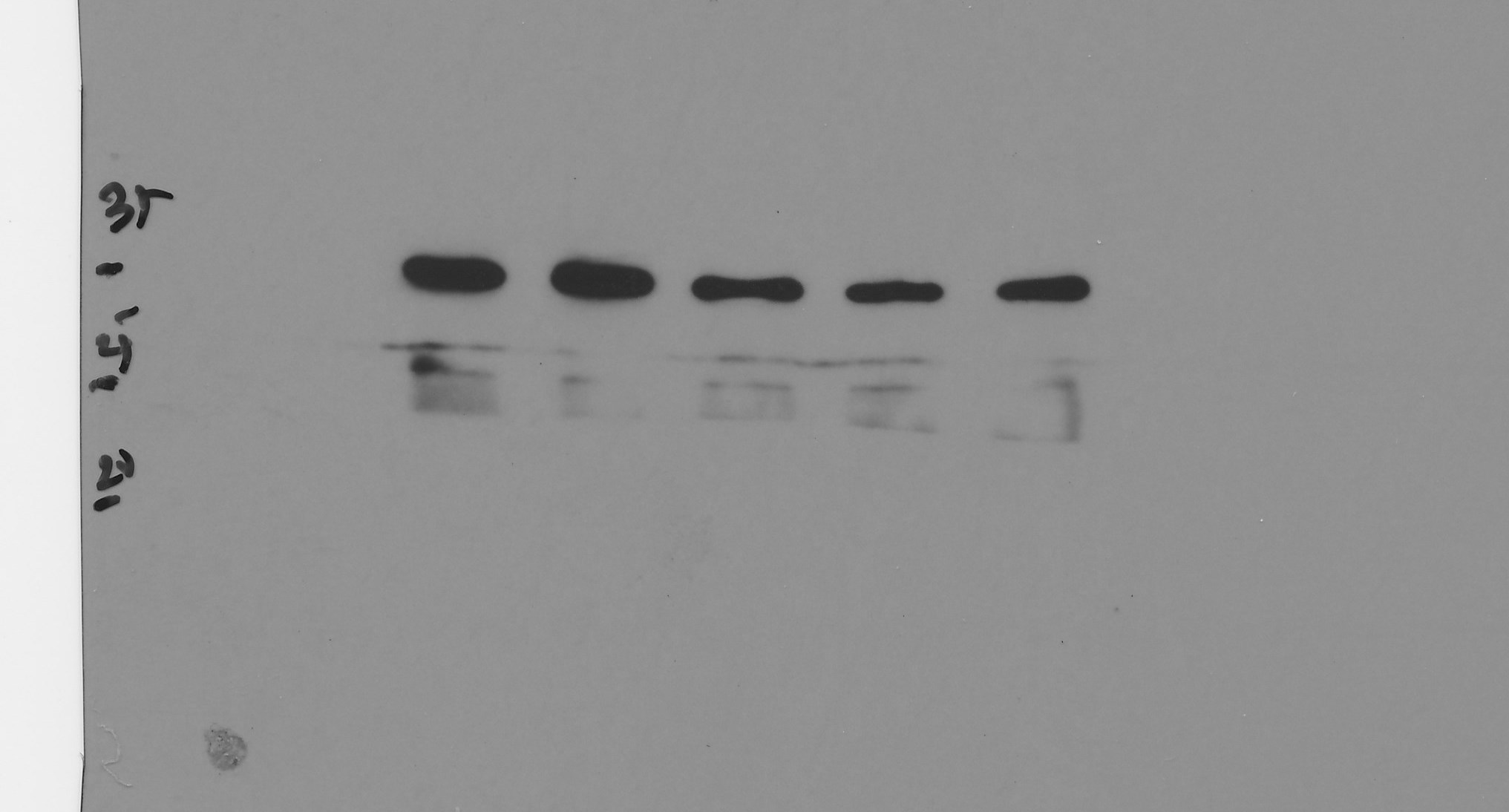

Supplement: Supplementary file 12 — Appendix Source Data [file 44319_2024_64_MOESM12_ESM.zip › Figure S1/1B/WCL IB GAPDH.jpg]

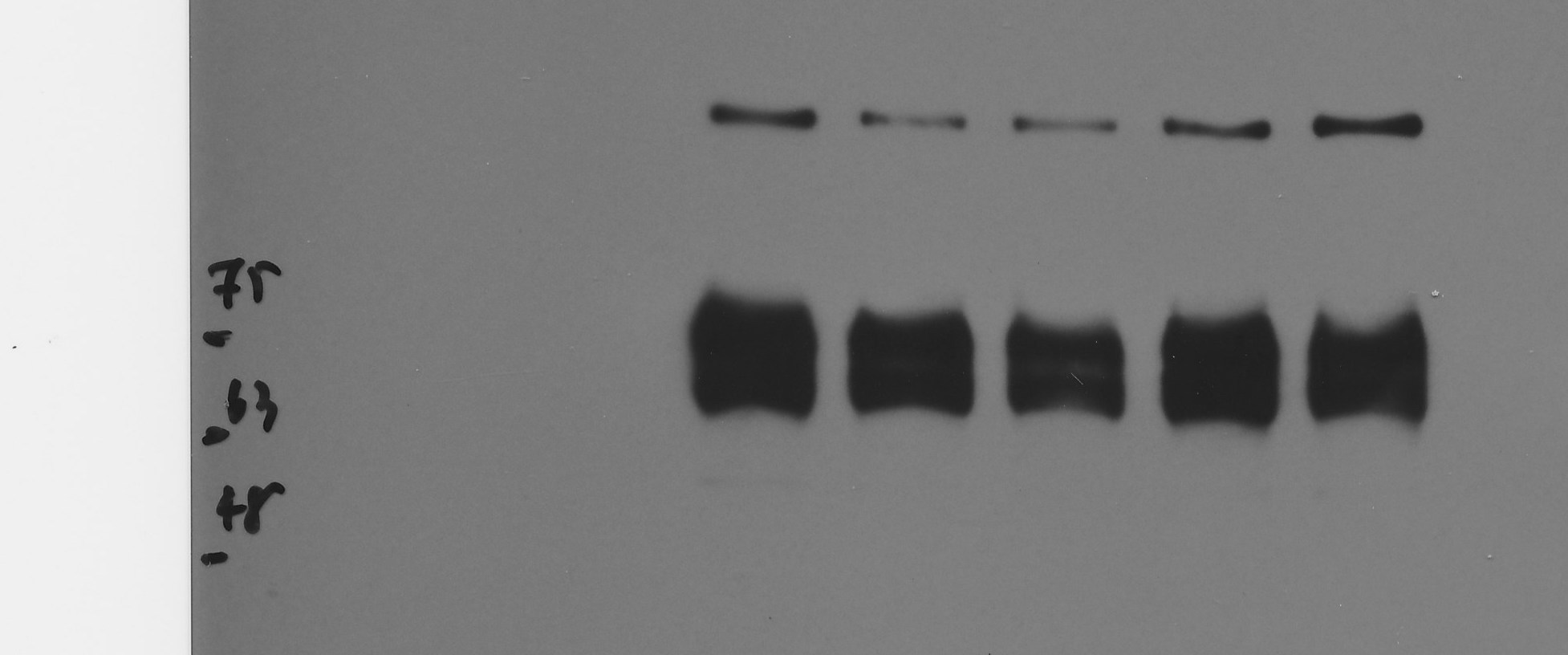

Supplement: Supplementary file 12 — Appendix Source Data [file 44319_2024_64_MOESM12_ESM.zip › Figure S1/1B/WCL IB p75NTR.jpg]

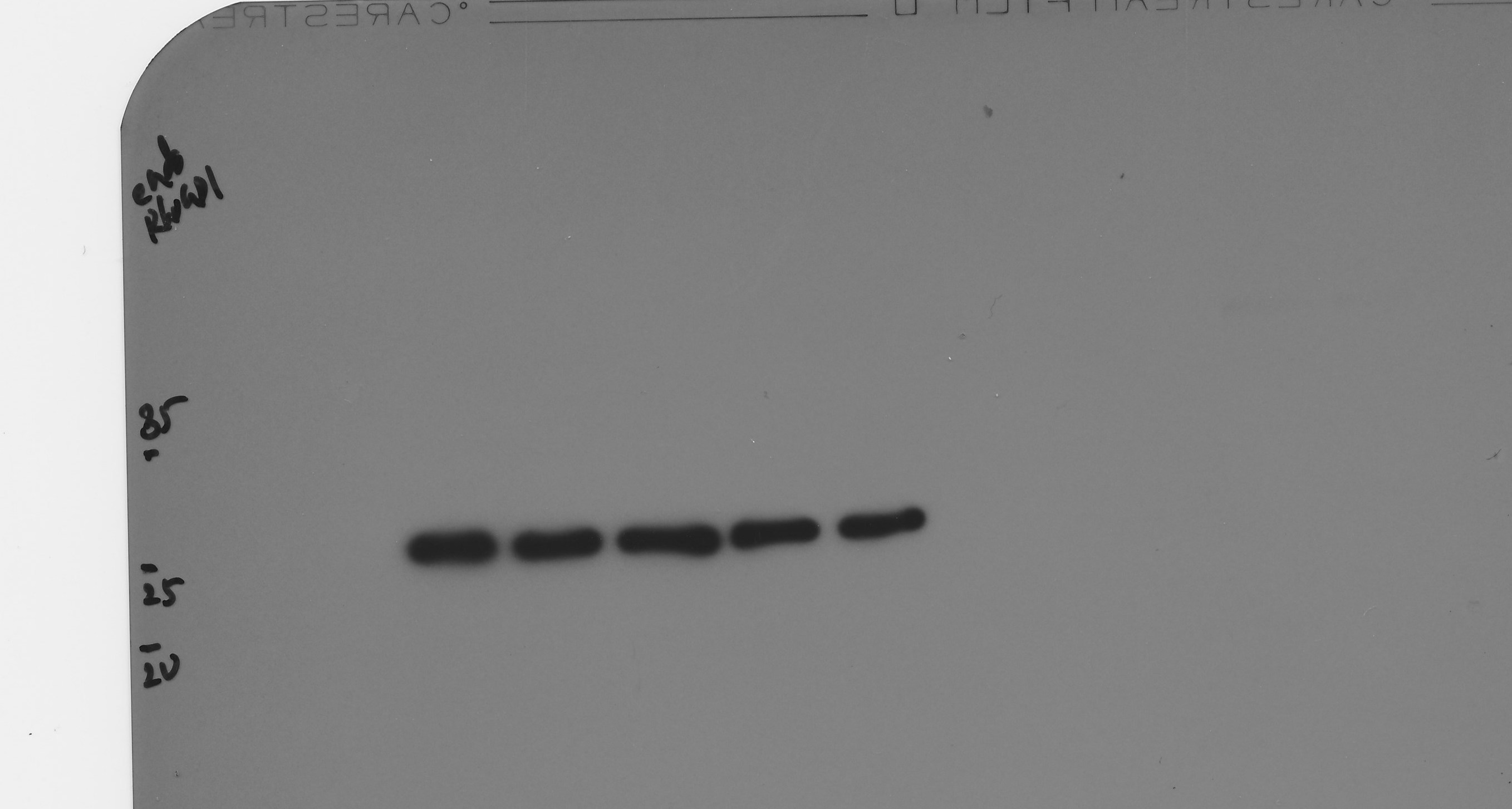

Supplement: Supplementary file 12 — Appendix Source Data [file 44319_2024_64_MOESM12_ESM.zip › Figure S1/1B/WCL IB RhoGDI.jpg]

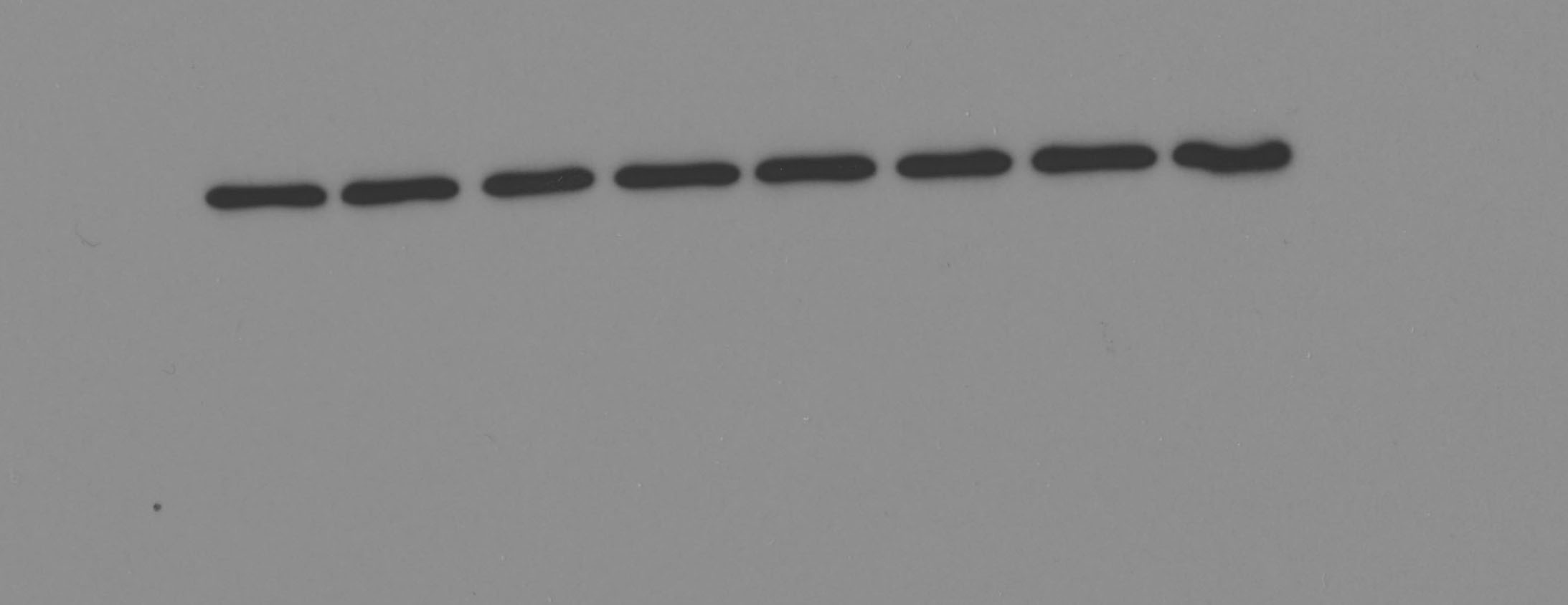

Supplement: Supplementary file 12 — Appendix Source Data [file 44319_2024_64_MOESM12_ESM.zip › Figure S1/1C/WCL IB GAPDH .jpg]

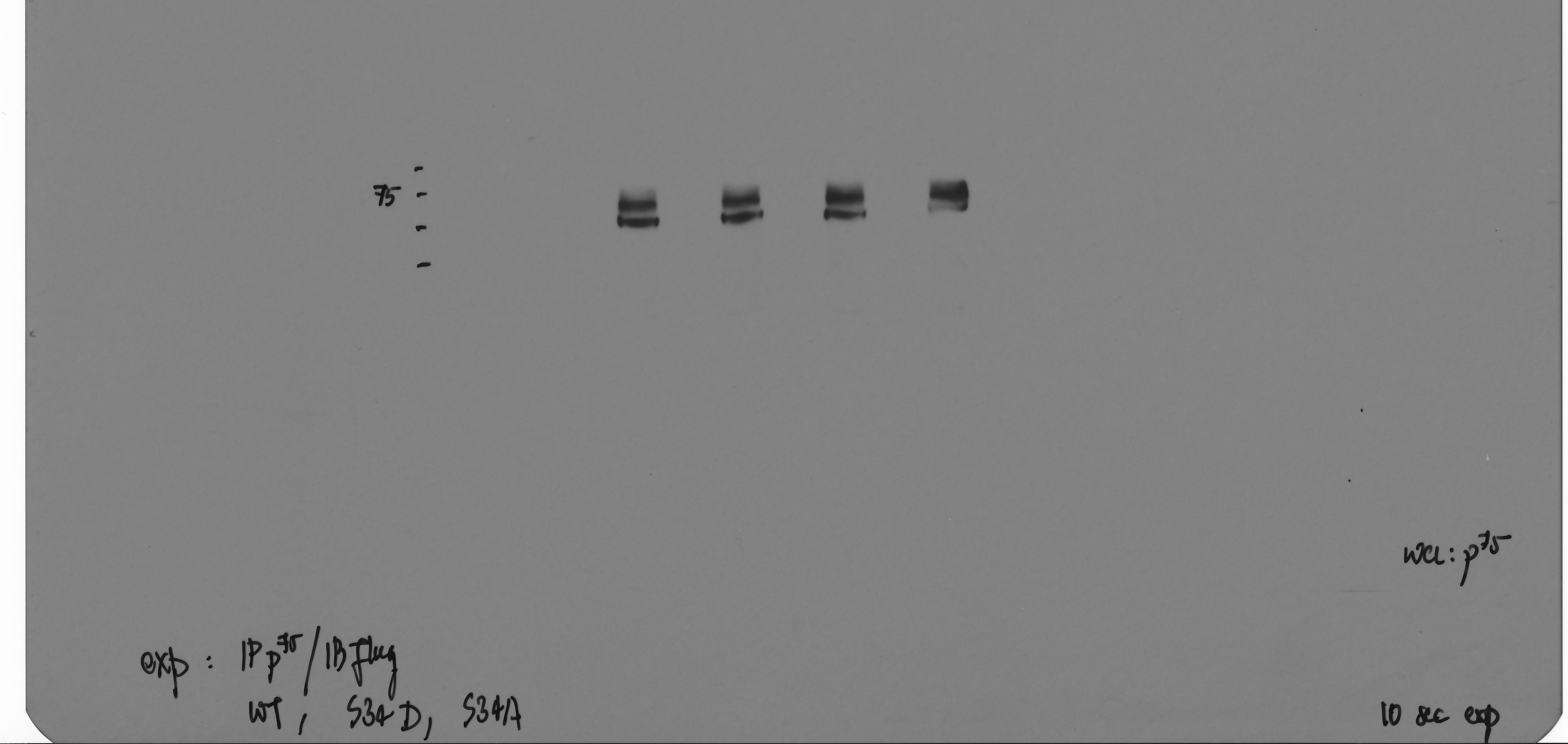

Supplement: Supplementary file 12 — Appendix Source Data [file 44319_2024_64_MOESM12_ESM.zip › Figure S1/1C/WCL IB p75NTR.jpg]

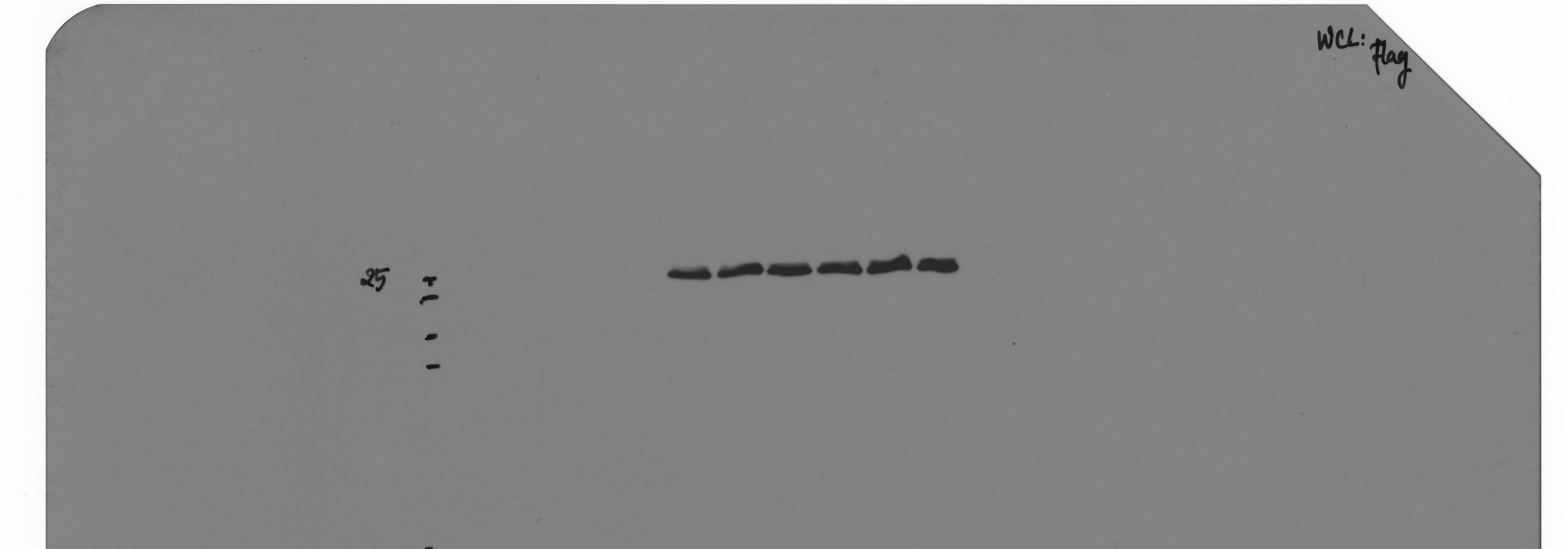

Supplement: Supplementary file 12 — Appendix Source Data [file 44319_2024_64_MOESM12_ESM.zip › Figure S1/1C/WCL IB RhoGDI.jpg]

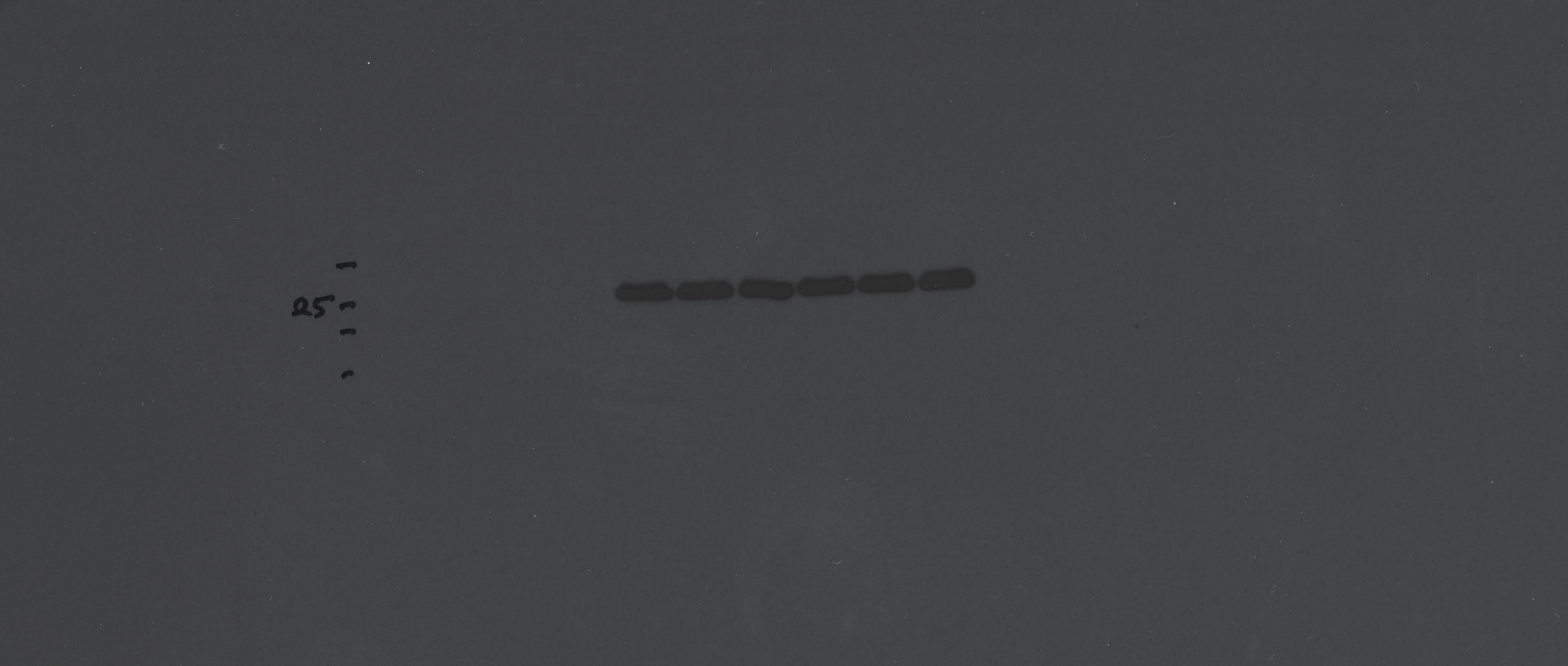

Supplement: Supplementary file 12 — Appendix Source Data [file 44319_2024_64_MOESM12_ESM.zip › Figure S1/1D/WCL IB Flag.jpg]

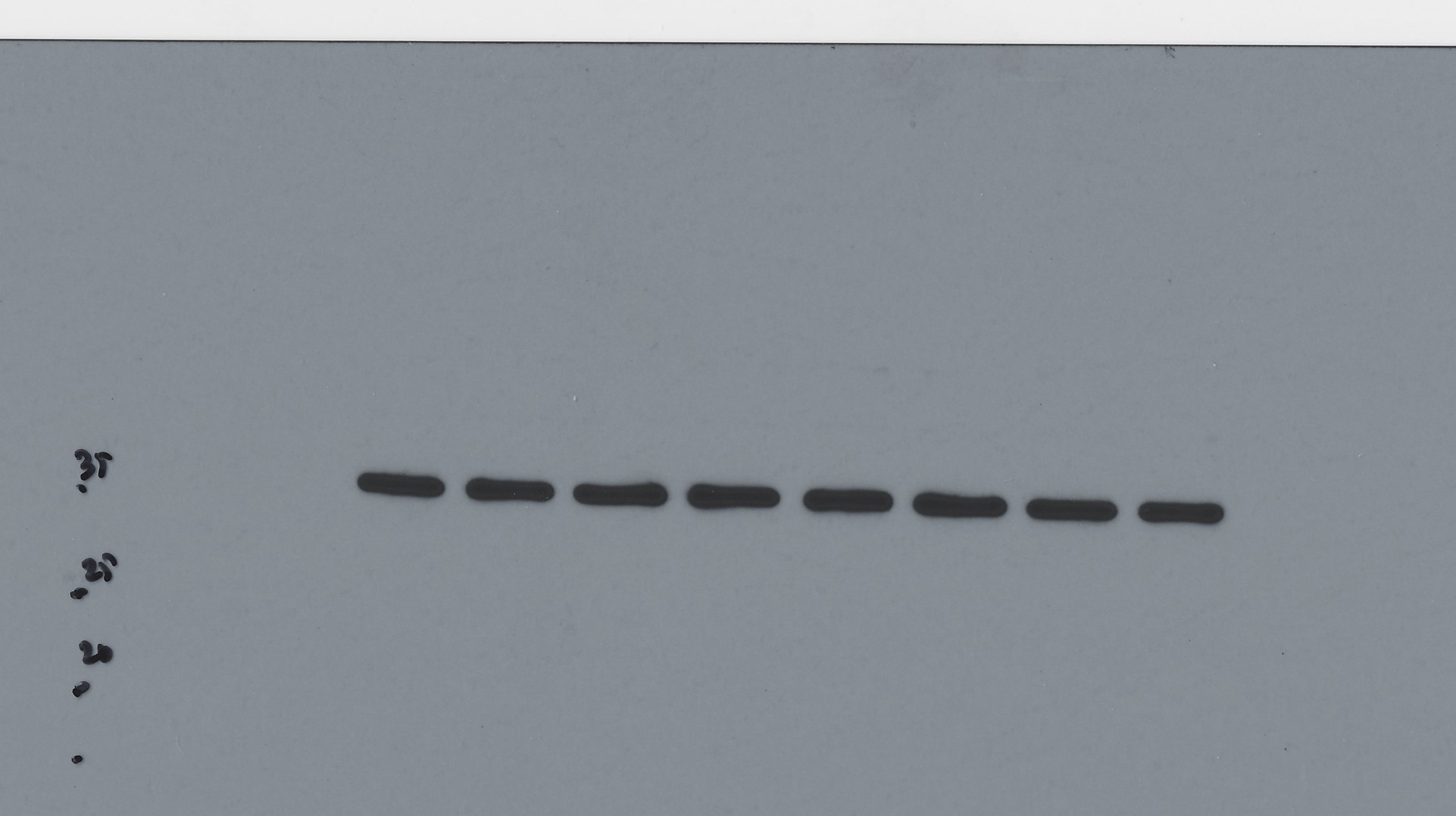

Supplement: Supplementary file 12 — Appendix Source Data [file 44319_2024_64_MOESM12_ESM.zip › Figure S1/1D/WCL IB GAPDH.jpg]

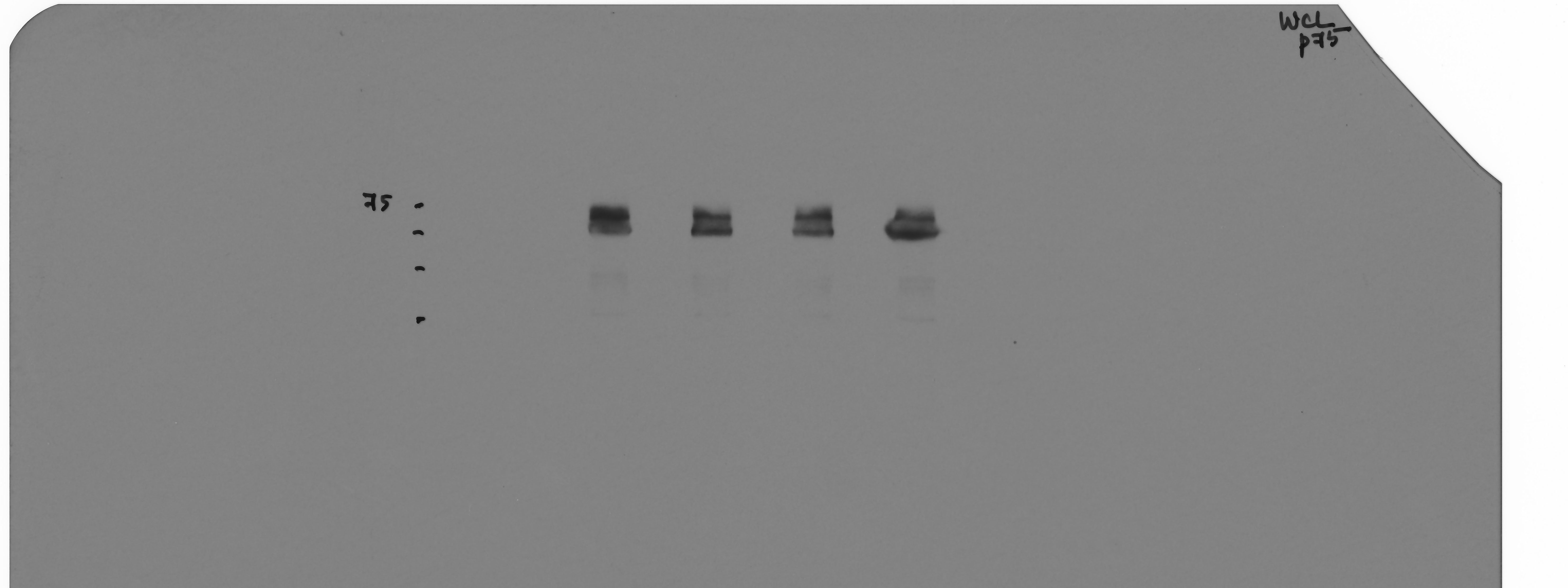

Supplement: Supplementary file 12 — Appendix Source Data [file 44319_2024_64_MOESM12_ESM.zip › Figure S1/1D/WCL IB p75NTR.jpg]

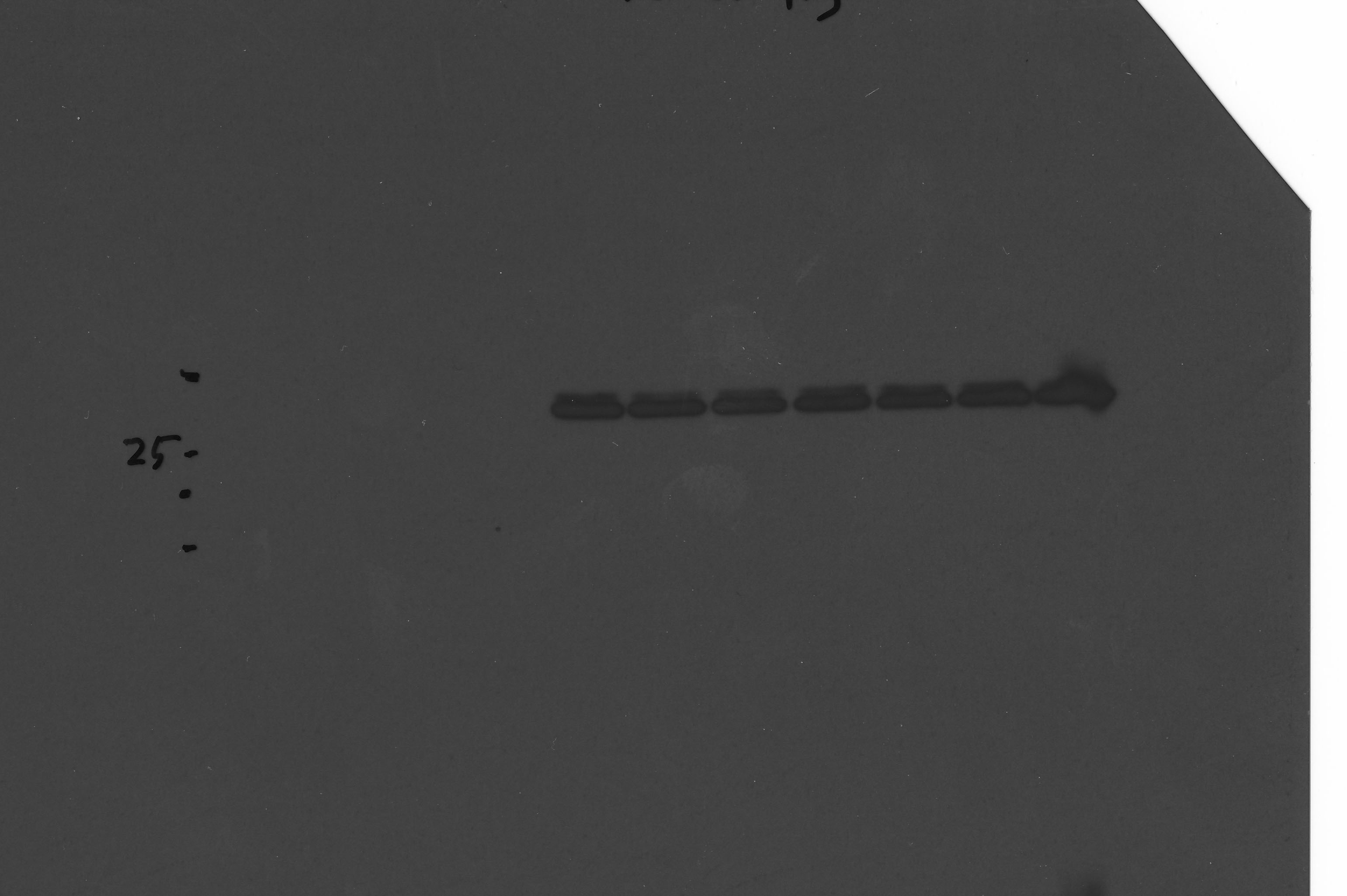

Supplement: Supplementary file 12 — Appendix Source Data [file 44319_2024_64_MOESM12_ESM.zip › Figure S1/1E/WCL IB flag.jpg]

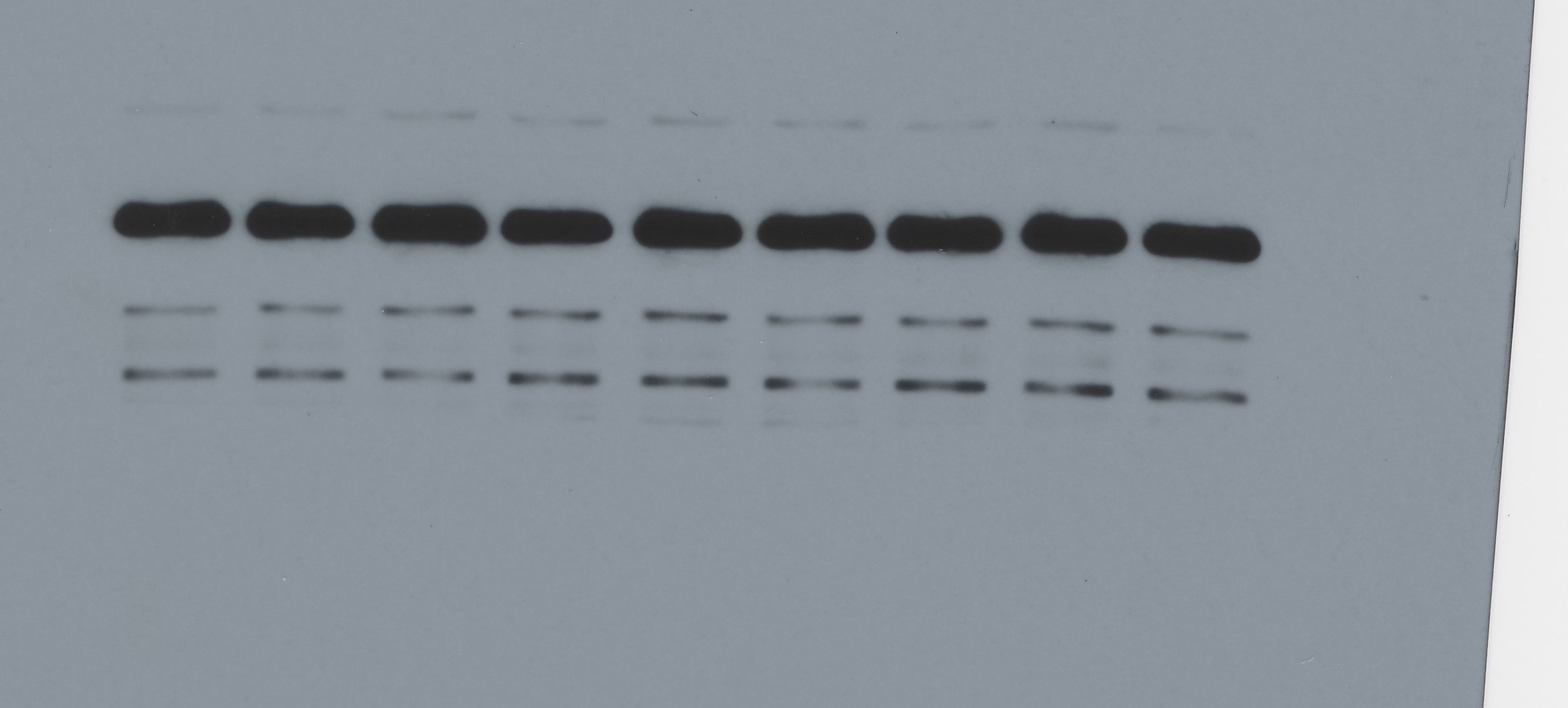

Supplement: Supplementary file 12 — Appendix Source Data [file 44319_2024_64_MOESM12_ESM.zip › Figure S1/1E/WCL IB GAPDH.jpg]

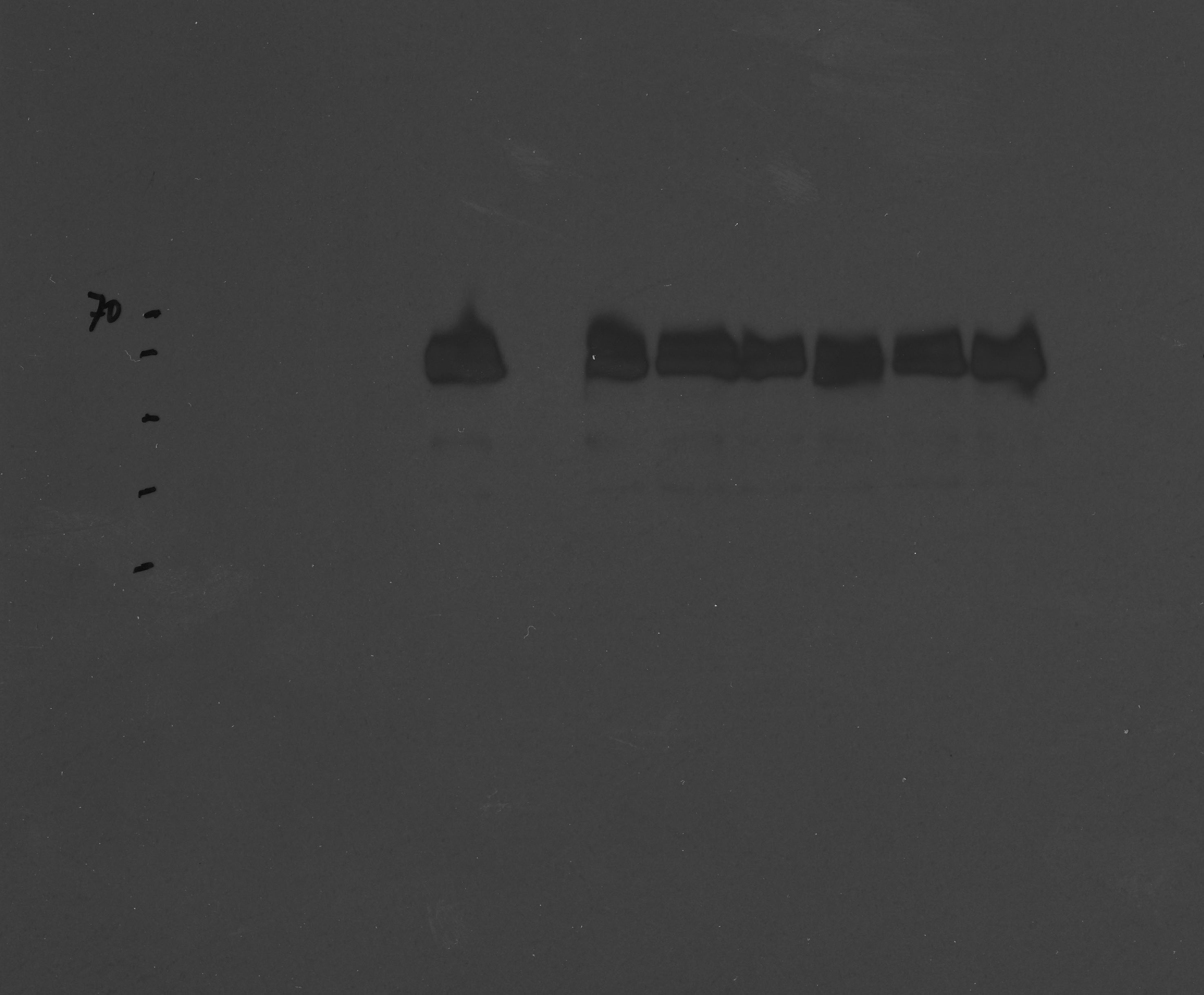

Supplement: Supplementary file 12 — Appendix Source Data [file 44319_2024_64_MOESM12_ESM.zip › Figure S1/1E/WCL IN p75NTR.jpg]

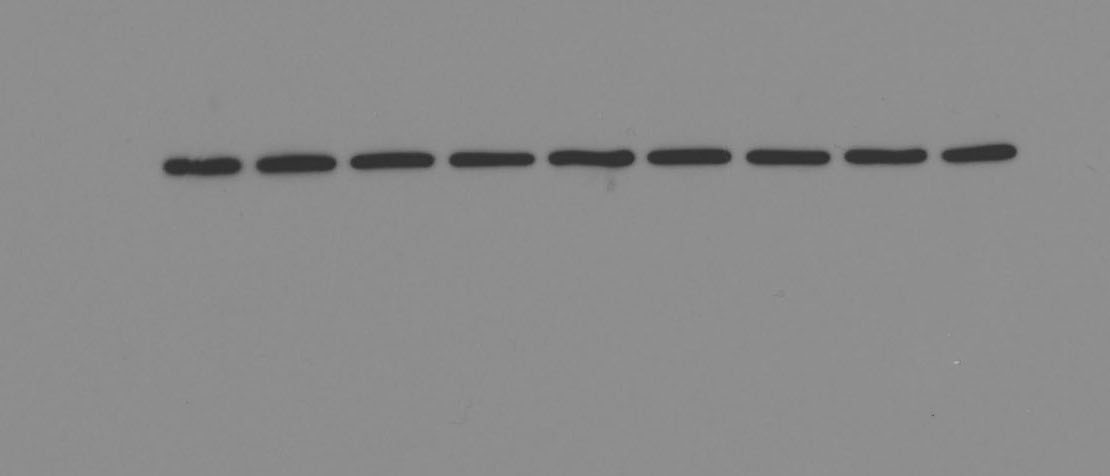

Supplement: Supplementary file 12 — Appendix Source Data [file 44319_2024_64_MOESM12_ESM.zip › Figure S1/1F/WCL IB GAPDH.jpg]

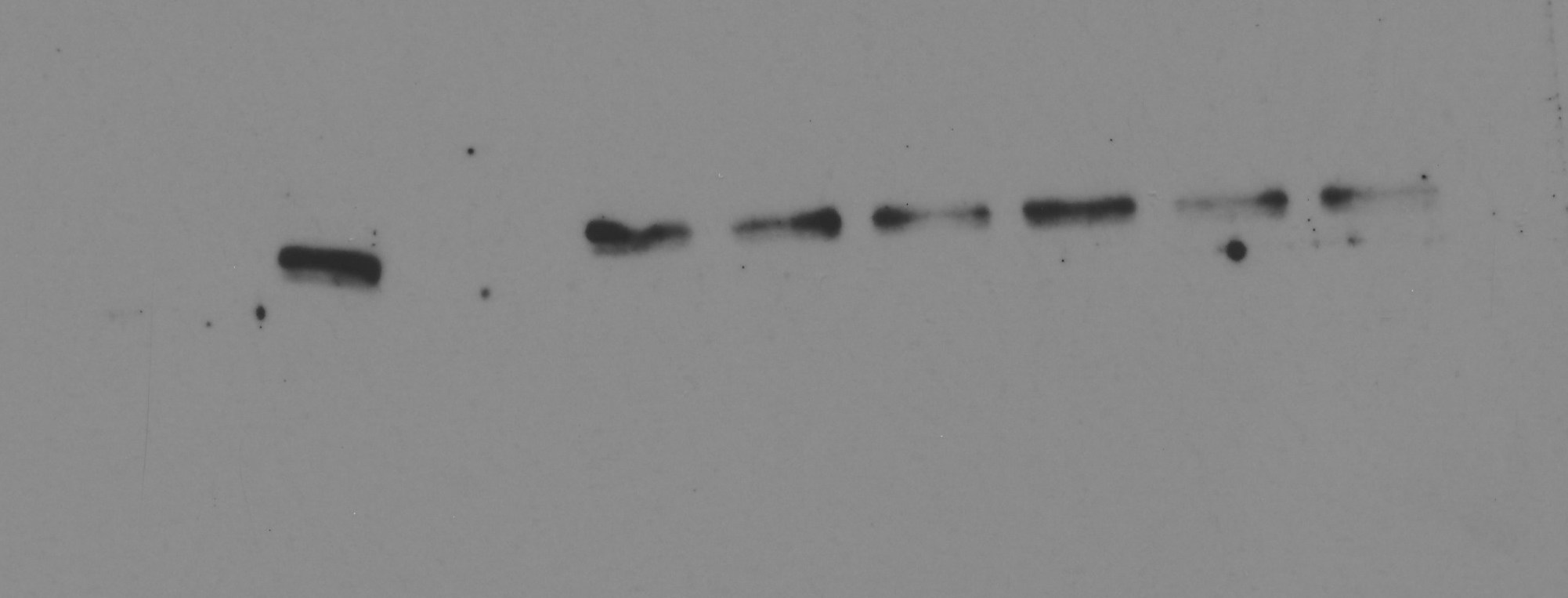

Supplement: Supplementary file 12 — Appendix Source Data [file 44319_2024_64_MOESM12_ESM.zip › Figure S1/1F/WCL IB p75NTR.jpg]

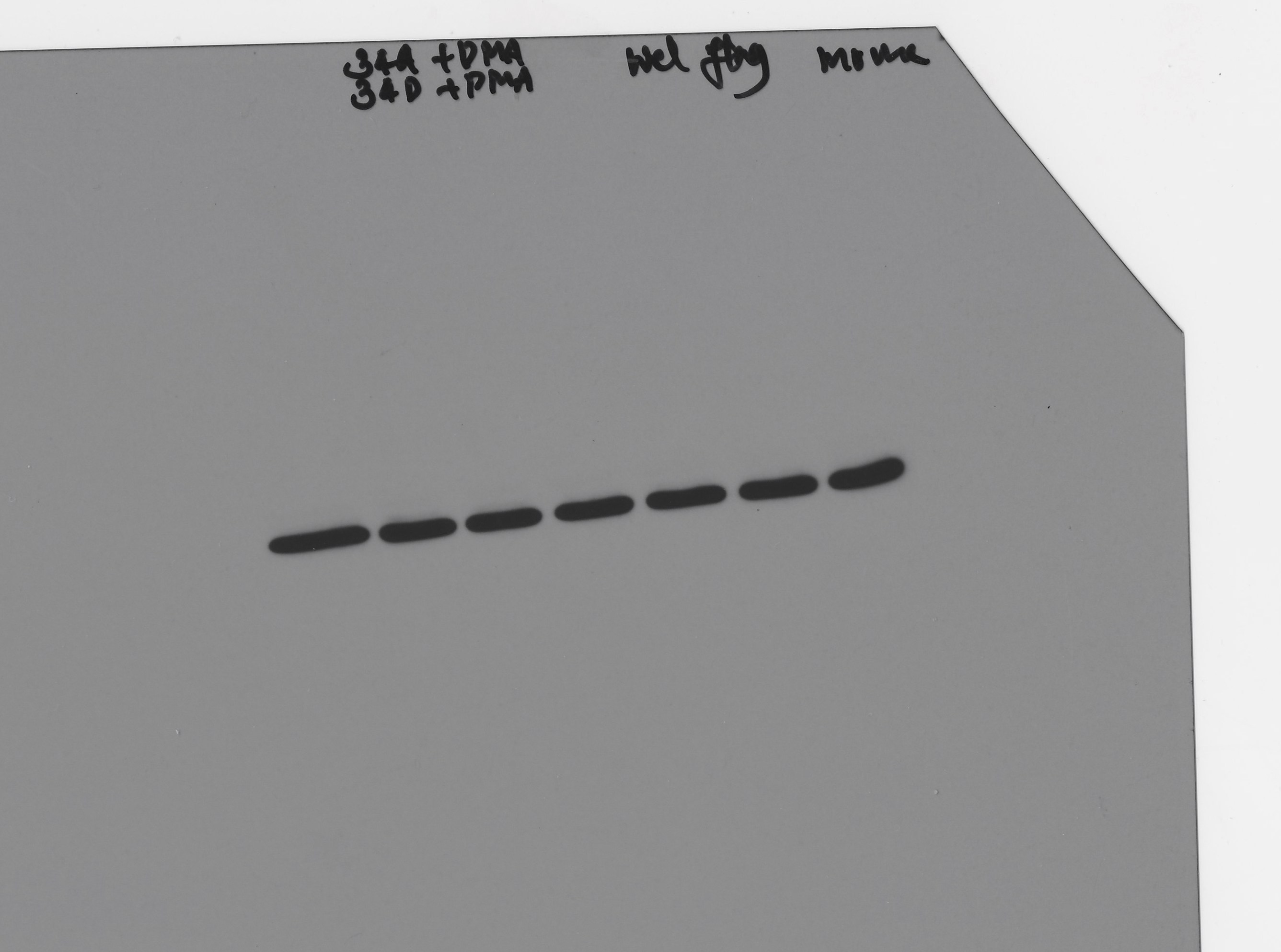

Supplement: Supplementary file 12 — Appendix Source Data [file 44319_2024_64_MOESM12_ESM.zip › Figure S1/1F/WCL IB RhoGDI.jpg]

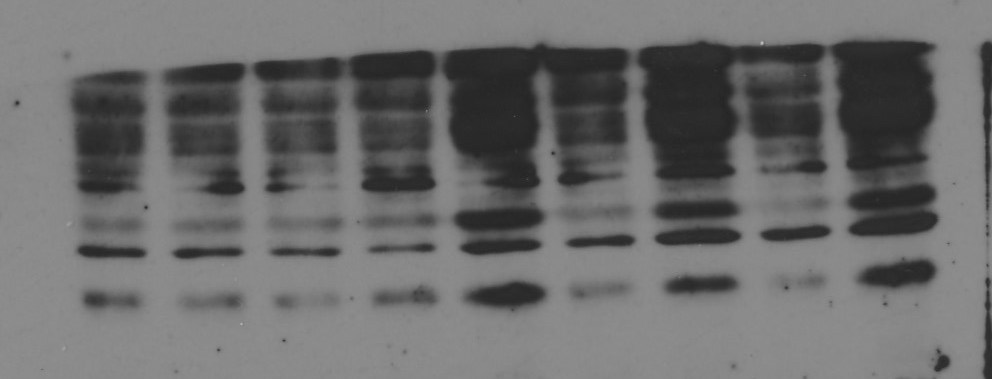

Supplement: Supplementary file 12 — Appendix Source Data [file 44319_2024_64_MOESM12_ESM.zip › Figure S1/1F/WCL P Ser PKC-alpha substrate antibody.jpg]

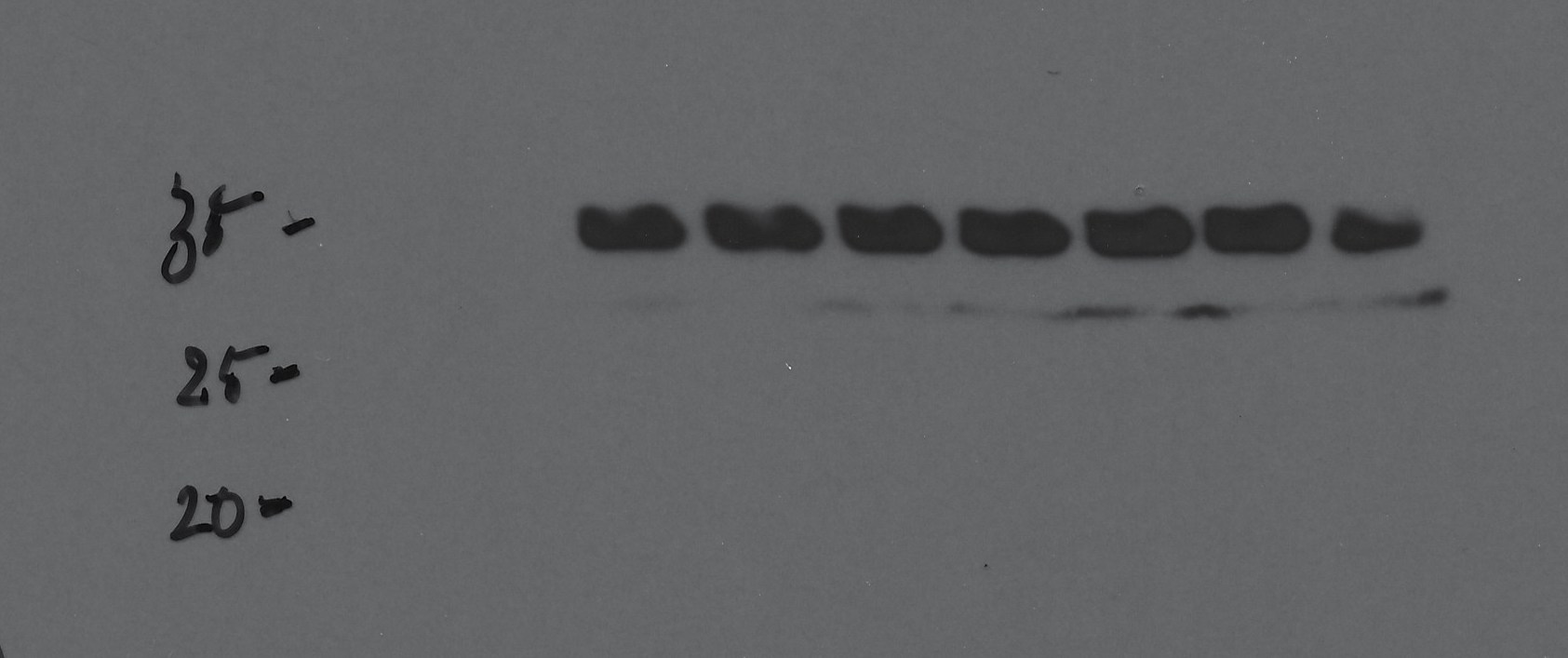

Supplement: Supplementary file 12 — Appendix Source Data [file 44319_2024_64_MOESM12_ESM.zip › Figure S1/1G/WCL IB GAPDH.jpg]

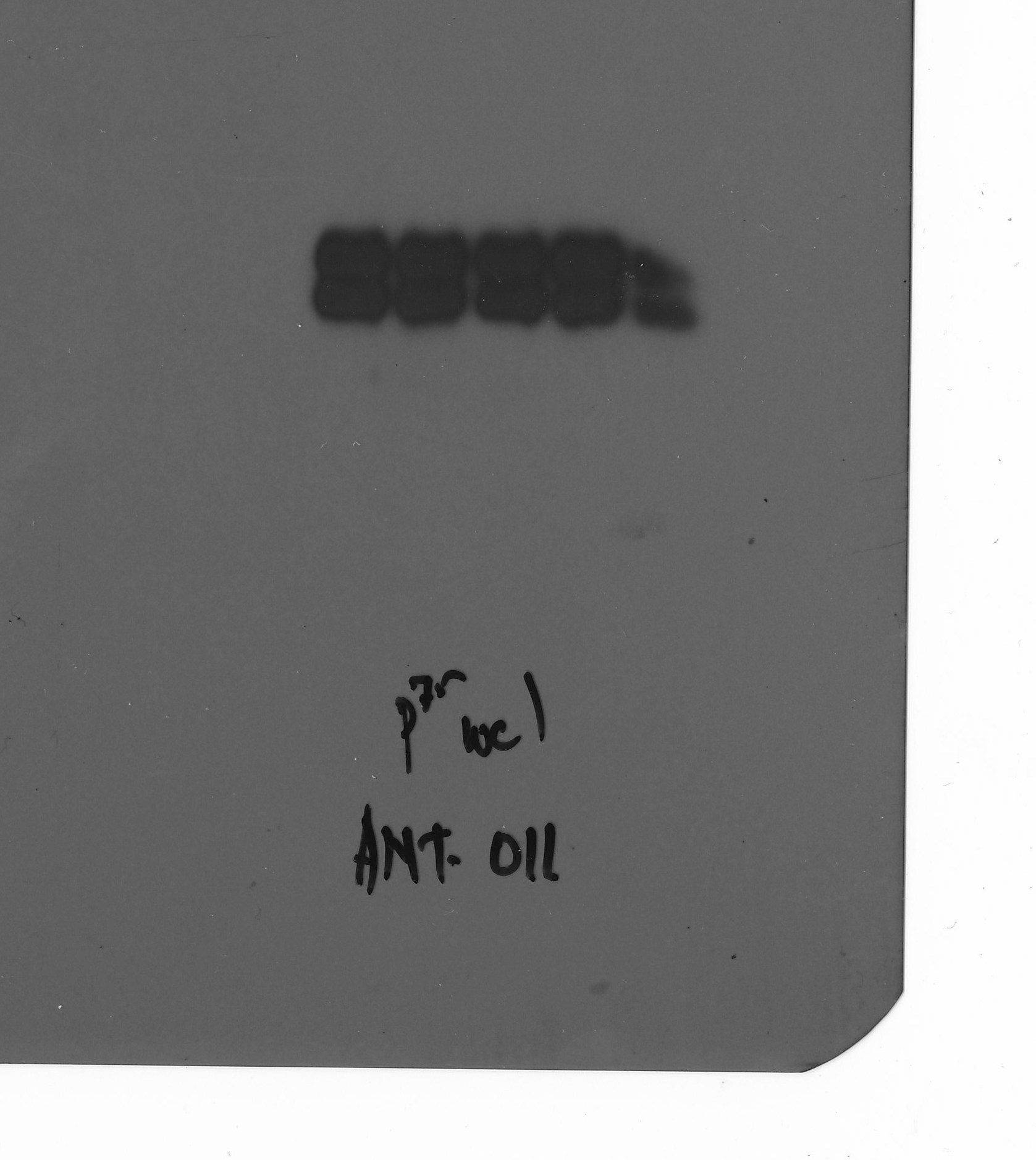

Supplement: Supplementary file 12 — Appendix Source Data [file 44319_2024_64_MOESM12_ESM.zip › Figure S1/1G/WCL IB p75NTR.jpg]

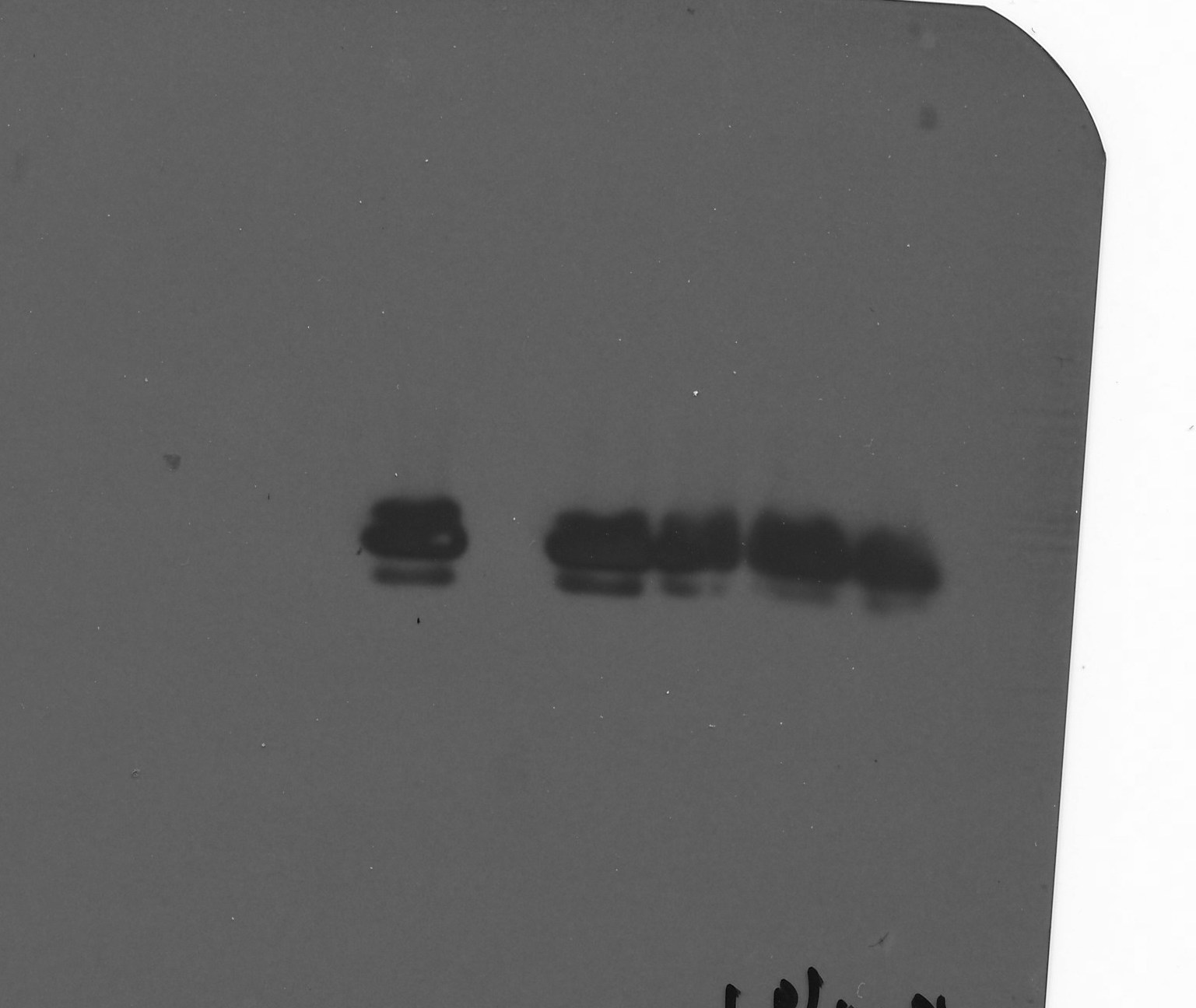

Supplement: Supplementary file 12 — Appendix Source Data [file 44319_2024_64_MOESM12_ESM.zip › Figure S1/1G/WCL IB RhoGDI.jpg]

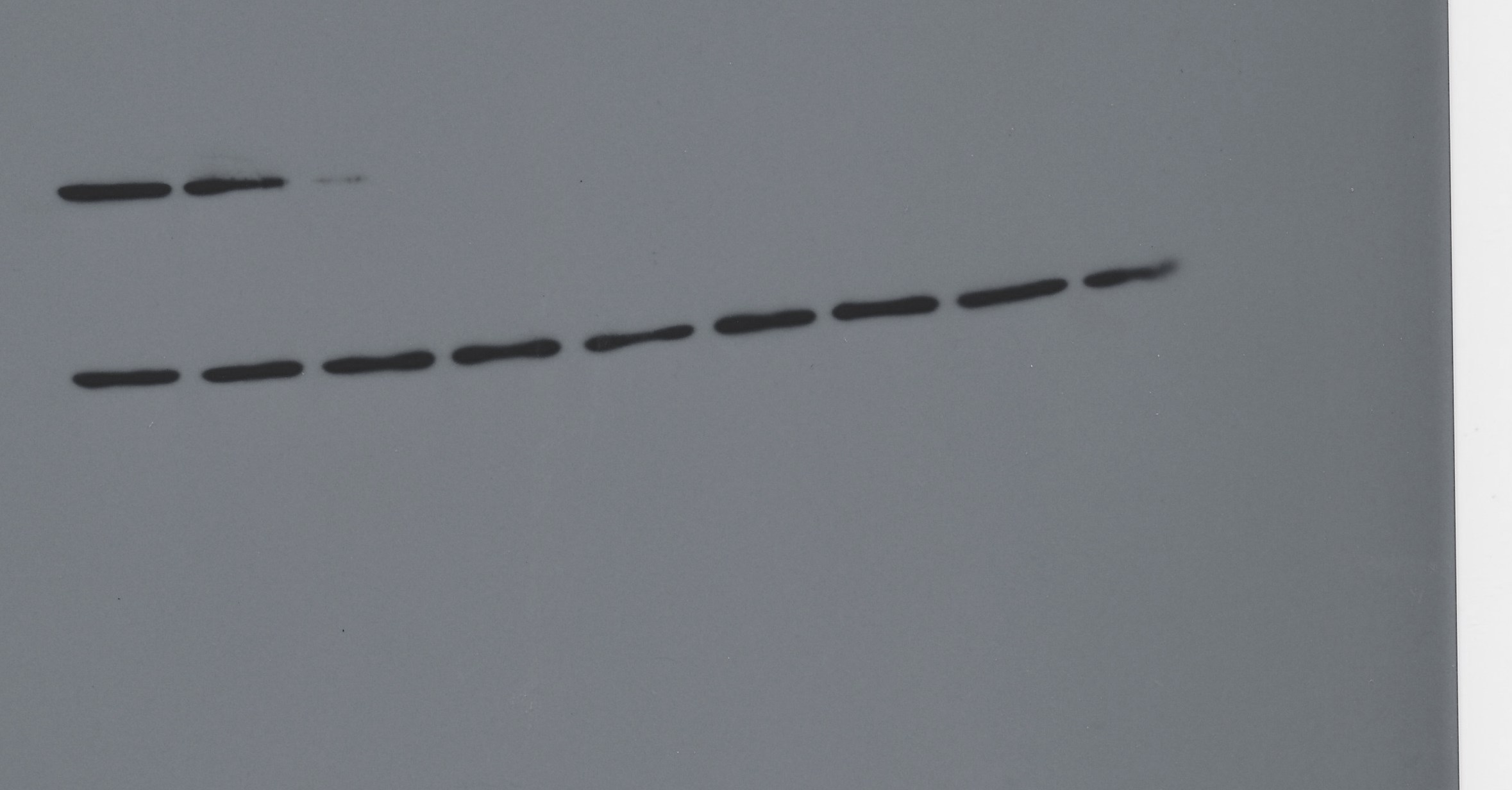

Supplement: Supplementary file 12 — Appendix Source Data [file 44319_2024_64_MOESM12_ESM.zip › Figure S3/3A/WCL IB GAPDH.jpg]

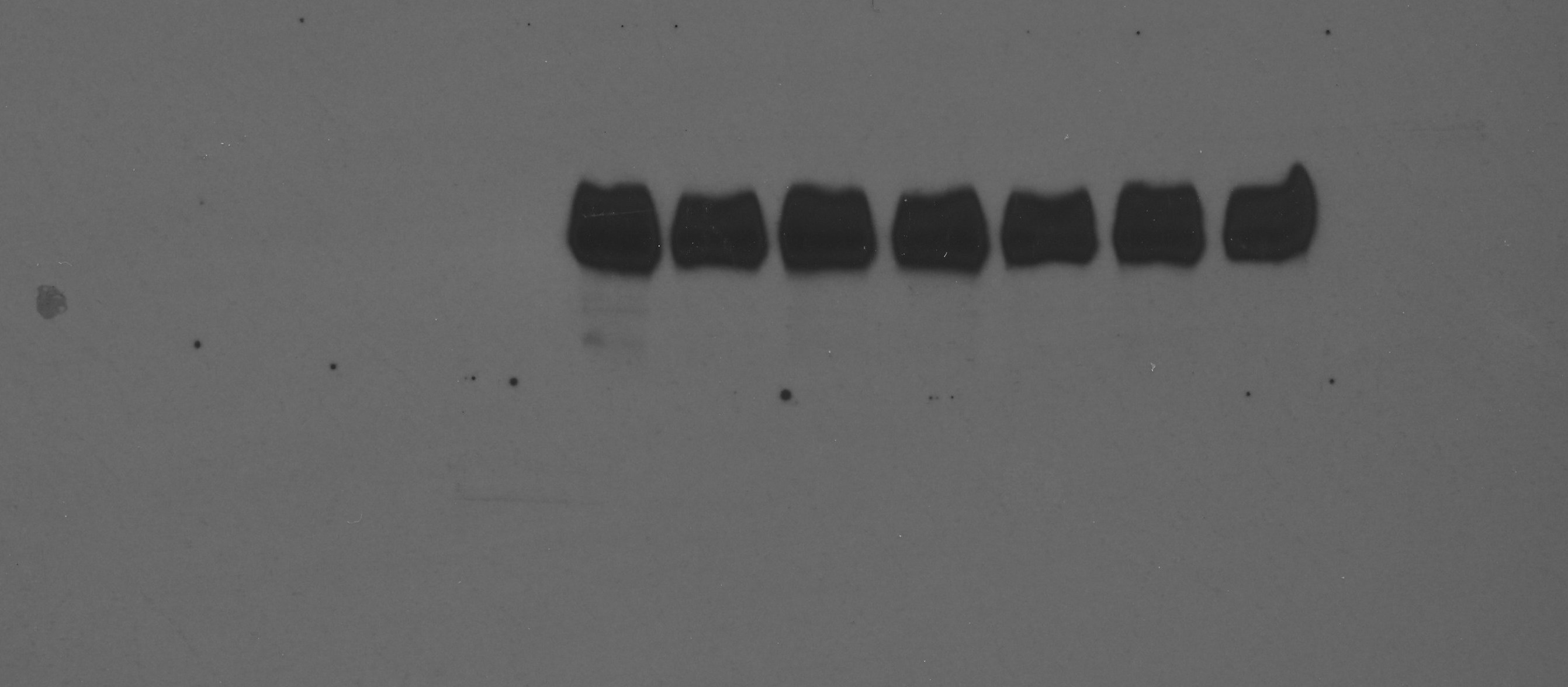

Supplement: Supplementary file 12 — Appendix Source Data [file 44319_2024_64_MOESM12_ESM.zip › Figure S3/3A/WCL IB p75NTR.jpg]

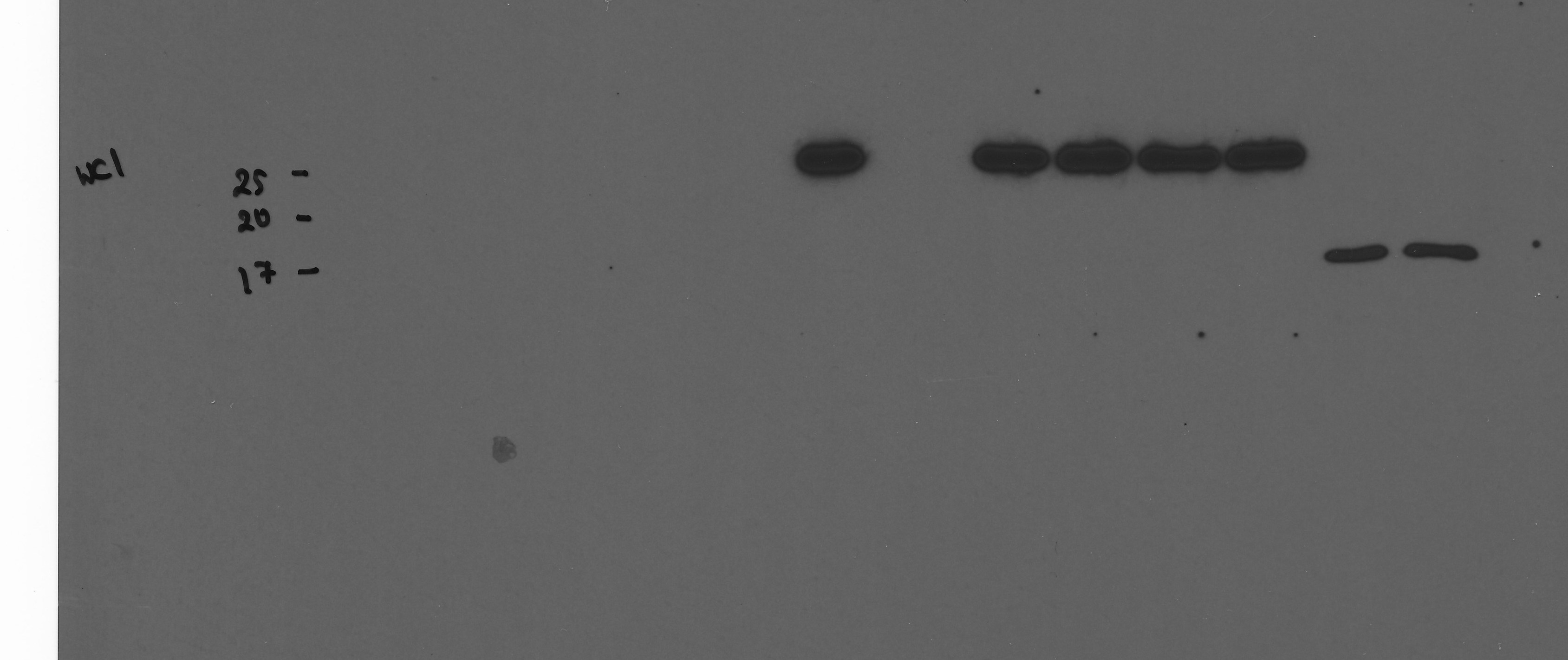

Supplement: Supplementary file 12 — Appendix Source Data [file 44319_2024_64_MOESM12_ESM.zip › Figure S3/3A/WCL IB RhoGDI.jpg]

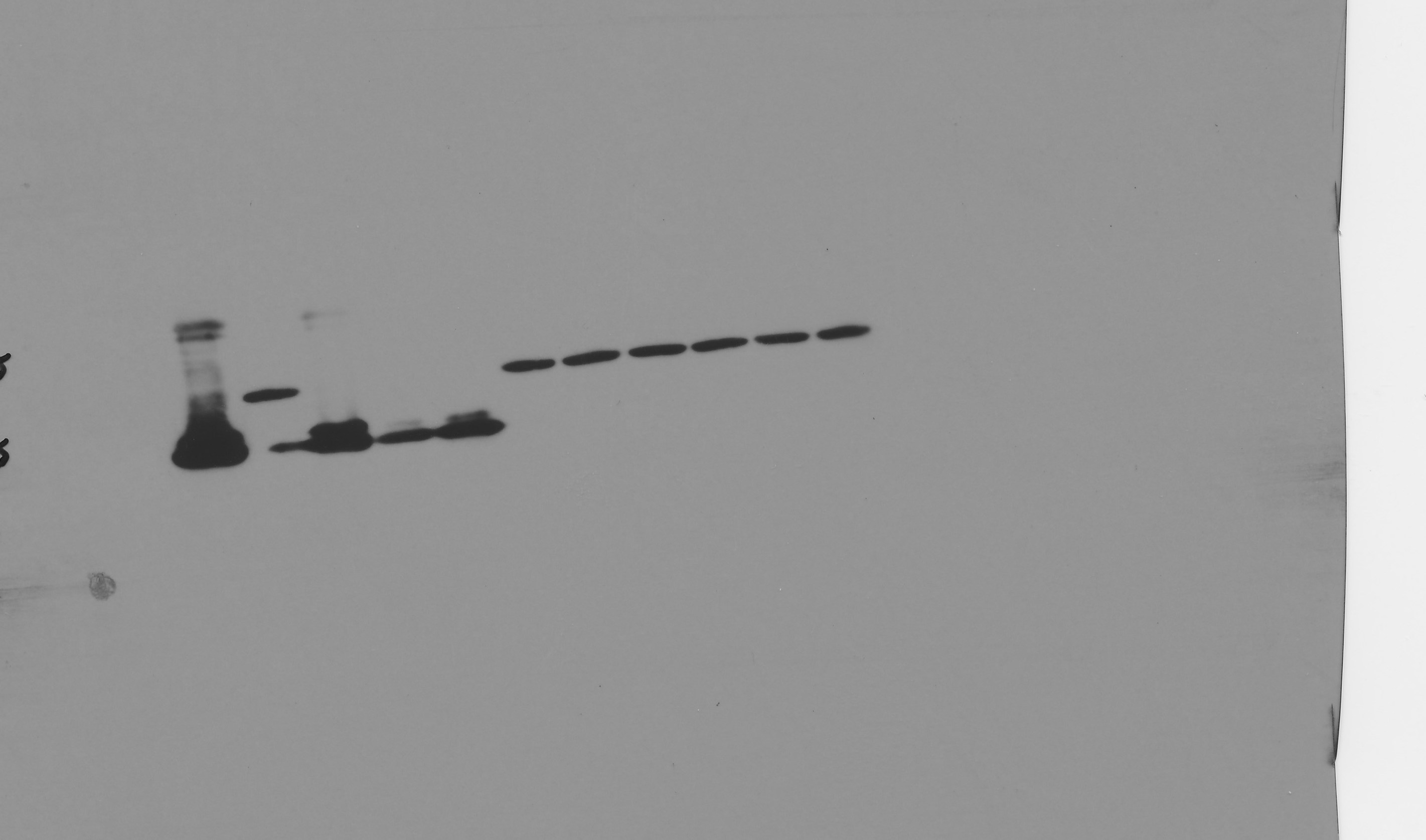

Supplement: Supplementary file 12 — Appendix Source Data [file 44319_2024_64_MOESM12_ESM.zip › Figure S3/3B/WCL IB Flag (RhoGDI).jpg]

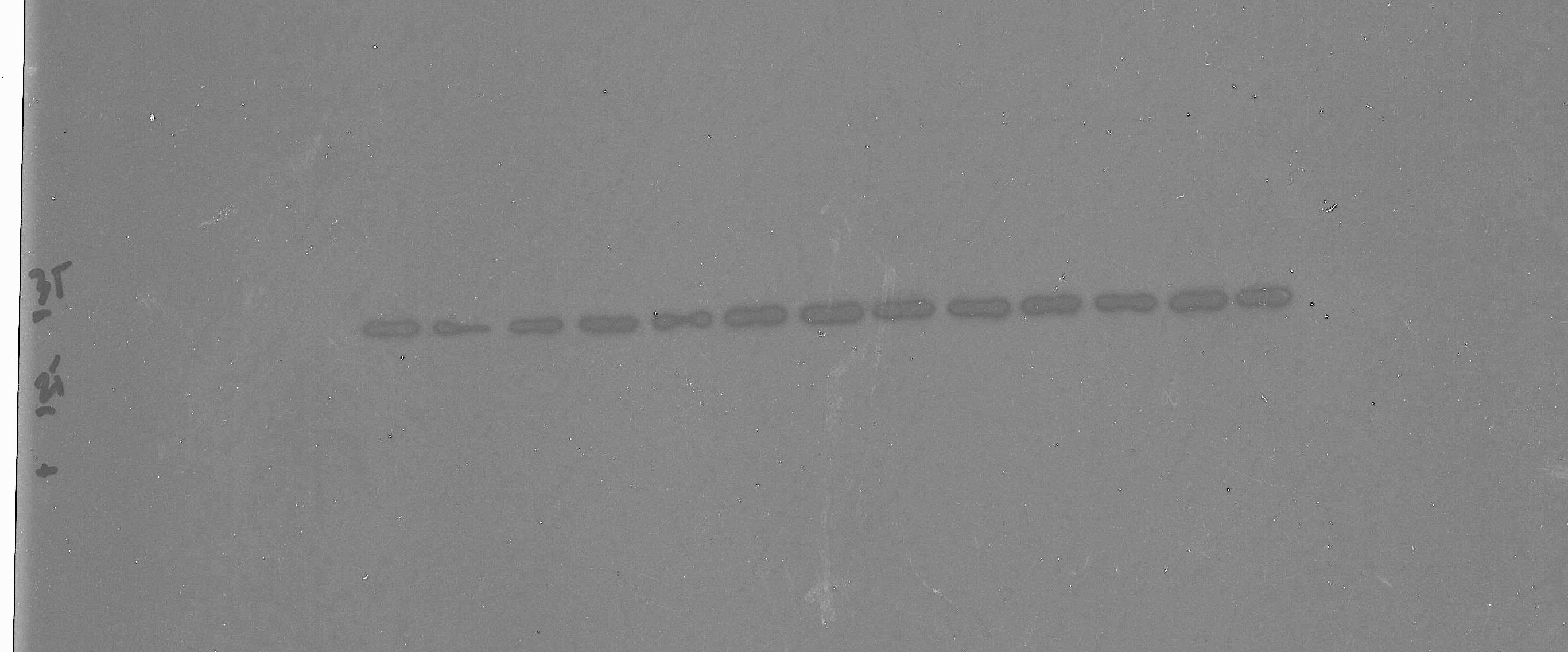

Supplement: Supplementary file 12 — Appendix Source Data [file 44319_2024_64_MOESM12_ESM.zip › Figure S3/3B/WCL IB GAPDH.jpg]

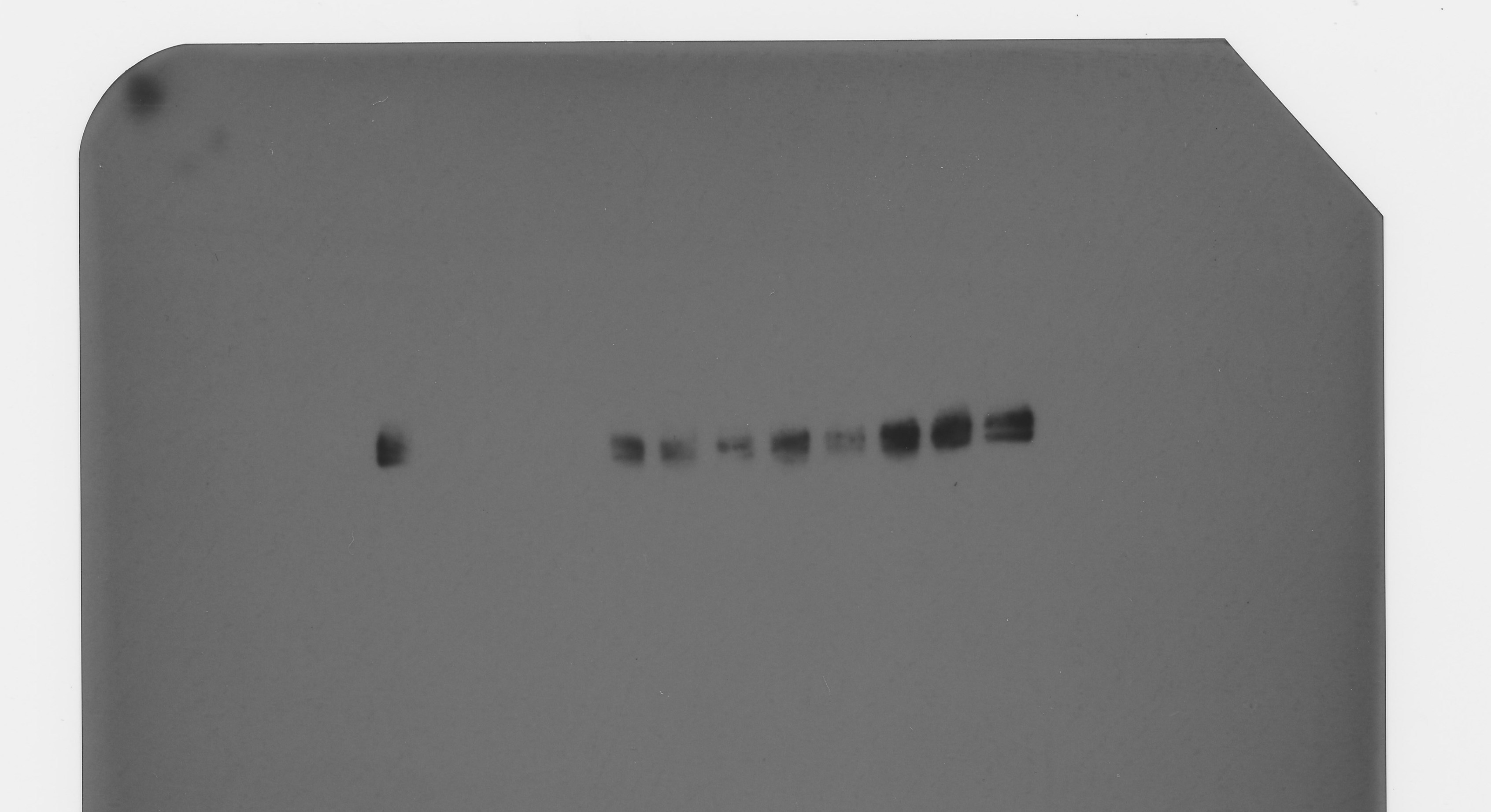

Supplement: Supplementary file 12 — Appendix Source Data [file 44319_2024_64_MOESM12_ESM.zip › Figure S3/3B/WCL IB p75NTR.jpg]

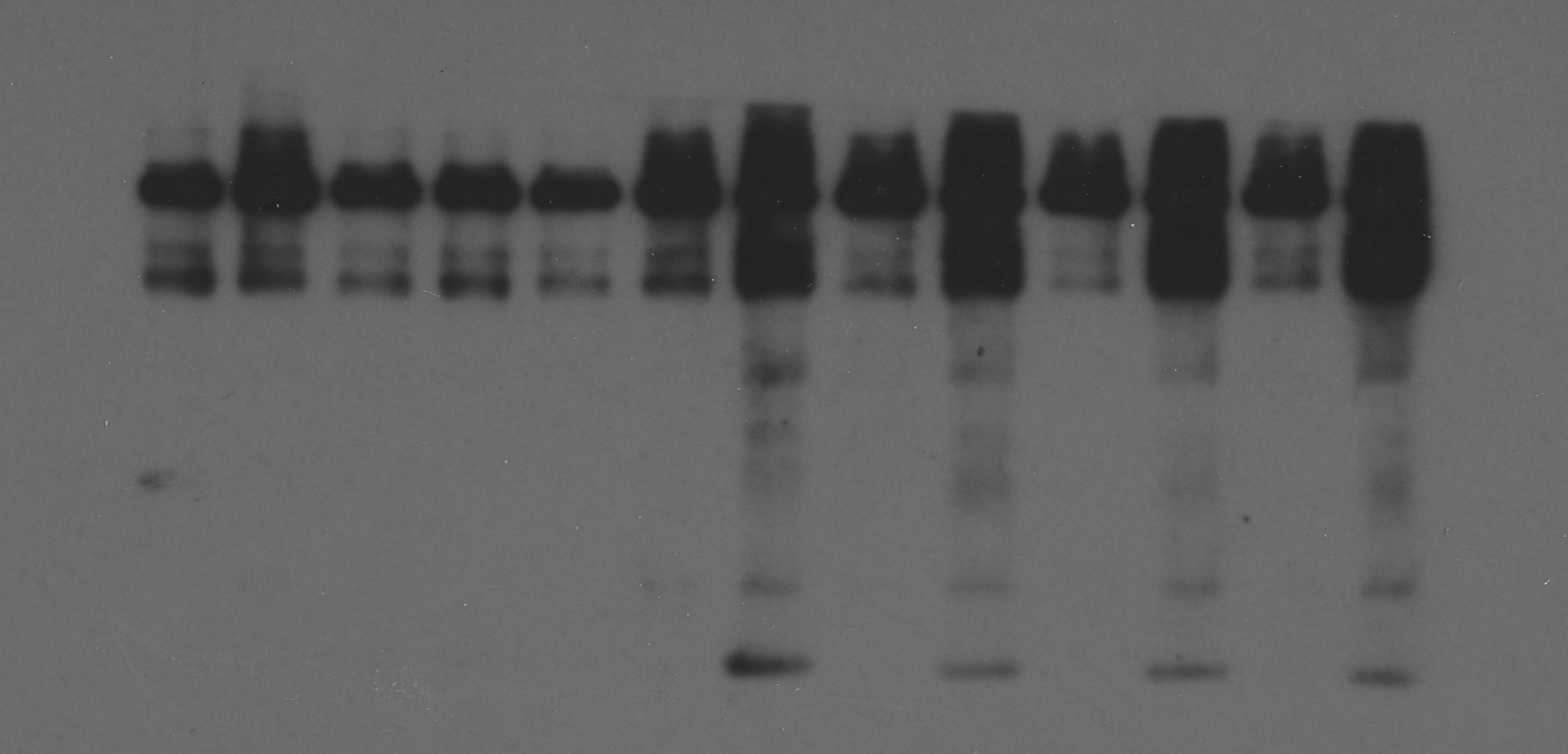

Supplement: Supplementary file 12 — Appendix Source Data [file 44319_2024_64_MOESM12_ESM.zip › Figure S3/3B/WCL Phospho serine PKC substrate.jpg]

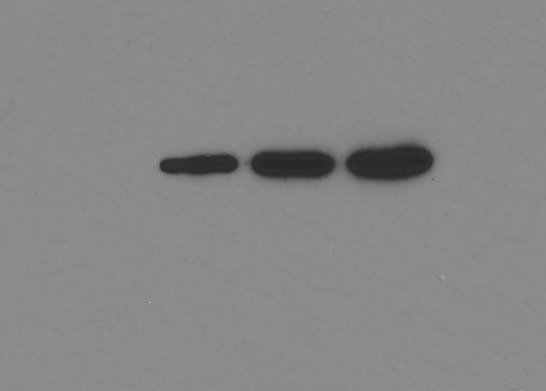

Supplement: Supplementary file 12 — Appendix Source Data [file 44319_2024_64_MOESM12_ESM.zip › Figure S3/3C/WCL IB GAPDH.jpg]
