# Supplementary figures and images for: RhoGDI phosphorylation by PKC promotes its interaction with death receptor p75NTR to gate axon growth and neuron survival (part 3 of 3)
Source: EMBO Rep. 2024 Jan 22;25(3):30. doi: 10.1038/s44319-024-00064-2 (PMC10933337; doi:10.1038/s44319-024-00064-2)

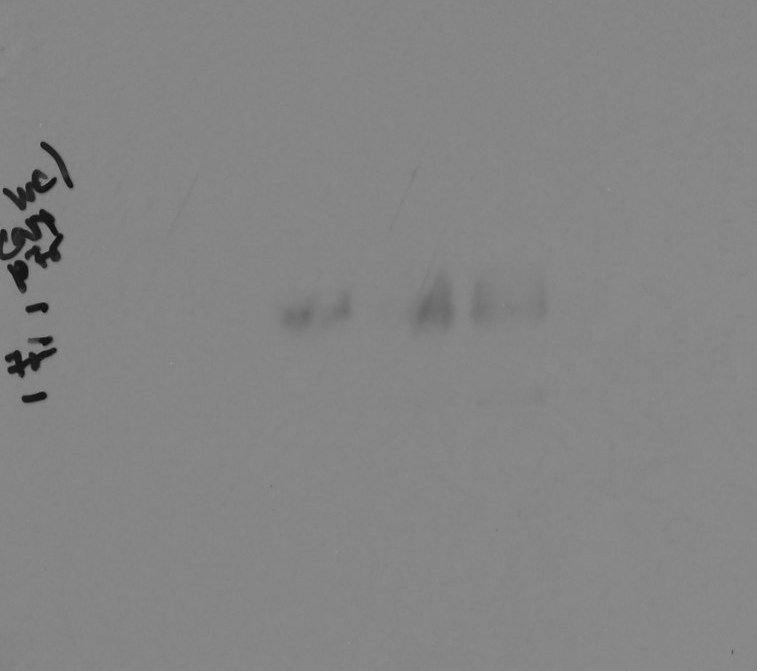

Supplement: Supplementary file 12 — Appendix Source Data [file 44319_2024_64_MOESM12_ESM.zip › Figure S3/3C/WCL IB p75NTR.jpg]

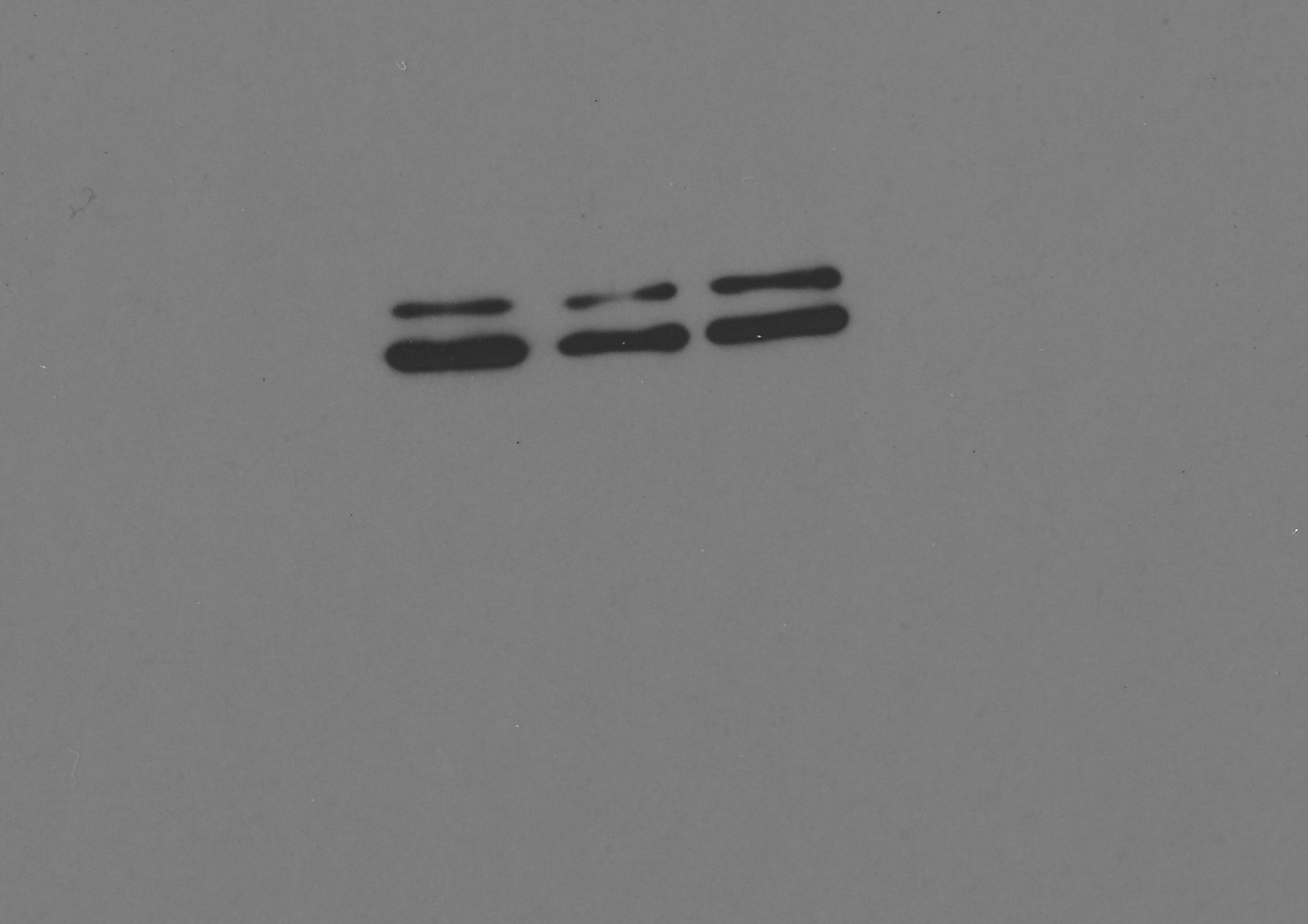

Supplement: Supplementary file 12 — Appendix Source Data [file 44319_2024_64_MOESM12_ESM.zip › Figure S3/3C/WCL IB RhoGDI.jpg]

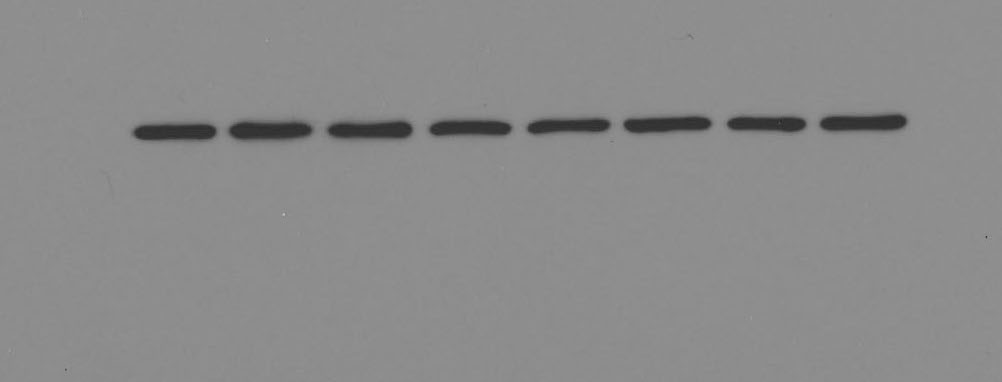

Supplement: Supplementary file 12 — Appendix Source Data [file 44319_2024_64_MOESM12_ESM.zip › Figure S2/2A/WCL IB GAPDH.jpg]

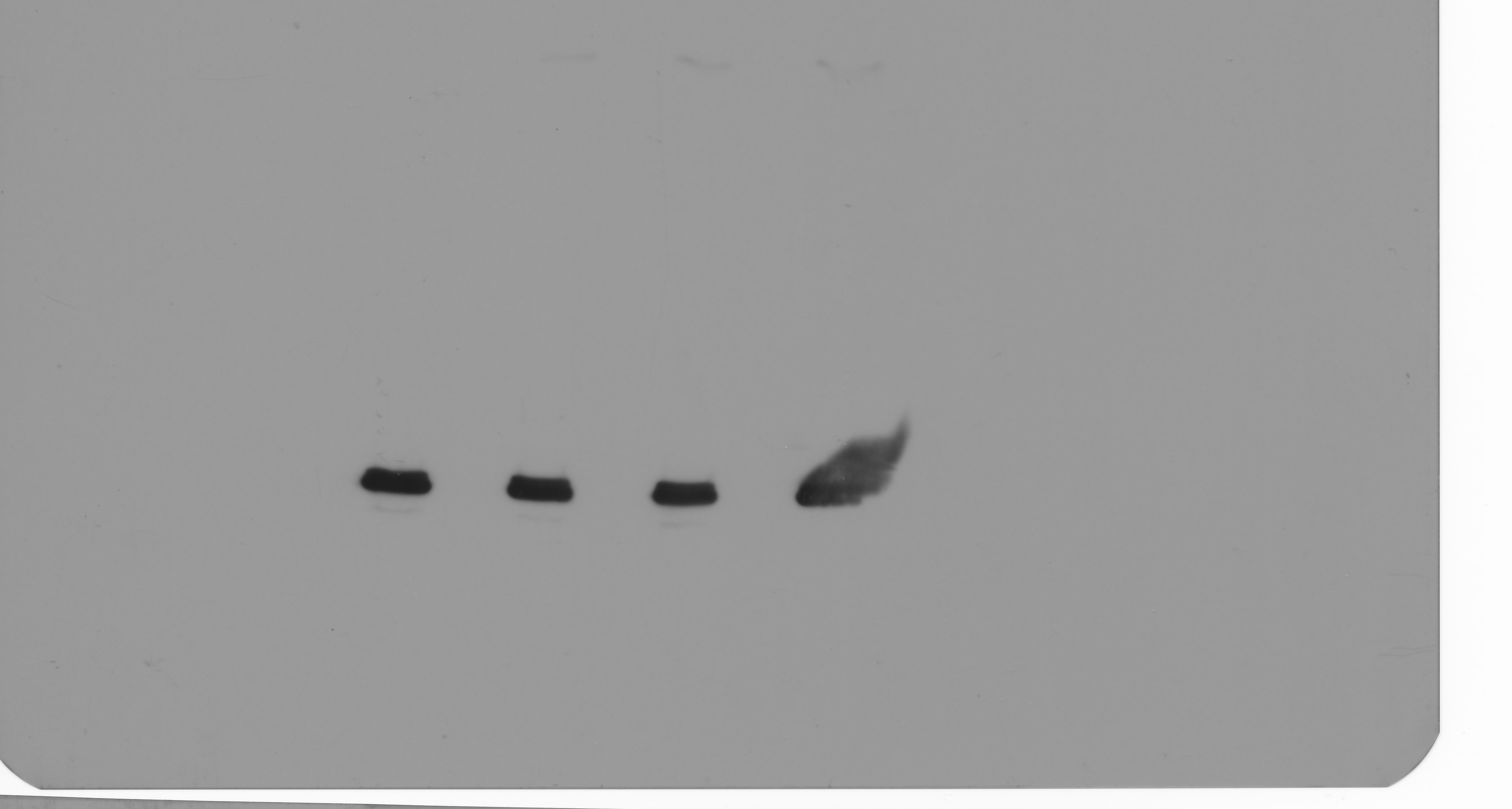

Supplement: Supplementary file 12 — Appendix Source Data [file 44319_2024_64_MOESM12_ESM.zip › Figure S2/2A/WCL IB RhoA.jpg]

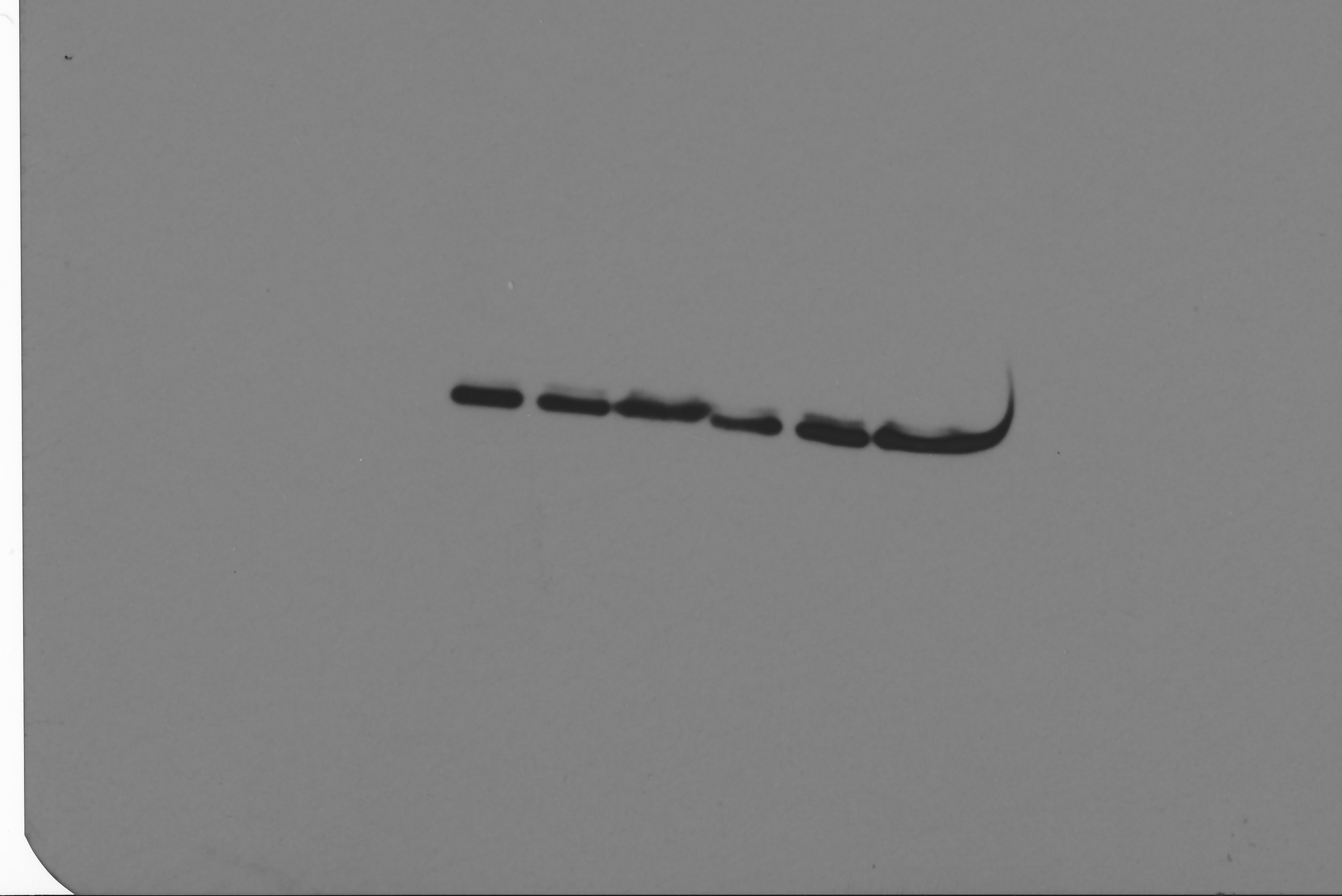

Supplement: Supplementary file 12 — Appendix Source Data [file 44319_2024_64_MOESM12_ESM.zip › Figure S2/2A/WCL IB RhoGDI.jpg]

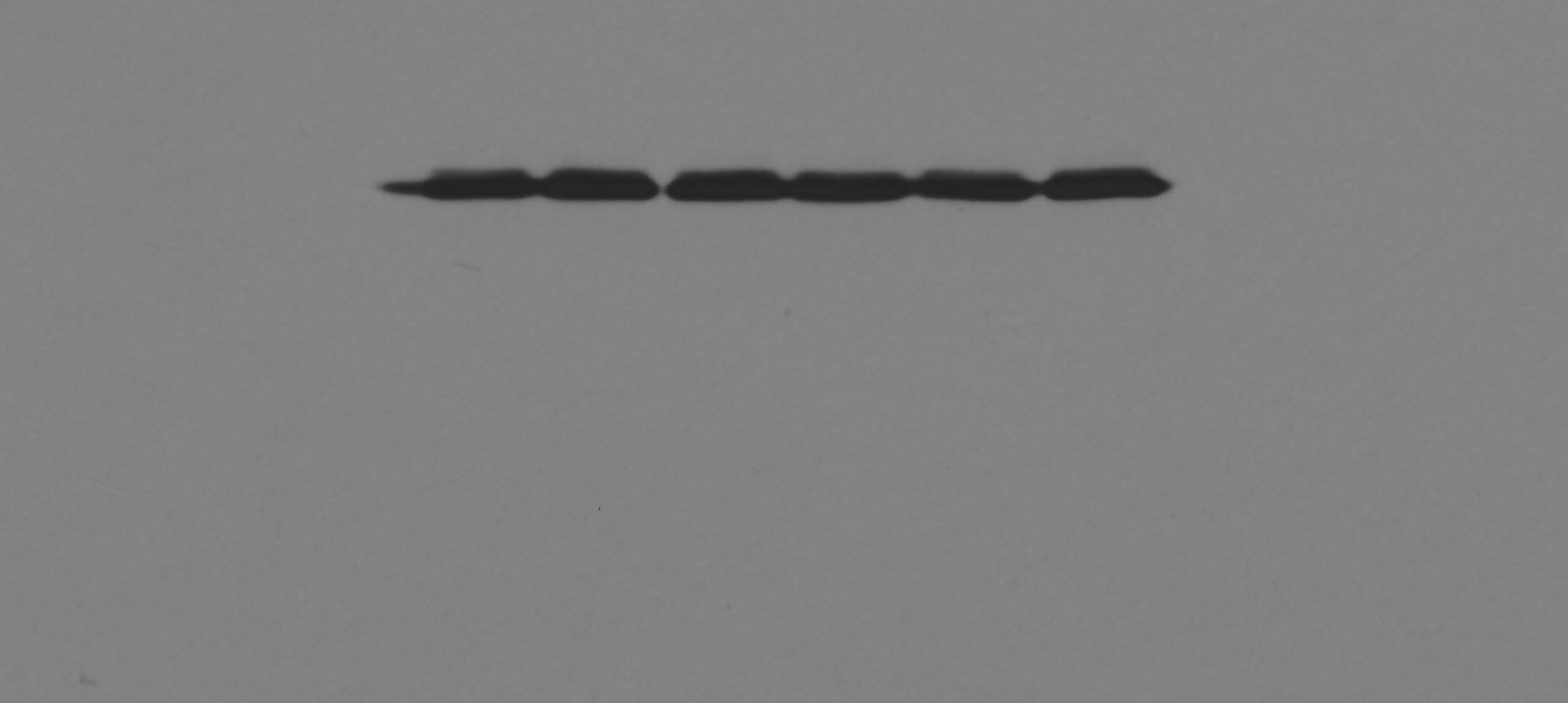

Supplement: Supplementary file 12 — Appendix Source Data [file 44319_2024_64_MOESM12_ESM.zip › Figure S2/2B/WCL IB Flag (RhoGDI).jpg]

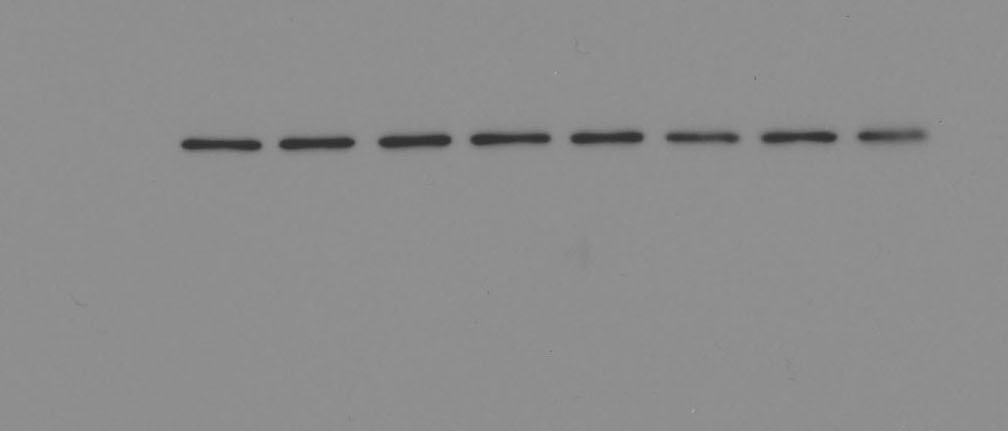

Supplement: Supplementary file 12 — Appendix Source Data [file 44319_2024_64_MOESM12_ESM.zip › Figure S2/2B/WCL IB GAPDH.jpg]

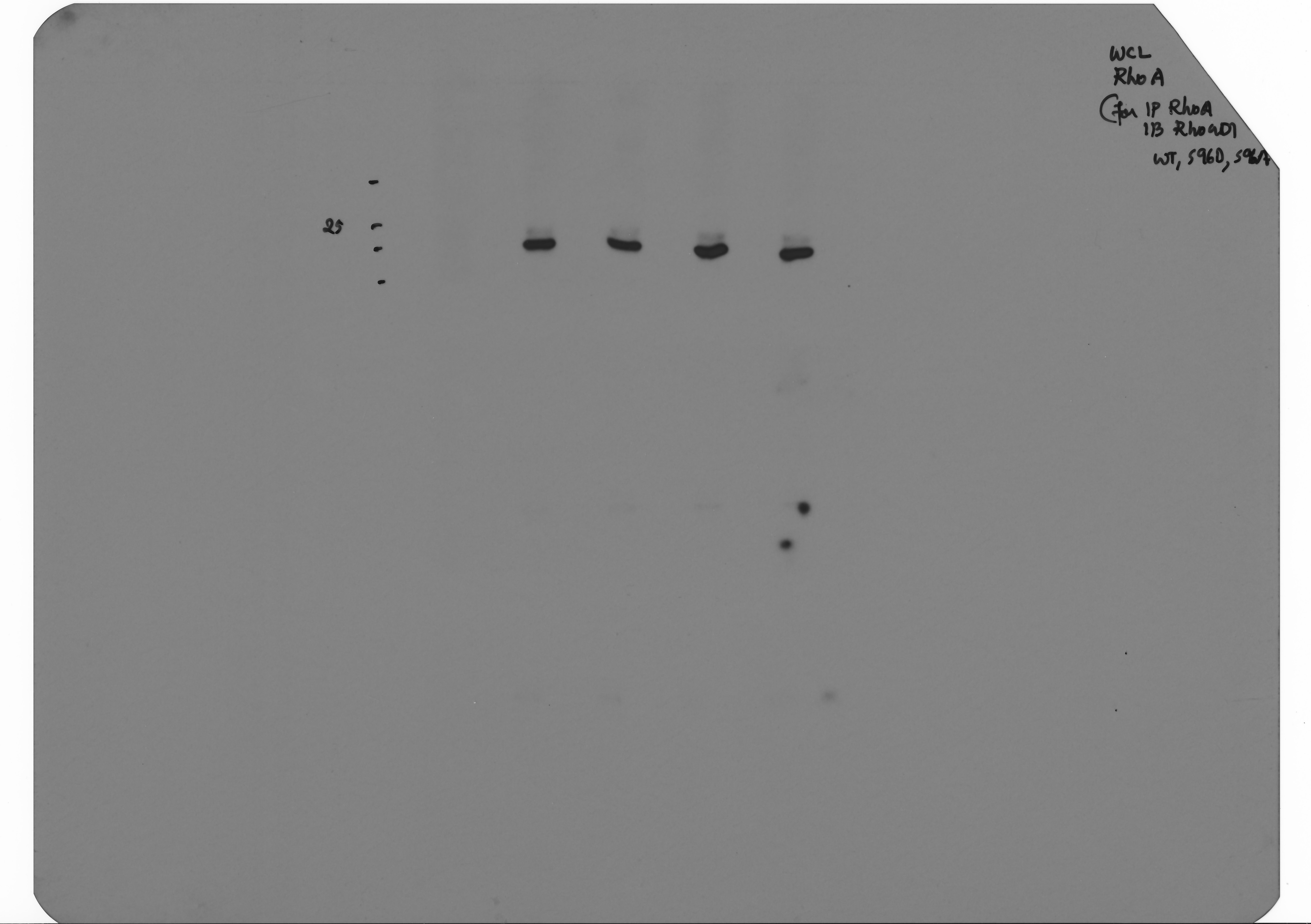

Supplement: Supplementary file 12 — Appendix Source Data [file 44319_2024_64_MOESM12_ESM.zip › Figure S2/2B/WCL IB HA (RhoA).jpg]

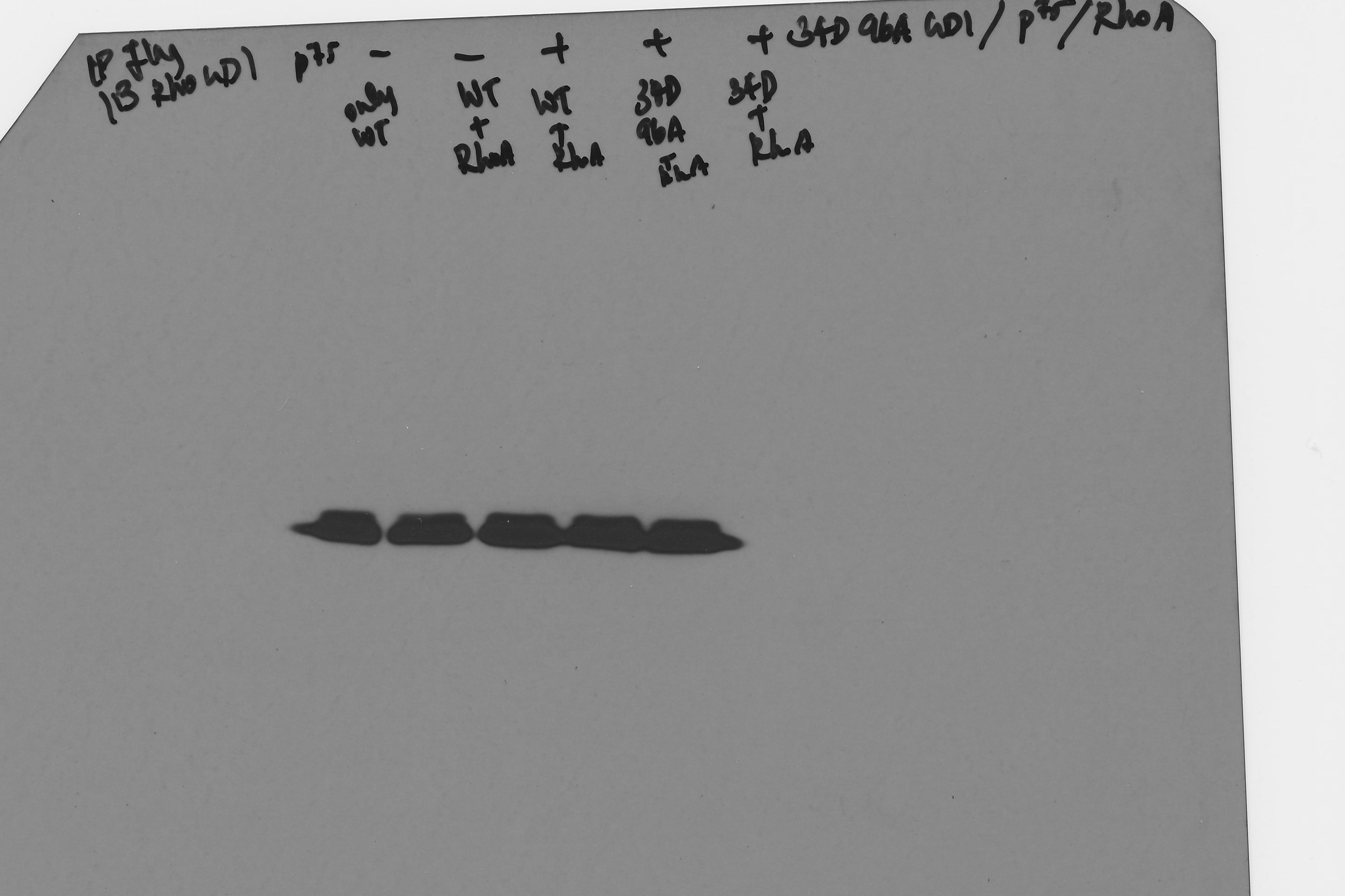

Supplement: Supplementary file 12 — Appendix Source Data [file 44319_2024_64_MOESM12_ESM.zip › Figure S2/2C/WCL IB Flag (RhoGDI).jpg]

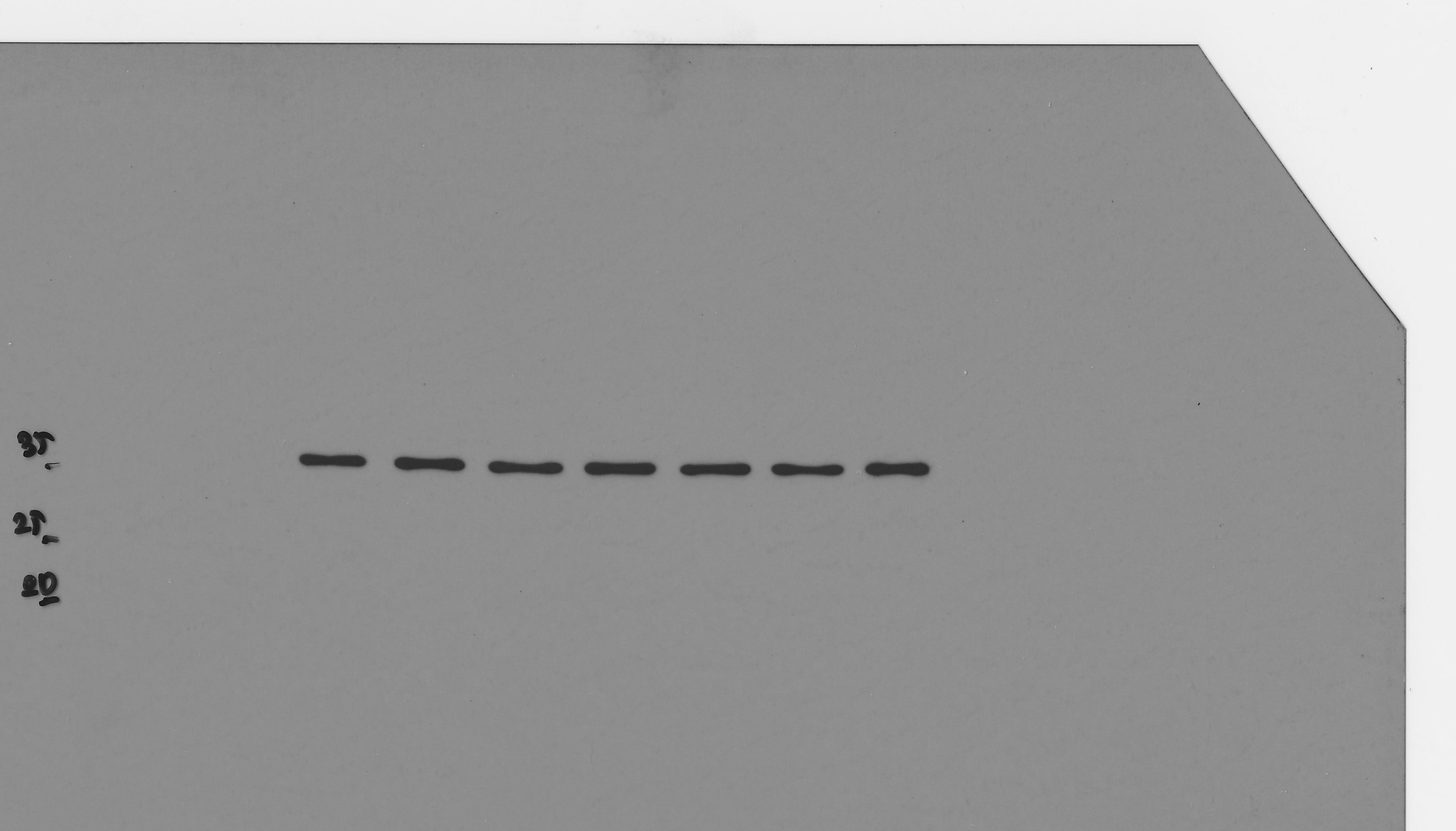

Supplement: Supplementary file 12 — Appendix Source Data [file 44319_2024_64_MOESM12_ESM.zip › Figure S2/2C/WCL IB GAPDH.jpg]

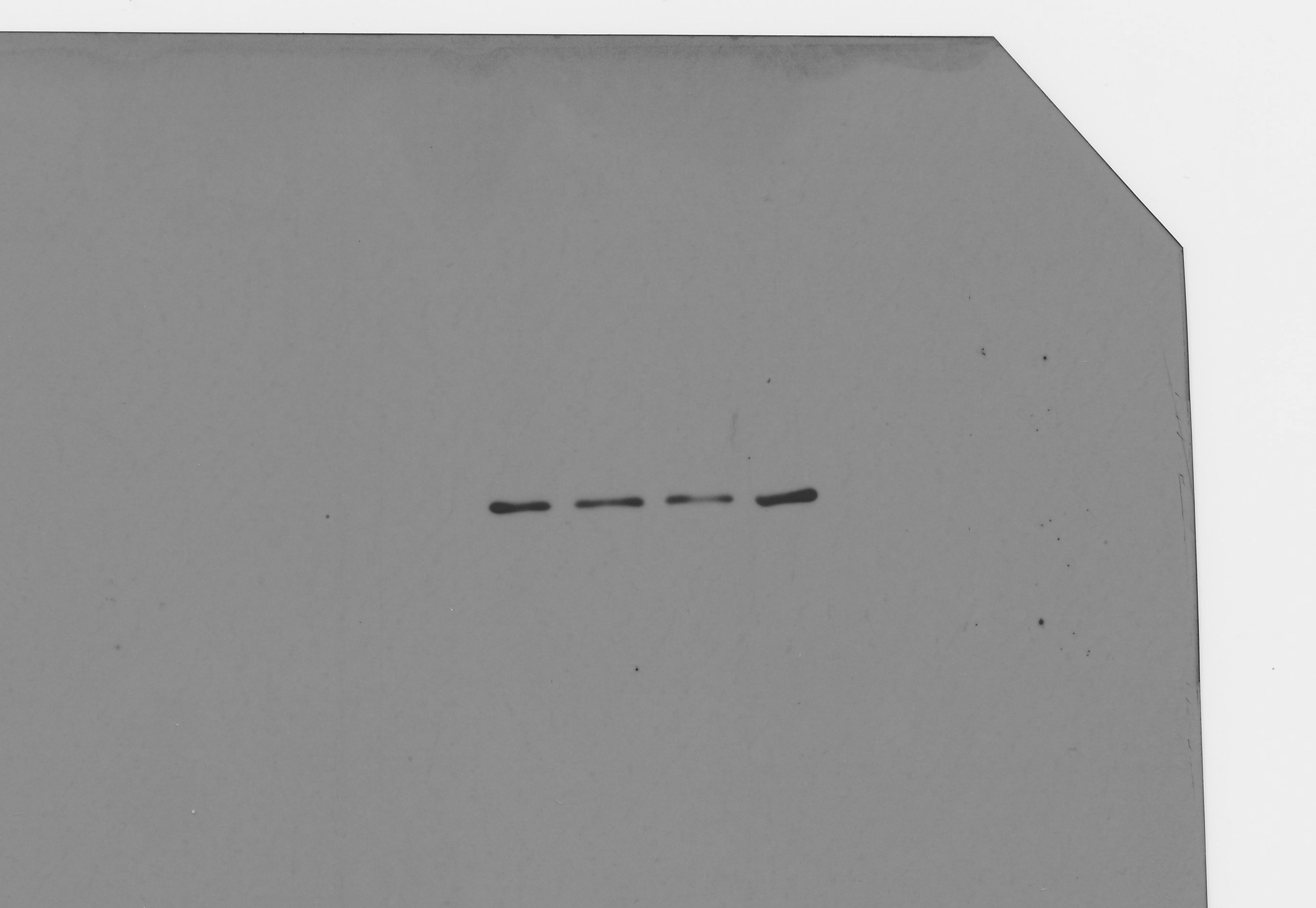

Supplement: Supplementary file 12 — Appendix Source Data [file 44319_2024_64_MOESM12_ESM.zip › Figure S2/2C/WCL IB HA (RhoA).jpg]

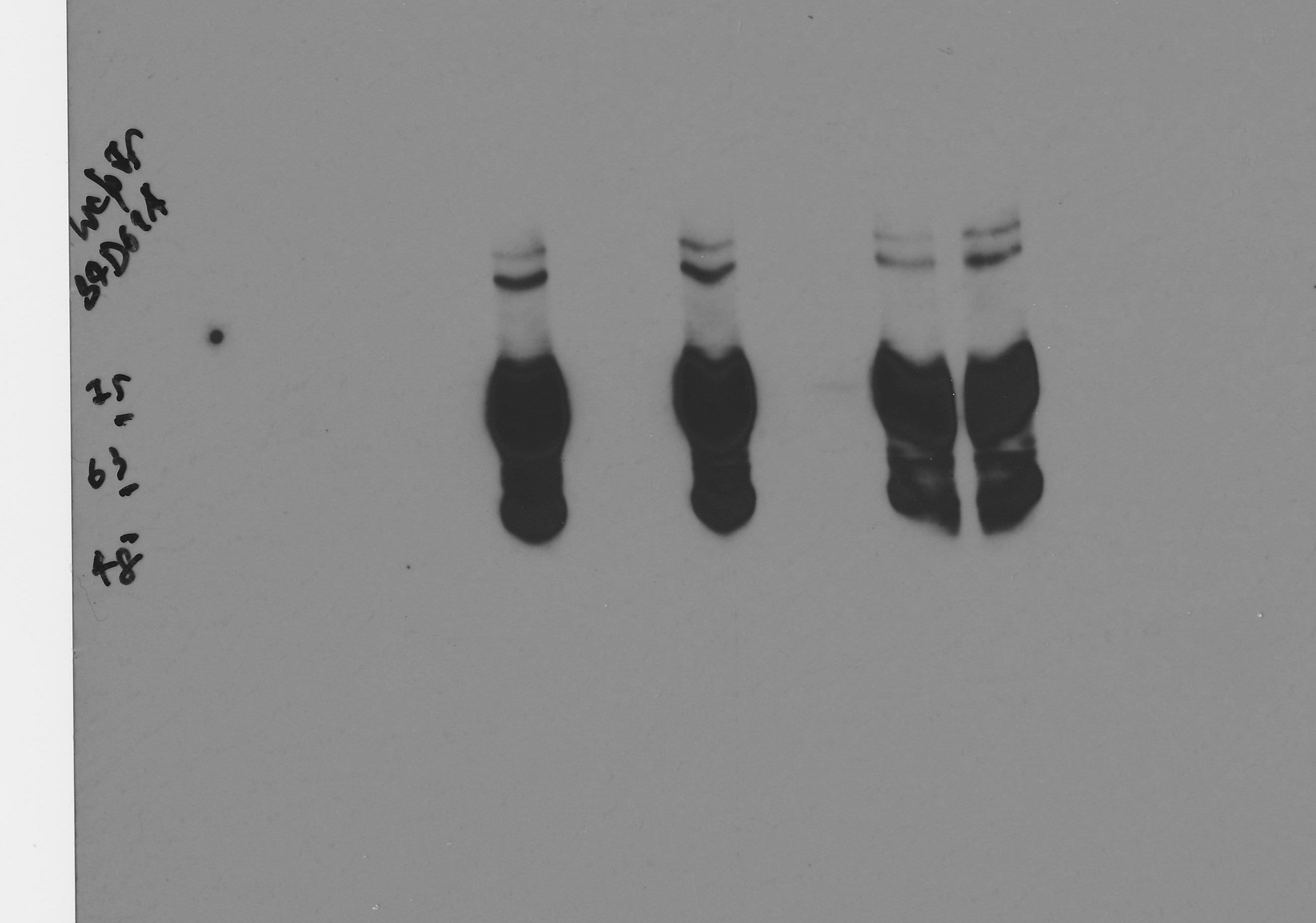

Supplement: Supplementary file 12 — Appendix Source Data [file 44319_2024_64_MOESM12_ESM.zip › Figure S2/2C/WCL IB P75NTR.jpg]

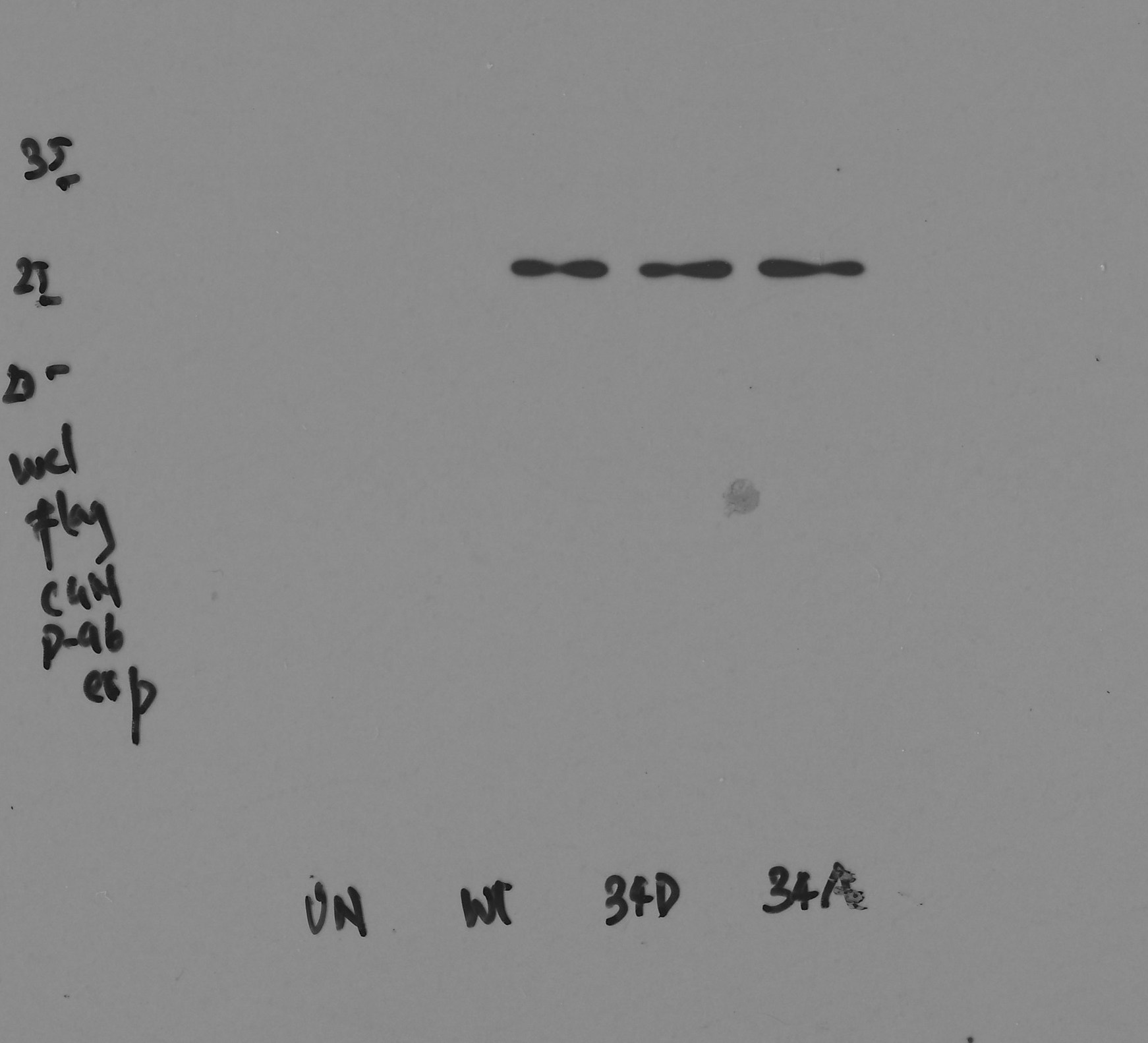

Supplement: Supplementary file 12 — Appendix Source Data [file 44319_2024_64_MOESM12_ESM.zip › Figure S2/2D/WCL IB Flag (RhoGDI).jpg]

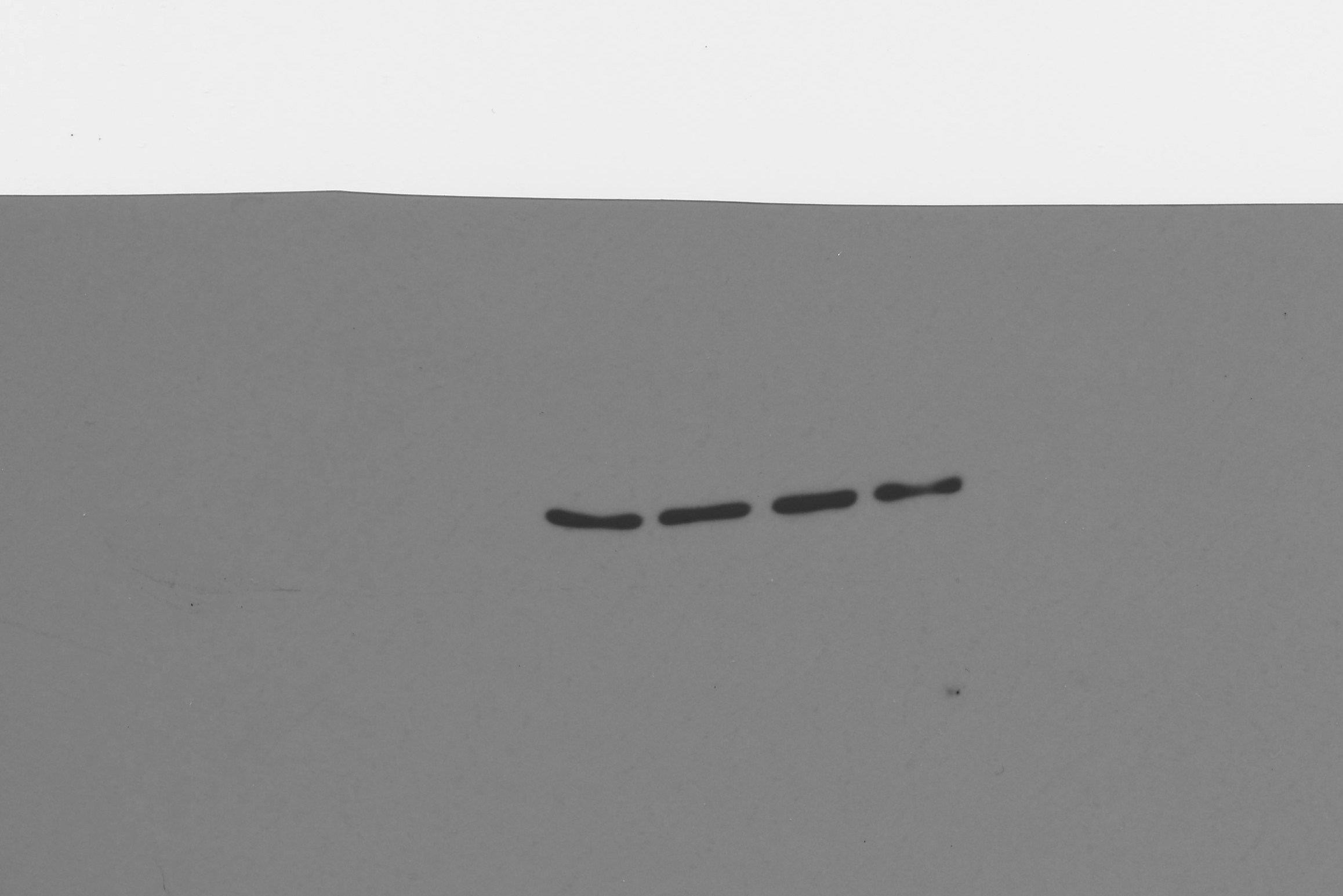

Supplement: Supplementary file 12 — Appendix Source Data [file 44319_2024_64_MOESM12_ESM.zip › Figure S2/2D/WCL IB GAPDH.jpg]

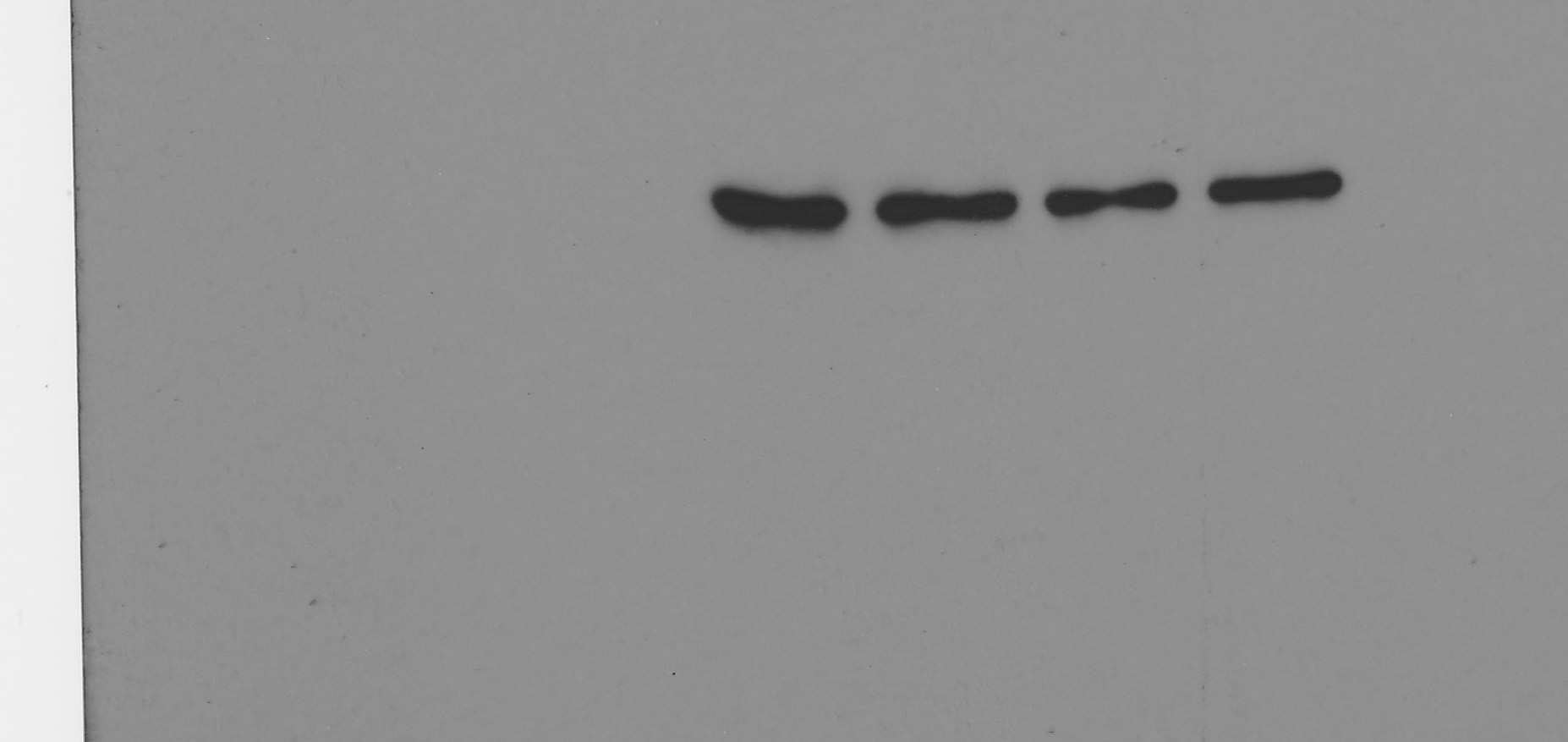

Supplement: Supplementary file 12 — Appendix Source Data [file 44319_2024_64_MOESM12_ESM.zip › Figure S2/2E/WCL IB GAPDH.jpg]

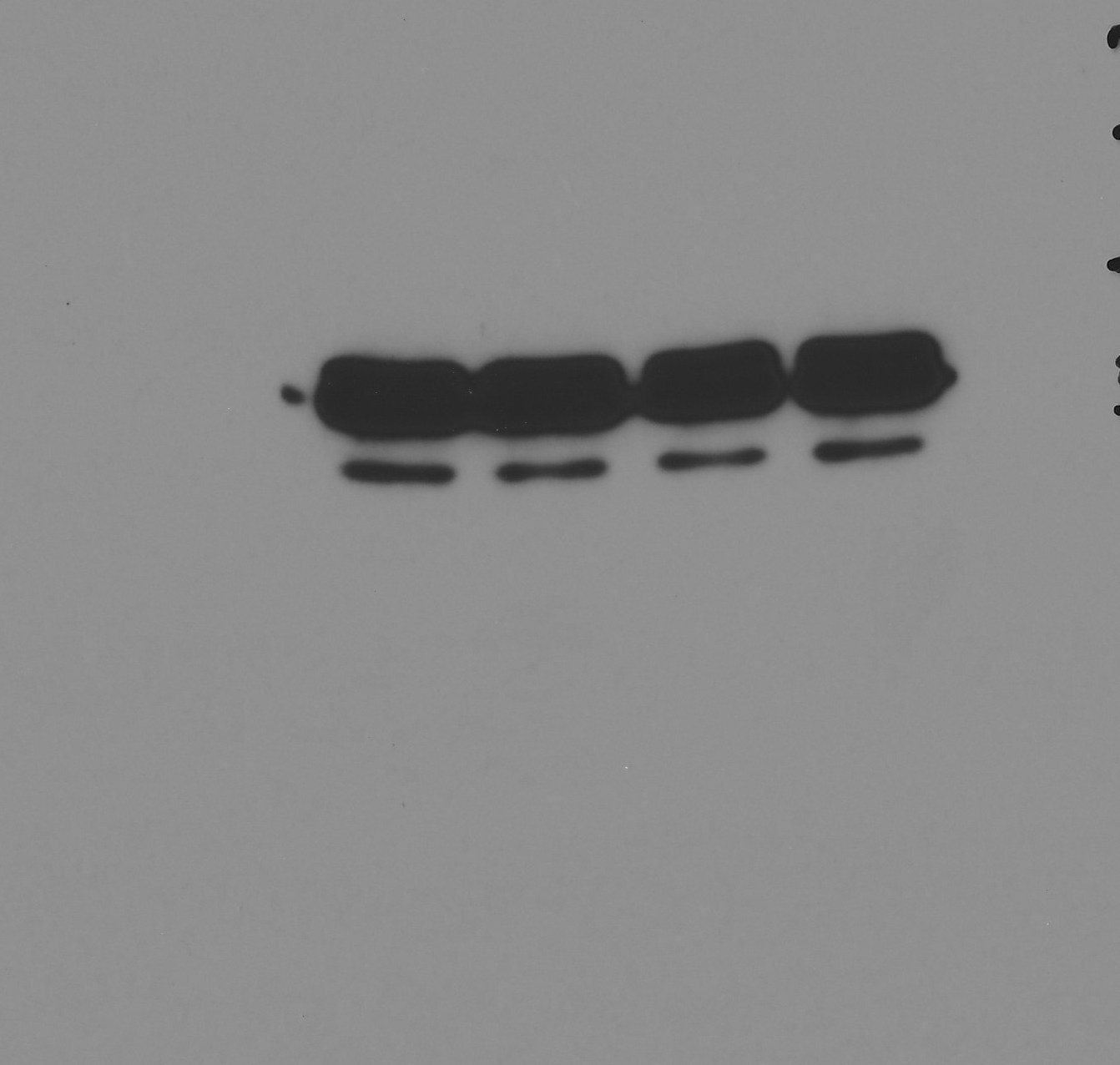

Supplement: Supplementary file 12 — Appendix Source Data [file 44319_2024_64_MOESM12_ESM.zip › Figure S2/2E/WCL IB RhoGDI.jpg]

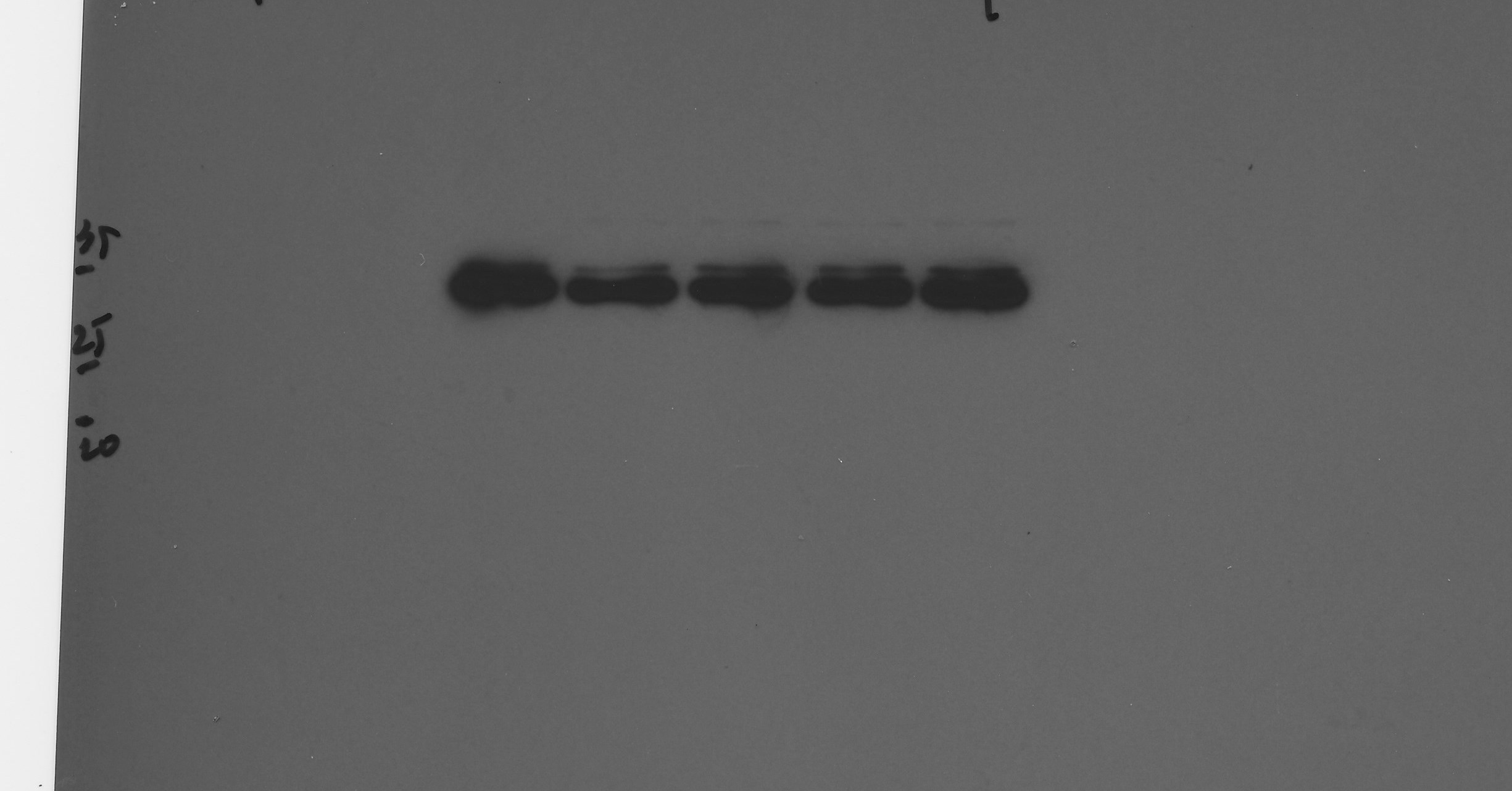

Supplement: Supplementary file 12 — Appendix Source Data [file 44319_2024_64_MOESM12_ESM.zip › Figure S2/2F/WCL IB GAPDH.jpg]

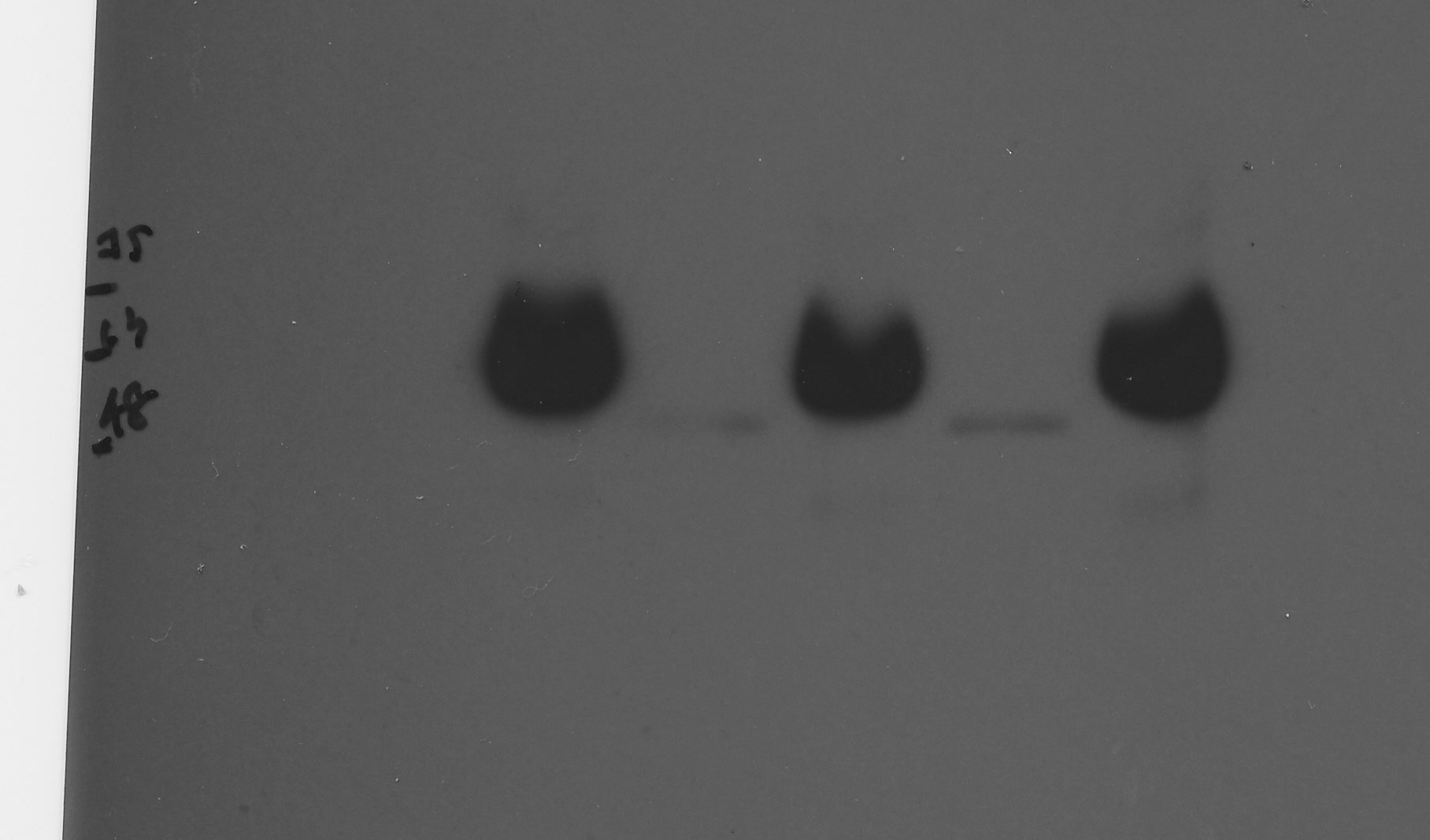

Supplement: Supplementary file 12 — Appendix Source Data [file 44319_2024_64_MOESM12_ESM.zip › Figure S2/2F/WCL IB p75NTR.jpg]

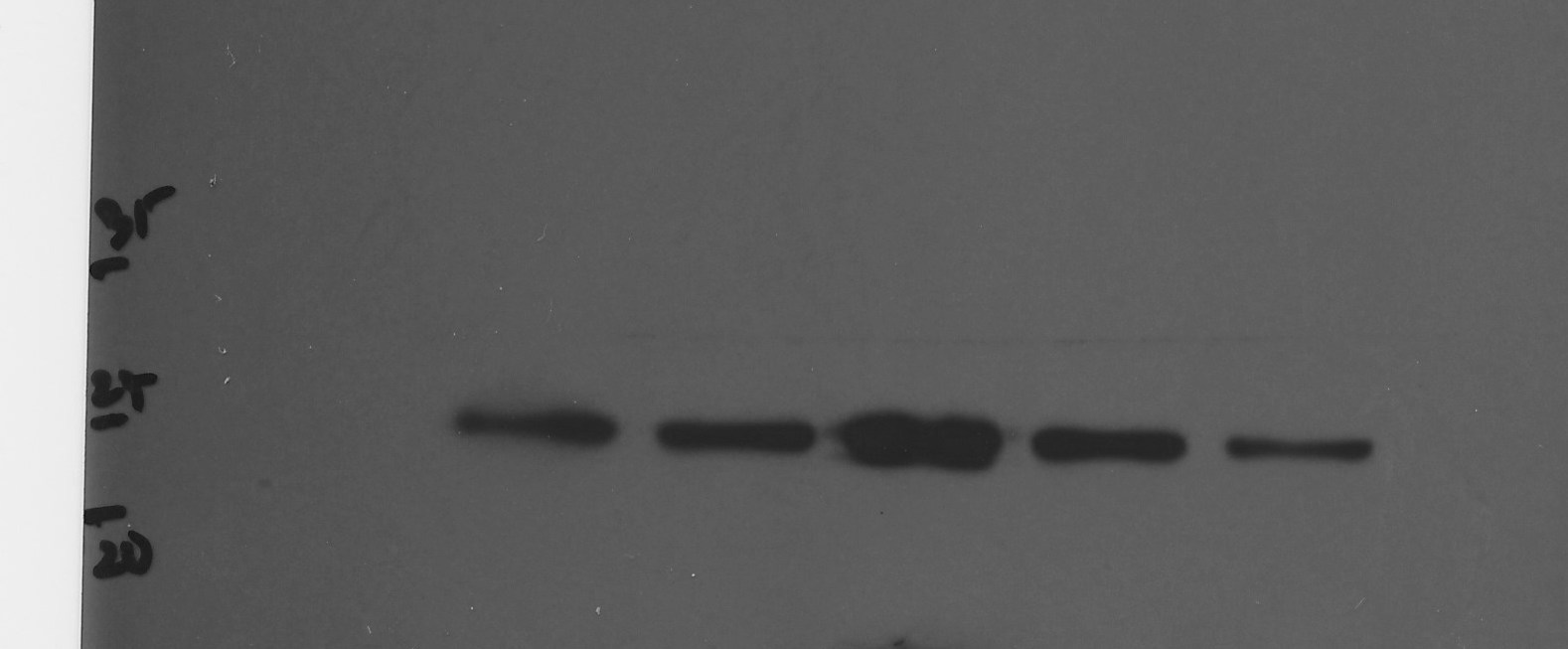

Supplement: Supplementary file 12 — Appendix Source Data [file 44319_2024_64_MOESM12_ESM.zip › Figure S2/2F/WCL IB RhoGDI.jpg]

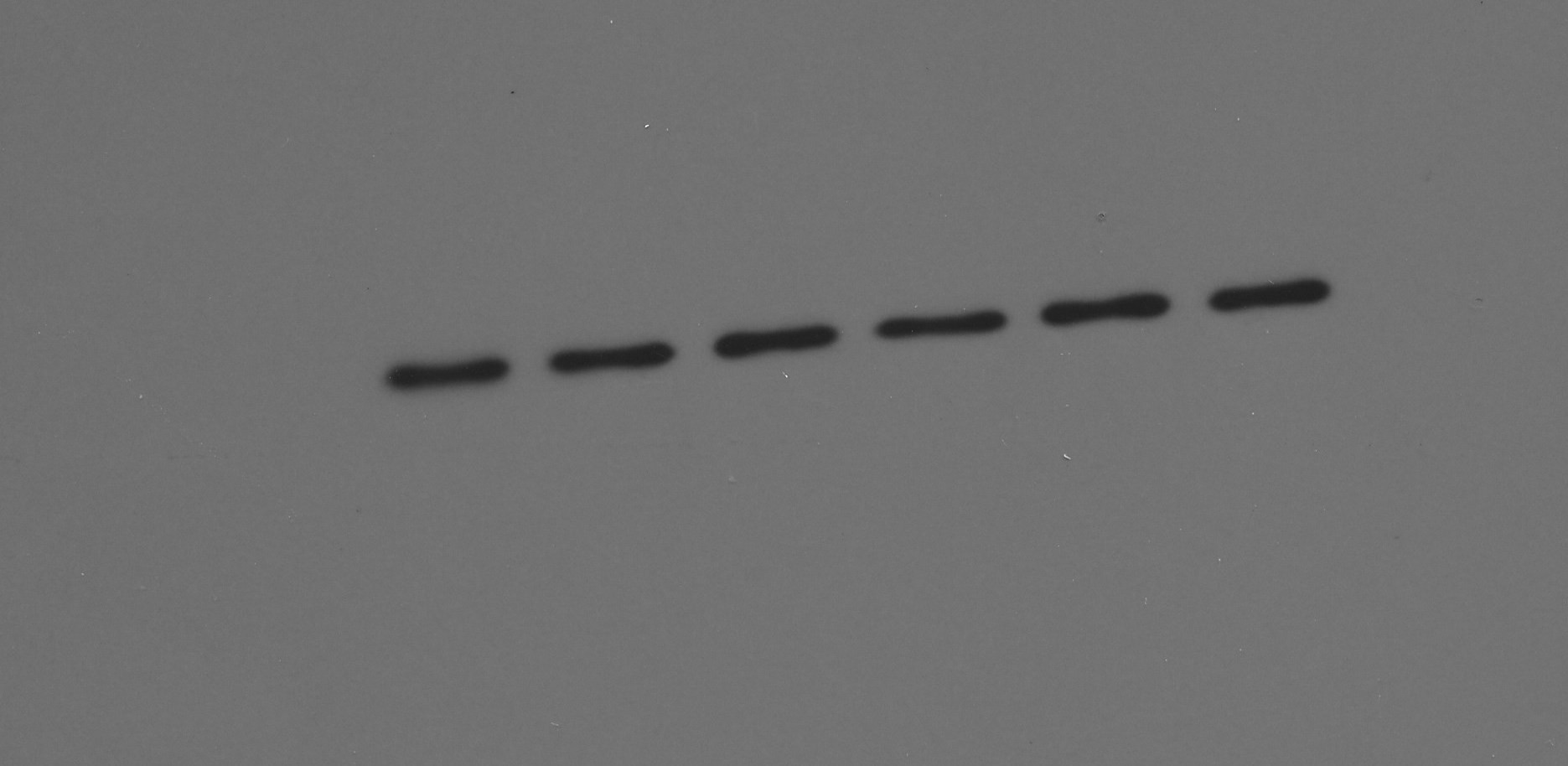

Supplement: Supplementary file 12 — Appendix Source Data [file 44319_2024_64_MOESM12_ESM.zip › Figure S2/2G/WCL IB Flag (RhoGDI).jpg]

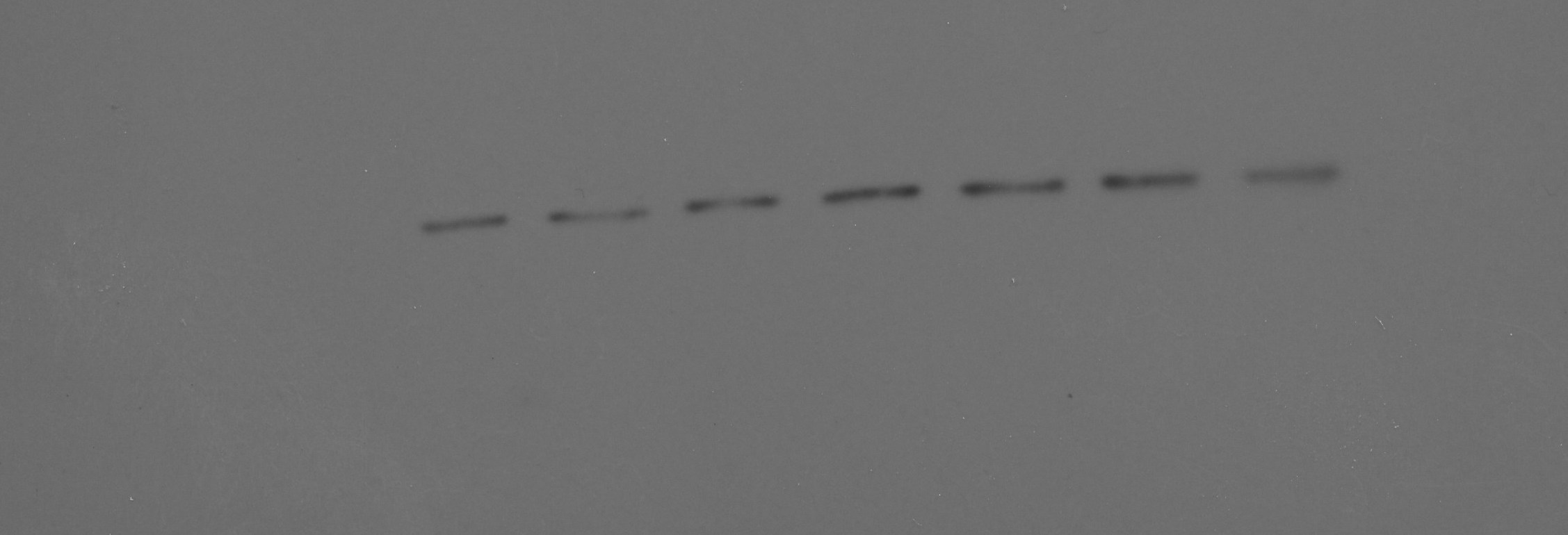

Supplement: Supplementary file 12 — Appendix Source Data [file 44319_2024_64_MOESM12_ESM.zip › Figure S2/2G/WCL IB GAPDH.jpg]

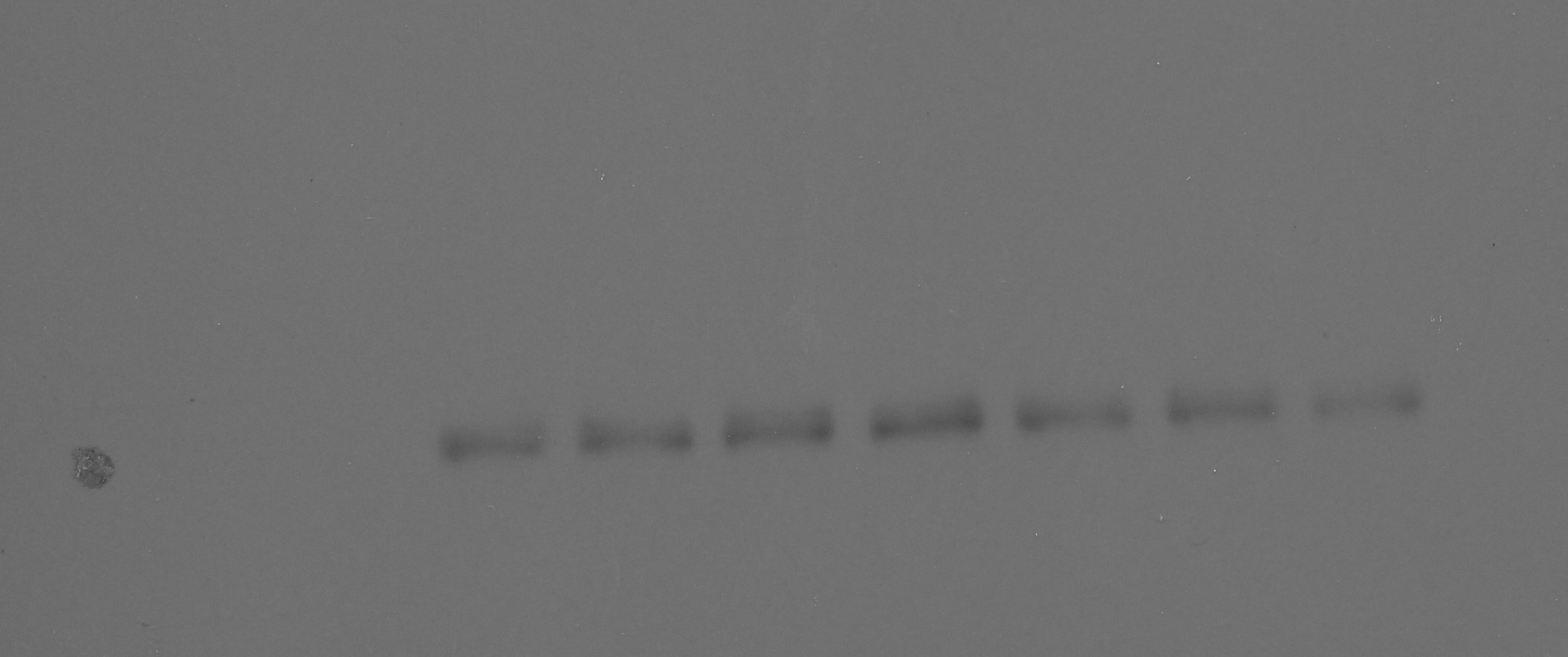

Supplement: Supplementary file 12 — Appendix Source Data [file 44319_2024_64_MOESM12_ESM.zip › Figure S2/2G/WCL IB p75NTR (endogenous).jpg]
