# Supplementary material for: RhoGDI phosphorylation by PKC promotes its interaction with death receptor p75NTR to gate axon growth and neuron survival
Source: EMBO Rep. 2024 Jan 22;25(3):30. doi: 10.1038/s44319-024-00064-2 (PMC10933337; doi:10.1038/s44319-024-00064-2)
Supplement: Supplementary file 14 — Expanded View Figures [file 44319_2024_64_MOESM14_ESM.pdf]

Expanded View Figures

Figure EV1. Solution structure of the complex between the p75^{NTR} JXT and RhoGDI NTD^{S34D}.

(**A**) SDS PAGE profiles of purified RhoGDI NTDS34D and p75NTR JXT domain. (**B**) [1H-15N] HSQC spectra of 15N-RhoGDI NTD in the absence (black) and presence (red) of p75^{NTR} JXT at 28 °C. The concentration of RhoGDI NTD and p75^{NTR} JXT was 0.3 mM and 1.5 mM, respectively. The backbone and side chain cross peaks undergoing significant chemical shift changes are labeled. * indicates unassigned cross-peak. (**C**) Representative slices from the 13C, 13C-filtered 3D NOESY spectrum. (**D**) Interaction diagram of complex interface created by LigPlot+. Amino acid residues involved in electrostatic interactions are shown as a ball-and-stick model, and residues involved in hydrophobic interactions are shown as an "eyebrow" shape. The black dashed line indicates the interaction interface and the green dashed line connects the residues involved in the electrostatic interactions. Molecule A represents RhoGDI NTDS34D, and B represents p75NTR JXT. (**E**) Schematic of *cis* and *trans* modes of RhoGDI interaction with the intracellular domains of the p75NTR dimer. (**F**) "Top view" of structural model of quaternary complex between p75^{NTR} death domain (DD, brown) and RhoGDI (cyan). The model is based on the solution structure of the RhoGDI CTD bound to p75^{NTR} DD (Lin 2015) and the structure of the full-length RhoGDI (from PDB ID:4F38). Arrows denote the spatial proximity of the RhoGDI N-terminal domain (NTD) to the N-terminus (N) of the p75^{NTR} DD that engages the C-terminal domain (CTD) of the second RhoGDI protomer. (**G**) Crystal structure of RhoGDI:RhoA complex (PDB ID: 4F38). Ser34 from the RhoGDI NTD is in close proximity to Glu121 from the RhoGDI CTD. The distances between side chain oxygen atoms of these two residues are ~5 Å.

© The Author(s)

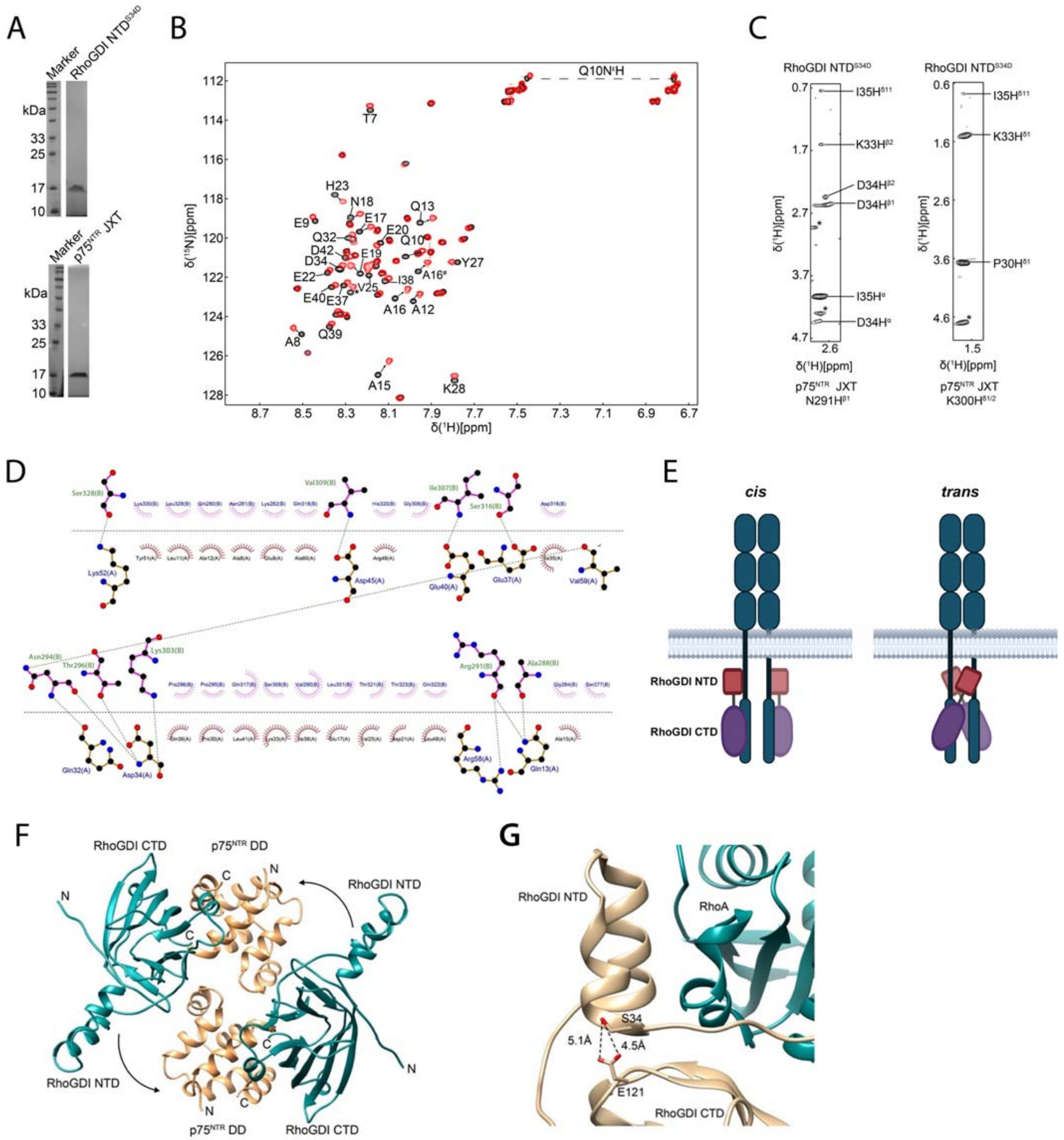
A
Marker
RhoGDI NTD S34D
kDa
33
25
17
10
Marker
p75NTR JXT
kDa
33
25
17
10
B
Q10NεH
δ(15N)[ppm]
δ(1H)[ppm]
T7
H23
N18
E9
E17
Q13
E20
Q32
Q10
D42
E19
D34
Y27
E22
I38
A16#
V25
E40
E37
A16
A12
Q39
A8
A15
K28
C
RhoGDI NTD S34D
I35Hδ11
K33Hβ2
D34Hβ2
D34Hβ1
I35Hα
D34Hα
δ(1H)[ppm]
p75NTR JXT
N291Hβ1
RhoGDI NTD S34D
I35Hδ11
K33Hδ1
P30Hδ1
p75NTR JXT
K300Hδ1/2
D
E
cis
trans
RhoGDI NTD
RhoGDI CTD
F
RhoGDI CTD
p75NTR DD
RhoGDI NTD
RhoGDI NTD
p75NTR DD
RhoGDI CTD
G
RhoGDI NTD
RhoA
S34
5.1Å
4.5Å
E121
RhoGDI CTD

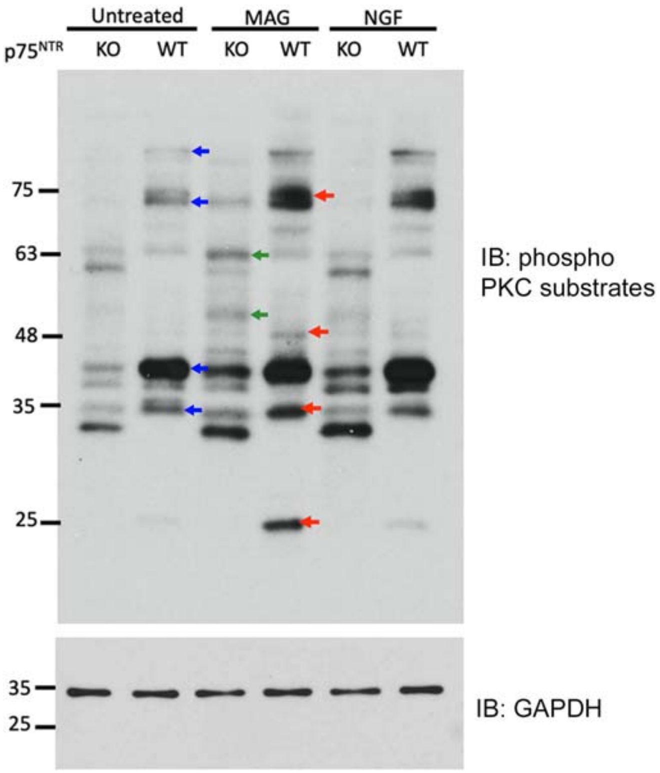

Figure EV2. Western blot of phospho PKC substrates in total CGN extracts.

Western blot of phospho PKC substrates in total cell extracts of P7 p75^{NTR} knock out (KO) or p75NTR wild type (WT) CGNs following treatment with MAG (25ug/ml) or NGF (100 ng/ml) for 30 min. Blue arrows denote protein species showing de-novo or enhanced phosphorylation in CGNs expressing p75^{NTR} compared to KO cells. Green arrows denote species showing increased phosphorylation after MAG treatment independently of p75^{NTR}. Red arrows denote species showing increased phosphorylation after MAG treatment in a p75^{NTR}-dependent manner.
